# Genes Expressed in Specific Areas of the Human Fetal Cerebral Cortex Display Distinct Patterns of Evolution

**DOI:** 10.1371/journal.pone.0017753

**Published:** 2011-03-18

**Authors:** Nelle Lambert, Marie-Alexandra Lambot, Angéline Bilheu, Valérie Albert, Yvon Englert, Frédérick Libert, Jean-Christophe Noel, Christos Sotiriou, Alisha K. Holloway, Katherine S. Pollard, Vincent Detours, Pierre Vanderhaeghen

**Affiliations:** 1 Institut de Recherches en Biologie Humaine et Moléculaire (IRIBHM), Université Libre de Bruxelles (ULB), Brussels, Belgium; 2 Hôpital Universitaire des Enfants Reine Fabiola, Child Psychiatry Department, Brussels, Belgium; 3 Department of Obstetrics and Gynaecology, Université Libre de Bruxelles (ULB), Brussels, Belgium; 4 Department of Pathology, Erasme Hospital, Université Libre de Bruxelles (ULB), Brussels, Belgium; 5 Bordet Cancer Institute, Université Libre de Bruxelles (ULB), Brussels, Belgium; 6 Gladstone Institutes, University of California San Francisco, San Francisco, California, United States of America; 7 Division of Biostatistics & Institute for Human Genetics, University of California San Francisco, San Francisco, California, United States of America; Virginia Industries for the Blind, Belgium

## Abstract

The developmental mechanisms through which the cerebral cortex increased in size and complexity during primate evolution are essentially unknown. To uncover genetic networks active in the developing cerebral cortex, we combined three-dimensional reconstruction of human fetal brains at midgestation and whole genome expression profiling. This novel approach enabled transcriptional characterization of neurons from accurately defined cortical regions containing presumptive Broca and Wernicke language areas, as well as surrounding associative areas. We identified hundreds of genes displaying differential expression between the two regions, but no significant difference in gene expression between left and right hemispheres. Validation by qRTPCR and *in situ* hybridization confirmed the robustness of our approach and revealed novel patterns of area- and layer-specific expression throughout the developing cortex. Genes differentially expressed between cortical areas were significantly associated with fast-evolving non-coding sequences harboring human-specific substitutions that could lead to divergence in their repertoires of transcription factor binding sites. Strikingly, while some of these sequences were accelerated in the human lineage only, many others were accelerated in chimpanzee and/or mouse lineages, indicating that genes important for cortical development may be particularly prone to changes in transcriptional regulation across mammals. Genes differentially expressed between cortical regions were also enriched for transcriptional targets of *FoxP2*, a key gene for the acquisition of language abilities in humans. Our findings point to a subset of genes with a unique combination of cortical areal expression and evolutionary patterns, suggesting that they play important roles in the transcriptional network underlying human-specific neural traits.

## Introduction

The cerebral cortex has acquired or expanded a variety of specific features during evolution of the primate lineage. These include a larger relative size but also many important qualitative differences [1], [2], [3], [4], [5], [6]. The human cortex displays an increased size and number of specific associative areas, and extensive specialization of some areas such as the language areas of Broca and Wernicke [7], [8]. These two areas also display a high degree of lateralization, with most language processing activities being localized in the left hemisphere [9].

What are the mechanisms underlying the emergence of specific features in the human cortex? It seems likely that the most dramatic changes in human brain evolution are related to specific early developmental events [2], [3]. For instance, the overall increase in cortical surface in primates may be linked to species-specific features of cortical progenitors [2], [4], [10], [11], [12], [13]. However, the developmental mechanisms underlying the species-specific patterning and diversity of cortical areas remain essentially unknown.

Recent studies have started to characterize the transcriptome of the human fetal cortex, thus identifying genes differentially expressed between distinct domains [14], [15] or displaying lateralized expression in the embryonic cortex [16], [17]. These studies have also uncovered high prevalence of alternative splicing among genes differentially expressed in the fetal cortex, as well as frequent association with human-specific evolution of their putative cis-regulatory elements or coding sequences [14], [17]. On the other hand, computational analyses have identified genes and putative transcriptional regulatory elements with signatures of positive selection in the primate lineage [3], [18], [19], [20], [21]. Among these, the so-called HAR (human accelerated regions) or haCNS (human accelerated conserved non coding sequences) elements are highly conserved non-coding regions that have changed rapidly along the human lineage and are thought to correspond mainly to regulatory elements that could contribute to species-specific transcriptional programs [18], [19], [22], [23]. Genomic studies have also uncovered potential changes in transcription factors that could regulate the development of features specific to the human brain. These include *FOXP2*, a gene required for the acquisition of language skills, which displays several human-specific molecular features [24].

Here we developed a novel approach combining three-dimensional reconstruction of human fetal brain and expression profiling, to define the transcriptome of neurons from accurately defined cortical regions containing presumptive language areas of Broca and Wernicke. Analysis of transcriptional patterns and evolutionary signatures of genes active in these areas uncovered a novel set of cortical genes displaying differential expression plus divergent evolution in their regulatory regions. These genes are promising candidates for discovering the genetic framework underlying the acquisition of human-specific neural traits.

## Results

### Using three-dimensional reconstruction of human fetal brain tissue to probe the transcriptome of specific cortical areas

In order to gain insight into the development of human-specific features of the cerebral cortex, we investigated gene expression patterns in cortical domains that contain areas thought to have undergone significant divergence during primate evolution. We focused on the presumptive language areas of Broca and Wernicke, as well as surrounding associative areas of the frontal and parieto-temporal cortex.

**Figure 1:**
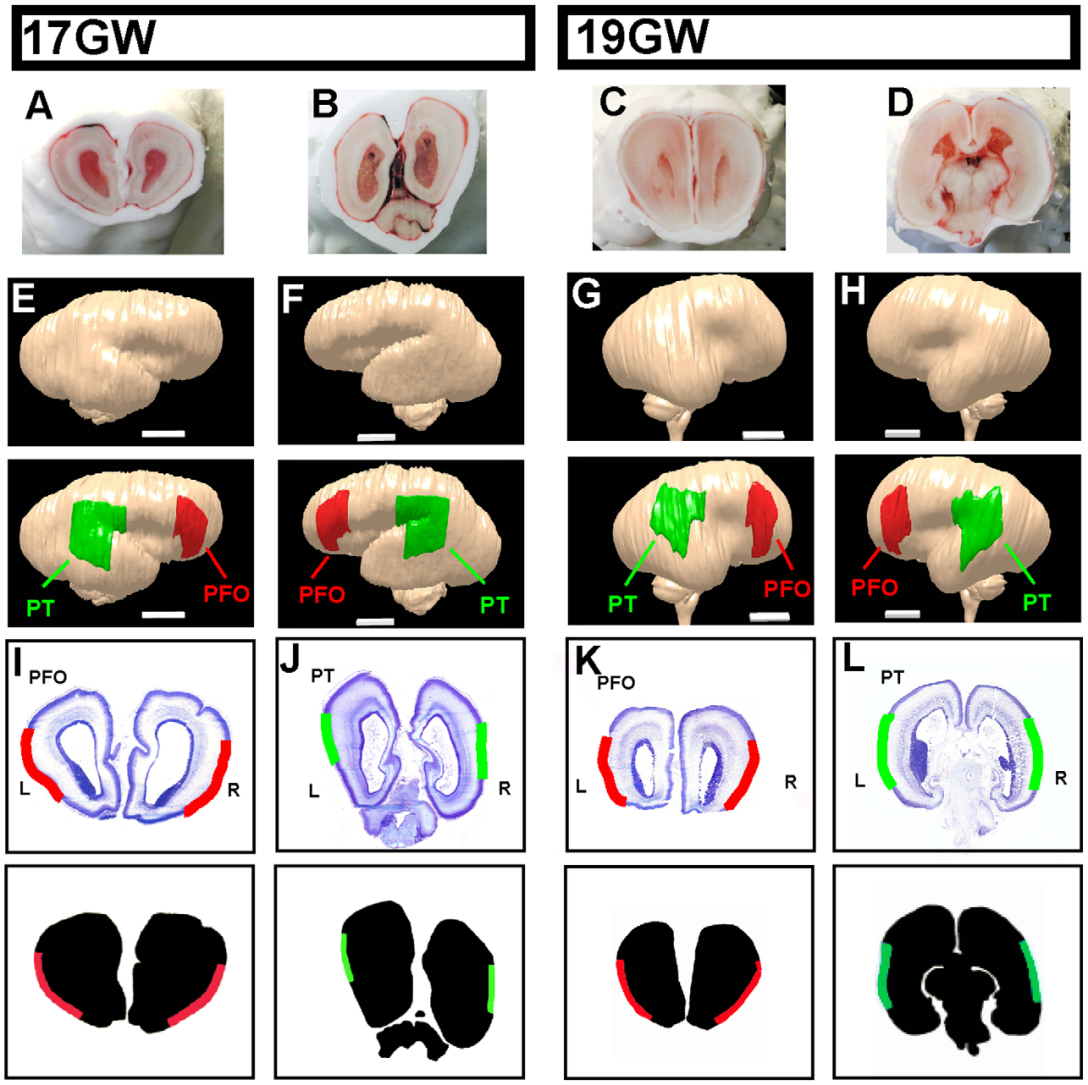
Neuroembryological reconstruction and microdissection procedures. (A–D) Examples of brain cryosection pictures used for the 3D reconstruction. (E–H) 3D reconstruction models of 17 (E, F) and 19 (G, H) GW, with and without highlighted dissected regions PFO (red) and PT (green). (I–L) Microdissected regions of PFO (I, K) and PT (J, L) areas at 17 (I, J) and 19 (K, L) GW, highlighted on top of cresyl violet-stained sections. Scale bar: 1 cm.

Two major challenges in this approach are to determine with precision the boundaries of the territories of interest, and to isolate them in a reproducible fashion from different tissue samples. To tackle these problems, we developed a procedure whereby human fetal brains of two developmental stages (17 and 19 gestational weeks (GW)) were processed as a whole, without any prior dissection. First, the brains were freshly frozen and cryosectioned (Figure 1A–D). Photographed sections were then used to reconstruct a three-dimensional (3D) model of each brain, which allowed us to visualize with great accuracy the areas of interest (Figure 1E–H). In order to obtain comparable cortical areas between different brains at different developmental stages, we performed a ‘morphing’ of each 3D model, thereby allowing a precise delineation of the same cortical domains in different samples at different developmental stages. These were used to determine two cortical regions of interest: one (which we named PFO), corresponding to parts of the prefrontal/frontal/orbito-frontal cortex and containing presumptive Broca area and surrounding associative areas, and the other (named PT), corresponding to parts of parieto-temporal cortex and containing presumptive Wernicke area and surrounding associative areas (Figure 1E–L). The corresponding tissues were then microdissected on the cryosections using templates generated from the 3D reconstructions (Figure 1I–L). Importantly, this method enabled us to collect selectively the tissue corresponding to the cortical plate, the region containing only post-migratory cortical neurons, and thus excluding cortical progenitors and migrating neurons from our samples (Figure 1I–L). Finally, mRNA was extracted from the corresponding tissue and profiled by conventional microarray analyses.

### Gene expression varies with cortical area but not with lateralization

We compared gene expression patterns in the context of three main parameters: the area dissected (PFO vs. PT), lateralization (left vs. right), and gestational age (17 vs. 19 GW). Due to the very limited availability of human fetal brain samples, each of these comparisons is based on eight tissue samples from two individuals. Thus, our power to reliably detect differential expression is *a priori* low. We therefore conducted simple, robust statistical analyses with strict significance thresholds. While this approach may miss some truly differentially expressed genes, we expected the detectable differences to be reliable. These expectations were confirmed by the relatively low number of differentially expressed genes that we identified, the high concordance observed between the data obtained from the two individuals, and the large proportion of these that we validated in independent, low throughput experiments (see below). Since samples at different gestational ages were dissected from different individuals, gene expression associated with this variable also corresponds to inter-individual variability and must be interpreted accordingly.

**Figure 2:**
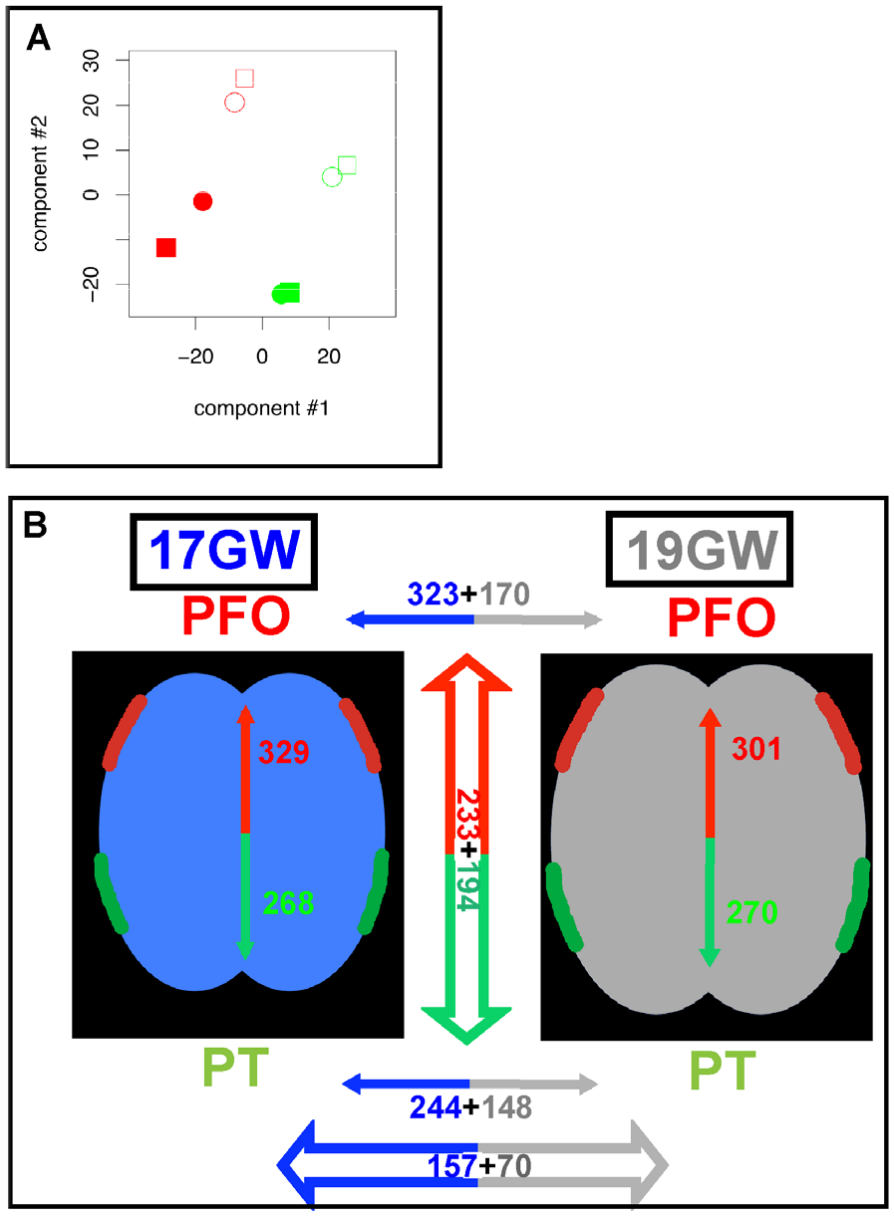
Primary microarray analysis. (A) Principal components analysis. Expression profiles are plotted in the space of the 1^st^ and 2^nd^ principal components, which account for 40% and 37% of the total variance, respectively. Red/green depicts samples from PFO (red) and PT (green) areas; fill/empty symbols, 17/19 GW; circles/square symbols, right/left. (B) Summary of gene profiling results depicting the number of genes displaying a fold change higher than 1.5 for found at 17 or 19 GW (PFO>PT in red; PT>PFO in green); genes displaying a fold change higher than 1.5 for PFO vs. PT at both 17 GW and 19 GW (intersection of genes displaying differential regulation between PFO and PT at either 17 or 19 GW)) are depicted within the central vertical thick arrow. The number of genes displaying a fold change higher than 1.5 for 17 vs. 19 GW found in PFO or PT (17GW>19GW in blue; 17GW<19GW in gray); genes displaying a fold change above 1.5 for 17 vs. 19 in both PFO and PT are shown in the bottom blue and gray thick arrow.

In order to visualize the dominant trends of global gene expression, we used principal components analysis (Figure 2A). Projecting gene expression variation onto the first two principal components, clear separation lines could be drawn between samples from different cortical areas. Samples from different developmental stages or individuals were also clearly separated. Importantly, there was no separation between left and right samples, even when including the third principal component (not shown). Hence, little expression variation, if any, can be explained by the lateralization axis compared to variations related to cortical area or gestational age/inter-individual variation.

We then investigated whether the expression of individual genes varies between cortical areas, hemispheres, and gestational age/individuals. Using the Significance Analysis of Microarray package [25], we detected (at multiple testing-corrected significance q<0.01) 1274 genes differentially expressed when comparing the PFO and PT areas (PFO vs. PT) and 1763 genes when comparing samples at 17 and 19 GW individuals (17 vs. 19 GW). In contrast, no genes were significantly differentially expressed when comparing the right- and left-side samples. These findings are consistent with our principal components analyses, where we observed high between-sample variation between cortical areas and time points, but not between hemispheres. We note, however, that we cannot rule out subtle expression variations along the lateralization axis that are missed due to low statistical power, with just four observations per hemisphere. Nonetheless, the lack of differential gene expression between left- and right-hemispheres is in agreement with previous reports suggesting that differential expression between sides of the brain is mainly a feature of much earlier developmental stages [14], [15], [16]. Since variation is much smaller, if not null, along the lateralization axis, right- and left-side expression profiles were averaged within cortical area or gestational age. We thus examined the magnitude of individual genes' expression changes using fold-change. We found 157 genes expressed at least 1.5 times higher at 17 compared to 19 GW in both the PFO and PT areas. By comparison, 70 genes were at least 1.5-fold downregulated at 17 versus 19 GW in both the PFO and PT areas. Comparing the PFO to the PT, 233 genes were upregulated and 194 were downregulated at both 17 and 19 GW (Figure 2). The concordance observed between the 17 and 19 GW samples was remarkably high, as the vast majority of genes regulated in PFO vs. PT in the 17 GW samples were found to be similarly regulated in the 19GW sample, and vice-versa (Figure 2B).

**Figure 3:**
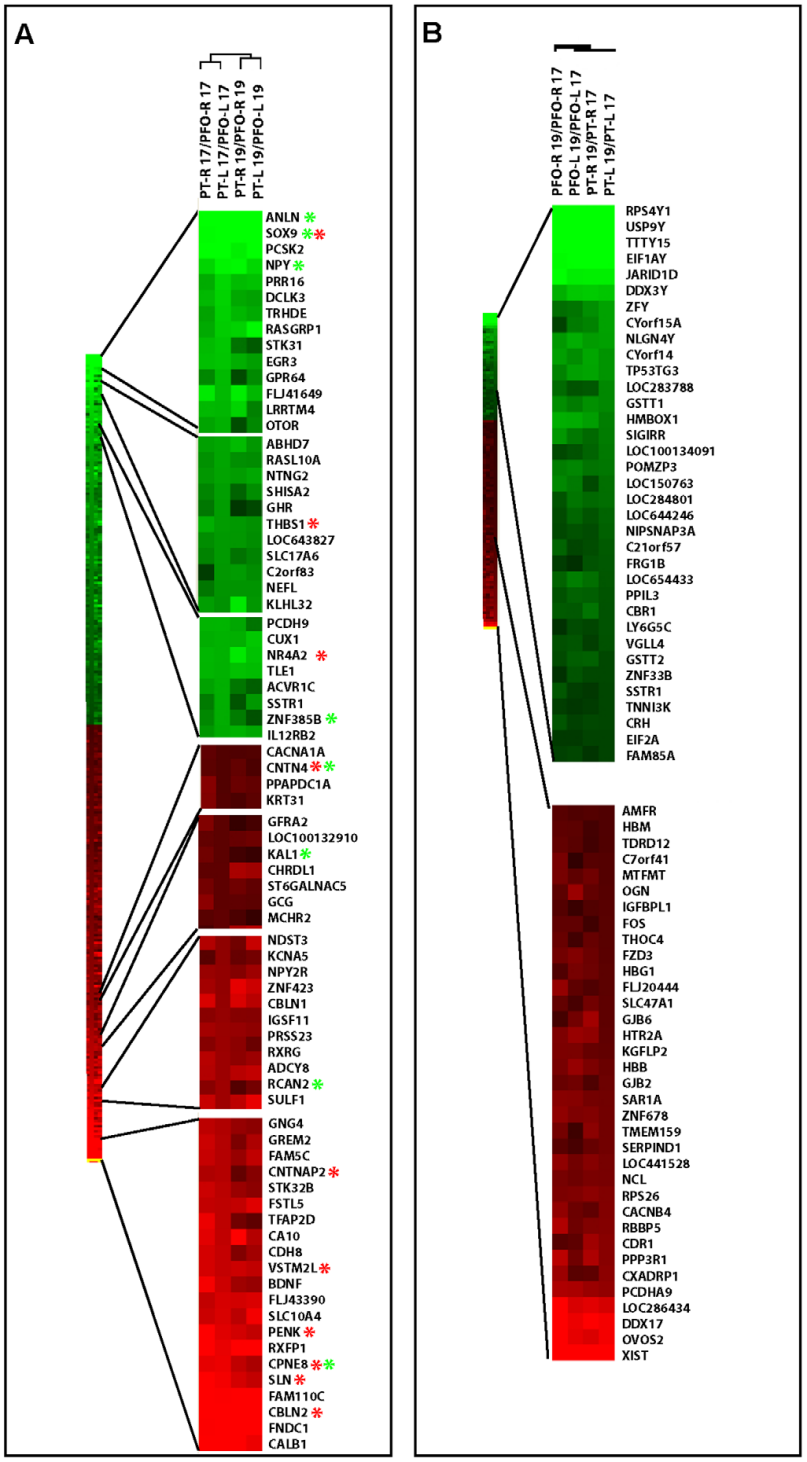
Hierarchical clustering of gene expression. (A, B) Clustering of genes differentially expressed (Fold change >1.5) between PFO VS PT (A) or 17/19GW (B), ordered by differential expression ratios. Red star: genes validated by ISH; green star: genes validated by qRTPCR.

Overall, consistent gene expression variations were thus found associated with inter-areal differences and with gestational age/inter-individual variation, and no variation was found along the lateralization axis (Figure 3 and File S1).

### Specific Gene Ontology patterns for genes differentially expressed between cortical areas

Gene Ontology analysis (http://david.abcc.ncifcrf.gov) was then performed in order to gain biological insights into the functions of the differentially expressed genes. Not surprisingly, most genes differentially expressed in PFO vs. PT areas were found to be associated with brain development, including neuronal differentiation, signal transduction and/or cell/axon guidance (Figure 3 and File S1). This gene set is also enriched for membrane receptors and channels, including adhesion molecules and axon guidance receptors. No significant enrichment for particular gene ontology patterns could be detected when comparing 17 and 19 GW samples (data not shown). This could be partly explained by the inter-individual variation that must also contribute to 17 vs. 19 GW differences, thereby making the gene ontology profile too heterogenous to reveal specific patterns. We therefore next focused our analysis on the genes differentially expressed between the PFO and PT areas, pursuing the hypothesis that these genes are most likely associated with area-specific programs of differentiation at the ages examined, with consistent patterns across individuals.

### Validation of regional expression data

**Figure 4:**
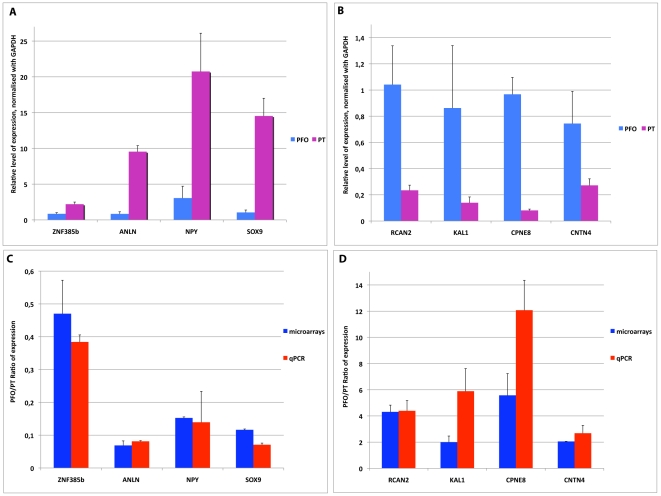
qRTPCR validation. (A, B) qRTPCR relative expression levels of genes upregulated in PT (A) and PFO (B) using the ΔΔCT relative quantification method, normalized to the PFO-17GW-right sample. and to the housekeeping gene GAPDH. (C, D) Comparison of the PFO VS PT expression ratios in microarray and qPCR results. Mean of the PFO 17GW/PT17GW and PFO19GW/PT19GW ratios for the qPCR and microarrays results for genes upregulated in PT (C) and PFO (D).

**Figure 5:**
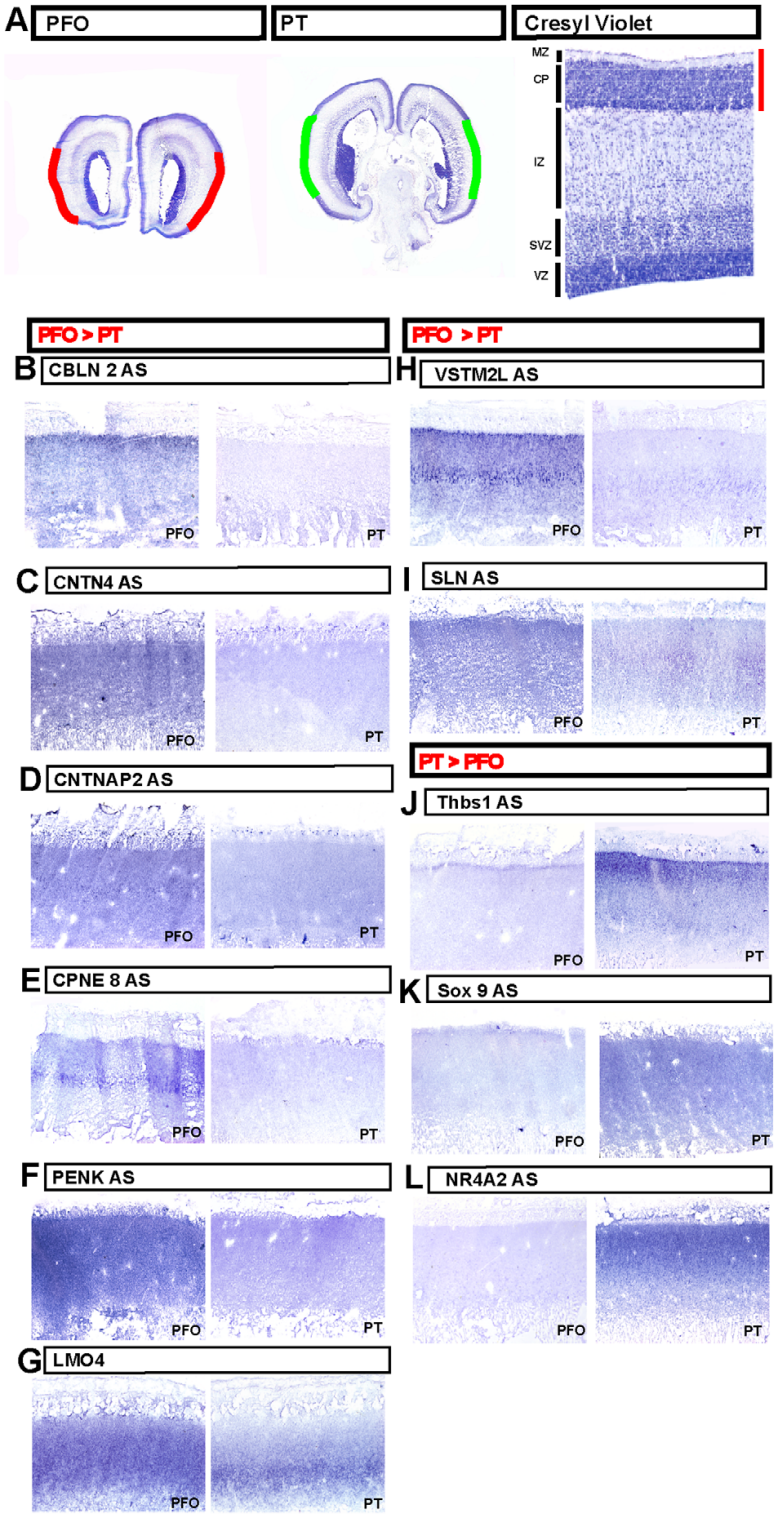
Validation by in situ hybridization. (A) Cresyl violet stained section examples of PFO and PT regions examined by *in situ* hybridization, centred on the cortical plate (right panel). (B–I) Expression pattern of genes displaying higher expression in PFO than PT: *CBLN2* (B), *CNTN4* (C), *CNTNAP2* (D), *CPNE8* (E), *PENK* (F) *LMO4* (G), *VSTM2L* (H) and *SLN* (I) at the level of the microdissected regions within PFO (left panel) and PT (right panel) domains. (J–L) Expression pattern of genes displaying higher expression in PT than PFO: *THBS1* (J), *SOX9* (K), *NR4A2* (L) at the level of the microdissected regions within PFO (left panel) and PT (right panel) domains.

Differential expression between PFO and PT areas was first validated in fetal brain samples by quantitative real-time reverse-transcriptase polymerase chain reaction (qRT-PCR) for eight genes: *RCAN2, KAL1, CPNE8, CNTN4, ZNF385b, ANLN, NPY* and *SOX9*. The expression patterns of all eight genes were confirmed (Figure 4), indicating a robust differential expression among the genes identified through microdissection and microarray analyses. We then performed a more stringent validation using *in situ* hybridization. This analysis was applied to nine genes with patterns of higher expression in the PFO compared to the PT (*CBLN2, CNTN4, CNTNAP2, SLN, CPNE8, PENK, VSTM2L, GNG4, LMO4*) and four genes with lower expression in PFO compared to PT (*THBS1, SOX9, NR4A2, SPON1*). For 11 out of the 13 genes examined, we observed strong differential expression within the cortical domains corresponding to the pattern detected by microarray analysis (Figure 5). The other two genes (*GNG4 and SPON1*) did not display any detectable pattern (data not shown). These results confirm the validity of the microarray data and indicate that our 3D reconstruction-based microdissection method was highly successful at isolating cells from targeted brain areas.

### Spatial patterns of gene expression reveal layer and areal specificity

**Figure 6:**
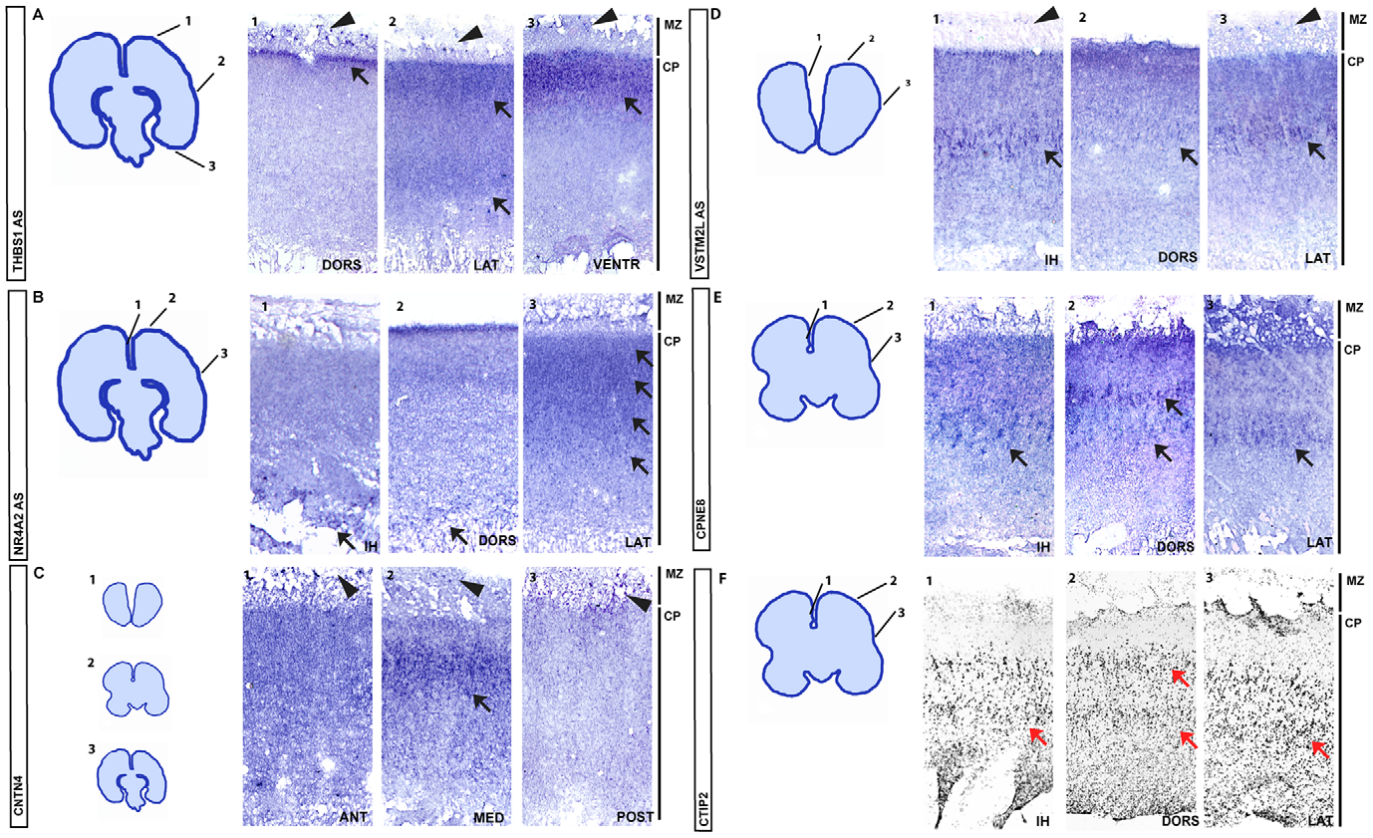
Area and layer-specific patterns of expression of selected genes differentially expressed between PF and PT. (A–E) Among the genes displaying differential expression between PFO and PT domains (*THBS1* (A), *NR4A2* (B), *CNTN4* (C), *VSTM2L* (D), *CPNE8* (E)), additional patterns of expression, corresponding to areal or layer-specificity, can be observed. See text for further description. (F) immunostaining pattern of layer V/VI-specific pattern of CTIP2 provided for comparison. Arrows depict highest levels of expression in the cortical plate for each gene. DORS is dorsal, VENTR is ventral, LAT is lateral, MED is medial, IH is interhemispheric, ANT is anterior, POST is posterior.

More detailed *in situ* hybridization analyses revealed a variety of complex spatial expression patterns both in terms of areal and layer specificity of expression (Figure 6). For example, *THBS1* transcripts were detected in some cells of the marginal zone (MZ) and in the most superficial part of the cortical plate (CP) in the medio-dorsal part of the parietal cortex (Figure 6A). Specifically, *THBS1* was expressed throughout the CP at more lateral levels and in a thick, heavily stained, superficial part of the CP at the most ventral levels. Similarly, *NR4A2* was found to be expressed in the superficial two thirds of the CP in the lateral cortex, while in more dorsal parts the strongest staining for *NR4A2* was localized to the deepest part of the CP (Figure 6B). For *CNTN4* (Figure 6C), the layer specificity of expression varied with anterior-posterior levels: it was found in some cells of the MZ, and diffusely in the CP in the PFO regions, while in PT domains, *CNTN4* was strongest in the superficial part of the CP. Finally, for other genes, such as *VSTM2L* and *CPNE8* (Figure 6D,E), the intensity of the staining depended of the area examined, but the signal was always localized to the same layer, corresponding to presumptive layer V (delineated by CTIP2 expression (Figure 6F)), with the strongest expression localized to the inter-hemispheric part of the CP.

### Evolutionary patterns of putative regulatory sequences of the differentially expressed genes

Together, our microarray, qRTPCR, and *in situ* hybridization data analyses pinpointed a set of genes differentially expressed between PFO and PT cortical domains at mid-gestation. Given the prominence of these cortical areas in human-specific brain anatomy and function, we next examined the evolutionary patterns of predicted regulatory sequences neighboring these genes. Our analysis extends the approach of Johnson et al. (2009), who tested for enrichment of fast-evolving conserved non-coding sequence (CNSs) nearby genes differentially expressed between fetal brain regions or cortical areas. Specifically, we estimated the enrichment of PFO vs. PT genes in several collections of CNSs that were recently identified on the basis of unique patterns of evolutionary acceleration in the human lineage, the so-called HAR or haCNS elements [18], [19]. For comparison, we used the phastCons [26] and phyloP programs [27] to compute parallel sets of elements that are fast-evolving in chimps or mice, rather than humans. Conserved non-coding sequences tend to be nearby genes involved in development [28]. Therefore, we tested for enrichment of HAR and haCNS elements nearby PFO vs. PT genes using the corresponding set of CNSs from which the HARs and haCNS were identified (rather than the set of nearby genes). The set of CNSs allowed us to establish a baseline expected number of HARs or haCNSs nearby PFO vs. PT genes if there were no association between accelerated evolution and differential expression. In other words, all our enrichment tests for accelerated regions are normalized for the excess of CNSs nearby developmental genes.

**Figure 7:**
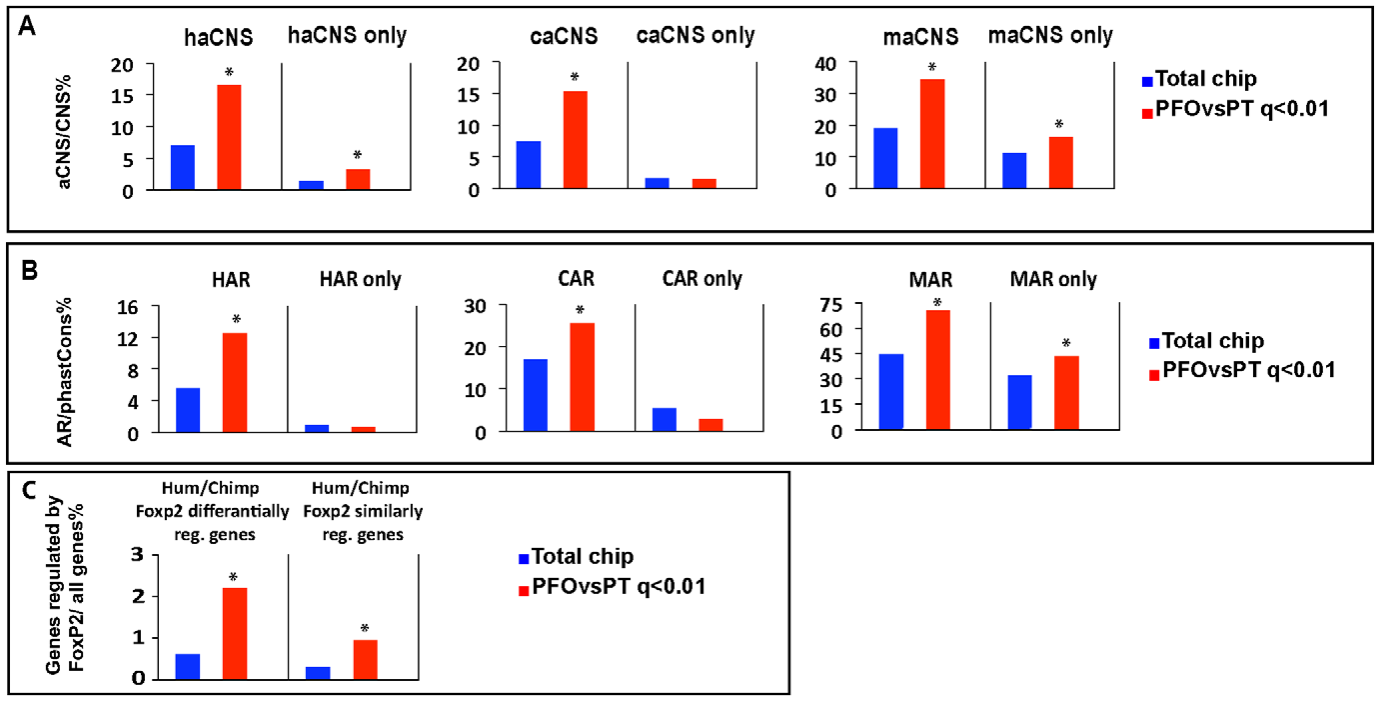
Sequence evolution of putative regulatory sequences nearby differentially expressed genes. (A) Proportion of PFO vs. PT differentially expressed genes found nearby either human (haCNS), chimpanzee (caCNS) or mouse (maCNS) accelerated regions [19], or found exclusively nearby accelerated regions of one species only (haCNS only, caCNS only, maCNS only), normalized to the total number of genes found nearby conserved non-coding sequences (CNS). Significantly enriched values are highlighted by stars. Corresponding p-values: haCNS 2.8E-24, haCNSonly 0.0000424, caCNS 8.43E-17, caCNS only 0.59532206, maCNS 3.18E-29, maCNSonly 0.00000215. Differential expression was defined with the SAM procedure and a q-value threshold of 0.01. (B) Same analysis as in (A) with HAR/CAR/MAR accelerated regions. HAR, CAR and MAR stand for human, chimp and mouse accelerated regions, respectively. Significantly enriched values are highlighted by stars. Corresponding p-values: HAR 3.16E-21, HARonly 0.8746785, CAR 4.36E-15, CARonly 0.999995635, MAR 6.16E-76, MARonly 1.09E-17. (C) Proportion of PFO vs. PT differentially expressed genes among putative targets of Foxp2, either regulated similarly or differentially by human and chimpanzee Foxp2. Significantly enriched values are highlighted by stars. Corresponding p-values are given for each proportion in the right panel.

First, we tested the null hypothesis that there are as many PFO vs. PT genes nearby HARs (or haCNSs), as there are nearby random similar-sized subsets of the complete set of CNSs. We found that predicted regulatory regions with accelerated substitution rates in humans (haCNS or HAR) are strikingly enriched nearby genes differentially expressed between PFO and PT (Figure 7A,B). We then performed the same analysis using regions accelerated in chimpanzees (CAR/caCNS) or mice (MAR/maCNS). Remarkably, the PFO vs. PT gene set was also enriched for these regions. In each species, we found a similar over-representation of predicted fast-evolving CNSs associated with human PFO vs. PT differentially expressed genes. These findings imply that genes differentially expressed between the PFO and PT cortical areas have a propensity to be associated with conserved non-coding sequences showing lineage-specific patterns of evolution in mammals, but that this pattern is not exclusive to the human lineage. Nonetheless, we did find PFO vs. PT differentially expressed genes that are exclusively associated with accelerated non-coding sequences in single lineages, including 27 genes with putative regulatory regions accelerated only in the human lineage (Figure 7, haCNS only). Consistent with the Gene ontology profiles, we found no such enrichment for lineage-specific regulatory evolution among the genes displaying differential expression between 17 and 19 GW samples (data not shown), suggesting that the enrichments we identified among the PFO vs. PT genes reflect particular patterns of evolution of regulatory sequences found nearby genes differentially expressed between cortical areas (File S1).

As a first step to try to relate these findings more directly to transcriptional regulation, we next analyzed the evolutionary divergence at putative transcription factor binding sites (TFBS) located within the HARs nearby genes assayed in our microarray experiments. Specifically, we used the JASPAR database of transcription factor binding motifs [29] to predict binding sites in the human and chimpanzee versions of each HAR sequence. Then, we quantified the overall loss or gain of binding motifs in the human lineage using a novel statistical test for regulatory divergence (Kostka et al., in preparation). This analysis identified 170 genes associated with HARs that display a significant loss or gain of binding sites in human compared with chimpanzee (TFBS divergence) (File S1). Interestingly, TFBS divergence is significantly enriched among PFO vs. PT differentially expressed genes (hypergeometric p = 0.019), but not among genes differentially expressed between 17 and 19 GW (p = 0.39). We noticed that many of the PFO vs. PT genes with TFBS divergence are involved in human cortex development and disease (including *WBSCR17, NLGN1, PDE4IP, MDGA1 and EFNA5*) (File S1). In addition, for several of these genes, we found that the corresponding TFs were also differentially expressed between PFO and PT (File S1), suggesting that they could be part of a rapidly changing pathway where TFs and their targets display differential expression and evolutionary divergence. Overall, our findings suggest that the human-specific substitutions in HARs nearby PFO vs. PT genes may have contributed to regulatory changes in these genes during human evolution.

We were curious whether transcription factors linked to human brain development and evolution differentially regulate genes expressed in the developing cortex. We therefore looked at the distribution of *FOXP2* target genes among the differentially expressed genes. We selected *FOXP2* because of its potential involvement in language-related neural functions and brain evolution, and because its targets have been experimentally investigated in several studies. We focused on two sets of targets: those that are proposed to be regulated similarly by human and chimpanzee forms of *FOXP2* and those that seem to be differentially regulated by either form [24]. We found a striking enrichment of both sets of putative *FOXP2* targets among genes differentially expressed between PFO and PT, but not between 17 and 19 GW (Figure 7C,D and File S1).

Overall, these data suggest that genes expressed differentially in PFO vs. PT areas display significant evolutionary differences in their transcriptional regulation. This regulatory evolution may have been achieved through changes mediated by HAR/haCNS enhancers, as well as by specific transcription factors, such as *FOXP2*, that display human-specific features.

## Discussion

A major challenge in neurobiology is to understand the basic mechanisms underlying the acquisition of species-specific neural traits, and in particular what makes the human species unique in terms of cognitive abilities. Transcriptome comparisons of human and chimpanzee have revealed interesting trends of human-specificity in the postnatal and adult brain [30], [31], [32], [33], [34]. Recent studies have also started to probe the transcriptome of the human fetal cortex, thereby identifying genes differentially expressed between distinct cortical domains or displaying lateralized expression, some of which display human-specific patterns of evolution [14], [15], [16]. Despite this recent progress, our knowledge of the primary features and mechanisms of gene expression in human developing cortex remains scarce, and strong links between gene expression studies and human genome evolution remain to be made.

### Combining neuroembryological reconstruction with whole transcriptome analysis

Here we combined neuroembryological and molecular approaches to unravel parts of the genetic networks potentially linking human brain development and evolution, focusing on areas of the cerebral cortex that underwent significant divergence in recent primate evolution. Our novel three-dimensional microdissection method has provided a highly accurate and reproducible delineation of the cortical areas of interest, which resulted in highly significant results despite the limited number of fetal cases examined. Such an approach may prove to be useful in the future to define and microdissect other regions of the developing brain with great accuracy, providing better reproducibility of such experiments between different samples or even laboratories. In this study, the 3D-reconstructions targeted dissections specifically to post-migratory neurons in the cortical plate. This targeted dissection is a significant advance compared to prior approaches, which involved dissecting the whole thickness of cortical hemispheres, thus producing samples containing an undefined mix of neural progenitors and neurons. Indeed some of the developmental and evolutionary mechanisms underlying the species-specific differentiation of cortical areas are likely linked to selective patterns of expression in postmitotic neurons.

### Distinct evolutionary patterns of transcriptional regulation in the human developing cortex

It has been proposed that changes in gene transcriptional regulation play a critical role in evolution, so that divergence of non-coding regulatory sequences may bear more significance than evolution of coding sequences [35]. We investigated regulatory divergence in the framework of our data sets by analyzing in detail the relationship between genes differentially expressed in the human developing cortex and accelerated non-coding regions of the mammalian genome, which are likely to correspond to transcriptional regulatory elements and display lineage-specific patterns of evolution [18], [19]. This investigation revealed a selective enrichment of such elements in the vicinity of the genes that are differentially expressed between distinct cortical domains (PFO vs. PT). A similar enrichment of human accelerated haCNS elements was previously detected for genes differentially expressed between different cortical regions, distinct from those examined in this study [14]. However, our extended analysis of regions accelerated in non-human mammalian genomes also enabled us to reveal that PFO vs. PT differentially expressed genes are additionally enriched for patterns of regulatory change in the chimpanzee and mouse lineages. Importantly, these enrichments for accelerated non-coding sequences cannot be explained by the more general enrichment of conserved elements nearby developmental genes [28]. Indeed, because we used the set of conserved elements from which the HARs, CARs, and MARs were identified as the baseline in our tests for enrichment, the observed patterns of fast-evolving regulatory elements nearby genes involved in areal patterning reflect a true enrichment above background expectations that is not biased by the larger number of CNSs nearby certain classes of genes [36]. Thus, our findings suggest that the transcriptional control of these genes is more likely to have undergone positive selection in many lineages. Importantly, regulatory changes in these developmental genes have the potential significantly affect important brain features, such as number of areas, size, and connectivity. Furthermore, our data nicely illustrate that evolutionary changes that drive species-specific patterns of gene expression in the developing brain are not a feature unique to the humans; the same sets of genes involved in brain patterning may be particularly prone to changes in transcriptional regulation in many mammal species. This suggests that cortical evolution in different mammalian species may be driven in part by species-specific changes in the regulation of the same genes and pathways, which are potentially important in brain patterning in many species. It would be interesting to test these hypotheses more directly by comparing gene expression patterns of the identified PFO vs. PT genes in the non-human developing cortex, and relating shared and species-specific differentially expressed genes to changes in each species' transcriptional regulatory elements.

Our findings are also consistent with a growing body of evidence that developmental enhancers, while sometimes deeply conserved throughout evolution [37], [38], can be quite dynamic in terms of their genomic position and sequence content [39], [40]. Since lineage-specific changes in enhancer sequences may result in changes in function [22] or not [41], our evolutionary approach was particularly useful for highlighting a unique set of developmental genes with both differential expression between cortical regions and species-specific DNA substitutions predicted to alter transcription factor binding in multiple mammals. These genes may have especially plastic regulatory programs and are therefore exciting candidates for further functional studies to link mammalian brain development and evolution.

As a first test for the relevance of these findings to transcriptional regulation, we used a novel paradigm to analyze the divergence in putative transcription factor binding sites in HARs nearby genes in our microarray study. This analysis enabled to identify 170 genes in the vicinity of accelerated regions that display significantly altered transcription factor binding profiles in the human genome compared to chimpanzee. Interestingly, we found enrichment for PFO vs. PT differentially expressed genes in this set, further suggesting specific evolutionary properties of the transcriptional control of the genes differentially expressed in the presumptive language and associative areas of the human developing cortex.

When looking at those genes differentially expressed between different stages/individuals we did not detect a similar enrichment for divergent regulatory regions. Consistent with this finding, our gene ontology analyses revealed that PFO vs. PT differentially expressed genes mainly correspond to brain development genes, likely involved in the building of area-specific patterns of neuronal identity and connectivity, while genes differentially expressed between 17 and 19 GW show no such ontology pattern. These differences may well be due to the fact that in this study the stage differences are also linked to inter-individual differences. It will be interesting to extend our analyses to more fetal cases at similar gestational ages, in order to determine more accurately the gene ontology and evolutionary patterns of the genes differentially expressed at different stages within the same cortical areas.

### Potential links with human-specific function and diseases

While the PFO vs. PT differential expression identified here may correspond in part to anterior-posterior differences between frontal and parieto-temporal cortex, some of it is also likely to reflect the transcriptional programmes that are specifically active in language areas. Language acquisition is thought to have occurred at some point during late hominid evolution, but the underlying mechanisms remain completely unknown. Several hypotheses have been proposed, including the evolutionary acceleration of genes involved in the control of fine motor control, such as the *FOXP2* gene, which is also mutated in human-specific forms of language production impairment [20], [42]. While we did not find significant differences in *FOXP2* expression between the cortical regions examined, we did find a significant enrichment for *FOXP2* putative targets among genes differentially expressed between the PFO and PT areas. Among these, *CNTNAP2* [24] has also been suggested to be associated with neurodevelopmental disorders affecting language [42]. These findings might reflect differential expression of *FOXP2* at an earlier developmental stage, or involvement of a co-regulator.

Aside from language, several genes were uncovered that could be involved in other important aspects of cortical function and disease. The *NR4A2* transcription factor gene is a particularly striking example as it displays highly complex differential expression in PFO and PT areas and layers (Figures 5,6), and for which higher divergence of binding sites are found in several target genes showing differential expression in the same regions (File S1). *NR4A2*, also known as Nurr1, encodes a member of the steroid-thyroid hormone-retinoid receptor superfamily that is expressed in a complex pattern in the cortex, which may be different in mouse and primates [43], [44], and could be involved in several human brain diseases [45], [46]. Similarly, we identified Thrombospondin-1 (*THBS1*), which binds to *LRP8* (a.k.a. APOER2) and *VLDLR* receptors, also receptors for reelin, and seems to play a role in murine postnatal neuronal migration, as well as in synaptogenesis [47], [48]. As this gene is differentially expressed between different cortical areas in humans and also expressed among pioneer Cajal-Retzius neurons, it constitutes an intriguing candidate gene linking early and late aspects of cortical development.

Our analyses of acceleration and TFBS divergence also highlighted several differentially expressed genes potentially involved in human cortex development and disease, such as *WBSCR17* (candidate gene of the cognitive Williams-Beuren syndrome)[49], *NLGN1* (implicated in synapse formation and autism)[50], *PDE4IP* (implicated in control of brain size or human microcephaly)[51], [52], *MDGA1* and *EFNA5* (guidance factors involved in cortical patterning) [53], [54], [55].

In conclusion, our approach combining neuroembryology and whole genome expression profiling, together with evolutionary analyses of putative regulatory regions, led to the identification of a distinct repertoire of cortical genes displaying selective patterns of expression and evolution. This gene set is a rich source of candidates to elucidate the genetic networks underlying human cortex evolution and the acquisition of higher neural functions.

## Materials and Methods

### Tissue collection and preparation

The study was approved by the three relevant Ethics Committees (Erasme Hospital, Université Libre de Bruxelles, and Belgian National Fund for Scientific Research FRS/FNRS) on research involving human subjects. Written informed consent was given by the parents in each case.

Human fetuses were obtained following medical pregnancy termination. Two fetuses aged 17 and 19 GW were used for the microarray analyses, while additional cases used for validation ranged from 9 to 24 GW. All the cases were examined with standard feto-pathological procedures [56] and none displayed clinical or neuropathological evidence of brain malformation. As soon as possible after expulsion (less than 6 hours), the brain was removed using the standard fetal autopsy procedure (Valdes-Dapena, 1983), embedded as a whole in OCT compound (Sakura), and snap-frozen in a 2-methylbutane on dry ice bath. Post-mortem delay before freezing was 2 h for the 19 GW fetus and 6 h for the 17GW fetus.

### 3D reconstruction and dissection of specific cortical areas

All specimens were cut in the coronal plane on a customized Leica CM3000 microtome. A digital picture was taken every ten slides (each 25 µm thick). 3D reconstruction of each brain was performed using customized procedures ([57] and File S1). Briefly, pictures were aligned manually using Adobe Photoshop to generate the image stack needed to perform the 3D reconstruction of the brain. 3D-doctor (Able Software Corp.®) was used for the rendering of the 3D model, which was then used to select defined cortical areas PFO and PT, containing presumptive Broca and Wernicke areas. These were first selected on the 17 GW left hemisphere, then transferred to the right hemisphere and to corresponding regions in the 19 GW brain. To this aim we used the 3D doctor registration function to transform the shape of the 17GW model into the shape and size of the 19GW model, creating a 17GW^p19^ model, which then could be faithfully matched to the actual 19GW model. Once all areas boundaries were drawn on the 19GW model, the 17GW^p19^ model was inserted inside the 19GW model to check the area match. The 3D model of each case was then used to determine the sections and subregions to be dissected, in relation with adjacent cresyl violet-stained sections, which enabled to generate precise templates of dissection of the sections of interest, focusing on focusing on the cortical plate of the PFO and PT domains (cf Figure 1). The sections were then dissected manually following these templates, before RNA extraction.

### Transcriptome analyses

RNA was extracted from each sample (PFO-17GW-left, PT-17GW-left, PFO-19GW-left, PT-19GW-left, PFO-17GW-right, PT-17GW-right, PFO-19GW-right, PT-19GW-right) using RNeasy kit (Qiagen), and the corresponding cDNAs were prepared and hybridized according to manufacturer's instructions (Affymetrix HGU133+v2.0). All statistical analyses were performed with the R language for statistical computing version 2.9.0 (R Core Development [58] and Bioconductor 2.4 [59] with all functions run using default parameters unless specified otherwise. Data are available from the GEO database (http://www.ncbi.nlm.nih.gov/geo) under accession number GSE21858.

#### Pre-processing

Affymetrix HGU133+v2.0 chips were normalized with the Robust Multi-array Analysis (RMA) program [60] and annotated with the HGU133+v2 Bioconductor annotation packages. Probe sets mapping to a same gene symbol were averaged.

#### Detection of regulated genes

Regulated genes were searched with the Significance Analysis of Microarray version 1.26, a nonparametric procedure that handles multiple testing [25]. The same unpaired two-class set up, 4 vs. 4 arrays, was used in the comparison of 17 vs. 19 weeks, left vs. right, and PFO vs. PT areas. As an alternative analysis, we defined differentially expressed genes as those with a 1.5-fold change in expression, i.e., expression values were averaged across the lateral axis in each class, and then the ratio of the averages of the two classes was calculated.

#### Dimension reduction

Principal components analysis was computed with R's *prcomp* function using all the genes present on the microarrays. Hierarchical clustering was calculated with Cluster [61].

### Evolutionary computational analyses

Each conserved non-coding sequence (CNS) plus the subsets of human- chimp- and mouse-specific accelerated regions were obtained from [19]. For comparison, we computed a larger set of conserved elements using multiple sequence alignments of all currently available mammalian genomes from the UCSC genome browser database using the *phastCons* program (Siepel et al., 2005). Then, we identified subsets of human-, chimp-, and mouse-accelerated elements using the program *phyloP* [27]. All conserved elements from both sources were mapped to the nearest gene with Galaxy [62] using appropriate UCSC human genome build, hg17 for CNS [19] and hg18 for phastCons elements. Nearest genes were then mapped to the Affymetrix microarray data on the basis of gene symbols. Enrichment analyses were performed using the hypergeometric test, with the entire CNS or phastCons list as a reference set.

Transcription factor binding sites were predicted in the human and chimp versions of each HAR sequence using position-specific weight matrices from the JASPAR database (release 12 Oct 2009) [29]. We assessed the statistical significance of binding site losses and gains by combining evidence across transcription factors, using a model for two correlated binomial distributions (Kostka et al., in prep.). Enrichment analyses were performed as above.

### In situ hybridization

In situ hybridization using digoxigenin-labeled RNA probes was performed as described previously using PCR amplified or plasmid templates [63], [64]. The PFO and PT alternate sections were always processed together in order to allow comparison of the obtained staining. Sense probe was used as a negative control for each probe and revealed no specific staining (data not shown).

### Quantitative RT-PCR

qPCR primers were designed using *primer3* (http://frodo.wi.mit.edu/primer3/). cDNA was synthesized from 250 ng total RNA from the 8 cortex samples using random hexamers (Qiagen) and SuperScript™ II Reverse Transcriptase (Invitrogen). qPCR reactions (10 ng cDNA) were performed using Brilliant II FAST SYBR Green QPCR Master Mix and ROX (Stratagene). Amplification of the gene of interest and the housekeeping control genes -GAPDH and TTC1 was done in duplicate from each sample and a no template control. Quantification was done using the ΔΔCT relative quantification method. The sample PFO-17GW-right was used for calibration.

## Supporting Information

File S1 (PDF)
