# Supplementary material for: Genes Expressed in Specific Areas of the Human Fetal Cerebral Cortex Display Distinct Patterns of Evolution
Source: PLoS One. 2011 Mar 18;6(3):e17753. doi: 10.1371/journal.pone.0017753 (PMC3060818; doi:10.1371/journal.pone.0017753)
Supplement: File S1 — (PDF) [file pone.0017753.s001.pdf]

Supporting Information

Legends to Supplementary Figures and Tables

Supplementary Figure 1

Hierarchical clustering of gene expression.

Genes differentially expressed between PFO vs. PT or 17 vs. 19GW (fold-change threshold was set to 2 for the sake of display clarity), hierarchical clustering by absolute levels of expression through the eight different areas/stages, PFO vs. PT and 17 vs. 19GW cluster together. (Left panel) A and B are two clusters of gene expression patterns across all 8 samples. Clusters A and B are shown enlarged in the middle and right panels.

Supplementary Tables

Supplementary Table 1:

Sequence evolution of putative regulatory sequences nearby differentially expressed genes (aCNS).

Genes differentially expressed between PFO vs. PT or 17GW vs. 19GW among the CNS, haCNS, caCNS, maCNS (Prabhakar et al.2006), the proportion of these genes among CNS are represented in Figure 7A.

PFOvsPT.q, PFOvsPT.p, 17vs19.q, 17vs19.dp: dp and q-values (i.e. multiple testing-adjusted confidence measures) for the considered analysis. d: SAM's moderated t-statistics.

PFOvsPT.FC, 17vs19.FC: Fold change values.

NA: “Not available”. For genes lists: the gene is not in the considered sub-list. For q-values: q-value could not be estimated.

Supplementary Table 2:

Sequence evolution of putative regulatory sequences nearby differentially expressed genes (ARs).

Genes differentially expressed between PFO vs. PT or 17GW vs. 19GW among the CNS, HAR, CAR, MAR, the proportion of these genes among CNS are represented in Figure 7B. (See legend Supplementary Table 1).

Supplementary Table 3:

TFBS divergence analaysis

First table:

List of genes next to an HAR containing at least one TFBS with turnover at FDR<0.05; C: TFs for these TFBS with FDR<0.05 and their expression values; q-value and fold-change (PFO vs. PT and 17GW vs. 19 GW) for these genes. In green genes with significant (q<0.01) PFO vs. PT differential expression, in red TFs related to TFBS with PFO vs. PT (q<0.01)

Second table:

Same list, but only with genes or TF with PFO vs. PT q<0.01, Red TF related to TFBS which are themselves differentially expressed in PT vs. PFO at q<0.01; location of the considered gene and the HAR in which the TFBS are predicted. Some genes appear more than one time if the TFBS are in two different HARs.

Supplementary Table 4:

Genes differentially expressed in PFO vs. PT (q<0,01), that are nearby a HAR containing at least one transcription factor (TF) with a significant loss or gain of predicted binding sites in the human lineage (FDR<0.05). The corresponding TFs are in the 2nd columm, TFs differentially expressed in PFO vs PT are in red.

Supplementary Table 5:

FoxP2 targets among differentially expressed genes.

Genes differentially expressed (between PFO vs. PT) among the genes differentially or similarly regulated by human and chimpanzee FOXP2 (represented in Figure 7C).

Supplementary Methods

3D reconstruction

Assembling the picture stack. The pictures of the frozen block face were manually aligned one at a time with Adobe Photoshop CS (Adobe Systems Inc.®). The picture of the first section was used as background onto which a picture of the adjacent section was superimposed, as a second layer. This "layer 2" was made semi-transparent and aligned using the transform-rotate and transform-scale commands to correct misalignments due to camera instability or block removal for the night. This allowed a reliable overlaying of the consecutive pictures. When the alignment was optimal, "layer 2" was made opaque again and the same procedure was applied to the next picture, and so on for the whole set of pictures. Each aligned layer was saved afterwards in an individual jpeg file numbered according to its position along the z-axis. This series of jpeg images corresponds to the image stack needed to perform the 3D reconstruction of the brain.

3D Model. 3D-doctor (Able Software Corp.®) was used for the rendering of the 3D model. The stack of images was loaded in the program and the true voxel size and the units (µm) were entered in the calibration menu. The image true pixel size in µm (x and y voxel value), was measured in Photoshop® by taking the picture of a scale bar graduated at intervals of 0.1 mm. The z value of the voxel corresponded to the slide thickness set on the microtome when cutting the sample.

The first step of the model construction was to outline the outer limits of the brain using free hand drawing to get its general shape. As 3D-doctor allowed adding slices to an existing stack, it was possible to start drawing from a restricted number of pictures, add the next slices progressively and check while drawing the global quality of the alignment for any drifting occurring in the stack due to human alignment errors. Transition from one section to the next was smooth. Only a very limited number of sections had to be realigned before the stack was judged optimal.

Once the brain boundaries were traced on all images, these boundaries were smoothened using the "boundary process-smooth boundary" command, reducing the distance between nodes to 10, to reduce computer processor time when generating the surface model. The available surface-rendering modes were tested; "complex rendering" gave the best results and was therefore used in all subsequent steps using the most detailed parameters (x=1, y=1, z=1). The generated surface model was smoothened three times using the "tools-smooth surface" command to reduce the "steps" generated by this mode of reconstruction and to give a more natural-looking appearance to the brain structures.

Selection of the target areas on the 17GW model. We wanted to compare the gene expression pattern of two cortical areas – Broca and Wernicke areas – which in the adult brain would be involved in speech to determine if some cortical asymmetry was already present in our foetal brains. Since most major cortical grooves start to develop during the third trimester, we decided to draw on our 17GW model a wide frontal area that in adult brain would encompass without doubts PFO and PT, encompassing Broca and Wernicke areas. These areas were drawn first on the left side of the model.

Once the choice of limits of these areas was firm, we reported them exactly on the right side of the 17GW brain.

In order to project exactly the same areas onto the right hemisphere, we generated two half models of the 17GW brain using the edit-boundary process-split object command. The left side of the brain including its chosen target areas was then flipped left to right in the model viewer using the tools-mirror model command and moved to merge as exactly as possible with the right side model. The target areas were the drawn on the right side model to match as closely as possible their left counterparts.

Transferring the target areas to the 19GW model. We first tried to match the 17GW model inside the 19GW model to transfer the target area for one model to the second, as we had done to transfer the left areas to the right hemisphere in the 17GW model. We observed that the size and shape of the hemispheres was too different between these models to allow unambiguous areas transfer between them on such a visual-only basis. We therefore used the 3D doctor registration function to transform the shape of our 17GW model into the shape and size of our 19GW model, creating a $17GW^{p19}$ model, which then could truly be matched to the actual 19GW model.

The registration command applies only to the pictures of the stack of origin. It will stretch the voxels according to the demand imposed by the new reference points, modifies the angle of slicing and reslices to match the angle and number of slices of the stack of destination. This command does not apply to the hand drawn boundaries. Yet we needed our chosen areas to go though the same transformation as the whole brain. To solve this issue, we exported for each image of our origin stack the pictures of the window showing the frozen block image and its hand-drawn boundaries, creating a new stack, identical to the first one but with the hand drawn boundaries

integrated into the frozen block image. In Photoshop® the selected cortical area boundaries were then filled with colour so they would be easier to recognize after registration reslicing. Registration requires the user to define at least 4 “identical” reference points on the two models. We tried different reference points and various numbers of them. The best result was obtained using 6 reference points placed respectively at the tip of the frontal, temporal and occipital lobes of the two hemispheres.

Once registration was done, the boundaries of the brain and of the four selected cortical areas were drawn on the new $17GW^{p19}$ stack. These boundaries were then saved and imported to their corresponding frozen block picture in the 19WG stack. Since transformation from the 17GW model to the 19GW model was not a perfect point to point morphing, some area boundaries had to be slightly rotated or shifted to follow more closely the actual curves of the 19GW brain. Once all areas boundaries were drawn on the 19GW model, the $17GW^{p19}$ model was inserted inside the 19GW model to check the area match.

Dissection of the 3D-selected cortical areas on the frozen sections

Pictures of the window showing the frozen block image corresponding to the areas to be actually dissected and its hand drawn boundaries were exported to Photoshop. The area boundaries were filled with bright printer friendly colour for easier visual identification. The images were flipped horizontally so they could be placed under their respective frozen section. The left side of the brain was marked on each picture to avoid left-right confusion during dissection. The images were printed in colour to the exact size of their frozen section counterpart. This was checked with the cresyl violet staining of adjacent sections. The colour pictures were put inside a small

RNAse-out treated plastic cover and placed under their respective frozen section for dissection. The slides were dissected on a bed of carboice to avoid RNA degradation. Dissection was done without magnification, with a precision blade, inside the chamber of the cryotome. The dissected material was recovered in seperate RNAse-free eppendorfs placed on carboice. RNA extraction was performed immediately after dissection of all sections was completed, using the RNeasy extraction kit.

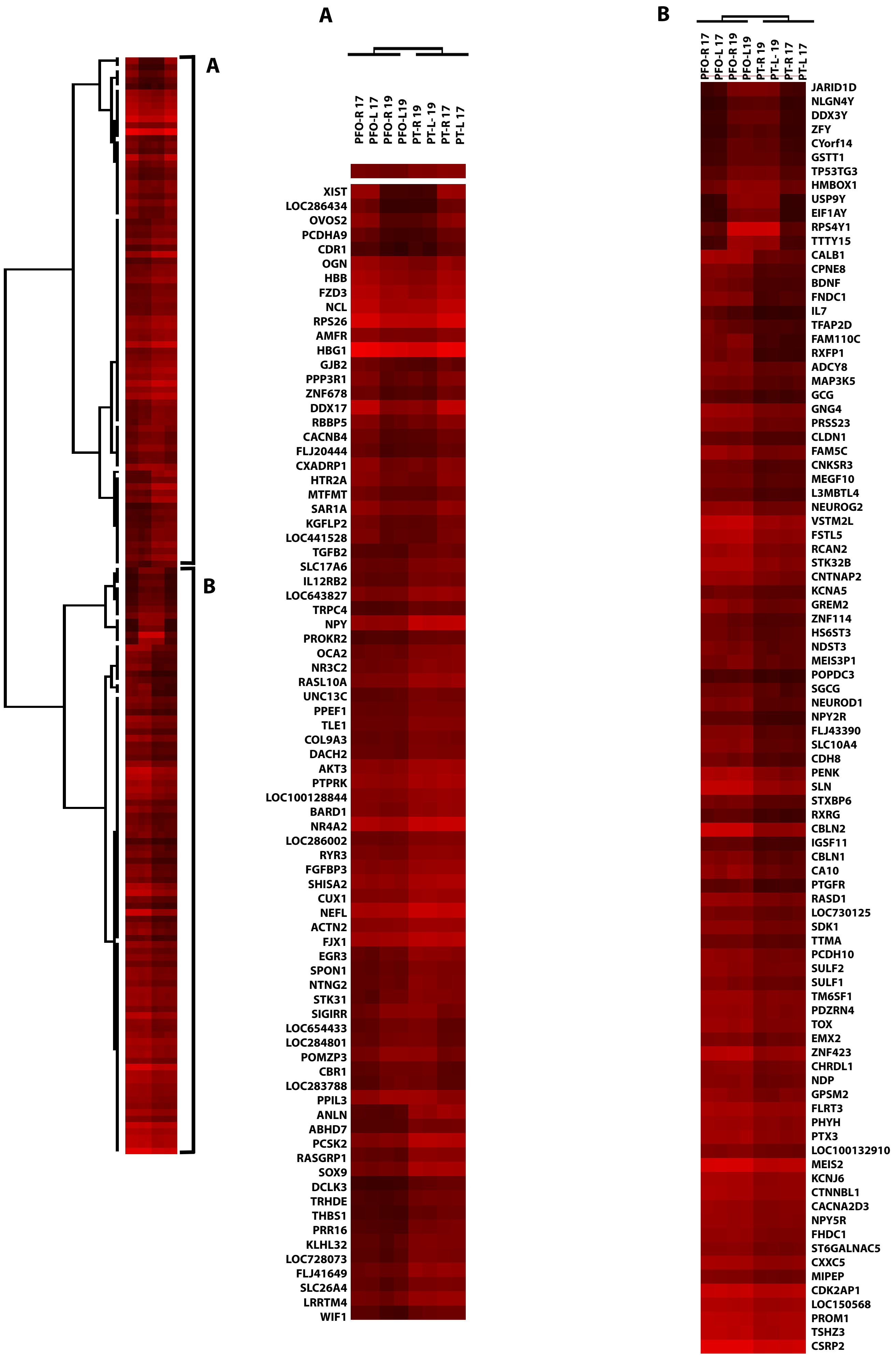

Lambert et al. suppl Fig 1

Supplementary table 1

haCNS PFOvsPT q<0.01

SYMBOL	PFOvsPT.d	PFOvsPT.FC	PFOvsPT.q	haCNS	caCNS	maCNS
CA10	-6.12	5.712212733	0	CA10	CA10	CA10
CDH8	-6.06468643	4.185181997	0	CDH8	CDH8	CDH8
TFAP2D	-3.50782293	3.658017759	0	TFAP2D	NA	TFAP2D
CNTNAP2	-5.12221366	3.340737607	0	CNTNAP2	CNTNAP2	CNTNAP2
STXBP6	-7.55969727	3.299394519	0	STXBP6	NA	STXBP6
MEIS2	-8.05642148	3.203435352	0	MEIS2	NA	MEIS2
TOX	-6.24702549	3.160502065	0	TOX	TOX	TOX
IGSF11	-7.55618738	3.097316213	0	IGSF11	IGSF11	IGSF11
PCDH10	-5.84345127	2.908355915	0	PCDH10	PCDH10	PCDH10
CHRDL1	-4.52809552	2.883850704	0	CHRDL1	NA	CHRDL1
L3MBTL4	-7.31353178	2.854053804	0	L3MBTL4	NA	NA
PDZRN4	-5.44764863	2.63707924	0	PDZRN4	PDZRN4	PDZRN4
BMP2	-4.21371593	2.614685809	0	BMP2	NA	BMP2
CTNNBL1	-5.1701204	2.605088071	0	CTNNBL1	NA	NA
HS6ST3	-3.6617101	2.521555404	0	HS6ST3	HS6ST3	HS6ST3
PART1	-4.64566281	2.46597251	0	PART1	PART1	PART1
EMX2	-3.67376004	2.379665984	0	EMX2	NA	EMX2
PPAPDC1A	-5.09062142	2.375287236	0	PPAPDC1A	NA	NA
CACNA2D3	-5.01836394	2.373116161	0	CACNA2D3	CACNA2D3	CACNA2D3
CSRP2	-5.78367684	2.305385047	0	CSRP2	NA	NA
ANKRD32	-5.24414946	2.29550366	0	ANKRD32	NA	ANKRD32
PTER	-3.93798799	2.213952941	0	PTER	NA	PTER
LMO4	-5.43330418	2.097623295	0	LMO4	LMO4	LMO4
CNTN4	-4.29563654	2.053456928	0	CNTN4	CNTN4	CNTN4
RPRM	-3.03115814	2.032294178	0	RPRM	NA	NA
MARCH3	-5.56101305	1.984234907	0	MARCH3	MARCH3	NA
DERA	-3.2478245	1.903953655	0	DERA	DERA	DERA
ASTN2	-3.537837	1.845172203	0	ASTN2	ASTN2	ASTN2
GBE1	-3.79150602	1.82357854	0	GBE1	GBE1	GBE1
CDCA7L	-3.20491701	1.821544626	0	CDCA7L	CDCA7L	NA
HS3ST5	-2.68310547	1.772104321	0	HS3ST5	NA	HS3ST5
ADAMTS16	-2.36220106	1.760054514	0	ADAMTS16	NA	ADAMTS16
EGLN3	-2.99677255	1.721010124	0	EGLN3	NA	NA
PTPRT	-2.74804604	1.717870851	0	PTPRT	PTPRT	PTPRT
ACCN1	-2.63076477	1.679795978	0	ACCN1	ACCN1	ACCN1
MEGF11	-2.95386633	1.677663997	0	MEGF11	NA	NA
GLIS3	-2.10301732	1.664953031	0.1132952	GLIS3	GLIS3	GLIS3
POU6F2	-2.68054389	1.636633705	0	POU6F2	NA	POU6F2
LYPD1	-2.40434003	1.635473385	0	LYPD1	NA	NA
CNTN6	-2.8124791	1.62508137	0	CNTN6	CNTN6	CNTN6
LMO3	-2.44233444	1.621965	0	LMO3	LMO3	LMO3
OLFM3	-2.34271783	1.609927878	0	OLFM3	OLFM3	NA
POU3F2	-2.94748566	1.608230126	0	POU3F2	POU3F2	POU3F2
NFIA	-2.02490921	1.547560662	0.1132952	NFIA	NFIA	NFIA
EPHA4	-2.32887211	1.521442732	0	EPHA4	EPHA4	EPHA4
PCDH18	-2.4106968	1.507983221	0	PCDH18	PCDH18	PCDH18
ELAVL2	-2.00023103	1.499451152	0.1132952	ELAVL2	ELAVL2	ELAVL2
POU3F3	-2.37286138	1.478080999	0	POU3F3	NA	POU3F3

LRRTM1	-1.74257338	1.476807726	0.5692172	LRRTM1	NA	LRRTM1
PARP8	-1.89900276	1.453057443	0.1920624	PARP8	NA	PARP8
LDB2	-2.89311943	1.4399603	0	LDB2	NA	LDB2
HS3ST1	-2.20065986	1.422808221	0	HS3ST1	HS3ST1	HS3ST1
SUPT3H	-1.88285687	1.410333462	0.1920624	SUPT3H	NA	SUPT3H
FAM19A5	-2.25379138	1.364349186	0	FAM19A5	NA	NA
SLITRK1	-2.3569568	1.363953962	0	SLITRK1	NA	SLITRK1
ROBO1	-2.1733223	1.360443688	0	ROBO1	ROBO1	ROBO1
DUSP6	-1.66485337	1.342443608	0.5692172	DUSP6	DUSP6	DUSP6
TNFRSF21	-2.27712882	1.337971486	0	TNFRSF21	NA	TNFRSF21
GRIK2	-2.10003409	1.327840632	0.1132952	GRIK2	NA	GRIK2
AADAT	-2.50286153	1.324581374	0	AADAT	NA	AADAT
NMNAT3	-1.75681692	1.320962511	0.5692172	NMNAT3	NA	NA
ZBTB20	-2.21439732	1.320427888	0	ZBTB20	ZBTB20	ZBTB20
DIAPH3	-1.69113303	1.316907999	0.5692172	DIAPH3	DIAPH3	DIAPH3
LPHN3	-1.93568195	1.287259152	0.1920624	LPHN3	LPHN3	LPHN3
TBR1	-1.78527877	1.281547876	0.5692172	TBR1	NA	TBR1
PITPNC1	-1.92418927	1.28031768	0.1920624	PITPNC1	NA	NA
CDH22	-1.64073284	1.269176176	0.8841996	CDH22	NA	NA
CDH11	-1.68075954	1.267706284	0.5692172	CDH11	NA	CDH11
XKR4	-1.6124775	1.258232866	0.8841996	XKR4	NA	NA
DCC	-1.54680033	1.256419694	0.8841996	DCC	DCC	DCC
ID4	-1.71418298	1.249410143	0.5692172	ID4	NA	ID4
SYT9	-1.71749433	1.248318572	0.5692172	SYT9	NA	NA
CSMD3	-1.53036727	1.234721195	0.8841996	CSMD3	NA	CSMD3
NRXN3	-1.67797848	1.232510319	0.5692172	NRXN3	NA	NRXN3
CSMD1	-1.6330538	1.202959298	0.8841996	CSMD1	CSMD1	CSMD1
PTPRD	1.601348957	0.823782065	0.8841996	PTPRD	PTPRD	PTPRD
RIMS4	1.571240855	0.806598496	0.8841996	RIMS4	NA	NA
EFNA5	1.623838337	0.805562936	0.8841996	EFNA5	EFNA5	EFNA5
XYLT1	1.922205135	0.80021924	0.1920624	XYLT1	NA	XYLT1
CDC73	1.56862806	0.79791535	0.8841996	CDC73	NA	NA
CLYBL	1.540808211	0.789655223	0.8841996	CLYBL	NA	CLYBL
NR2F2	1.681058537	0.787077506	0.5692172	NR2F2	NR2F2	NR2F2
LRRC4C	1.772317163	0.783808549	0.5692172	LRRC4C	LRRC4C	LRRC4C
MITF	1.626351977	0.782694496	0.8841996	MITF	NA	MITF
ZNF365	1.608012079	0.782204854	0.8841996	ZNF365	ZNF365	ZNF365
HNMT	1.641286706	0.779961652	0.8841996	HNMT	NA	HNMT
CNTNAP5	1.867266525	0.778953903	0.1920624	CNTNAP5	NA	CNTNAP5
FANCL	1.958283069	0.776417081	0.1920624	FANCL	FANCL	FANCL
SMAD7	1.582277207	0.772544417	0.8841996	SMAD7	NA	NA
TCF4	1.72942339	0.770627215	0.5692172	TCF4	TCF4	TCF4
C2orf3	1.841798128	0.755857562	0.1920624	C2orf3	NA	NA
GRIA3	1.84845098	0.749806617	0.1920624	GRIA3	GRIA3	GRIA3
ACVR1	1.901681292	0.74909317	0.1920624	ACVR1	NA	ACVR1
LMO1	1.553677926	0.745715017	0.8841996	LMO1	NA	NA
CXCR4	2.472439403	0.739130617	0	CXCR4	NA	CXCR4
PTPRG	1.702970376	0.737949782	0.5692172	PTPRG	PTPRG	PTPRG
SNX16	2.022273678	0.735425143	0.1132952	SNX16	SNX16	SNX16
LRRC7	2.138431829	0.730778933	0.1132952	LRRC7	NA	LRRC7
ZNF618	2.4840875	0.730109049	0	ZNF618	NA	NA
CTNNA2	2.349801056	0.726774235	0	CTNNA2	CTNNA2	CTNNA2
EDIL3	2.471871838	0.714879738	0	EDIL3	EDIL3	EDIL3
PBX3	2.630409665	0.710301985	0	PBX3	NA	PBX3
CDH13	1.576661194	0.710124456	0.8841996	CDH13	CDH13	CDH13
NLGN1	1.972999378	0.70393173	0.1920624	NLGN1	NLGN1	NLGN1
KIAA1797	2.297017196	0.701044018	0	KIAA1797	NA	KIAA1797

PPARGC1A	2.525981736	0.696051571	0	PPARGC1A	PPARGC1A	PPARGC1A
DYRK2	2.330251518	0.686718348	0	DYRK2	NA	NA
GRIK1	2.095029121	0.683951491	0.1132952	GRIK1	GRIK1	GRIK1
HAS2	2.05747991	0.683146203	0.1132952	HAS2	NA	NA
DOK5	3.468938151	0.682774508	0	DOK5	DOK5	DOK5
EFNB2	2.068676182	0.67820706	0.1132952	EFNB2	EFNB2	EFNB2
TGFBR1	2.419917641	0.652633398	0	TGFBR1	NA	NA
EPHA7	2.325415831	0.643391453	0	EPHA7	EPHA7	EPHA7
ITPR1	3.650092417	0.639788798	0	ITPR1	NA	ITPR1
DPP10	2.354621865	0.6215833	0	DPP10	DPP10	DPP10
LOC283392	3.210919415	0.587372463	0	LOC283392	NA	NA
MCTP1	3.610437125	0.580458414	0	MCTP1	NA	MCTP1
BCL11A	2.887541535	0.577424878	0	BCL11A	BCL11A	BCL11A
FST	2.456987371	0.573029999	0	FST	FST	FST
SNTG1	4.143762175	0.571025733	0	SNTG1	NA	NA
OPCML	3.730784512	0.528980935	0	OPCML	OPCML	OPCML
PCDH7	4.99435407	0.528261246	0	PCDH7	PCDH7	PCDH7
CDH9	3.347253803	0.502386394	0	CDH9	CDH9	CDH9
NR2F1	4.533384717	0.490489804	0	NR2F1	NR2F1	NR2F1
FAM19A1	4.348552767	0.488005419	0	FAM19A1	FAM19A1	FAM19A1
NCAM2	3.86902789	0.486966988	0	NCAM2	NCAM2	NA
SORCS1	4.398843879	0.484518816	0	SORCS1	NA	SORCS1
LPHN2	4.227025654	0.470334365	0	LPHN2	LPHN2	LPHN2
SORCS3	3.775183207	0.469115015	0	SORCS3	SORCS3	SORCS3
ROBO2	5.389565456	0.464744626	0	ROBO2	ROBO2	ROBO2
NFE2L3	3.290581592	0.457712017	0	NFE2L3	NA	NA
PTPRK	4.763210281	0.397753704	0	PTPRK	NA	PTPRK
FJX1	5.572196893	0.396179661	0	FJX1	NA	NA
BARD1	3.883937603	0.394424527	0	BARD1	NA	BARD1
PCDH9	5.611957099	0.383387329	0	PCDH9	PCDH9	PCDH9
NR3C2	6.294811103	0.354365206	0	NR3C2	NA	NR3C2
LRRTM4	5.923967514	0.235613006	0	LRRTM4	LRRTM4	LRRTM4
TRHDE	7.463230607	0.22727706	0	TRHDE	TRHDE	TRHDE
SOX9	12.91514	0.116417689	0	SOX9	SOX9	SOX9

ha-caCNS PFOvsPT q<0.01

SYMBOL	PFOvsPT.d	PFOvsPT.FC	PFOvsPT.q	haCNS	caCNS	maCNS
TFAP2D	-3.50782293	3.658017759	0	TFAP2D	NA	TFAP2D
STXBP6	-7.55969727	3.299394519	0	STXBP6	NA	STXBP6
MEIS2	-8.05642148	3.203435352	0	MEIS2	NA	MEIS2
CHRDL1	-4.52809552	2.883850704	0	CHRDL1	NA	CHRDL1
L3MBTL4	-7.31353178	2.854053804	0	L3MBTL4	NA	NA
BMP2	-4.21371593	2.614685809	0	BMP2	NA	BMP2
CTNNBL1	-5.1701204	2.605088071	0	CTNNBL1	NA	NA
EMX2	-3.67376004	2.379665984	0	EMX2	NA	EMX2
PPAPDC1A	-5.09062142	2.375287236	0	PPAPDC1A	NA	NA
CSRP2	-5.78367684	2.305385047	0	CSRP2	NA	NA
ANKRD32	-5.24414946	2.29550366	0	ANKRD32	NA	ANKRD32
PTER	-3.93798799	2.213952941	0	PTER	NA	PTER
RPRM	-3.03115814	2.032294178	0	RPRM	NA	NA
HS3ST5	-2.68310547	1.772104321	0	HS3ST5	NA	HS3ST5
ADAMTS16	-2.36220106	1.760054514	0	ADAMTS16	NA	ADAMTS16
EGLN3	-2.99677255	1.721010124	0	EGLN3	NA	NA
MEGF11	-2.95386633	1.677663997	0	MEGF11	NA	NA
POU6F2	-2.68054389	1.636633705	0	POU6F2	NA	POU6F2

LYPD1	-2.40434003	1.635473385	0	LYPD1	NA	NA
POU3F3	-2.37286138	1.478080999	0	POU3F3	NA	POU3F3
LRRTM1	-1.74257338	1.476807726	0.5692172	LRRTM1	NA	LRRTM1
PARP8	-1.89900276	1.453057443	0.1920624	PARP8	NA	PARP8
LDB2	-2.89311943	1.4399603	0	LDB2	NA	LDB2
SUPT3H	-1.88285687	1.410333462	0.1920624	SUPT3H	NA	SUPT3H
FAM19A5	-2.25379138	1.364349186	0	FAM19A5	NA	NA
SLITRK1	-2.3569568	1.363953962	0	SLITRK1	NA	SLITRK1
TNFRSF21	-2.27712882	1.337971486	0	TNFRSF21	NA	TNFRSF21
GRIK2	-2.10003409	1.327840632	0.1132952	GRIK2	NA	GRIK2
AADAT	-2.50286153	1.324581374	0	AADAT	NA	AADAT
NMNAT3	-1.75681692	1.320962511	0.5692172	NMNAT3	NA	NA
TBR1	-1.78527877	1.281547876	0.5692172	TBR1	NA	TBR1
PITPNC1	-1.92418927	1.28031768	0.1920624	PITPNC1	NA	NA
CDH22	-1.64073284	1.269176176	0.8841996	CDH22	NA	NA
CDH11	-1.68075954	1.267706284	0.5692172	CDH11	NA	CDH11
XKR4	-1.6124775	1.258232866	0.8841996	XKR4	NA	NA
ID4	-1.71418298	1.249410143	0.5692172	ID4	NA	ID4
SYT9	-1.71749433	1.248318572	0.5692172	SYT9	NA	NA
CSMD3	-1.53036727	1.234721195	0.8841996	CSMD3	NA	CSMD3
NRXN3	-1.67797848	1.232510319	0.5692172	NRXN3	NA	NRXN3
RIMS4	1.571240855	0.806598496	0.8841996	RIMS4	NA	NA
XYLT1	1.922205135	0.80021924	0.1920624	XYLT1	NA	XYLT1
CDC73	1.56862806	0.79791535	0.8841996	CDC73	NA	NA
CLYBL	1.540808211	0.789655223	0.8841996	CLYBL	NA	CLYBL
MITF	1.626351977	0.782694496	0.8841996	MITF	NA	MITF
HNMT	1.641286706	0.779961652	0.8841996	HNMT	NA	HNMT
CNTNAP5	1.867266525	0.778953903	0.1920624	CNTNAP5	NA	CNTNAP5
SMAD7	1.582277207	0.772544417	0.8841996	SMAD7	NA	NA
C2orf3	1.841798128	0.755857562	0.1920624	C2orf3	NA	NA
ACVR1	1.901681292	0.74909317	0.1920624	ACVR1	NA	ACVR1
LMO1	1.553677926	0.745715017	0.8841996	LMO1	NA	NA
CXCR4	2.472439403	0.739130617	0	CXCR4	NA	CXCR4
LRRC7	2.138431829	0.730778933	0.1132952	LRRC7	NA	LRRC7
ZNF618	2.4840875	0.730109049	0	ZNF618	NA	NA
PBX3	2.630409665	0.710301985	0	PBX3	NA	PBX3
KIAA1797	2.297017196	0.701044018	0	KIAA1797	NA	KIAA1797
DYRK2	2.330251518	0.686718348	0	DYRK2	NA	NA
HAS2	2.05747991	0.683146203	0.1132952	HAS2	NA	NA
TGFBR1	2.419917641	0.652633398	0	TGFBR1	NA	NA
ITPR1	3.650092417	0.639788798	0	ITPR1	NA	ITPR1
LOC283392	3.210919415	0.587372463	0	LOC283392	NA	NA
MCTP1	3.610437125	0.580458414	0	MCTP1	NA	MCTP1
SNTG1	4.143762175	0.571025733	0	SNTG1	NA	NA
SORCS1	4.398843879	0.484518816	0	SORCS1	NA	SORCS1
NFE2L3	3.290581592	0.457712017	0	NFE2L3	NA	NA
PTPRK	4.763210281	0.397753704	0	PTPRK	NA	PTPRK
FJX1	5.572196893	0.396179661	0	FJX1	NA	NA
BARD1	3.883937603	0.394424527	0	BARD1	NA	BARD1
NR3C2	6.294811103	0.354365206	0	NR3C2	NA	NR3C2

haCNS only PFOvsPT q<0.01

SYMBOL	PFOvsPT.d	PFOvsPT.FC	PFOvsPT.q	haCNS	caCNS	maCNS

L3MBTL4	-7.31353178	2.854053804	0	L3MBTL4	NA	NA
CTNNBL1	-5.1701204	2.605088071	0	CTNNBL1	NA	NA
PPAPDC1A	-5.09062142	2.375287236	0	PPAPDC1A	NA	NA
CSRP2	-5.78367684	2.305385047	0	CSRP2	NA	NA
RPRM	-3.03115814	2.032294178	0	RPRM	NA	NA
EGLN3	-2.99677255	1.721010124	0	EGLN3	NA	NA
MEGF11	-2.95386633	1.677663997	0	MEGF11	NA	NA
LYPD1	-2.40434003	1.635473385	0	LYPD1	NA	NA
FAM19A5	-2.25379138	1.364349186	0	FAM19A5	NA	NA
NMNAT3	-1.75681692	1.320962511	0.5692172	NMNAT3	NA	NA
PITPNC1	-1.92418927	1.28031768	0.1920624	PITPNC1	NA	NA
CDH22	-1.64073284	1.269176176	0.8841996	CDH22	NA	NA
XKR4	-1.6124775	1.258232866	0.8841996	XKR4	NA	NA
SYT9	-1.71749433	1.248318572	0.5692172	SYT9	NA	NA
RIMS4	1.571240855	0.806598496	0.8841996	RIMS4	NA	NA
CDC73	1.56862806	0.79791535	0.8841996	CDC73	NA	NA
SMAD7	1.582277207	0.772544417	0.8841996	SMAD7	NA	NA
C2orf3	1.841798128	0.755857562	0.1920624	C2orf3	NA	NA
LMO1	1.553677926	0.745715017	0.8841996	LMO1	NA	NA
ZNF618	2.4840875	0.730109049	0	ZNF618	NA	NA
DYRK2	2.330251518	0.686718348	0	DYRK2	NA	NA
HAS2	2.05747991	0.683146203	0.1132952	HAS2	NA	NA
TGFBR1	2.419917641	0.652633398	0	TGFBR1	NA	NA
LOC283392	3.210919415	0.587372463	0	LOC283392	NA	NA
SNTG1	4.143762175	0.571025733	0	SNTG1	NA	NA
NFE2L3	3.290581592	0.457712017	0	NFE2L3	NA	NA
FJX1	5.572196893	0.396179661	0	FJX1	NA	NA

caCNS PFOvsPT q<0.01

SYMBOL	PFOvsPT.d	PFOvsPT.FC	PFOvsPT.q	haCNS	caCNS	maCNS
CBLN2	-14.5548679	11.25652753	0	NA	CBLN2	CBLN2
CA10	-6.12	5.712212733	0	CA10	CA10	CA10
NEUROD1	-7.56103288	4.913636718	0	NA	NEUROD1	NEUROD1
FAM5C	-6.27335413	4.382939739	0	NA	FAM5C	FAM5C
CDH8	-6.06468643	4.185181997	0	CDH8	CDH8	CDH8
CNTNAP2	-5.12221366	3.340737607	0	CNTNAP2	CNTNAP2	CNTNAP2
MEGF10	-7.23068618	3.232916311	0	NA	MEGF10	MEGF10
TOX	-6.24702549	3.160502065	0	TOX	TOX	TOX
IGSF11	-7.55618738	3.097316213	0	IGSF11	IGSF11	IGSF11
SDK1	-8.51713497	2.978886894	0	NA	SDK1	NA
PCDH10	-5.84345127	2.908355915	0	PCDH10	PCDH10	PCDH10
PDZRN4	-5.44764863	2.63707924	0	PDZRN4	PDZRN4	PDZRN4
HS6ST3	-3.6617101	2.521555404	0	HS6ST3	HS6ST3	HS6ST3
PART1	-4.64566281	2.46597251	0	PART1	PART1	PART1
CACNA2D3	-5.01836394	2.373116161	0	CACNA2D3	CACNA2D3	CACNA2D3
PCDH17	-5.06712243	2.341529223	0	NA	PCDH17	PCDH17
FLRT3	-6.03290609	2.315812978	0	NA	FLRT3	FLRT3
CNTN3	-5.12265522	2.1416389	0	NA	CNTN3	CNTN3
LMO4	-5.43330418	2.097623295	0	LMO4	LMO4	LMO4
CNTN4	-4.29563654	2.053456928	0	CNTN4	CNTN4	CNTN4
SLITRK3	-4.2346195	2.044461	0	NA	SLITRK3	NA

MARCH 3	-5.56101305	1.984234907	0	MARCH 3	MARCH 3	NA
DERA	-3.2478245	1.903953655	0	DERA	DERA	DERA
FGF14	-4.16133602	1.89013512	0	NA	FGF14	FGF14
PMP22	-2.1046144	1.881694179	0.1132952	NA	PMP22	NA
ASTN2	-3.537837	1.845172203	0	ASTN2	ASTN2	ASTN2
GBE1	-3.79150602	1.82357854	0	GBE1	GBE1	GBE1
CDCA7L	-3.20491701	1.821544626	0	CDCA7L	CDCA7L	NA
FAM59A	-2.49346488	1.784624664	0	NA	FAM59A	FAM59A
PTPRT	-2.74804604	1.717870851	0	PTPRT	PTPRT	PTPRT
SFRP2	-2.9745118	1.690623422	0	NA	SFRP2	NA
ACCN1	-2.63076477	1.679795978	0	ACCN1	ACCN1	ACCN1
GLIS3	-2.10301732	1.664953031	0.1132952	GLIS3	GLIS3	GLIS3
ASAM	-2.13060341	1.626374035	0.1132952	NA	ASAM	ASAM
CNTN6	-2.8124791	1.62508137	0	CNTN6	CNTN6	CNTN6
LMO3	-2.44233444	1.621965	0	LMO3	LMO3	LMO3
CBLN4	-2.64785393	1.618761792	0	NA	CBLN4	CBLN4
OLFM3	-2.34271783	1.609927878	0	OLFM3	OLFM3	NA
POU3F2	-2.94748566	1.608230126	0	POU3F2	POU3F2	POU3F2
SLIT2	-3.08793809	1.565862657	0	NA	SLIT2	SLIT2
NFIA	-2.02490921	1.547560662	0.1132952	NFIA	NFIA	NFIA
EPHA4	-2.32887211	1.521442732	0	EPHA4	EPHA4	EPHA4
PCDH18	-2.4106968	1.507983221	0	PCDH18	PCDH18	PCDH18
ITGBL1	-2.8160076	1.505764735	0	NA	ITGBL1	ITGBL1
MID1IP1	-3.31841086	1.502607182	0	NA	MID1IP1	MID1IP1
ELAVL2	-2.00023103	1.499451152	0.1132952	ELAVL2	ELAVL2	ELAVL2
TCF7L2	-2.71959235	1.468822822	0	NA	TCF7L2	TCF7L2
SNCAIP	-2.39993038	1.444568076	0	NA	SNCAIP	SNCAIP
KLF6	-2.74045034	1.429321375	0	NA	KLF6	NA
HS3ST1	-2.20065986	1.422808221	0	HS3ST1	HS3ST1	HS3ST1
MN1	-2.37409167	1.376844253	0	NA	MN1	MN1
PRKG1	-2.24761358	1.361509379	0	NA	PRKG1	PRKG1
ROBO1	-2.1733223	1.360443688	0	ROBO1	ROBO1	ROBO1
DUSP6	-1.66485337	1.342443608	0.5692172	DUSP6	DUSP6	DUSP6
PKP4	-2.46392511	1.339819605	0	NA	PKP4	PKP4
SLC7A11	-1.51258674	1.335705596	0.8841996	NA	SLC7A11	SLC7A11
GRM7	-1.99458151	1.333356807	0.1132952	NA	GRM7	GRM7
PBX1	-1.73647124	1.321257503	0.5692172	NA	PBX1	PBX1
ZBTB20	-2.21439732	1.320427888	0	ZBTB20	ZBTB20	ZBTB20
DIAPH3	-1.69113303	1.316907999	0.5692172	DIAPH3	DIAPH3	DIAPH3
AJAP1	-2.03845117	1.315715838	0.1132952	NA	AJAP1	AJAP1
LPHN3	-1.93568195	1.287259152	0.1920624	LPHN3	LPHN3	LPHN3
AKR1B1	-1.88738255	1.266693289	0.1920624	NA	AKR1B1	NA
ZNF703	-1.72195868	1.264914497	0.5692172	NA	ZNF703	ZNF703
DCC	-1.54680033	1.256419694	0.8841996	DCC	DCC	DCC
DCTD	-1.79166421	1.254213126	0.5692172	NA	DCTD	DCTD
ELAVL4	-1.58841102	1.23423612	0.8841996	NA	ELAVL4	ELAVL4
CSMD1	-1.6330538	1.202959298	0.8841996	CSMD1	CSMD1	CSMD1
UNC5C	1.582604759	0.825391046	0.8841996	NA	UNC5C	UNC5C
PTPRD	1.601348957	0.823782065	0.8841996	PTPRD	PTPRD	PTPRD
EFNA5	1.623838337	0.805562936	0.8841996	EFNA5	EFNA5	EFNA5
SATB1	1.865850608	0.798173064	0.1920624	NA	SATB1	SATB1
NR2F2	1.681058537	0.787077506	0.5692172	NR2F2	NR2F2	NR2F2
ATP2B1	1.710136306	0.786368648	0.5692172	NA	ATP2B1	ATP2B1
LRRC4C	1.772317163	0.783808549	0.5692172	LRRC4C	LRRC4C	LRRC4C
ZNF365	1.608012079	0.782204854	0.8841996	ZNF365	ZNF365	ZNF365
FANCL	1.958283069	0.776417081	0.1920624	FANCL	FANCL	FANCL
TCF4	1.72942339	0.770627215	0.5692172	TCF4	TCF4	TCF4

DDHD1	1.642298817	0.769818565	0.8841996	NA	DDHD1	DDHD1
STRBP	1.562599486	0.763233235	0.8841996	NA	STRBP	NA
PFTK1	1.720018754	0.757570187	0.5692172	NA	PFTK1	PFTK1
GRIA3	1.84845098	0.749806617	0.1920624	GRIA3	GRIA3	GRIA3
FAM84A	1.882711005	0.743572731	0.1920624	NA	FAM84A	FAM84A
FBXL4	1.703086334	0.743222085	0.5692172	NA	FBXL4	NA
DOK6	1.968978048	0.741947724	0.1920624	NA	DOK6	NA
PTPRG	1.702970376	0.737949782	0.5692172	PTPRG	PTPRG	PTPRG
ARHGAP15	2.095321106	0.736425741	0.1132952	NA	ARHGAP15	ARHGAP15
ETV5	2.064366806	0.735964832	0.1132952	NA	ETV5	NA
SNX16	2.022273678	0.735425143	0.1132952	SNX16	SNX16	SNX16
CTNNA2	2.349801056	0.726774235	0	CTNNA2	CTNNA2	CTNNA2
FOXP1	2.573537464	0.714898217	0	NA	FOXP1	FOXP1
EDIL3	2.471871838	0.714879738	0	EDIL3	EDIL3	EDIL3
NPAS2	2.413001389	0.711419704	0	NA	NPAS2	NA
CDH13	1.576661194	0.710124456	0.8841996	CDH13	CDH13	CDH13
NLGN1	1.972999378	0.70393173	0.1920624	NLGN1	NLGN1	NLGN1
PPARGC1A	2.525981736	0.696051571	0	PPARGC1A	PPARGC1A	PPARGC1A
VRK2	1.671637439	0.689973897	0.5692172	NA	VRK2	VRK2
GPAM	2.140470994	0.686996974	0.1132952	NA	GPAM	GPAM
GRIK1	2.095029121	0.683951491	0.1132952	GRIK1	GRIK1	GRIK1
DOK5	3.468938151	0.682774508	0	DOK5	DOK5	DOK5
MOCS2	2.692411446	0.681645694	0	NA	MOCS2	MOCS2
EFNB2	2.068676182	0.67820706	0.1132952	EFNB2	EFNB2	EFNB2
PDZRN3	2.399581709	0.662704991	0	NA	PDZRN3	PDZRN3
EPHA7	2.325415831	0.643391453	0	EPHA7	EPHA7	EPHA7
NTNG1	2.56015907	0.6424647	0	NA	NTNG1	NTNG1
SYNPR	2.211211773	0.6402151	0	NA	SYNPR	SYNPR
DPP10	2.354621865	0.6215833	0	DPP10	DPP10	DPP10
ZNF238	2.839086702	0.596913	0	NA	ZNF238	ZNF238
ANTXR2	3.917695399	0.583697192	0	NA	ANTXR2	ANTXR2
BCL11A	2.887541535	0.577424878	0	BCL11A	BCL11A	BCL11A
FST	2.456987371	0.573029999	0	FST	FST	FST
MID1	3.336562507	0.560042328	0	NA	MID1	MID1
OPCML	3.730784512	0.528980935	0	OPCML	OPCML	OPCML
PCDH7	4.99435407	0.528261246	0	PCDH7	PCDH7	PCDH7
CDH9	3.347253803	0.502386394	0	CDH9	CDH9	CDH9
NR2F1	4.533384717	0.490489804	0	NR2F1	NR2F1	NR2F1
FAM19A1	4.348552767	0.488005419	0	FAM19A1	FAM19A1	FAM19A1
NCAM2	3.86902789	0.486966988	0	NCAM2	NCAM2	NA
LPHN2	4.227025654	0.470334365	0	LPHN2	LPHN2	LPHN2
SORCS3	3.775183207	0.469115015	0	SORCS3	SORCS3	SORCS3
ROBO2	5.389565456	0.464744626	0	ROBO2	ROBO2	ROBO2
LHX2	5.766844002	0.463951405	0	NA	LHX2	NA
PCDH9	5.611957099	0.383387329	0	PCDH9	PCDH9	PCDH9
NR4A2	5.372879604	0.310340327	0	NA	NR4A2	NR4A2
NTNG2	5.038729141	0.27228346	0	NA	NTNG2	NA
LRRTM4	5.923967514	0.235613006	0	LRRTM4	LRRTM4	LRRTM4
TRHDE	7.463230607	0.22727706	0	TRHDE	TRHDE	TRHDE
SOX9	12.91514	0.116417689	0	SOX9	SOX9	SOX9

ca-haCNS PFOvsPT q<0.01

SYMBOL	PFOvsPT.d	PFOvsPT.FC	PFOvsPT.q	haCNS	caCNS	maCNS
CBLN2	-14.5548679	11.25652753	0	NA	CBLN2	CBLN2
NEUROD1	-7.56103288	4.913636718	0	NA	NEUROD1	NEUROD1
FAM5C	-6.27335413	4.382939739	0	NA	FAM5C	FAM5C
MEGF10	-7.23068618	3.232916311	0	NA	MEGF10	MEGF10
SDK1	-8.51713497	2.978886894	0	NA	SDK1	NA
PCDH17	-5.06712243	2.341529223	0	NA	PCDH17	PCDH17
FLRT3	-6.03290609	2.315812978	0	NA	FLRT3	FLRT3
CNTN3	-5.12265522	2.1416389	0	NA	CNTN3	CNTN3
SLITRK3	-4.2346195	2.044461	0	NA	SLITRK3	NA
FGF14	-4.16133602	1.89013512	0	NA	FGF14	FGF14
PMP22	-2.1046144	1.881694179	0.1132952	NA	PMP22	NA
FAM59A	-2.49346488	1.784624664	0	NA	FAM59A	FAM59A
SFRP2	-2.9745118	1.690623422	0	NA	SFRP2	NA
ASAM	-2.13060341	1.626374035	0.1132952	NA	ASAM	ASAM
CBLN4	-2.64785393	1.618761792	0	NA	CBLN4	CBLN4
SLIT2	-3.08793809	1.565862657	0	NA	SLIT2	SLIT2
ITGBL1	-2.8160076	1.505764735	0	NA	ITGBL1	ITGBL1
MID1IP1	-3.31841086	1.502607182	0	NA	MID1IP1	MID1IP1
TCF7L2	-2.71959235	1.468822822	0	NA	TCF7L2	TCF7L2
SNCAIP	-2.39993038	1.444568076	0	NA	SNCAIP	SNCAIP
KLF6	-2.74045034	1.429321375	0	NA	KLF6	NA
MN1	-2.37409167	1.376844253	0	NA	MN1	MN1
PRKG1	-2.24761358	1.361509379	0	NA	PRKG1	PRKG1
PKP4	-2.46392511	1.339819605	0	NA	PKP4	PKP4
SLC7A11	-1.51258674	1.335705596	0.8841996	NA	SLC7A11	SLC7A11
GRM7	-1.99458151	1.333356807	0.1132952	NA	GRM7	GRM7
PBX1	-1.73647124	1.321257503	0.5692172	NA	PBX1	PBX1
AJAP1	-2.03845117	1.315715838	0.1132952	NA	AJAP1	AJAP1
AKR1B1	-1.88738255	1.266693289	0.1920624	NA	AKR1B1	NA
ZNF703	-1.72195868	1.264914497	0.5692172	NA	ZNF703	ZNF703
DCTD	-1.79166421	1.254213126	0.5692172	NA	DCTD	DCTD
ELAVL4	-1.58841102	1.23423612	0.8841996	NA	ELAVL4	ELAVL4
UNC5C	1.582604759	0.825391046	0.8841996	NA	UNC5C	UNC5C
SATB1	1.865850608	0.798173064	0.1920624	NA	SATB1	SATB1
ATP2B1	1.710136306	0.786368648	0.5692172	NA	ATP2B1	ATP2B1
DDHD1	1.642298817	0.769818565	0.8841996	NA	DDHD1	DDHD1
STRBP	1.562599486	0.763233235	0.8841996	NA	STRBP	NA
PFTK1	1.720018754	0.757570187	0.5692172	NA	PFTK1	PFTK1
FAM84A	1.882711005	0.743572731	0.1920624	NA	FAM84A	FAM84A
FBXL4	1.703086334	0.743222085	0.5692172	NA	FBXL4	NA
DOK6	1.968978048	0.741947724	0.1920624	NA	DOK6	NA
ARHGAP15	2.095321106	0.736425741	0.1132952	NA	ARHGAP15	ARHGAP15
ETV5	2.064366806	0.735964832	0.1132952	NA	ETV5	NA
FOXP1	2.573537464	0.714898217	0	NA	FOXP1	FOXP1
NPAS2	2.413001389	0.711419704	0	NA	NPAS2	NA
VRK2	1.671637439	0.689973897	0.5692172	NA	VRK2	VRK2
GPAM	2.140470994	0.686996974	0.1132952	NA	GPAM	GPAM
MOCS2	2.692411446	0.681645694	0	NA	MOCS2	MOCS2
PDZRN3	2.399581709	0.662704991	0	NA	PDZRN3	PDZRN3
NTNG1	2.56015907	0.6424647	0	NA	NTNG1	NTNG1
SYNPR	2.211211773	0.6402151	0	NA	SYNPR	SYNPR
ZNF238	2.839086702	0.596913	0	NA	ZNF238	ZNF238

ANTXR2	3.917695399	0.583697192	0	NA	ANTXR2	ANTXR2
MID1	3.336562507	0.560042328	0	NA	MID1	MID1
LHX2	5.766844002	0.463951405	0	NA	LHX2	NA
NR4A2	5.372879604	0.310340327	0	NA	NR4A2	NR4A2
NTNG2	5.038729141	0.27228346	0	NA	NTNG2	NA

caCNS only PFOvsPT q<0.01

SYMBOL	PFOvsPT.d	PFOvsPT.FC	PFOvsPT.q	haCNS	caCNS	maCNS
SDK1	-8.51713497	2.978886894	0	NA	SDK1	NA
SLITRK3	-4.2346195	2.044461	0	NA	SLITRK3	NA
PMP22	-2.1046144	1.881694179	0.1132952	NA	PMP22	NA
SFRP2	-2.9745118	1.690623422	0	NA	SFRP2	NA
KLF6	-2.74045034	1.429321375	0	NA	KLF6	NA
AKR1B1	-1.88738255	1.266693289	0.1920624	NA	AKR1B1	NA
STRBP	1.562599486	0.763233235	0.8841996	NA	STRBP	NA
FBXL4	1.703086334	0.743222085	0.5692172	NA	FBXL4	NA
DOK6	1.968978048	0.741947724	0.1920624	NA	DOK6	NA
ETV5	2.064366806	0.735964832	0.1132952	NA	ETV5	NA
NPAS2	2.413001389	0.711419704	0	NA	NPAS2	NA
LHX2	5.766844002	0.463951405	0	NA	LHX2	NA
NTNG2	5.038729141	0.27228346	0	NA	NTNG2	NA

maCNS PFOvsPT q<0.01

SYMBOL	PFOvsPT.d	PFOvsPT.FC	PFOvsPT.q	haCNS	caCNS	maCNS
CBLN2	-14.5548679	11.25652753	0	NA	CBLN2	CBLN2
CA10	-6.12	5.712212733	0	CA10	CA10	CA10
FSTL5	-9.13405901	5.144152677	0	NA	NA	FSTL5
NEUROD1	-7.56103288	4.913636718	0	NA	NEUROD1	NEUROD1
CBLN1	-7.70411465	4.807194815	0	NA	NA	CBLN1
BDNF	-6.02848348	4.730594258	0	NA	NA	BDNF
ZNF423	-6.90224806	4.597465111	0	NA	NA	ZNF423
FAM5C	-6.27335413	4.382939739	0	NA	FAM5C	FAM5C
CDH8	-6.06468643	4.185181997	0	CDH8	CDH8	CDH8
TFAP2D	-3.50782293	3.658017759	0	TFAP2D	NA	TFAP2D
CNTNAP2	-5.12221366	3.340737607	0	CNTNAP2	CNTNAP2	CNTNAP2
STXBP6	-7.55969727	3.299394519	0	STXBP6	NA	STXBP6
MEGF10	-7.23068618	3.232916311	0	NA	MEGF10	MEGF10
MEIS2	-8.05642148	3.203435352	0	MEIS2	NA	MEIS2
TOX	-6.24702549	3.160502065	0	TOX	TOX	TOX
IGSF11	-7.55618738	3.097316213	0	IGSF11	IGSF11	IGSF11
PCDH10	-5.84345127	2.908355915	0	PCDH10	PCDH10	PCDH10
CHRDL1	-4.52809552	2.883850704	0	CHRDL1	NA	CHRDL1
PTX3	-5.00633748	2.692349177	0	NA	NA	PTX3
PDZRN4	-5.44764863	2.63707924	0	PDZRN4	PDZRN4	PDZRN4
BMP2	-4.21371593	2.614685809	0	BMP2	NA	BMP2
HS6ST3	-3.6617101	2.521555404	0	HS6ST3	HS6ST3	HS6ST3
PART1	-4.64566281	2.46597251	0	PART1	PART1	PART1
EMX2	-3.67376004	2.379665984	0	EMX2	NA	EMX2

CACNA2D3	-5.01836394	2.373116161	0	CACNA2D3	CACNA2D3	CACNA2D3
ST6GALNAC5	-6.09921617	2.366577756	0	NA	NA	ST6GALNAC5
PCDH17	-5.06712243	2.341529223	0	NA	PCDH17	PCDH17
FLRT3	-6.03290609	2.315812978	0	NA	FLRT3	FLRT3
ANKRD32	-5.24414946	2.29550366	0	ANKRD32	NA	ANKRD32
PTER	-3.93798799	2.213952941	0	PTER	NA	PTER
CNTN3	-5.12265522	2.1416389	0	NA	CNTN3	CNTN3
LMO4	-5.43330418	2.097623295	0	LMO4	LMO4	LMO4
MC4R	-2.74483075	2.077390683	0	NA	NA	MC4R
CYP26A1	-2.91046367	2.070475578	0	NA	NA	CYP26A1
CNTN4	-4.29563654	2.053456928	0	CNTN4	CNTN4	CNTN4
LRRC3B	-4.8019253	1.974090641	0	NA	NA	LRRC3B
DERA	-3.2478245	1.903953655	0	DERA	DERA	DERA
FGF14	-4.16133602	1.89013512	0	NA	FGF14	FGF14
ASTN2	-3.537837	1.845172203	0	ASTN2	ASTN2	ASTN2
PLK2	-4.18086128	1.840616327	0	NA	NA	PLK2
GBE1	-3.79150602	1.82357854	0	GBE1	GBE1	GBE1
KCNQ5	-3.4225801	1.815481633	0	NA	NA	KCNQ5
NEBL	-3.95962297	1.814775522	0	NA	NA	NEBL
FAM59A	-2.49346488	1.784624664	0	NA	FAM59A	FAM59A
HS3ST5	-2.68310547	1.772104321	0	HS3ST5	NA	HS3ST5
ADAMTS16	-2.36220106	1.760054514	0	ADAMTS16	NA	ADAMTS16
RPH3A	-1.83743736	1.750384294	0.1920624	NA	NA	RPH3A
BAMBI	-2.54868324	1.746195742	0	NA	NA	BAMBI
TNFAIP3	-1.74599811	1.72654034	0.5692172	NA	NA	TNFAIP3
PTPRT	-2.74804604	1.717870851	0	PTPRT	PTPRT	PTPRT
NRIP1	-3.2856362	1.715123759	0	NA	NA	NRIP1
CDH12	-3.09316342	1.701936008	0	NA	NA	CDH12
CXXC4	-3.9002226	1.683449791	0	NA	NA	CXXC4
ACCN1	-2.63076477	1.679795978	0	ACCN1	ACCN1	ACCN1
GLIS3	-2.10301732	1.664953031	0.1132952	GLIS3	GLIS3	GLIS3
PHLDA1	-4.05016913	1.659226237	0	NA	NA	PHLDA1
KIT	-3.17697957	1.637540014	0	NA	NA	KIT
POU6F2	-2.68054389	1.636633705	0	POU6F2	NA	POU6F2
UST	-2.35119547	1.632375331	0	NA	NA	UST
ASAM	-2.13060341	1.626374035	0.1132952	NA	ASAM	ASAM
SOCS2	-2.38288631	1.626189776	0	NA	NA	SOCS2
CNTN6	-2.8124791	1.62508137	0	CNTN6	CNTN6	CNTN6
LMO3	-2.44233444	1.621965	0	LMO3	LMO3	LMO3
CBLN4	-2.64785393	1.618761792	0	NA	CBLN4	CBLN4
ADCYAP1	-3.09707721	1.615928824	0	NA	NA	ADCYAP1
POU3F2	-2.94748566	1.608230126	0	POU3F2	POU3F2	POU3F2
THRB	-2.1344228	1.601725202	0.1132952	NA	NA	THRB
FAM5B	-2.75243221	1.600873707	0	NA	NA	FAM5B
SLIT2	-3.08793809	1.565862657	0	NA	SLIT2	SLIT2
NFIA	-2.02490921	1.547560662	0.1132952	NFIA	NFIA	NFIA
TSPAN6	-1.97172607	1.546995067	0.1920624	NA	NA	TSPAN6
QPCT	-1.69896847	1.522678029	0.5692172	NA	NA	QPCT
EPHA4	-2.32887211	1.521442732	0	EPHA4	EPHA4	EPHA4
PCDH18	-2.4106968	1.507983221	0	PCDH18	PCDH18	PCDH18
ITGBL1	-2.8160076	1.505764735	0	NA	ITGBL1	ITGBL1
MID1IP1	-3.31841086	1.502607182	0	NA	MID1IP1	MID1IP1
CTGF	-1.7315289	1.501443714	0.5692172	NA	NA	CTGF
ELAVL2	-2.00023103	1.499451152	0.1132952	ELAVL2	ELAVL2	ELAVL2
ITGA9	-2.81551544	1.488179049	0	NA	NA	ITGA9
C7orf36	-2.15755103	1.485669325	0.1132952	NA	NA	C7orf36
ADAMTS3	-2.41327432	1.484830156	0	NA	NA	ADAMTS3

POU3F3	-2.37286138	1.478080999	0	POU3F3	NA	POU3F3
LRRTM1	-1.74257338	1.476807726	0.5692172	LRRTM1	NA	LRRTM1
TCF7L2	-2.71959235	1.468822822	0	NA	TCF7L2	TCF7L2
COL12A1	-2.56005564	1.458176067	0	NA	NA	COL12A1
PARP8	-1.89900276	1.453057443	0.1920624	PARP8	NA	PARP8
SNCAIP	-2.39993038	1.444568076	0	NA	SNCAIP	SNCAIP
LDB2	-2.89311943	1.4399603	0	LDB2	NA	LDB2
GAS1	-2.82070171	1.439256816	0	NA	NA	GAS1
RRAS2	-2.21380586	1.436160734	0	NA	NA	RRAS2
RFK	-3.07661835	1.428068941	0	NA	NA	RFK
HS3ST1	-2.20065986	1.422808221	0	HS3ST1	HS3ST1	HS3ST1
SUPT3H	-1.88285687	1.410333462	0.1920624	SUPT3H	NA	SUPT3H
H2AFY2	-2.90931696	1.401067056	0	NA	NA	H2AFY2
PLCB4	-1.9883246	1.386326283	0.1920624	NA	NA	PLCB4
CHN1	-2.31384252	1.384545514	0	NA	NA	CHN1
MN1	-2.37409167	1.376844253	0	NA	MN1	MN1
SLITRK1	-2.3569568	1.363953962	0	SLITRK1	NA	SLITRK1
PRKG1	-2.24761358	1.361509379	0	NA	PRKG1	PRKG1
ROBO1	-2.1733223	1.360443688	0	ROBO1	ROBO1	ROBO1
DIO2	-1.68458127	1.345644949	0.5692172	NA	NA	DIO2
DUSP6	-1.66485337	1.342443608	0.5692172	DUSP6	DUSP6	DUSP6
PKP4	-2.46392511	1.339819605	0	NA	PKP4	PKP4
TNFRSF21	-2.27712882	1.337971486	0	TNFRSF21	NA	TNFRSF21
APBB2	-1.93694713	1.337819274	0.1920624	NA	NA	APBB2
SH3D19	-2.18016659	1.337417312	0	NA	NA	SH3D19
ZADH2	-1.93886437	1.336059328	0.1920624	NA	NA	ZADH2
SLC7A11	-1.51258674	1.335705596	0.8841996	NA	SLC7A11	SLC7A11
GRM7	-1.99458151	1.333356807	0.1132952	NA	GRM7	GRM7
QDPR	-2.19795663	1.330727363	0	NA	NA	QDPR
GRIK2	-2.10003409	1.327840632	0.1132952	GRIK2	NA	GRIK2
AADAT	-2.50286153	1.324581374	0	AADAT	NA	AADAT
DPF3	-1.99485721	1.322803272	0.1132952	NA	NA	DPF3
PRKD1	-1.92726666	1.322584174	0.1920624	NA	NA	PRKD1
GRM5	-2.08737967	1.322098237	0.1132952	NA	NA	GRM5
PBX1	-1.73647124	1.321257503	0.5692172	NA	PBX1	PBX1
ADRA2A	-2.2026569	1.32088762	0	NA	NA	ADRA2A
NINJ2	-1.80627593	1.320586093	0.5692172	NA	NA	NINJ2
ZBTB20	-2.21439732	1.320427888	0	ZBTB20	ZBTB20	ZBTB20
DIAPH3	-1.69113303	1.316907999	0.5692172	DIAPH3	DIAPH3	DIAPH3
KCNK2	-1.6590451	1.315874513	0.5692172	NA	NA	KCNK2
AJAP1	-2.03845117	1.315715838	0.1132952	NA	AJAP1	AJAP1
CRADD	-1.62279567	1.302153136	0.8841996	NA	NA	CRADD
LPHN3	-1.93568195	1.287259152	0.1920624	LPHN3	LPHN3	LPHN3
RUNX2	-1.6858028	1.284678259	0.5692172	NA	NA	RUNX2
RSPO3	-1.53076447	1.282867266	0.8841996	NA	NA	RSPO3
TBR1	-1.78527877	1.281547876	0.5692172	TBR1	NA	TBR1
E2F3	-1.74676342	1.278940873	0.5692172	NA	NA	E2F3
MYO1B	-1.65357423	1.269053424	0.8841996	NA	NA	MYO1B
CDH11	-1.68075954	1.267706284	0.5692172	CDH11	NA	CDH11
ZNF703	-1.72195868	1.264914497	0.5692172	NA	ZNF703	ZNF703
MAOA	-1.72301752	1.264390327	0.5692172	NA	NA	MAOA
CACNA1D	-1.80598597	1.264322789	0.5692172	NA	NA	CACNA1D
WWP2	-1.66442974	1.262947586	0.5692172	NA	NA	WWP2
MYC	-1.84922178	1.260900838	0.1920624	NA	NA	MYC
DCC	-1.54680033	1.256419694	0.8841996	DCC	DCC	DCC
DCTD	-1.79166421	1.254213126	0.5692172	NA	DCTD	DCTD
ID4	-1.71418298	1.249410143	0.5692172	ID4	NA	ID4

CA8	-1.72634986	1.239795344	0.5692172	NA	NA	CA8
CSMD3	-1.53036727	1.234721195	0.8841996	CSMD3	NA	CSMD3
ELAVL4	-1.58841102	1.23423612	0.8841996	NA	ELAVL4	ELAVL4
NRXN3	-1.67797848	1.232510319	0.5692172	NRXN3	NA	NRXN3
ZNF706	-1.84899918	1.22405684	0.1920624	NA	NA	ZNF706
ZNF395	-1.59880805	1.21514445	0.8841996	NA	NA	ZNF395
KCNF1	-1.62789501	1.214471747	0.8841996	NA	NA	KCNF1
ARL4C	-1.70895532	1.213335346	0.5692172	NA	NA	ARL4C
CSMD1	-1.6330538	1.202959298	0.8841996	CSMD1	CSMD1	CSMD1
UNC5C	1.582604759	0.825391046	0.8841996	NA	UNC5C	UNC5C
PTPRD	1.601348957	0.823782065	0.8841996	PTPRD	PTPRD	PTPRD
DGKH	1.670851083	0.817662921	0.5692172	NA	NA	DGKH
RHOBTB1	1.520346451	0.806511173	0.8841996	NA	NA	RHOBTB1
EFNA5	1.623838337	0.805562936	0.8841996	EFNA5	EFNA5	EFNA5
ITGA4	1.554255687	0.803971291	0.8841996	NA	NA	ITGA4
CACNB2	1.758489199	0.803207464	0.5692172	NA	NA	CACNB2
XYLT1	1.922205135	0.80021924	0.1920624	XYLT1	NA	XYLT1
KBTBD8	1.572020284	0.799328082	0.8841996	NA	NA	KBTBD8
SATB1	1.865850608	0.798173064	0.1920624	NA	SATB1	SATB1
TLN2	1.634174676	0.797965962	0.8841996	NA	NA	TLN2
GRM3	1.796391511	0.795173215	0.5692172	NA	NA	GRM3
AMIGO2	1.538916893	0.790467469	0.8841996	NA	NA	AMIGO2
CLYBL	1.540808211	0.789655223	0.8841996	CLYBL	NA	CLYBL
GPD2	1.594044077	0.789623285	0.8841996	NA	NA	GPD2
DNAJC1	1.883652286	0.787195104	0.1920624	NA	NA	DNAJC1
NR2F2	1.681058537	0.787077506	0.5692172	NR2F2	NR2F2	NR2F2
ATP2B1	1.710136306	0.786368648	0.5692172	NA	ATP2B1	ATP2B1
LRRC4C	1.772317163	0.783808549	0.5692172	LRRC4C	LRRC4C	LRRC4C
MITF	1.626351977	0.782694496	0.8841996	MITF	NA	MITF
ZNF365	1.608012079	0.782204854	0.8841996	ZNF365	ZNF365	ZNF365
AP1S2	2.177174512	0.781873197	0.1132952	NA	NA	AP1S2
HNMT	1.641286706	0.779961652	0.8841996	HNMT	NA	HNMT
AMMECR1	1.789781392	0.779275819	0.5692172	NA	NA	AMMECR1
CNTNAP5	1.867266525	0.778953903	0.1920624	CNTNAP5	NA	CNTNAP5
FANCL	1.958283069	0.776417081	0.1920624	FANCL	FANCL	FANCL
NR4A3	2.057562608	0.775546131	0.1132952	NA	NA	NR4A3
BRAF	1.606726902	0.773847676	0.8841996	NA	NA	BRAF
TCF4	1.72942339	0.770627215	0.5692172	TCF4	TCF4	TCF4
DDHD1	1.642298817	0.769818565	0.8841996	NA	DDHD1	DDHD1
IGSF21	1.819825649	0.764397197	0.1920624	NA	NA	IGSF21
TRIM44	1.980988715	0.763210785	0.1920624	NA	NA	TRIM44
HCN1	1.876834754	0.759545131	0.1920624	NA	NA	HCN1
PFTK1	1.720018754	0.757570187	0.5692172	NA	PFTK1	PFTK1
DPYD	1.91158004	0.757162176	0.1920624	NA	NA	DPYD
PTPRZ1	2.110731279	0.756312617	0.1132952	NA	NA	PTPRZ1
PAG1	1.992347617	0.753555654	0.1920624	NA	NA	PAG1
GRIA3	1.84845098	0.749806617	0.1920624	GRIA3	GRIA3	GRIA3
ACVR1	1.901681292	0.74909317	0.1920624	ACVR1	NA	ACVR1
RPS6KA3	2.039452923	0.748799368	0.1132952	NA	NA	RPS6KA3
FAM84A	1.882711005	0.743572731	0.1920624	NA	FAM84A	FAM84A
SLC44A5	1.508018492	0.740117703	0.8841996	NA	NA	SLC44A5
CXCR4	2.472439403	0.739130617	0	CXCR4	NA	CXCR4
PTPRG	1.702970376	0.737949782	0.5692172	PTPRG	PTPRG	PTPRG
ARHGAP15	2.095321106	0.736425741	0.1132952	NA	ARHGAP15	ARHGAP15
STIM2	1.601732135	0.736379264	0.8841996	NA	NA	STIM2
SNX16	2.022273678	0.735425143	0.1132952	SNX16	SNX16	SNX16
PHF17	1.940661664	0.735165164	0.1920624	NA	NA	PHF17

NEDD4	2.135901336	0.73156625	0.1132952	NA	NA	NEDD4
CDCA7	2.32970259	0.730819321	0	NA	NA	CDCA7
LRRC7	2.138431829	0.730778933	0.1132952	LRRC7	NA	LRRC7
ETV1	1.869400066	0.729205933	0.1920624	NA	NA	ETV1
CTNNA2	2.349801056	0.726774235	0	CTNNA2	CTNNA2	CTNNA2
C18orf1	2.627212485	0.726582856	0	NA	NA	C18orf1
BCL2	1.626209371	0.724973607	0.8841996	NA	NA	BCL2
OMG	2.048961701	0.722212659	0.1132952	NA	NA	OMG
COMMD2	2.67383655	0.720154885	0	NA	NA	COMMD2
RFC3	2.091418981	0.715670472	0.1132952	NA	NA	RFC3
FOXP1	2.573537464	0.714898217	0	NA	FOXP1	FOXP1
EDIL3	2.471871838	0.714879738	0	EDIL3	EDIL3	EDIL3
FBXL17	1.890691069	0.714815869	0.1920624	NA	NA	FBXL17
PBX3	2.630409665	0.710301985	0	PBX3	NA	PBX3
CDH13	1.576661194	0.710124456	0.8841996	CDH13	CDH13	CDH13
NLGN1	1.972999378	0.70393173	0.1920624	NLGN1	NLGN1	NLGN1
MGST1	1.860475221	0.701691591	0.1920624	NA	NA	MGST1
KIAA1797	2.297017196	0.701044018	0	KIAA1797	NA	KIAA1797
SEZ6L	2.383782992	0.700835855	0	NA	NA	SEZ6L
PPARGC1A	2.525981736	0.696051571	0	PPARGC1A	PPARGC1A	PPARGC1A
VRK2	1.671637439	0.689973897	0.5692172	NA	VRK2	VRK2
ANK2	1.980579316	0.689171439	0.1920624	NA	NA	ANK2
MAN2A1	1.735773326	0.688175588	0.5692172	NA	NA	MAN2A1
GPAM	2.140470994	0.686996974	0.1132952	NA	GPAM	GPAM
GRIK1	2.095029121	0.683951491	0.1132952	GRIK1	GRIK1	GRIK1
DOK5	3.468938151	0.682774508	0	DOK5	DOK5	DOK5
MOCS2	2.692411446	0.681645694	0	NA	MOCS2	MOCS2
PAPPA2	3.35479031	0.678214016	0	NA	NA	PAPPA2
EFNB2	2.068676182	0.67820706	0.1132952	EFNB2	EFNB2	EFNB2
CAMK4	2.598144006	0.673869172	0	NA	NA	CAMK4
PDGFD	2.180255964	0.672588258	0.1132952	NA	NA	PDGFD
UFM1	2.580881702	0.671223127	0	NA	NA	UFM1
FRZB	2.87648549	0.666397665	0	NA	NA	FRZB
SATB2	2.013867357	0.665230402	0.1132952	NA	NA	SATB2
PDZRN3	2.399581709	0.662704991	0	NA	PDZRN3	PDZRN3
NCOA2	2.324436097	0.655431563	0	NA	NA	NCOA2
KCNJ2	1.894681369	0.651676841	0.1920624	NA	NA	KCNJ2
DACT1	3.761208821	0.651434098	0	NA	NA	DACT1
PTDSS1	3.51016526	0.65010501	0	NA	NA	PTDSS1
CPNE4	2.235536593	0.649730283	0	NA	NA	CPNE4
B3GALT2	3.186066515	0.643552774	0	NA	NA	B3GALT2
EPHA7	2.325415831	0.643391453	0	EPHA7	EPHA7	EPHA7
NTNG1	2.56015907	0.6424647	0	NA	NTNG1	NTNG1
SYNPR	2.211211773	0.6402151	0	NA	SYNPR	SYNPR
ITPR1	3.650092417	0.639788798	0	ITPR1	NA	ITPR1
LRRN3	3.792313283	0.62994277	0	NA	NA	LRRN3
DPP10	2.354621865	0.6215833	0	DPP10	DPP10	DPP10
PLEKHH2	2.707449053	0.600841423	0	NA	NA	PLEKHH2
ZNF238	2.839086702	0.596913	0	NA	ZNF238	ZNF238
ANTXR2	3.917695399	0.583697192	0	NA	ANTXR2	ANTXR2
MCTP1	3.610437125	0.580458414	0	MCTP1	NA	MCTP1
TNFAIP8	2.545221104	0.577656874	0	NA	NA	TNFAIP8
BCL11A	2.887541535	0.577424878	0	BCL11A	BCL11A	BCL11A
FST	2.456987371	0.573029999	0	FST	FST	FST
MET	3.694266312	0.562007528	0	NA	NA	MET
MID1	3.336562507	0.560042328	0	NA	MID1	MID1
NR3C1	3.988644401	0.55590161	0	NA	NA	NR3C1

INHBA	3.850344657	0.535772384	0	NA	NA	INHBA
KCNT2	2.308815908	0.535764159	0	NA	NA	KCNT2
OPCML	3.730784512	0.528980935	0	OPCML	OPCML	OPCML
PCDH7	4.99435407	0.528261246	0	PCDH7	PCDH7	PCDH7
LDLRAD3	4.033439074	0.510085712	0	NA	NA	LDLRAD3
CDH9	3.347253803	0.502386394	0	CDH9	CDH9	CDH9
GRB14	2.572872656	0.493036094	0	NA	NA	GRB14
NR2F1	4.533384717	0.490489804	0	NR2F1	NR2F1	NR2F1
FAM19A1	4.348552767	0.488005419	0	FAM19A1	FAM19A1	FAM19A1
SORCS1	4.398843879	0.484518816	0	SORCS1	NA	SORCS1
GPR22	3.324830791	0.478356979	0	NA	NA	GPR22
PLCXD3	4.26694021	0.476284155	0	NA	NA	PLCXD3
LPHN2	4.227025654	0.470334365	0	LPHN2	LPHN2	LPHN2
SORCS3	3.775183207	0.469115015	0	SORCS3	SORCS3	SORCS3
ROBO2	5.389565456	0.464744626	0	ROBO2	ROBO2	ROBO2
RSPO2	4.472734211	0.456294973	0	NA	NA	RSPO2
MPP6	2.827116918	0.455774816	0	NA	NA	MPP6
DGKI	2.8821568	0.445093854	0	NA	NA	DGKI
ST6GAL2	4.531853789	0.443982207	0	NA	NA	ST6GAL2
LGI1	5.01261104	0.4257431	0	NA	NA	LGI1
PROK2	3.992329864	0.403362522	0	NA	NA	PROK2
PTPRK	4.763210281	0.397753704	0	PTPRK	NA	PTPRK
BARD1	3.883937603	0.394424527	0	BARD1	NA	BARD1
DACH2	5.39837332	0.393896261	0	NA	NA	DACH2
PCDH9	5.611957099	0.383387329	0	PCDH9	PCDH9	PCDH9
AKT3	5.224519542	0.382303266	0	NA	NA	AKT3
TGFB2	5.287867211	0.380547566	0	NA	NA	TGFB2
NR3C2	6.294811103	0.354365206	0	NR3C2	NA	NR3C2
TLE1	9.233379941	0.333319215	0	NA	NA	TLE1
NR4A2	5.372879604	0.310340327	0	NA	NR4A2	NR4A2
THBS1	5.220709261	0.278367583	0	NA	NA	THBS1
LRRTM4	5.923967514	0.235613006	0	LRRTM4	LRRTM4	LRRTM4
TRHDE	7.463230607	0.22727706	0	TRHDE	TRHDE	TRHDE
NPY	10.48770135	0.152609434	0	NA	NA	NPY
PCSK2	11.20597583	0.125898457	0	NA	NA	PCSK2
SOX9	12.91514	0.116417689	0	SOX9	SOX9	SOX9

ma-haCNS PFOvsPT q<0.01

SYMBOL	PFOvsPT.d	PFOvsPT.FC	PFOvsPT.q	haCNS	caCNS	maCNS
CBLN2	-14.5548679	11.25652753	0	NA	CBLN2	CBLN2
FSTL5	-9.13405901	5.144152677	0	NA	NA	FSTL5
NEUROD1	-7.56103288	4.913636718	0	NA	NEUROD1	NEUROD1
CBLN1	-7.70411465	4.807194815	0	NA	NA	CBLN1
BDNF	-6.02848348	4.730594258	0	NA	NA	BDNF
ZNF423	-6.90224806	4.597465111	0	NA	NA	ZNF423
FAM5C	-6.27335413	4.382939739	0	NA	FAM5C	FAM5C
MEGF10	-7.23068618	3.232916311	0	NA	MEGF10	MEGF10
PTX3	-5.00633748	2.692349177	0	NA	NA	PTX3
ST6GALNAC5	-6.09921617	2.366577756	0	NA	NA	ST6GALNAC5

PCDH17	-5.06712243	2.341529223	0	NA	PCDH17	PCDH17
FLRT3	-6.03290609	2.315812978	0	NA	FLRT3	FLRT3
CNTN3	-5.12265522	2.1416389	0	NA	CNTN3	CNTN3
MC4R	-2.74483075	2.077390683	0	NA	NA	MC4R
CYP26A1	-2.91046367	2.070475578	0	NA	NA	CYP26A1
LRRC3B	-4.8019253	1.974090641	0	NA	NA	LRRC3B
FGF14	-4.16133602	1.89013512	0	NA	FGF14	FGF14
PLK2	-4.18086128	1.840616327	0	NA	NA	PLK2
KCNQ5	-3.4225801	1.815481633	0	NA	NA	KCNQ5
NEBL	-3.95962297	1.814775522	0	NA	NA	NEBL
FAM59A	-2.49346488	1.784624664	0	NA	FAM59A	FAM59A
RPH3A	-1.83743736	1.750384294	0.1920624	NA	NA	RPH3A
BAMBI	-2.54868324	1.746195742	0	NA	NA	BAMBI
TNFAIP3	-1.74599811	1.72654034	0.5692172	NA	NA	TNFAIP3
NRIP1	-3.2856362	1.715123759	0	NA	NA	NRIP1
CDH12	-3.09316342	1.701936008	0	NA	NA	CDH12
CXXC4	-3.9002226	1.683449791	0	NA	NA	CXXC4
PHLDA1	-4.05016913	1.659226237	0	NA	NA	PHLDA1
KIT	-3.17697957	1.637540014	0	NA	NA	KIT
UST	-2.35119547	1.632375331	0	NA	NA	UST
ASAM	-2.13060341	1.626374035	0.1132952	NA	ASAM	ASAM
SOCS2	-2.38288631	1.626189776	0	NA	NA	SOCS2
CBLN4	-2.64785393	1.618761792	0	NA	CBLN4	CBLN4
ADCYAP1	-3.09707721	1.615928824	0	NA	NA	ADCYAP1
THRB	-2.1344228	1.601725202	0.1132952	NA	NA	THRB
FAM5B	-2.75243221	1.600873707	0	NA	NA	FAM5B
SLIT2	-3.08793809	1.565862657	0	NA	SLIT2	SLIT2
TSPAN6	-1.97172607	1.546995067	0.1920624	NA	NA	TSPAN6
QPCT	-1.69896847	1.522678029	0.5692172	NA	NA	QPCT
ITGBL1	-2.8160076	1.505764735	0	NA	ITGBL1	ITGBL1
MID1IP1	-3.31841086	1.502607182	0	NA	MID1IP1	MID1IP1
CTGF	-1.7315289	1.501443714	0.5692172	NA	NA	CTGF
ITGA9	-2.81551544	1.488179049	0	NA	NA	ITGA9
C7orf36	-2.15755103	1.485669325	0.1132952	NA	NA	C7orf36
ADAMTS3	-2.41327432	1.484830156	0	NA	NA	ADAMTS3
TCF7L2	-2.71959235	1.468822822	0	NA	TCF7L2	TCF7L2
COL12A1	-2.56005564	1.458176067	0	NA	NA	COL12A1
SNCAIP	-2.39993038	1.444568076	0	NA	SNCAIP	SNCAIP
GAS1	-2.82070171	1.439256816	0	NA	NA	GAS1
RRAS2	-2.21380586	1.436160734	0	NA	NA	RRAS2
RFK	-3.07661835	1.428068941	0	NA	NA	RFK
H2AFY2	-2.90931696	1.401067056	0	NA	NA	H2AFY2
PLCB4	-1.9883246	1.386326283	0.1920624	NA	NA	PLCB4
CHN1	-2.31384252	1.384545514	0	NA	NA	CHN1
MN1	-2.37409167	1.376844253	0	NA	MN1	MN1
PRKG1	-2.24761358	1.361509379	0	NA	PRKG1	PRKG1
DIO2	-1.68458127	1.345644949	0.5692172	NA	NA	DIO2
PKP4	-2.46392511	1.339819605	0	NA	PKP4	PKP4
APBB2	-1.93694713	1.337819274	0.1920624	NA	NA	APBB2
SH3D19	-2.18016659	1.337417312	0	NA	NA	SH3D19
ZADH2	-1.93886437	1.336059328	0.1920624	NA	NA	ZADH2
SLC7A11	-1.51258674	1.335705596	0.8841996	NA	SLC7A11	SLC7A11
GRM7	-1.99458151	1.333356807	0.1132952	NA	GRM7	GRM7
QDPR	-2.19795663	1.330727363	0	NA	NA	QDPR
DPF3	-1.99485721	1.322803272	0.1132952	NA	NA	DPF3
PRKD1	-1.92726666	1.322584174	0.1920624	NA	NA	PRKD1
GRM5	-2.08737967	1.322098237	0.1132952	NA	NA	GRM5

PBX1	-1.73647124	1.321257503	0.5692172	NA	PBX1	PBX1
ADRA2A	-2.2026569	1.32088762	0	NA	NA	ADRA2A
NINJ2	-1.80627593	1.320586093	0.5692172	NA	NA	NINJ2
KCNK2	-1.6590451	1.315874513	0.5692172	NA	NA	KCNK2
AJAP1	-2.03845117	1.315715838	0.1132952	NA	AJAP1	AJAP1
CRADD	-1.62279567	1.302153136	0.8841996	NA	NA	CRADD
RUNX2	-1.6858028	1.284678259	0.5692172	NA	NA	RUNX2
RSPO3	-1.53076447	1.282867266	0.8841996	NA	NA	RSPO3
E2F3	-1.74676342	1.278940873	0.5692172	NA	NA	E2F3
MYO1B	-1.65357423	1.269053424	0.8841996	NA	NA	MYO1B
ZNF703	-1.72195868	1.264914497	0.5692172	NA	ZNF703	ZNF703
MAOA	-1.72301752	1.264390327	0.5692172	NA	NA	MAOA
CACNA1D	-1.80598597	1.264322789	0.5692172	NA	NA	CACNA1D
WWP2	-1.66442974	1.262947586	0.5692172	NA	NA	WWP2
MYC	-1.84922178	1.260900838	0.1920624	NA	NA	MYC
DCTD	-1.79166421	1.254213126	0.5692172	NA	DCTD	DCTD
CA8	-1.72634986	1.239795344	0.5692172	NA	NA	CA8
ELAVL4	-1.58841102	1.23423612	0.8841996	NA	ELAVL4	ELAVL4
ZNF706	-1.84899918	1.22405684	0.1920624	NA	NA	ZNF706
ZNF395	-1.59880805	1.21514445	0.8841996	NA	NA	ZNF395
KCNF1	-1.62789501	1.214471747	0.8841996	NA	NA	KCNF1
ARL4C	-1.70895532	1.213335346	0.5692172	NA	NA	ARL4C
UNC5C	1.582604759	0.825391046	0.8841996	NA	UNC5C	UNC5C
DGKH	1.670851083	0.817662921	0.5692172	NA	NA	DGKH
RHOBTB1	1.520346451	0.806511173	0.8841996	NA	NA	RHOBTB1
ITGA4	1.554255687	0.803971291	0.8841996	NA	NA	ITGA4
CACNB2	1.758489199	0.803207464	0.5692172	NA	NA	CACNB2
KBTBD8	1.572020284	0.799328082	0.8841996	NA	NA	KBTBD8
SATB1	1.865850608	0.798173064	0.1920624	NA	SATB1	SATB1
TLN2	1.634174676	0.797965962	0.8841996	NA	NA	TLN2
GRM3	1.796391511	0.795173215	0.5692172	NA	NA	GRM3
AMIGO2	1.538916893	0.790467469	0.8841996	NA	NA	AMIGO2
GPD2	1.594044077	0.789623285	0.8841996	NA	NA	GPD2
DNAJC1	1.883652286	0.787195104	0.1920624	NA	NA	DNAJC1
ATP2B1	1.710136306	0.786368648	0.5692172	NA	ATP2B1	ATP2B1
AP1S2	2.177174512	0.781873197	0.1132952	NA	NA	AP1S2
AMMECR1	1.789781392	0.779275819	0.5692172	NA	NA	AMMECR1
NR4A3	2.057562608	0.775546131	0.1132952	NA	NA	NR4A3
BRAF	1.606726902	0.773847676	0.8841996	NA	NA	BRAF
DDHD1	1.642298817	0.769818565	0.8841996	NA	DDHD1	DDHD1
IGSF21	1.819825649	0.764397197	0.1920624	NA	NA	IGSF21
TRIM44	1.980988715	0.763210785	0.1920624	NA	NA	TRIM44
HCN1	1.876834754	0.759545131	0.1920624	NA	NA	HCN1
PFTK1	1.720018754	0.757570187	0.5692172	NA	PFTK1	PFTK1
DPYD	1.91158004	0.757162176	0.1920624	NA	NA	DPYD
PTPRZ1	2.110731279	0.756312617	0.1132952	NA	NA	PTPRZ1
PAG1	1.992347617	0.753555654	0.1920624	NA	NA	PAG1
RPS6KA3	2.039452923	0.748799368	0.1132952	NA	NA	RPS6KA3
FAM84A	1.882711005	0.743572731	0.1920624	NA	FAM84A	FAM84A
SLC44A5	1.508018492	0.740117703	0.8841996	NA	NA	SLC44A5
ARHGAP15	2.095321106	0.736425741	0.1132952	NA	ARHGAP15	ARHGAP15
STIM2	1.601732135	0.736379264	0.8841996	NA	NA	STIM2
PHF17	1.940661664	0.735165164	0.1920624	NA	NA	PHF17
NEDD4	2.135901336	0.73156625	0.1132952	NA	NA	NEDD4
CDCA7	2.32970259	0.730819321	0	NA	NA	CDCA7
ETV1	1.869400066	0.729205933	0.1920624	NA	NA	ETV1
C18orf1	2.627212485	0.726582856	0	NA	NA	C18orf1

BCL2	1.626209371	0.724973607	0.8841996	NA	NA	BCL2
OMG	2.048961701	0.722212659	0.1132952	NA	NA	OMG
COMMD2	2.67383655	0.720154885	0	NA	NA	COMMD2
RFC3	2.091418981	0.715670472	0.1132952	NA	NA	RFC3
FOXP1	2.573537464	0.714898217	0	NA	FOXP1	FOXP1
FBXL17	1.890691069	0.714815869	0.1920624	NA	NA	FBXL17
MGST1	1.860475221	0.701691591	0.1920624	NA	NA	MGST1
SEZ6L	2.383782992	0.700835855	0	NA	NA	SEZ6L
VRK2	1.671637439	0.689973897	0.5692172	NA	VRK2	VRK2
ANK2	1.980579316	0.689171439	0.1920624	NA	NA	ANK2
MAN2A1	1.735773326	0.688175588	0.5692172	NA	NA	MAN2A1
GPAM	2.140470994	0.686996974	0.1132952	NA	GPAM	GPAM
MOCS2	2.692411446	0.681645694	0	NA	MOCS2	MOCS2
PAPPA2	3.35479031	0.678214016	0	NA	NA	PAPPA2
CAMK4	2.598144006	0.673869172	0	NA	NA	CAMK4
PDGFD	2.180255964	0.672588258	0.1132952	NA	NA	PDGFD
UFM1	2.580881702	0.671223127	0	NA	NA	UFM1
FRZB	2.87648549	0.666397665	0	NA	NA	FRZB
SATB2	2.013867357	0.665230402	0.1132952	NA	NA	SATB2
PDZRN3	2.399581709	0.662704991	0	NA	PDZRN3	PDZRN3
NCOA2	2.324436097	0.655431563	0	NA	NA	NCOA2
KCNJ2	1.894681369	0.651676841	0.1920624	NA	NA	KCNJ2
DACT1	3.761208821	0.651434098	0	NA	NA	DACT1
PTDSS1	3.51016526	0.65010501	0	NA	NA	PTDSS1
CPNE4	2.235536593	0.649730283	0	NA	NA	CPNE4
B3GALT2	3.186066515	0.643552774	0	NA	NA	B3GALT2
NTNG1	2.56015907	0.6424647	0	NA	NTNG1	NTNG1
SYNPR	2.211211773	0.6402151	0	NA	SYNPR	SYNPR
LRRN3	3.792313283	0.62994277	0	NA	NA	LRRN3
PLEKHH2	2.707449053	0.600841423	0	NA	NA	PLEKHH2
ZNF238	2.839086702	0.596913	0	NA	ZNF238	ZNF238
ANTXR2	3.917695399	0.583697192	0	NA	ANTXR2	ANTXR2
TNFAIP8	2.545221104	0.577656874	0	NA	NA	TNFAIP8
MET	3.694266312	0.562007528	0	NA	NA	MET
MID1	3.336562507	0.560042328	0	NA	MID1	MID1
NR3C1	3.988644401	0.55590161	0	NA	NA	NR3C1
INHBA	3.850344657	0.535772384	0	NA	NA	INHBA
KCNT2	2.308815908	0.535764159	0	NA	NA	KCNT2
LDLRAD3	4.033439074	0.510085712	0	NA	NA	LDLRAD3
GRB14	2.572872656	0.493036094	0	NA	NA	GRB14
GPR22	3.324830791	0.478356979	0	NA	NA	GPR22
PLCXD3	4.26694021	0.476284155	0	NA	NA	PLCXD3
RSPO2	4.472734211	0.456294973	0	NA	NA	RSPO2
MPP6	2.827116918	0.455774816	0	NA	NA	MPP6
DGKI	2.8821568	0.445093854	0	NA	NA	DGKI
ST6GAL2	4.531853789	0.443982207	0	NA	NA	ST6GAL2
LGI1	5.01261104	0.4257431	0	NA	NA	LGI1
PROK2	3.992329864	0.403362522	0	NA	NA	PROK2
DACH2	5.39837332	0.393896261	0	NA	NA	DACH2
AKT3	5.224519542	0.382303266	0	NA	NA	AKT3
TGFB2	5.287867211	0.380547566	0	NA	NA	TGFB2
TLE1	9.233379941	0.333319215	0	NA	NA	TLE1
NR4A2	5.372879604	0.310340327	0	NA	NR4A2	NR4A2
THBS1	5.220709261	0.278367583	0	NA	NA	THBS1
NPY	10.48770135	0.152609434	0	NA	NA	NPY
PCSK2	11.20597583	0.125898457	0	NA	NA	PCSK2

maCNS only PFO vs PT q<0.01

SYMBOL	PFOvsPT.d	PFOvsPT.FC	PFOvsPT.q	haCNS	caCNS	maCNS
FSTL5	-9.13405901	5.144152677	0	NA	NA	FSTL5
CBLN1	-7.70411465	4.807194815	0	NA	NA	CBLN1
BDNF	-6.02848348	4.730594258	0	NA	NA	BDNF
ZNF423	-6.90224806	4.597465111	0	NA	NA	ZNF423
PTX3	-5.00633748	2.692349177	0	NA	NA	PTX3
ST6GALNAC5	-6.09921617	2.366577756	0	NA	NA	ST6GALNAC5
MC4R	-2.74483075	2.077390683	0	NA	NA	MC4R
CYP26A1	-2.91046367	2.070475578	0	NA	NA	CYP26A1
LRRC3B	-4.8019253	1.974090641	0	NA	NA	LRRC3B
PLK2	-4.18086128	1.840616327	0	NA	NA	PLK2
KCNQ5	-3.4225801	1.815481633	0	NA	NA	KCNQ5
NEBL	-3.95962297	1.814775522	0	NA	NA	NEBL
RPH3A	-1.83743736	1.750384294	0.1920624	NA	NA	RPH3A
BAMBI	-2.54868324	1.746195742	0	NA	NA	BAMBI
TNFAIP3	-1.74599811	1.72654034	0.5692172	NA	NA	TNFAIP3
NRIP1	-3.2856362	1.715123759	0	NA	NA	NRIP1
CDH12	-3.09316342	1.701936008	0	NA	NA	CDH12
CXXC4	-3.9002226	1.683449791	0	NA	NA	CXXC4
PHLDA1	-4.05016913	1.659226237	0	NA	NA	PHLDA1
KIT	-3.17697957	1.637540014	0	NA	NA	KIT
UST	-2.35119547	1.632375331	0	NA	NA	UST
SOCS2	-2.38288631	1.626189776	0	NA	NA	SOCS2
ADCYAP1	-3.09707721	1.615928824	0	NA	NA	ADCYAP1
THRB	-2.1344228	1.601725202	0.1132952	NA	NA	THRB
FAM5B	-2.75243221	1.600873707	0	NA	NA	FAM5B
TSPAN6	-1.97172607	1.546995067	0.1920624	NA	NA	TSPAN6
QPCT	-1.69896847	1.522678029	0.5692172	NA	NA	QPCT
CTGF	-1.7315289	1.501443714	0.5692172	NA	NA	CTGF
ITGA9	-2.81551544	1.488179049	0	NA	NA	ITGA9
C7orf36	-2.15755103	1.485669325	0.1132952	NA	NA	C7orf36
ADAMTS3	-2.41327432	1.484830156	0	NA	NA	ADAMTS3
COL12A1	-2.56005564	1.458176067	0	NA	NA	COL12A1
GAS1	-2.82070171	1.439256816	0	NA	NA	GAS1
RRAS2	-2.21380586	1.436160734	0	NA	NA	RRAS2
RFK	-3.07661835	1.428068941	0	NA	NA	RFK
H2AFY2	-2.90931696	1.401067056	0	NA	NA	H2AFY2
PLCB4	-1.9883246	1.386326283	0.1920624	NA	NA	PLCB4
CHN1	-2.31384252	1.384545514	0	NA	NA	CHN1
DIO2	-1.68458127	1.345644949	0.5692172	NA	NA	DIO2
APBB2	-1.93694713	1.337819274	0.1920624	NA	NA	APBB2
SH3D19	-2.18016659	1.337417312	0	NA	NA	SH3D19
ZADH2	-1.93886437	1.336059328	0.1920624	NA	NA	ZADH2
QDPR	-2.19795663	1.330727363	0	NA	NA	QDPR
DPF3	-1.99485721	1.322803272	0.1132952	NA	NA	DPF3
PRKD1	-1.92726666	1.322584174	0.1920624	NA	NA	PRKD1
GRM5	-2.08737967	1.322098237	0.1132952	NA	NA	GRM5
ADRA2A	-2.2026569	1.32088762	0	NA	NA	ADRA2A

NINJ2	-1.80627593	1.320586093	0.5692172	NA	NA	NINJ2
KCNK2	-1.6590451	1.315874513	0.5692172	NA	NA	KCNK2
CRADD	-1.62279567	1.302153136	0.8841996	NA	NA	CRADD
RUNX2	-1.6858028	1.284678259	0.5692172	NA	NA	RUNX2
RSPO3	-1.53076447	1.282867266	0.8841996	NA	NA	RSPO3
E2F3	-1.74676342	1.278940873	0.5692172	NA	NA	E2F3
MYO1B	-1.65357423	1.269053424	0.8841996	NA	NA	MYO1B
MAOA	-1.72301752	1.264390327	0.5692172	NA	NA	MAOA
CACNA1D	-1.80598597	1.264322789	0.5692172	NA	NA	CACNA1D
WWP2	-1.66442974	1.262947586	0.5692172	NA	NA	WWP2
MYC	-1.84922178	1.260900838	0.1920624	NA	NA	MYC
CA8	-1.72634986	1.239795344	0.5692172	NA	NA	CA8
ZNF706	-1.84899918	1.22405684	0.1920624	NA	NA	ZNF706
ZNF395	-1.59880805	1.21514445	0.8841996	NA	NA	ZNF395
KCNF1	-1.62789501	1.214471747	0.8841996	NA	NA	KCNF1
ARL4C	-1.70895532	1.213335346	0.5692172	NA	NA	ARL4C
DGKH	1.670851083	0.817662921	0.5692172	NA	NA	DGKH
RHOBTB1	1.520346451	0.806511173	0.8841996	NA	NA	RHOBTB1
ITGA4	1.554255687	0.803971291	0.8841996	NA	NA	ITGA4
CACNB2	1.758489199	0.803207464	0.5692172	NA	NA	CACNB2
KBTBD8	1.572020284	0.799328082	0.8841996	NA	NA	KBTBD8
TLN2	1.634174676	0.797965962	0.8841996	NA	NA	TLN2
GRM3	1.796391511	0.795173215	0.5692172	NA	NA	GRM3
AMIGO2	1.538916893	0.790467469	0.8841996	NA	NA	AMIGO2
GPD2	1.594044077	0.789623285	0.8841996	NA	NA	GPD2
DNAJC1	1.883652286	0.787195104	0.1920624	NA	NA	DNAJC1
AP1S2	2.177174512	0.781873197	0.1132952	NA	NA	AP1S2
AMMECR1	1.789781392	0.779275819	0.5692172	NA	NA	AMMECR1
NR4A3	2.057562608	0.775546131	0.1132952	NA	NA	NR4A3
BRAF	1.606726902	0.773847676	0.8841996	NA	NA	BRAF
IGSF21	1.819825649	0.764397197	0.1920624	NA	NA	IGSF21
TRIM44	1.980988715	0.763210785	0.1920624	NA	NA	TRIM44
HCN1	1.876834754	0.759545131	0.1920624	NA	NA	HCN1
DPYD	1.91158004	0.757162176	0.1920624	NA	NA	DPYD
PTPRZ1	2.110731279	0.756312617	0.1132952	NA	NA	PTPRZ1
PAG1	1.992347617	0.753555654	0.1920624	NA	NA	PAG1
RPS6KA3	2.039452923	0.748799368	0.1132952	NA	NA	RPS6KA3
SLC44A5	1.508018492	0.740117703	0.8841996	NA	NA	SLC44A5
STIM2	1.601732135	0.736379264	0.8841996	NA	NA	STIM2
PHF17	1.940661664	0.735165164	0.1920624	NA	NA	PHF17
NEDD4	2.135901336	0.73156625	0.1132952	NA	NA	NEDD4
CDCA7	2.32970259	0.730819321	0	NA	NA	CDCA7
ETV1	1.869400066	0.729205933	0.1920624	NA	NA	ETV1
C18orf1	2.627212485	0.726582856	0	NA	NA	C18orf1
BCL2	1.626209371	0.724973607	0.8841996	NA	NA	BCL2
OMG	2.048961701	0.722212659	0.1132952	NA	NA	OMG
COMMD2	2.67383655	0.720154885	0	NA	NA	COMMD2
RFC3	2.091418981	0.715670472	0.1132952	NA	NA	RFC3
FBXL17	1.890691069	0.714815869	0.1920624	NA	NA	FBXL17
MGST1	1.860475221	0.701691591	0.1920624	NA	NA	MGST1
SEZ6L	2.383782992	0.700835855	0	NA	NA	SEZ6L
ANK2	1.980579316	0.689171439	0.1920624	NA	NA	ANK2
MAN2A1	1.735773326	0.688175588	0.5692172	NA	NA	MAN2A1
PAPPA2	3.35479031	0.678214016	0	NA	NA	PAPPA2
CAMK4	2.598144006	0.673869172	0	NA	NA	CAMK4
PDGFD	2.180255964	0.672588258	0.1132952	NA	NA	PDGFD
UFM1	2.580881702	0.671223127	0	NA	NA	UFM1

FRZB	2.87648549	0.666397665	0	NA	NA	FRZB
SATB2	2.013867357	0.665230402	0.1132952	NA	NA	SATB2
NCOA2	2.324436097	0.655431563	0	NA	NA	NCOA2
KCNJ2	1.894681369	0.651676841	0.1920624	NA	NA	KCNJ2
DACT1	3.761208821	0.651434098	0	NA	NA	DACT1
PTDSS1	3.51016526	0.65010501	0	NA	NA	PTDSS1
CPNE4	2.235536593	0.649730283	0	NA	NA	CPNE4
B3GALT2	3.186066515	0.643552774	0	NA	NA	B3GALT2
LRRN3	3.792313283	0.62994277	0	NA	NA	LRRN3
PLEKHH2	2.707449053	0.600841423	0	NA	NA	PLEKHH2
TNFAIP8	2.545221104	0.577656874	0	NA	NA	TNFAIP8
MET	3.694266312	0.562007528	0	NA	NA	MET
NR3C1	3.988644401	0.55590161	0	NA	NA	NR3C1
INHBA	3.850344657	0.535772384	0	NA	NA	INHBA
KCNT2	2.308815908	0.535764159	0	NA	NA	KCNT2
LDLRAD3	4.033439074	0.510085712	0	NA	NA	LDLRAD3
GRB14	2.572872656	0.493036094	0	NA	NA	GRB14
GPR22	3.324830791	0.478356979	0	NA	NA	GPR22
PLCXD3	4.26694021	0.476284155	0	NA	NA	PLCXD3
RSPO2	4.472734211	0.456294973	0	NA	NA	RSPO2
MPP6	2.827116918	0.455774816	0	NA	NA	MPP6
DGKI	2.8821568	0.445093854	0	NA	NA	DGKI
ST6GAL2	4.531853789	0.443982207	0	NA	NA	ST6GAL2
LGI1	5.01261104	0.4257431	0	NA	NA	LGI1
PROK2	3.992329864	0.403362522	0	NA	NA	PROK2
DACH2	5.39837332	0.393896261	0	NA	NA	DACH2
AKT3	5.224519542	0.382303266	0	NA	NA	AKT3
TGFB2	5.287867211	0.380547566	0	NA	NA	TGFB2
TLE1	9.233379941	0.333319215	0	NA	NA	TLE1
THBS1	5.220709261	0.278367583	0	NA	NA	THBS1
NPY	10.48770135	0.152609434	0	NA	NA	NPY
PCSK2	11.20597583	0.125898457	0	NA	NA	PCSK2

haCNS 17vs19 q<0.01

**SYMBOL**	**17vs19.d**	**17vs19.FC**	**17vs19.q**	**haCNS**	**caCNS**	**maCNS**
GJA1	-3.17337837	2.191557093	0	GJA1	GJA1	NA
PDGFRL	-3.50868784	2.13452466	0	PDGFRL	NA	NA
CDKAL1	-6.8380691	1.932350629	0	CDKAL1	NA	CDKAL1
GPC6	-2.9574624	1.70632811	0	GPC6	NA	GPC6
CLSTN2	-2.46368422	1.678856802	0.1540868	CLSTN2	CLSTN2	CLSTN2
PDGFC	-4.43591912	1.580637285	0	PDGFC	PDGFC	NA
EFEMP1	-2.26012455	1.512079005	0.1540868	EFEMP1	EFEMP1	EFEMP1
PLXNA2	-1.97437948	1.490453198	0.520402	PLXNA2	PLXNA2	PLXNA2
MAN1A1	-2.34859792	1.474033488	0.1540868	MAN1A1	MAN1A1	MAN1A1
ANXA2	-1.99979244	1.453295433	0.520402	ANXA2	ANXA2	NA
SDPR	-3.04083896	1.447760103	0	SDPR	SDPR	SDPR
TLE4	-2.22090823	1.420751773	0.520402	TLE4	TLE4	TLE4
SLC16A7	-2.11445294	1.419373195	0.520402	SLC16A7	SLC16A7	SLC16A7
PCSK5	-3.0316199	1.397304819	0	PCSK5	NA	NA
LMO7	-2.86362537	1.393641999	0	LMO7	LMO7	LMO7
MPDZ	-3.80357657	1.388112978	0	MPDZ	MPDZ	MPDZ
LHFPL3	-3.49938347	1.365308367	0	LHFPL3	NA	NA
NAV2	-4.33146512	1.322265666	0	NAV2	NA	NAV2
PKN2	-2.50864858	1.301000047	0.1540868	PKN2	PKN2	PKN2

CDH5	-2.99957549	1.29949249	0	CDH5	NA	NA
TXNL1	-1.95194037	1.295710276	0.520402	TXNL1	NA	TXNL1
PREP	-1.98403198	1.275789004	0.520402	PREP	NA	PREP
TWISTNB	-1.95938135	1.238987049	0.520402	TWISTNB	NA	NA
HS3ST4	-1.88909404	1.237218832	0.520402	HS3ST4	HS3ST4	HS3ST4
MAP3K7	-2.31255986	1.236699116	0.1540868	MAP3K7	MAP3K7	MAP3K7
PSAT1	-2.31918136	1.227550292	0.1540868	PSAT1	PSAT1	PSAT1
CD47	-1.97752486	1.212944821	0.520402	CD47	NA	CD47
ADPGK	-2.07538358	1.211264074	0.520402	ADPGK	NA	ADPGK
MTERF	-2.19888817	1.205433619	0.520402	MTERF	NA	MTERF
HCCS	-2.28285887	1.20536856	0.1540868	HCCS	NA	NA
VRK1	-2.02209067	1.195584346	0.520402	VRK1	NA	VRK1
ZFP90	-1.94156111	1.17520105	0.520402	ZFP90	NA	NA
P2RY10	2.172263829	0.859254145	0.520402	P2RY10	NA	NA
PAX1	2.365958635	0.824003886	0.520402	PAX1	NA	NA
TM7SF4	2.620467402	0.771725549	0.1540868	TM7SF4	NA	NA
PNOC	2.573718838	0.755997961	0.1540868	PNOC	NA	NA
PCDH8	2.407177097	0.715150483	0.520402	PCDH8	NA	PCDH8
SOX21	3.53743389	0.651630457	0	SOX21	NA	SOX21

haCNS-caCNS 17vs19 q<0.01

**SYMBOL**	**17vs19.d**	**17vs19.FC**	**17vs19.q**	**haCNS**	**caCNS**	**maCNS**
PDGFRL	-3.50868784	2.13452466	0	PDGFRL	NA	NA
CDKAL1	-6.8380691	1.932350629	0	CDKAL1	NA	CDKAL1
GPC6	-2.9574624	1.70632811	0	GPC6	NA	GPC6
PCSK5	-3.0316199	1.397304819	0	PCSK5	NA	NA
LHFPL3	-3.49938347	1.365308367	0	LHFPL3	NA	NA
NAV2	-4.33146512	1.322265666	0	NAV2	NA	NAV2
CDH5	-2.99957549	1.29949249	0	CDH5	NA	NA
TXNL1	-1.95194037	1.295710276	0.520402	TXNL1	NA	TXNL1
PREP	-1.98403198	1.275789004	0.520402	PREP	NA	PREP
TWISTNB	-1.95938135	1.238987049	0.520402	TWISTNB	NA	NA
CD47	-1.97752486	1.212944821	0.520402	CD47	NA	CD47
ADPGK	-2.07538358	1.211264074	0.520402	ADPGK	NA	ADPGK
MTERF	-2.19888817	1.205433619	0.520402	MTERF	NA	MTERF
HCCS	-2.28285887	1.20536856	0.1540868	HCCS	NA	NA
VRK1	-2.02209067	1.195584346	0.520402	VRK1	NA	VRK1
ZFP90	-1.94156111	1.17520105	0.520402	ZFP90	NA	NA
P2RY10	2.172263829	0.859254145	0.520402	P2RY10	NA	NA
PAX1	2.365958635	0.824003886	0.520402	PAX1	NA	NA
TM7SF4	2.620467402	0.771725549	0.1540868	TM7SF4	NA	NA
PNOC	2.573718838	0.755997961	0.1540868	PNOC	NA	NA
PCDH8	2.407177097	0.715150483	0.520402	PCDH8	NA	PCDH8
SOX21	3.53743389	0.651630457	0	SOX21	NA	SOX21

haCNS only 17vs19 q<0.01

**SYMBOL**	**17vs19.d**	**17vs19.FC**	**17vs19.q**	**haCNS**	**caCNS**	**maCNS**
PDGFRL	-3.50868784	2.13452466	0	PDGFRL	NA	NA
CDH5	-2.99957549	1.29949249	0	CDH5	NA	NA

LHFPL3	-3.49938347	1.365308367	0	LHFPL3	NA	NA
PCSK5	-3.0316199	1.397304819	0	PCSK5	NA	NA
HCCS	-2.28285887	1.20536856	0.1540868	HCCS	NA	NA
PNOC	2.573718838	0.755997961	0.1540868	PNOC	NA	NA
TM7SF4	2.620467402	0.771725549	0.1540868	TM7SF4	NA	NA
ZFP90	-1.94156111	1.17520105	0.520402	ZFP90	NA	NA
P2RY10	2.172263829	0.859254145	0.520402	P2RY10	NA	NA
PAX1	2.365958635	0.824003886	0.520402	PAX1	NA	NA
TWISTNB	-1.95938135	1.238987049	0.520402	TWISTNB	NA	NA

caCNS 17vs19 q<0.01

SYMBOL	17vs19.d	17vs19.FC	17vs19.q	haCNS	caCNS	maCNS
GJA1	-3.17337837	2.191557093	0	GJA1	GJA1	NA
CLSTN2	-2.46368422	1.678856802	0.1540868	CLSTN2	CLSTN2	CLSTN2
PDGFC	-4.43591912	1.580637285	0	PDGFC	PDGFC	NA
ZFPM2	-4.55035079	1.527789009	0	NA	ZFPM2	ZFPM2
EFEMP1	-2.26012455	1.512079005	0.1540868	EFEMP1	EFEMP1	EFEMP1
EYA1	-3.22782472	1.505148939	0	NA	EYA1	EYA1
PLXNA2	-1.97437948	1.490453198	0.520402	PLXNA2	PLXNA2	PLXNA2
MAN1A1	-2.34859792	1.474033488	0.1540868	MAN1A1	MAN1A1	MAN1A1
ANXA2	-1.99979244	1.453295433	0.520402	ANXA2	ANXA2	NA
SDPR	-3.04083896	1.447760103	0	SDPR	SDPR	SDPR
DAD1	-3.40908343	1.424753181	0	NA	DAD1	NA
TLE4	-2.22090823	1.420751773	0.520402	TLE4	TLE4	TLE4
SLC16A7	-2.11445294	1.419373195	0.520402	SLC16A7	SLC16A7	SLC16A7
LMO7	-2.86362537	1.393641999	0	LMO7	LMO7	LMO7
MPDZ	-3.80357657	1.388112978	0	MPDZ	MPDZ	MPDZ
HHAT	-2.6642639	1.37203725	0.1540868	NA	HHAT	NA
RBMS1	-2.12815335	1.345814478	0.520402	NA	RBMS1	RBMS1
EXT1	-2.79394171	1.335902263	0	NA	EXT1	EXT1
CRBN	-4.49368722	1.319828382	0	NA	CRBN	NA
ADK	-2.13027331	1.319249551	0.520402	NA	ADK	ADK
SLC35B3	-2.29595733	1.312717866	0.1540868	NA	SLC35B3	SLC35B3
PKN2	-2.50864858	1.301000047	0.1540868	PKN2	PKN2	PKN2
KCTD12	-1.90459309	1.283296933	0.520402	NA	KCTD12	NA
CTTNBP2	-2.18193564	1.254580236	0.520402	NA	CTTNBP2	NA
STX18	-2.37313517	1.249432252	0.1540868	NA	STX18	NA
TNIK	-1.92592989	1.240527733	0.520402	NA	TNIK	TNIK
HS3ST4	-1.88909404	1.237218832	0.520402	HS3ST4	HS3ST4	HS3ST4
MAP3K7	-2.31255986	1.236699116	0.1540868	MAP3K7	MAP3K7	MAP3K7
PSAT1	-2.31918136	1.227550292	0.1540868	PSAT1	PSAT1	PSAT1
SHFM1	-2.02317348	1.217277413	0.520402	NA	SHFM1	SHFM1
HIBADH	-1.98143835	1.211610456	0.520402	NA	HIBADH	HIBADH
MCPH1	-2.16212023	1.181917204	0.520402	NA	MCPH1	MCPH1
RBMS3	-2.53824342	1.174347585	0.1540868	NA	RBMS3	RBMS3
WARS2	-1.98825236	1.165129453	0.520402	NA	WARS2	NA
PFKP	2.154326983	0.80612248	0.520402	NA	PFKP	PFKP
CRB1	2.721836542	0.778340406	0.1540868	NA	CRB1	CRB1
FAM19A2	3.079103642	0.755605038	0	NA	FAM19A2	FAM19A2
FLJ32063	3.421259059	0.747785344	0	NA	FLJ32063	FLJ32063
DNAH7	2.419109983	0.707731731	0.520402	NA	DNAH7	DNAH7
GABRB2	2.371876049	0.688095369	0.520402	NA	GABRB2	NA

caCNS-haCNS 17vs19 q<0.01

SYMBOL	17vs19.d	17vs19.FC	17vs19.q	haCNS	caCNS	maCNS
ZFPM2	-4.55035079	1.527789009	0	NA	ZFPM2	ZFPM2
EYA1	-3.22782472	1.505148939	0	NA	EYA1	EYA1
DAD1	-3.40908343	1.424753181	0	NA	DAD1	NA
HHAT	-2.6642639	1.37203725	0.1540868	NA	HHAT	NA
RBMS1	-2.12815335	1.345814478	0.520402	NA	RBMS1	RBMS1
EXT1	-2.79394171	1.335902263	0	NA	EXT1	EXT1
CRBN	-4.49368722	1.319828382	0	NA	CRBN	NA
ADK	-2.13027331	1.319249551	0.520402	NA	ADK	ADK
SLC35B3	-2.29595733	1.312717866	0.1540868	NA	SLC35B3	SLC35B3
KCTD12	-1.90459309	1.283296933	0.520402	NA	KCTD12	NA
CTTNBP2	-2.18193564	1.254580236	0.520402	NA	CTTNBP2	NA
STX18	-2.37313517	1.249432252	0.1540868	NA	STX18	NA
TNIK	-1.92592989	1.240527733	0.520402	NA	TNIK	TNIK
SHFM1	-2.02317348	1.217277413	0.520402	NA	SHFM1	SHFM1
HIBADH	-1.98143835	1.211610456	0.520402	NA	HIBADH	HIBADH
MCPH1	-2.16212023	1.181917204	0.520402	NA	MCPH1	MCPH1
RBMS3	-2.53824342	1.174347585	0.1540868	NA	RBMS3	RBMS3
WARS2	-1.98825236	1.165129453	0.520402	NA	WARS2	NA
PFKP	2.154326983	0.80612248	0.520402	NA	PFKP	PFKP
CRB1	2.721836542	0.778340406	0.1540868	NA	CRB1	CRB1
FAM19A2	3.079103642	0.755605038	0	NA	FAM19A2	FAM19A2
FLJ32063	3.421259059	0.747785344	0	NA	FLJ32063	FLJ32063
DNAH7	2.419109983	0.707731731	0.520402	NA	DNAH7	DNAH7
GABRB2	2.371876049	0.688095369	0.520402	NA	GABRB2	NA

caCNS only 17vs19 q<0.01

SYMBOL	17vs19.d	17vs19.FC	17vs19.q	haCNS	caCNS	maCNS
DAD1	-3.40908343	1.424753181	0	NA	DAD1	NA
CRBN	-4.49368722	1.319828382	0	NA	CRBN	NA
HHAT	-2.6642639	1.37203725	0.1540868	NA	HHAT	NA
STX18	-2.37313517	1.249432252	0.1540868	NA	STX18	NA
GABRB2	2.371876049	0.688095369	0.520402	NA	GABRB2	NA
KCTD12	-1.90459309	1.283296933	0.520402	NA	KCTD12	NA
WARS2	-1.98825236	1.165129453	0.520402	NA	WARS2	NA
CTTNBP2	-2.18193564	1.254580236	0.520402	NA	CTTNBP2	NA

maCNS 17vs19 q<0.01

SYMBOL	17vs19.d	17vs19.FC	17vs19.q	haCNS	caCNS	maCNS
FZD3	-4.97298279	2.702230523	0	NA	NA	FZD3
CDKAL1	-6.8380691	1.932350629	0	CDKAL1	NA	CDKAL1
GRIN2B	-2.78934944	1.864057191	0	NA	NA	GRIN2B

RSRC1	-3.56506296	1.713336184	0	NA	NA	RSRC1
GPC6	-2.9574624	1.70632811	0	GPC6	NA	GPC6
DKK1	-3.65002751	1.683887969	0	NA	NA	DKK1
CLSTN2	-2.46368422	1.678856802	0.1540868	CLSTN2	CLSTN2	CLSTN2
DOCK7	-5.76606227	1.628981586	0	NA	NA	DOCK7
ZNF644	-2.5407901	1.554425864	0.1540868	NA	NA	ZNF644
DKK2	-2.79721009	1.543479741	0	NA	NA	DKK2
COL1A2	-2.52470031	1.537572268	0.1540868	NA	NA	COL1A2
TBX15	-3.22463323	1.533015644	0	NA	NA	TBX15
ZFPM2	-4.55035079	1.527789009	0	NA	ZFPM2	ZFPM2
EFEMP1	-2.26012455	1.512079005	0.1540868	EFEMP1	EFEMP1	EFEMP1
EYA1	-3.22782472	1.505148939	0	NA	EYA1	EYA1
BBX	-2.72541303	1.503352785	0	NA	NA	BBX
PLXNA2	-1.97437948	1.490453198	0.520402	PLXNA2	PLXNA2	PLXNA2
MAN1A1	-2.34859792	1.474033488	0.1540868	MAN1A1	MAN1A1	MAN1A1
SDPR	-3.04083896	1.447760103	0	SDPR	SDPR	SDPR
TOP1	-2.58635713	1.421338755	0.1540868	NA	NA	TOP1
TLE4	-2.22090823	1.420751773	0.520402	TLE4	TLE4	TLE4
SLC16A7	-2.11445294	1.419373195	0.520402	SLC16A7	SLC16A7	SLC16A7
PEX7	-2.83204478	1.415530499	0	NA	NA	PEX7
LMO7	-2.86362537	1.393641999	0	LMO7	LMO7	LMO7
BCL11B	-3.06436444	1.391389965	0	NA	NA	BCL11B
CBX5	-2.34906748	1.389545675	0.1540868	NA	NA	CBX5
MPDZ	-3.80357657	1.388112978	0	MPDZ	MPDZ	MPDZ
C20orf94	-2.87595556	1.387615802	0	NA	NA	C20orf94
TWIST1	-2.17847544	1.387156152	0.520402	NA	NA	TWIST1
C14orf101	-3.32291015	1.386521163	0	NA	NA	C14orf101
SYT6	-2.1545715	1.3776413	0.520402	NA	NA	SYT6
ZIC1	-2.0479031	1.373878606	0.520402	NA	NA	ZIC1
ALDH1A2	-2.91445951	1.370079363	0	NA	NA	ALDH1A2
GLCE	-3.29791405	1.361077222	0	NA	NA	GLCE
ZNF673	-2.82951419	1.350680326	0	NA	NA	ZNF673
FBN1	-2.22232971	1.350007951	0.520402	NA	NA	FBN1
RBMS1	-2.12815335	1.345814478	0.520402	NA	RBMS1	RBMS1
EIF2C3	-2.04353922	1.342983542	0.520402	NA	NA	EIF2C3
EXT1	-2.79394171	1.335902263	0	NA	EXT1	EXT1
C14orf118	-2.35311208	1.330164075	0.1540868	NA	NA	C14orf118
ZNF281	-3.44029224	1.328545073	0	NA	NA	ZNF281
NAV2	-4.33146512	1.322265666	0	NAV2	NA	NAV2
ADAMTS5	-2.55787551	1.321249272	0.1540868	NA	NA	ADAMTS5
ADK	-2.13027331	1.319249551	0.520402	NA	ADK	ADK
SLC35B3	-2.29595733	1.312717866	0.1540868	NA	SLC35B3	SLC35B3
KLF5	-3.32459292	1.312042574	0	NA	NA	KLF5
FRMD6	-2.21758141	1.302538678	0.520402	NA	NA	FRMD6
PKN2	-2.50864858	1.301000047	0.1540868	PKN2	PKN2	PKN2
CASC3	-2.66470508	1.300765107	0.1540868	NA	NA	CASC3
FZD10	-2.03362973	1.299214783	0.520402	NA	NA	FZD10
TIMP3	-2.08494387	1.296640498	0.520402	NA	NA	TIMP3
TFAM	-2.21244877	1.295833643	0.520402	NA	NA	TFAM
TXNL1	-1.95194037	1.295710276	0.520402	TXNL1	NA	TXNL1
BOC	-2.80684746	1.276563183	0	NA	NA	BOC
PREP	-1.98403198	1.275789004	0.520402	PREP	NA	PREP
CCDC28A	-2.13096962	1.264175159	0.520402	NA	NA	CCDC28A
MMP16	-1.99497007	1.259489116	0.520402	NA	NA	MMP16
CLCN3	-2.07964964	1.255031196	0.520402	NA	NA	CLCN3
PEX5L	-2.16408121	1.245397069	0.520402	NA	NA	PEX5L
TNIK	-1.92592989	1.240527733	0.520402	NA	TNIK	TNIK

ZNF326	-2.32096285	1.239165076	0.1540868	NA	NA	ZNF326
HS3ST4	-1.88909404	1.237218832	0.520402	HS3ST4	HS3ST4	HS3ST4
MAP3K7	-2.31255986	1.236699116	0.1540868	MAP3K7	MAP3K7	MAP3K7
PSAT1	-2.31918136	1.227550292	0.1540868	PSAT1	PSAT1	PSAT1
SS18	-1.96813576	1.226954949	0.520402	NA	NA	SS18
AKAP12	-2.27860814	1.224420608	0.1540868	NA	NA	AKAP12
SAMD9	-1.96276899	1.223845755	0.520402	NA	NA	SAMD9
SUMF1	-2.10382774	1.219291023	0.520402	NA	NA	SUMF1
MPPED1	-2.01198389	1.217516571	0.520402	NA	NA	MPPED1
SHFM1	-2.02317348	1.217277413	0.520402	NA	SHFM1	SHFM1
C3orf17	-1.91280258	1.213563178	0.520402	NA	NA	C3orf17
CD47	-1.97752486	1.212944821	0.520402	CD47	NA	CD47
HIBADH	-1.98143835	1.211610456	0.520402	NA	HIBADH	HIBADH
ADPGK	-2.07538358	1.211264074	0.520402	ADPGK	NA	ADPGK
MTERF	-2.19888817	1.205433619	0.520402	MTERF	NA	MTERF
VRK1	-2.02209067	1.195584346	0.520402	VRK1	NA	VRK1
MAML3	-2.2695581	1.182789139	0.1540868	NA	NA	MAML3
AHI1	-1.9220662	1.182095369	0.520402	NA	NA	AHI1
MCPH1	-2.16212023	1.181917204	0.520402	NA	MCPH1	MCPH1
RBMS3	-2.53824342	1.174347585	0.1540868	NA	RBMS3	RBMS3
TSPAN3	-1.90848538	1.122098293	0.520402	NA	NA	TSPAN3
SLC39A10	2.208794368	0.836722995	0.520402	NA	NA	SLC39A10
PFKP	2.154326983	0.80612248	0.520402	NA	PFKP	PFKP
CACNG2	2.453906731	0.790183967	0.520402	NA	NA	CACNG2
PITRM1	2.777443066	0.789302367	0.1540868	NA	NA	PITRM1
CHEK2	2.114178998	0.785876281	0.520402	NA	NA	CHEK2
CRB1	2.721836542	0.778340406	0.1540868	NA	CRB1	CRB1
PON1	2.083377541	0.777106183	0.520402	NA	NA	PON1
LMX1A	2.455077253	0.771723071	0.520402	NA	NA	LMX1A
FLJ46321	2.449140041	0.767112038	0.520402	NA	NA	FLJ46321
TFDP3	2.230751504	0.763275533	0.520402	NA	NA	TFDP3
RGS22	2.172300014	0.760066715	0.520402	NA	NA	RGS22
FAM19A2	3.079103642	0.755605038	0	NA	FAM19A2	FAM19A2
FLJ32063	3.421259059	0.747785344	0	NA	FLJ32063	FLJ32063
ATIC	2.099638565	0.745570268	0.520402	NA	NA	ATIC
WIT1	2.083036558	0.740352502	0.520402	NA	NA	WIT1
HSD11B2	2.292516661	0.72482175	0.520402	NA	NA	HSD11B2
MAS1	2.096200531	0.723824276	0.520402	NA	NA	MAS1
HDDC2	2.513864005	0.718404422	0.1540868	NA	NA	HDDC2
PCDH8	2.407177097	0.715150483	0.520402	PCDH8	NA	PCDH8
DNAH7	2.419109983	0.707731731	0.520402	NA	DNAH7	DNAH7
SOX21	3.53743389	0.651630457	0	SOX21	NA	SOX21
KCTD16	2.354394955	0.60252427	0.520402	NA	NA	KCTD16
BMPR1B	2.606890498	0.576279577	0.1540868	NA	NA	BMPR1B
TNNI3K	4.763428484	0.575831723	0	NA	NA	TNNI3K

maCNS-haCNS 17vs19 q<0.01

SYMBOL	17vs19.d	17vs19.FC	17vs19.q	haCNS	caCNS	maCNS
FZD3	-4.97298279	2.702230523	0	NA	NA	FZD3
GRIN2B	-2.78934944	1.864057191	0	NA	NA	GRIN2B
RSRC1	-3.56506296	1.713336184	0	NA	NA	RSRC1
DKK1	-3.65002751	1.683887969	0	NA	NA	DKK1
DOCK7	-5.76606227	1.628981586	0	NA	NA	DOCK7

ZNF644	-2.5407901	1.554425864	0.1540868	NA	NA	ZNF644
DKK2	-2.79721009	1.543479741	0	NA	NA	DKK2
COL1A2	-2.52470031	1.537572268	0.1540868	NA	NA	COL1A2
TBX15	-3.22463323	1.533015644	0	NA	NA	TBX15
ZFPM2	-4.55035079	1.527789009	0	NA	ZFPM2	ZFPM2
EYA1	-3.22782472	1.505148939	0	NA	EYA1	EYA1
BBX	-2.72541303	1.503352785	0	NA	NA	BBX
TOP1	-2.58635713	1.421338755	0.1540868	NA	NA	TOP1
PEX7	-2.83204478	1.415530499	0	NA	NA	PEX7
BCL11B	-3.06436444	1.391389965	0	NA	NA	BCL11B
CBX5	-2.34906748	1.389545675	0.1540868	NA	NA	CBX5
C20orf94	-2.87595556	1.387615802	0	NA	NA	C20orf94
TWIST1	-2.17847544	1.387156152	0.520402	NA	NA	TWIST1
C14orf101	-3.32291015	1.386521163	0	NA	NA	C14orf101
SYT6	-2.1545715	1.3776413	0.520402	NA	NA	SYT6
ZIC1	-2.0479031	1.373878606	0.520402	NA	NA	ZIC1
ALDH1A2	-2.91445951	1.370079363	0	NA	NA	ALDH1A2
GLCE	-3.29791405	1.361077222	0	NA	NA	GLCE
ZNF673	-2.82951419	1.350680326	0	NA	NA	ZNF673
FBN1	-2.22232971	1.350007951	0.520402	NA	NA	FBN1
RBMS1	-2.12815335	1.345814478	0.520402	NA	RBMS1	RBMS1
EIF2C3	-2.04353922	1.342983542	0.520402	NA	NA	EIF2C3
EXT1	-2.79394171	1.335902263	0	NA	EXT1	EXT1
C14orf118	-2.35311208	1.330164075	0.1540868	NA	NA	C14orf118
ZNF281	-3.44029224	1.328545073	0	NA	NA	ZNF281
ADAMTS5	-2.55787551	1.321249272	0.1540868	NA	NA	ADAMTS5
ADK	-2.13027331	1.319249551	0.520402	NA	ADK	ADK
SLC35B3	-2.29595733	1.312717866	0.1540868	NA	SLC35B3	SLC35B3
KLF5	-3.32459292	1.312042574	0	NA	NA	KLF5
FRMD6	-2.21758141	1.302538678	0.520402	NA	NA	FRMD6
CASC3	-2.66470508	1.300765107	0.1540868	NA	NA	CASC3
FZD10	-2.03362973	1.299214783	0.520402	NA	NA	FZD10
TIMP3	-2.08494387	1.296640498	0.520402	NA	NA	TIMP3
TFAM	-2.21244877	1.295833643	0.520402	NA	NA	TFAM
BOC	-2.80684746	1.276563183	0	NA	NA	BOC
CCDC28A	-2.13096962	1.264175159	0.520402	NA	NA	CCDC28A
MMP16	-1.99497007	1.259489116	0.520402	NA	NA	MMP16
CLCN3	-2.07964964	1.255031196	0.520402	NA	NA	CLCN3
PEX5L	-2.16408121	1.245397069	0.520402	NA	NA	PEX5L
TNIK	-1.92592989	1.240527733	0.520402	NA	TNIK	TNIK
ZNF326	-2.32096285	1.239165076	0.1540868	NA	NA	ZNF326
SS18	-1.96813576	1.226954949	0.520402	NA	NA	SS18
AKAP12	-2.27860814	1.224420608	0.1540868	NA	NA	AKAP12
SAMD9	-1.96276899	1.223845755	0.520402	NA	NA	SAMD9
SUMF1	-2.10382774	1.219291023	0.520402	NA	NA	SUMF1
MPPED1	-2.01198389	1.217516571	0.520402	NA	NA	MPPED1
SHFM1	-2.02317348	1.217277413	0.520402	NA	SHFM1	SHFM1
C3orf17	-1.91280258	1.213563178	0.520402	NA	NA	C3orf17
HIBADH	-1.98143835	1.211610456	0.520402	NA	HIBADH	HIBADH
MAML3	-2.2695581	1.182789139	0.1540868	NA	NA	MAML3
AHI1	-1.9220662	1.182095369	0.520402	NA	NA	AHI1
MCPH1	-2.16212023	1.181917204	0.520402	NA	MCPH1	MCPH1
RBMS3	-2.53824342	1.174347585	0.1540868	NA	RBMS3	RBMS3
TSPAN3	-1.90848538	1.122098293	0.520402	NA	NA	TSPAN3
SLC39A10	2.208794368	0.836722995	0.520402	NA	NA	SLC39A10
PFKP	2.154326983	0.80612248	0.520402	NA	PFKP	PFKP
CACNG2	2.453906731	0.790183967	0.520402	NA	NA	CACNG2

PITRM1	2.777443066	0.789302367	0.1540868	NA	NA	PITRM1
CHEK2	2.114178998	0.785876281	0.520402	NA	NA	CHEK2
CRB1	2.721836542	0.778340406	0.1540868	NA	CRB1	CRB1
PON1	2.083377541	0.777106183	0.520402	NA	NA	PON1
LMX1A	2.455077253	0.771723071	0.520402	NA	NA	LMX1A
FLJ46321	2.449140041	0.767112038	0.520402	NA	NA	FLJ46321
TFDP3	2.230751504	0.763275533	0.520402	NA	NA	TFDP3
RGS22	2.172300014	0.760066715	0.520402	NA	NA	RGS22
FAM19A2	3.079103642	0.755605038	0	NA	FAM19A2	FAM19A2
FLJ32063	3.421259059	0.747785344	0	NA	FLJ32063	FLJ32063
ATIC	2.099638565	0.745570268	0.520402	NA	NA	ATIC
WIT1	2.083036558	0.740352502	0.520402	NA	NA	WIT1
HSD11B2	2.292516661	0.72482175	0.520402	NA	NA	HSD11B2
MAS1	2.096200531	0.723824276	0.520402	NA	NA	MAS1
HDDC2	2.513864005	0.718404422	0.1540868	NA	NA	HDDC2
DNAH7	2.419109983	0.707731731	0.520402	NA	DNAH7	DNAH7
KCTD16	2.354394955	0.60252427	0.520402	NA	NA	KCTD16
BMPR1B	2.606890498	0.576279577	0.1540868	NA	NA	BMPR1B
TNNI3K	4.763428484	0.575831723	0	NA	NA	TNNI3K

maCNS only 17vs19 q<0.01

SYMBOL	17vs19.d	17vs19.FC	17vs19.q	haCNS	caCNS	maCNS
FZD3	-4.97298279	2.702230523	0	NA	NA	FZD3
GRIN2B	-2.78934944	1.864057191	0	NA	NA	GRIN2B
RSRC1	-3.56506296	1.713336184	0	NA	NA	RSRC1
DKK1	-3.65002751	1.683887969	0	NA	NA	DKK1
DOCK7	-5.76606227	1.628981586	0	NA	NA	DOCK7
ZNF644	-2.5407901	1.554425864	0.1540868	NA	NA	ZNF644
DKK2	-2.79721009	1.543479741	0	NA	NA	DKK2
COL1A2	-2.52470031	1.537572268	0.1540868	NA	NA	COL1A2
TBX15	-3.22463323	1.533015644	0	NA	NA	TBX15
BBX	-2.72541303	1.503352785	0	NA	NA	BBX
TOP1	-2.58635713	1.421338755	0.1540868	NA	NA	TOP1
PEX7	-2.83204478	1.415530499	0	NA	NA	PEX7
BCL11B	-3.06436444	1.391389965	0	NA	NA	BCL11B
CBX5	-2.34906748	1.389545675	0.1540868	NA	NA	CBX5
C20orf94	-2.87595556	1.387615802	0	NA	NA	C20orf94
TWIST1	-2.17847544	1.387156152	0.520402	NA	NA	TWIST1
C14orf101	-3.32291015	1.386521163	0	NA	NA	C14orf101
SYT6	-2.1545715	1.3776413	0.520402	NA	NA	SYT6
ZIC1	-2.0479031	1.373878606	0.520402	NA	NA	ZIC1
ALDH1A2	-2.91445951	1.370079363	0	NA	NA	ALDH1A2
GLCE	-3.29791405	1.361077222	0	NA	NA	GLCE
ZNF673	-2.82951419	1.350680326	0	NA	NA	ZNF673
FBN1	-2.22232971	1.350007951	0.520402	NA	NA	FBN1
EIF2C3	-2.04353922	1.342983542	0.520402	NA	NA	EIF2C3
C14orf118	-2.35311208	1.330164075	0.1540868	NA	NA	C14orf118
ZNF281	-3.44029224	1.328545073	0	NA	NA	ZNF281
ADAMTS5	-2.55787551	1.321249272	0.1540868	NA	NA	ADAMTS5
KLF5	-3.32459292	1.312042574	0	NA	NA	KLF5
FRMD6	-2.21758141	1.302538678	0.520402	NA	NA	FRMD6
CASC3	-2.66470508	1.300765107	0.1540868	NA	NA	CASC3
FZD10	-2.03362973	1.299214783	0.520402	NA	NA	FZD10

TIMP3	-2.08494387	1.296640498	0.520402	NA	NA	TIMP3
TFAM	-2.21244877	1.295833643	0.520402	NA	NA	TFAM
BOC	-2.80684746	1.276563183	0	NA	NA	BOC
CCDC28A	-2.13096962	1.264175159	0.520402	NA	NA	CCDC28A
MMP16	-1.99497007	1.259489116	0.520402	NA	NA	MMP16
CLCN3	-2.07964964	1.255031196	0.520402	NA	NA	CLCN3
PEX5L	-2.16408121	1.245397069	0.520402	NA	NA	PEX5L
ZNF326	-2.32096285	1.239165076	0.1540868	NA	NA	ZNF326
SS18	-1.96813576	1.226954949	0.520402	NA	NA	SS18
AKAP12	-2.27860814	1.224420608	0.1540868	NA	NA	AKAP12
SAMD9	-1.96276899	1.223845755	0.520402	NA	NA	SAMD9
SUMF1	-2.10382774	1.219291023	0.520402	NA	NA	SUMF1
MPPED1	-2.01198389	1.217516571	0.520402	NA	NA	MPPED1
C3orf17	-1.91280258	1.213563178	0.520402	NA	NA	C3orf17
MAML3	-2.2695581	1.182789139	0.1540868	NA	NA	MAML3
AHI1	-1.9220662	1.182095369	0.520402	NA	NA	AHI1
TSPAN3	-1.90848538	1.122098293	0.520402	NA	NA	TSPAN3
SLC39A10	2.208794368	0.836722995	0.520402	NA	NA	SLC39A10
CACNG2	2.453906731	0.790183967	0.520402	NA	NA	CACNG2
PITRM1	2.777443066	0.789302367	0.1540868	NA	NA	PITRM1
CHEK2	2.114178998	0.785876281	0.520402	NA	NA	CHEK2
PON1	2.083377541	0.777106183	0.520402	NA	NA	PON1
LMX1A	2.455077253	0.771723071	0.520402	NA	NA	LMX1A
FLJ46321	2.449140041	0.767112038	0.520402	NA	NA	FLJ46321
TFDP3	2.230751504	0.763275533	0.520402	NA	NA	TFDP3
RGS22	2.172300014	0.760066715	0.520402	NA	NA	RGS22
ATIC	2.099638565	0.745570268	0.520402	NA	NA	ATIC
WIT1	2.083036558	0.740352502	0.520402	NA	NA	WIT1
HSD11B2	2.292516661	0.72482175	0.520402	NA	NA	HSD11B2
MAS1	2.096200531	0.723824276	0.520402	NA	NA	MAS1
HDDC2	2.513864005	0.718404422	0.1540868	NA	NA	HDDC2
KCTD16	2.354394955	0.60252427	0.520402	NA	NA	KCTD16
BMPR1B	2.606890498	0.576279577	0.1540868	NA	NA	BMPR1B
TNNI3K	4.763428484	0.575831723	0	NA	NA	TNNI3K

Supplementary table 2

HAR PFOvsPT q<0.01

SYMBOL	PFOvsPT.d	PFOvsPT.FC	PFOvsPT.q	HAR	CAR	MAR
ADRA2A	-2.2026569	1.32088762	0	ADRA2A	ADRA2A	ADRA2A
AKT3	5.2245195	0.38230327	0	AKT3	AKT3	AKT3
ASTN2	-3.537837	1.8451722	0	ASTN2	ASTN2	ASTN2
BCL11A	2.8875415	0.57742488	0	BCL11A	BCL11A	BCL11A
BCL3	-2.7106502	2.07461849	0	BCL3	BCL3	NA
BMP2	-4.2137159	2.61468581	0	BMP2	BMP2	BMP2
C4orf32	2.3831704	0.76084728	0	C4orf32	C4orf32	C4orf32
CA10	-6.12	5.71221273	0	CA10	CA10	CA10
CDH8	-6.0646864	4.185182	0	CDH8	CDH8	CDH8
CNTN4	-4.2956365	2.05345693	0	CNTN4	CNTN4	CNTN4
CNTN6	-2.8124791	1.62508137	0	CNTN6	CNTN6	CNTN6
CTNNA2	2.3498011	0.72677424	0	CTNNA2	CTNNA2	CTNNA2
DPP10	2.3546219	0.6215833	0	DPP10	DPP10	DPP10
EGLN3	-2.9967726	1.72101012	0	EGLN3	EGLN3	EGLN3
FAM181B	-2.4483969	1.44723701	0	FAM181B	FAM181B	FAM181B
FAM5C	-6.2733541	4.38293974	0	FAM5C	FAM5C	FAM5C
FST	2.4569874	0.57303	0	FST	FST	FST
GBE1	-3.791506	1.82357854	0	GBE1	GBE1	GBE1
GFRA2	-3.4819788	2.03486324	0	GFRA2	GFRA2	GFRA2
KCNV1	-2.3638489	1.62181689	0	KCNV1	KCNV1	KCNV1
LHX2	5.766844	0.4639514	0	LHX2	LHX2	LHX2
LPHN2	4.2270257	0.47033437	0	LPHN2	LPHN2	LPHN2
LRRC8B	-2.8892578	1.4786014	0	LRRC8B	LRRC8B	LRRC8B
MEIS2	-8.0564215	3.20343535	0	MEIS2	MEIS2	MEIS2
MID1IP1	-3.3184109	1.50260718	0	MID1IP1	MID1IP1	MID1IP1
ADCYAP1	-3.0970772	1.61592882	0	ADCYAP1	NA	ADCYAP1
ADRB1	3.2483725	0.62843359	0	ADRB1	NA	ADRB1
CBLN4	-2.6478539	1.61876179	0	CBLN4	NA	CBLN4
CYTIP	2.420378	0.61081395	0	CYTIP	NA	CYTIP
FAM19A1	4.3485528	0.48800542	0	FAM19A1	NA	FAM19A1
FLJ13197	3.651636	0.51804829	0	FLJ13197	NA	FLJ13197
GPR64	4.0661047	0.33444216	0	GPR64	NA	GPR64
GSTA4	3.1060179	0.61447252	0	GSTA4	NA	GSTA4
HS3ST5	-2.6831055	1.77210432	0	HS3ST5	NA	HS3ST5
INHBA	3.8503447	0.53577238	0	INHBA	NA	INHBA
ITGBL1	-2.8160076	1.50576473	0	ITGBL1	NA	ITGBL1
KCNQ5	-3.4225801	1.81548163	0	KCNQ5	NA	KCNQ5
KIAA1797	2.2970172	0.70104402	0	KIAA1797	NA	KIAA1797
KLF9	-2.1921795	1.29718322	0	KLF9	NA	NA
L3MBTL4	-7.3135318	2.8540538	0	L3MBTL4	NA	L3MBTL4
LRRN3	3.7923133	0.62994277	0	LRRN3	NA	LRRN3
LYPD1	-2.40434	1.63547339	0	LYPD1	NA	LYPD1
MN1	-2.3740917	1.37684425	0	MN1	NA	MN1
NDST3	-5.1022264	3.64574782	0	NDST3	NA	NDST3
NEUROG2	-9.5369696	4.35523689	0	NEUROG2	NA	NA
PCDH10	-5.8434513	2.90835591	0	PCDH10	NA	PCDH10
PLEKHH2	2.7074491	0.60084142	0	PLEKHH2	NA	PLEKHH2

PLK2	-4.1808613	1.84061633	0	PLK2	NA	PLK2
POU3F2	-2.9474857	1.60823013	0	POU3F2	NA	POU3F2
PROKR2	6.4919927	0.36565726	0	PROKR2	NA	NA
RAB23	2.6716488	0.69797553	0	RAB23	NA	RAB23
RSPO2	4.4727342	0.45629497	0	RSPO2	NA	RSPO2
SDK1	-8.517135	2.97888689	0	SDK1	NA	SDK1
SLC17A6	6.1620466	0.33699863	0	SLC17A6	NA	NA
SLITRK1	-2.3569568	1.36395396	0	SLITRK1	NA	SLITRK1
SNAP25	-3.1847382	1.56861137	0	SNAP25	NA	NA
SPATA18	-2.9348408	1.57045958	0	SPATA18	NA	SPATA18
TCERG1L	-4.262795	2.66773624	0	TCERG1L	NA	TCERG1L
THBS1	5.2207093	0.27836758	0	THBS1	NA	THBS1
TIAM1	2.5587991	0.72165618	0	TIAM1	NA	NA
TMCC3	2.9003394	0.62058748	0	TMCC3	NA	TMCC3
VLDLR	-2.9318408	1.37952353	0	VLDLR	NA	NA
VSTM2L	-7.6922484	5.1814054	0	VSTM2L	NA	VSTM2L
NCAM2	3.8690279	0.48696699	0	NCAM2	NCAM2	NCAM2
OPCML	3.7307845	0.52898093	0	OPCML	OPCML	OPCML
PART1	-4.6456628	2.46597251	0	PART1	PART1	PART1
PCDH7	4.9943541	0.52826125	0	PCDH7	PCDH7	PCDH7
PCDH9	5.6119571	0.38338733	0	PCDH9	PCDH9	PCDH9
POU3F3	-2.3728614	1.478081	0	POU3F3	POU3F3	POU3F3
PRAGMIN	-5.4987885	2.1050636	0	PRAGMIN	PRAGMIN	PRAGMIN
PRR16	7.3717628	0.20836697	0	PRR16	PRR16	PRR16
ROBO1	-2.1733223	1.36044369	0	ROBO1	ROBO1	ROBO1
SORCS3	3.7751832	0.46911502	0	SORCS3	SORCS3	SORCS3
SOX8	2.2105106	0.62278216	0	SOX8	SOX8	NA
ST6GALNAC5	-6.0992162	2.36657776	0	ST6GALNAC5	ST6GALNAC5	ST6GALNAC5
TFAP2D	-3.5078229	3.65801776	0	TFAP2D	TFAP2D	TFAP2D
TRHDE	7.4632306	0.22727706	0	TRHDE	TRHDE	TRHDE
YES1	-3.7572556	1.84409437	0	YES1	YES1	YES1
ZBTB20	-2.2143973	1.32042789	0	ZBTB20	ZBTB20	ZBTB20
ARAP2	2.1194073	0.7517203	0.11329523	ARAP2	ARAP2	ARAP2
ARHGAP15	2.0953211	0.73642574	0.11329523	ARHGAP15	ARHGAP15	ARHGAP15
EFNB2	2.0686762	0.67820706	0.11329523	EFNB2	EFNB2	EFNB2
ELAVL2	-2.000231	1.49945115	0.11329523	ELAVL2	ELAVL2	ELAVL2
FLJ42709	2.0428552	0.71173922	0.11329523	FLJ42709	FLJ42709	FLJ42709
GLIS3	-2.1030173	1.66495303	0.11329523	GLIS3	GLIS3	GLIS3
ETV5	2.0643668	0.73596483	0.11329523	ETV5	NA	ETV5
FAM86B1	-2.0214709	1.32618968	0.11329523	FAM86B1	NA	NA
NFIA	-2.0249092	1.54756066	0.11329523	NFIA	NA	NFIA
NR4A3	2.0575626	0.77554613	0.11329523	NR4A3	NA	NR4A3
PMP22	-2.1046144	1.88169418	0.11329523	PMP22	NA	PMP22
SNX16	2.0222737	0.73542514	0.11329523	SNX16	NA	SNX16
THRB	-2.1344228	1.6017252	0.11329523	THRB	NA	THRB
RPS6KA3	2.0394529	0.74879937	0.11329523	RPS6KA3	RPS6KA3	RPS6KA3
C8orf83	1.9837388	0.75462987	0.19206239	C8orf83	C8orf83	C8orf83
CNTNAP5	1.8672665	0.7789539	0.19206239	CNTNAP5	CNTNAP5	CNTNAP5
ETV1	1.8694001	0.72920593	0.19206239	ETV1	ETV1	ETV1
FAM84A	1.882711	0.74357273	0.19206239	FAM84A	FAM84A	FAM84A
FANCL	1.9582831	0.77641708	0.19206239	FANCL	FANCL	FANCL
FBXL17	1.8906911	0.71481587	0.19206239	FBXL17	FBXL17	FBXL17
GRIA3	1.848451	0.74980662	0.19206239	GRIA3	GRIA3	GRIA3
MGST1	1.8604752	0.70169159	0.19206239	MGST1	MGST1	MGST1
C3orf58	1.9392572	0.78454523	0.19206239	C3orf58	NA	C3orf58
DNAJC1	1.8836523	0.7871951	0.19206239	DNAJC1	NA	DNAJC1
DOK6	1.968978	0.74194772	0.19206239	DOK6	NA	DOK6
KCNJ2	1.8946814	0.65167684	0.19206239	KCNJ2	NA	KCNJ2

KIAA0114	1.8648166	0.75999109	0.19206239	KIAA0114	NA	KIAA0114
LPHN3	-1.935682	1.28725915	0.19206239	LPHN3	NA	LPHN3
PLCB4	-1.9883246	1.38632628	0.19206239	PLCB4	NA	PLCB4
NLGN1	1.9729994	0.70393173	0.19206239	NLGN1	NLGN1	NLGN1
PARP8	-1.8990028	1.45305744	0.19206239	PARP8	PARP8	PARP8
POU3F1	-1.895519	1.42385159	0.19206239	POU3F1	POU3F1	POU3F1
PRKD1	-1.9272667	1.32258417	0.19206239	PRKD1	PRKD1	PRKD1
SATB1	1.8658506	0.79817306	0.19206239	SATB1	SATB1	SATB1
XYLT1	1.9222051	0.80021924	0.19206239	XYLT1	XYLT1	XYLT1
CA8	-1.7263499	1.23979534	0.56921718	CA8	CA8	CA8
CDH11	-1.6807595	1.26770628	0.56921718	CDH11	CDH11	CDH11
DCTD	-1.7916642	1.25421313	0.56921718	DCTD	DCTD	DCTD
DIO2	-1.6845813	1.34564495	0.56921718	DIO2	DIO2	DIO2
LRRTM1	-1.7425734	1.47680773	0.56921718	LRRTM1	LRRTM1	LRRTM1
BIN1	-1.7670401	1.42300715	0.56921718	BIN1	NA	BIN1
NMNAT3	-1.7568169	1.32096251	0.56921718	NMNAT3	NA	NMNAT3
NTN1	-1.7302086	1.30085813	0.56921718	NTN1	NA	NTN1
PBX1	-1.7364712	1.3212575	0.56921718	PBX1	NA	PBX1
TCF4	1.7294234	0.77062722	0.56921718	TCF4	NA	TCF4
PTPRG	1.7029704	0.73794978	0.56921718	PTPRG	PTPRG	PTPRG
RAP1GAP	1.7936958	0.72250668	0.56921718	RAP1GAP	RAP1GAP	RAP1GAP
SLC35F4	-1.7080815	1.50640057	0.56921718	SLC35F4	SLC35F4	SLC35F4
VRK2	1.6716374	0.6899739	0.56921718	VRK2	VRK2	VRK2
ZNF703	-1.7219587	1.2649145	0.56921718	ZNF703	ZNF703	ZNF703
CADM1	1.5423172	0.78415892	0.88419955	CADM1	CADM1	CADM1
CADM2	1.6480507	0.78473465	0.88419955	CADM2	CADM2	CADM2
CDC73	1.5686281	0.79791535	0.88419955	CDC73	CDC73	CDC73
CSMD1	-1.6330538	1.2029593	0.88419955	CSMD1	CSMD1	NA
EFNA5	1.6238383	0.80556294	0.88419955	EFNA5	EFNA5	EFNA5
HNMT	1.6412867	0.77996165	0.88419955	HNMT	HNMT	HNMT
KIF16B	1.6187313	0.77976982	0.88419955	KIF16B	KIF16B	KIF16B
LMO1	1.5536779	0.74571502	0.88419955	LMO1	LMO1	LMO1
LOC220930	1.5258504	0.73363307	0.88419955	LOC220930	LOC220930	LOC220930
LRRC37A	1.5081892	0.70175357	0.88419955	LRRC37A	LRRC37A	NA
CSMD3	-1.5303673	1.2347212	0.88419955	CSMD3	NA	CSMD3
ETV6	-1.5174112	1.34083786	0.88419955	ETV6	NA	ETV6
FTSJD1	1.615738	0.73069219	0.88419955	FTSJD1	NA	FTSJD1
ITGA4	1.5542557	0.80397129	0.88419955	ITGA4	NA	ITGA4
NRCAM	-1.5882778	1.23232595	0.88419955	NRCAM	NA	NRCAM
SLC7A11	-1.5125867	1.3357056	0.88419955	SLC7A11	NA	SLC7A11
STIM2	1.6017321	0.73637926	0.88419955	STIM2	NA	STIM2
PTPRD	1.601349	0.82378207	0.88419955	PTPRD	PTPRD	PTPRD
UNC5C	1.5826048	0.82539105	0.88419955	UNC5C	UNC5C	UNC5C

HAR-CAR PFOvsPT q<0.01

SYMBOL	PFOvsPT.d	PFOvsPT.FC	PFOvsPT.q	HAR	CAR	MAR
ADCYAP1	-3.0970772	1.61592882	0	ADCYAP1	NA	ADCYAP1
ADRB1	3.2483725	0.62843359	0	ADRB1	NA	ADRB1
CBLN4	-2.6478539	1.61876179	0	CBLN4	NA	CBLN4
CYTIP	2.420378	0.61081395	0	CYTIP	NA	CYTIP
FAM19A1	4.3485528	0.48800542	0	FAM19A1	NA	FAM19A1
FLJ13197	3.651636	0.51804829	0	FLJ13197	NA	FLJ13197
GPR64	4.0661047	0.33444216	0	GPR64	NA	GPR64

GSTA4	3.1060179	0.61447252	0	GSTA4	NA	GSTA4
HS3ST5	-2.6831055	1.77210432	0	HS3ST5	NA	HS3ST5
INHBA	3.8503447	0.53577238	0	INHBA	NA	INHBA
ITGBL1	-2.8160076	1.50576473	0	ITGBL1	NA	ITGBL1
KCNQ5	-3.4225801	1.81548163	0	KCNQ5	NA	KCNQ5
KIAA1797	2.2970172	0.70104402	0	KIAA1797	NA	KIAA1797
L3MBTL4	-7.3135318	2.8540538	0	L3MBTL4	NA	L3MBTL4
LRRN3	3.7923133	0.62994277	0	LRRN3	NA	LRRN3
LYPD1	-2.40434	1.63547339	0	LYPD1	NA	LYPD1
MN1	-2.3740917	1.37684425	0	MN1	NA	MN1
KLF9	-2.1921795	1.29718322	0	KLF9	NA	NA
NEUROG2	-9.5369696	4.35523689	0	NEUROG2	NA	NA
PROKR2	6.4919927	0.36565726	0	PROKR2	NA	NA
SLC17A6	6.1620466	0.33699863	0	SLC17A6	NA	NA
SNAP25	-3.1847382	1.56861137	0	SNAP25	NA	NA
TIAM1	2.5587991	0.72165618	0	TIAM1	NA	NA
VLDLR	-2.9318408	1.37952353	0	VLDLR	NA	NA
NDST3	-5.1022264	3.64574782	0	NDST3	NA	NDST3
PCDH10	-5.8434513	2.90835591	0	PCDH10	NA	PCDH10
PLEKHH2	2.7074491	0.60084142	0	PLEKHH2	NA	PLEKHH2
PLK2	-4.1808613	1.84061633	0	PLK2	NA	PLK2
POU3F2	-2.9474857	1.60823013	0	POU3F2	NA	POU3F2
RAB23	2.6716488	0.69797553	0	RAB23	NA	RAB23
RSPO2	4.4727342	0.45629497	0	RSPO2	NA	RSPO2
SDK1	-8.517135	2.97888689	0	SDK1	NA	SDK1
SLITRK1	-2.3569568	1.36395396	0	SLITRK1	NA	SLITRK1
SPATA18	-2.9348408	1.57045958	0	SPATA18	NA	SPATA18
TCERG1L	-4.262795	2.66773624	0	TCERG1L	NA	TCERG1L
THBS1	5.2207093	0.27836758	0	THBS1	NA	THBS1
TMCC3	2.9003394	0.62058748	0	TMCC3	NA	TMCC3
VSTM2L	-7.6922484	5.1814054	0	VSTM2L	NA	VSTM2L
ETV5	2.0643668	0.73596483	0.11329523	ETV5	NA	ETV5
FAM86B1	-2.0214709	1.32618968	0.11329523	FAM86B1	NA	NA
NFIA	-2.0249092	1.54756066	0.11329523	NFIA	NA	NFIA
NR4A3	2.0575626	0.77554613	0.11329523	NR4A3	NA	NR4A3
PMP22	-2.1046144	1.88169418	0.11329523	PMP22	NA	PMP22
SNX16	2.0222737	0.73542514	0.11329523	SNX16	NA	SNX16
THRB	-2.1344228	1.6017252	0.11329523	THRB	NA	THRB
C3orf58	1.9392572	0.78454523	0.19206239	C3orf58	NA	C3orf58
DNAJC1	1.8836523	0.7871951	0.19206239	DNAJC1	NA	DNAJC1
DOK6	1.968978	0.74194772	0.19206239	DOK6	NA	DOK6
KCNJ2	1.8946814	0.65167684	0.19206239	KCNJ2	NA	KCNJ2
KIAA0114	1.8648166	0.75999109	0.19206239	KIAA0114	NA	KIAA0114
LPHN3	-1.935682	1.28725915	0.19206239	LPHN3	NA	LPHN3
PLCB4	-1.9883246	1.38632628	0.19206239	PLCB4	NA	PLCB4
BIN1	-1.7670401	1.42300715	0.56921718	BIN1	NA	BIN1
NMNAT3	-1.7568169	1.32096251	0.56921718	NMNAT3	NA	NMNAT3
NTN1	-1.7302086	1.30085813	0.56921718	NTN1	NA	NTN1
PBX1	-1.7364712	1.3212575	0.56921718	PBX1	NA	PBX1
TCF4	1.7294234	0.77062722	0.56921718	TCF4	NA	TCF4
CSMD3	-1.5303673	1.2347212	0.88419955	CSMD3	NA	CSMD3
ETV6	-1.5174112	1.34083786	0.88419955	ETV6	NA	ETV6
FTSJD1	1.615738	0.73069219	0.88419955	FTSJD1	NA	FTSJD1
ITGA4	1.5542557	0.80397129	0.88419955	ITGA4	NA	ITGA4
NRCAM	-1.5882778	1.23232595	0.88419955	NRCAM	NA	NRCAM
SLC7A11	-1.5125867	1.3357056	0.88419955	SLC7A11	NA	SLC7A11
STIM2	1.6017321	0.73637926	0.88419955	STIM2	NA	STIM2

HAR only PFOvsPT q<0.01

SYMBOL	PFOvsPT.d	PFOvsPT.FC	PFOvsPT.q	HAR	CAR	MAR
KLF9	-2.1921795	1.29718322	0	KLF9	NA	NA
NEUROG2	-9.5369696	4.35523689	0	NEUROG2	NA	NA
PROKR2	6.4919927	0.36565726	0	PROKR2	NA	NA
SLC17A6	6.1620466	0.33699863	0	SLC17A6	NA	NA
SNAP25	-3.1847382	1.56861137	0	SNAP25	NA	NA
TIAM1	2.5587991	0.72165618	0	TIAM1	NA	NA
VLDLR	-2.9318408	1.37952353	0	VLDLR	NA	NA
FAM86B1	-2.0214709	1.32618968	0.11329523	FAM86B1	NA	NA

CAR PFOvsPT q<0.01

SYMBOL	PFOvsPT.d	PFOvsPT.FC	PFOvsPT.q	HAR	CAR	MAR
ADRA2A	-2.2026569	1.32088762	0	ADRA2A	ADRA2A	ADRA2A
AKT3	5.2245195	0.38230327	0	AKT3	AKT3	AKT3
ASTN2	-3.537837	1.8451722	0	ASTN2	ASTN2	ASTN2
BCL11A	2.8875415	0.57742488	0	BCL11A	BCL11A	BCL11A
BCL3	-2.7106502	2.07461849	0	BCL3	BCL3	NA
BMP2	-4.2137159	2.61468581	0	BMP2	BMP2	BMP2
C4orf32	2.3831704	0.76084728	0	C4orf32	C4orf32	C4orf32
CA10	-6.12	5.71221273	0	CA10	CA10	CA10
CDH8	-6.0646864	4.185182	0	CDH8	CDH8	CDH8
CNTN4	-4.2956365	2.05345693	0	CNTN4	CNTN4	CNTN4
CNTN6	-2.8124791	1.62508137	0	CNTN6	CNTN6	CNTN6
CTNNA2	2.3498011	0.72677424	0	CTNNA2	CTNNA2	CTNNA2
DPP10	2.3546219	0.6215833	0	DPP10	DPP10	DPP10
EGLN3	-2.9967726	1.72101012	0	EGLN3	EGLN3	EGLN3
FAM181B	-2.4483969	1.44723701	0	FAM181B	FAM181B	FAM181B
FAM5C	-6.2733541	4.38293974	0	FAM5C	FAM5C	FAM5C
FST	2.4569874	0.57303	0	FST	FST	FST
GBE1	-3.791506	1.82357854	0	GBE1	GBE1	GBE1
GFRA2	-3.4819788	2.03486324	0	GFRA2	GFRA2	GFRA2
KCNV1	-2.3638489	1.62181689	0	KCNV1	KCNV1	KCNV1
LHX2	5.766844	0.4639514	0	LHX2	LHX2	LHX2
LPHN2	4.2270257	0.47033437	0	LPHN2	LPHN2	LPHN2
LRRC8B	-2.8892578	1.4786014	0	LRRC8B	LRRC8B	LRRC8B
MEIS2	-8.0564215	3.20343535	0	MEIS2	MEIS2	MEIS2
MID1IP1	-3.3184109	1.50260718	0	MID1IP1	MID1IP1	MID1IP1
ACCN1	-2.6307648	1.67979598	0	NA	ACCN1	ACCN1
ADAMTS16	-2.3622011	1.76005451	0	NA	ADAMTS16	ADAMTS16
AK5	2.4672315	0.67898931	0	NA	AK5	AK5
ANKRD32	-5.2441495	2.29550366	0	NA	ANKRD32	ANKRD32
APOO	-2.2526913	1.32642345	0	NA	APOO	APOO
ARHGDIB	-2.6187164	1.37947451	0	NA	ARHGDIB	NA
ARNTL	2.5357596	0.65285149	0	NA	ARNTL	ARNTL
CACNA1A	-5.7244908	2.02037437	0	NA	CACNA1A	NA
CACNA2D3	-5.0183639	2.37311616	0	NA	CACNA2D3	CACNA2D3
CBLN1	-7.7041147	4.80719481	0	NA	CBLN1	CBLN1

CCDC109A	-2.989666	1.63539425	0	NA	CCDC109A	CCDC109A
CDH12	-3.0931634	1.70193601	0	NA	CDH12	CDH12
CDH9	3.3472538	0.50238639	0	NA	CDH9	CDH9
CEP120	-2.8706623	1.55083986	0	NA	CEP120	CEP120
CGRRF1	-4.1255188	2.23860247	0	NA	CGRRF1	CGRRF1
CNKSR3	-5.9495054	2.9706848	0	NA	CNKSR3	NA
CNTN3	-5.1226552	2.1416389	0	NA	CNTN3	CNTN3
COX7A1	3.6220653	0.49840726	0	NA	COX7A1	COX7A1
CPNE8	-7.9019211	5.45090012	0	NA	CPNE8	CPNE8
CTNNBL1	-5.1701204	2.60508807	0	NA	CTNNBL1	CTNNBL1
CTXN3	-3.1547723	2.02249669	0	NA	CTXN3	CTXN3
CXorf1	3.5295083	0.5214482	0	NA	CXorf1	CXorf1
CYP27C1	-4.0171215	2.88173718	0	NA	CYP27C1	NA
DACH2	5.3983733	0.39389626	0	NA	DACH2	DACH2
DACT1	3.7612088	0.6514341	0	NA	DACT1	DACT1
DCBLD1	2.2063284	0.71430327	0	NA	DCBLD1	NA
DGKI	2.8821568	0.44509385	0	NA	DGKI	DGKI
DLG1	3.1684757	0.60089689	0	NA	DLG1	DLG1
DNAH10	-2.8406866	1.5059201	0	NA	DNAH10	NA
DOK5	3.4689382	0.68277451	0	NA	DOK5	DOK5
DYRK2	2.3302515	0.68671835	0	NA	DYRK2	DYRK2
EPHA4	-2.3288721	1.52144273	0	NA	EPHA4	EPHA4
FAM19A5	-2.2537914	1.36434919	0	NA	FAM19A5	FAM19A5
FAM5B	-2.7524322	1.60087371	0	NA	FAM5B	FAM5B
FGF12	-3.1985688	1.60028385	0	NA	FGF12	FGF12
FGF14	-4.161336	1.89013512	0	NA	FGF14	FGF14
FLJ43390	-9.7499031	5.56194442	0	NA	FLJ43390	FLJ43390
FLRT3	-6.0329061	2.31581298	0	NA	FLRT3	FLRT3
FRMPD4	2.6700364	0.72369361	0	NA	FRMPD4	FRMPD4
FSIP1	3.2066337	0.54366788	0	NA	FSIP1	NA
GABRA1	-2.4861275	1.5458172	0	NA	GABRA1	GABRA1
GABRA5	4.1635031	0.57212962	0	NA	GABRA5	GABRA5
GABRG2	-2.500529	1.35616898	0	NA	GABRG2	GABRG2
GAS1	-2.8207017	1.43925682	0	NA	GAS1	GAS1
GOLSYN	3.3184939	0.65282491	0	NA	GOLSYN	GOLSYN
GRB14	2.5728727	0.49303609	0	NA	GRB14	GRB14
H2AFY2	-2.909317	1.40106706	0	NA	H2AFY2	H2AFY2
HLF	4.0234794	0.4754812	0	NA	HLF	HLF
ID2	3.8786506	0.44944894	0	NA	ID2	ID2
IGFBP4	2.3828134	0.68418134	0	NA	IGFBP4	IGFBP4
IGFBP5	-2.834741	1.61843461	0	NA	IGFBP5	IGFBP5
IGSF11	-7.5561874	3.09731621	0	NA	IGSF11	IGSF11
KIAA0182	2.8095351	0.6575132	0	NA	KIAA0182	KIAA0182
KLF6	-2.7404503	1.42932138	0	NA	KLF6	KLF6
LGALS8	2.2259948	0.72480252	0	NA	LGALS8	LGALS8
LHFP	3.3336246	0.60532507	0	NA	LHFP	LHFP
LMO3	-2.4423344	1.621965	0	NA	LMO3	LMO3
LMO4	-5.4333042	2.09762329	0	NA	LMO4	LMO4
LOC150568	-4.5808282	2.16132196	0	NA	LOC150568	LOC150568
LOC283392	3.2109194	0.58737246	0	NA	LOC283392	LOC283392
LOC645323	2.2641538	0.74917028	0	NA	LOC645323	LOC645323
LOC653786	2.4826133	0.66960833	0	NA	LOC653786	LOC653786
LRRTM4	5.9239675	0.23561301	0	NA	LRRTM4	LRRTM4
MAMLD1	-2.732106	1.53315954	0	NA	MAMLD1	MAMLD1
MARCH3	-5.561013	1.98423491	0	NA	MARCH3	MARCH3
MAST4	-4.5965819	1.89897414	0	NA	MAST4	MAST4
MC4R	-2.7448307	2.07739068	0	NA	MC4R	MC4R
MDGA1	-2.3368477	1.47049165	0	NA	MDGA1	MDGA1

MEIS3P1	-4.0994783	3.16136077	0	NA	MEIS3P1	NA
MID1	3.3365625	0.56004233	0	NA	MID1	MID1
MOSC1	-2.4501897	1.4052336	0	NA	MOSC1	MOSC1
NDP	-4.9907327	3.07052035	0	NA	NDP	NDP
NOG	-3.1842631	2.05302024	0	NA	NOG	NOG
NR3C1	3.9886444	0.55590161	0	NA	NR3C1	NR3C1
NR3C2	6.2948111	0.35436521	0	NA	NR3C2	NR3C2
NR4A2	5.3728796	0.31034033	0	NA	NR4A2	NR4A2
NTNG2	5.0387291	0.27228346	0	NA	NTNG2	NTNG2
NXPH3	3.2378358	0.63766383	0	NA	NXPH3	NXPH3
ODZ4	2.4831271	0.71491135	0	NA	ODZ4	ODZ4
OLFM3	-2.3427178	1.60992788	0	NA	OLFM3	OLFM3
PBX3	2.6304097	0.71030199	0	NA	PBX3	PBX3
PCDH17	-5.0671224	2.34152922	0	NA	PCDH17	PCDH17
PCDH18	-2.4106968	1.50798322	0	NA	PCDH18	PCDH18
PCSK9	2.8585448	0.66795293	0	NA	PCSK9	PCSK9
PDE4DIP	2.650362	0.69291976	0	NA	PDE4DIP	PDE4DIP
PDZRN4	-5.4476486	2.63707924	0	NA	PDZRN4	PDZRN4
PHLDA1	-4.0501691	1.65922624	0	NA	PHLDA1	PHLDA1
PIK3AP1	2.7624911	0.64434479	0	NA	PIK3AP1	PIK3AP1
PKIG	-2.4710853	1.51923202	0	NA	PKIG	NA
PLAGL1	2.7294518	0.62240269	0	NA	PLAGL1	NA
PPARGC1A	2.5259817	0.69605157	0	NA	PPARGC1A	PPARGC1A
PSD2	-2.1802477	1.30380301	0	NA	PSD2	PSD2
PTER	-3.937988	2.21395294	0	NA	PTER	PTER
PTPRK	4.7632103	0.3977537	0	NA	PTPRK	PTPRK
QDPR	-2.1979566	1.33072736	0	NA	QDPR	QDPR
RAB7L1	3.8609759	0.56498331	0	NA	RAB7L1	RAB7L1
RGS20	2.36348	0.71130303	0	NA	RGS20	RGS20
RNF182	4.7106408	0.47962861	0	NA	RNF182	RNF182
ROBO2	5.3895655	0.46474463	0	NA	ROBO2	ROBO2
RPRM	-3.0311581	2.03229418	0	NA	RPRM	RPRM
SFRP1	-3.1715505	1.60973547	0	NA	SFRP1	SFRP1
SFRP2	-2.9745118	1.69062342	0	NA	SFRP2	SFRP2
SLC16A1	3.2925757	0.59571496	0	NA	SLC16A1	NA
SLC6A15	2.7910928	0.67529137	0	NA	SLC6A15	SLC6A15
SLCO3A1	-2.3528977	1.73020418	0	NA	SLCO3A1	SLCO3A1
SLIT2	-3.0879381	1.56586266	0	NA	SLIT2	SLIT2
SORCS1	4.3988439	0.48451882	0	NA	SORCS1	SORCS1
SPON1	5.0932677	0.30520412	0	NA	SPON1	SPON1
ST8SIA3	-2.4241874	1.47965344	0	NA	ST8SIA3	ST8SIA3
STXBP6	-7.5596973	3.29939452	0	NA	STXBP6	STXBP6
SULF2	-4.7353515	2.57250946	0	NA	SULF2	SULF2
SYNE1	2.7432808	0.71460998	0	NA	SYNE1	SYNE1
SYNM	-3.2827036	1.63034073	0	NA	SYNM	SYNM
TLE1	9.2333799	0.33331922	0	NA	TLE1	TLE1
TMCC2	2.363984	0.72802771	0	NA	TMCC2	TMCC2
TNFAIP8	2.5452211	0.57765687	0	NA	TNFAIP8	NA
TOX	-6.2470255	3.16050207	0	NA	TOX	TOX
TPBG	-2.3870222	1.57377889	0	NA	TPBG	TPBG
TRH	-4.3485877	1.92544255	0	NA	TRH	TRH
WBSCR17	-3.4505242	1.54274478	0	NA	WBSCR17	WBSCR17
WNT11	-2.185204	1.57874503	0	NA	WNT11	WNT11
ZNF238	2.8390867	0.596913	0	NA	ZNF238	ZNF238
ZNF423	-6.9022481	4.59746511	0	NA	ZNF423	ZNF423
NCAM2	3.8690279	0.48696699	0	NCAM2	NCAM2	NCAM2
OPCML	3.7307845	0.52898093	0	OPCML	OPCML	OPCML
PART1	-4.6456628	2.46597251	0	PART1	PART1	PART1

PCDH7	4.9943541	0.52826125	0	PCDH7	PCDH7	PCDH7
PCDH9	5.6119571	0.38338733	0	PCDH9	PCDH9	PCDH9
POU3F3	-2.3728614	1.478081	0	POU3F3	POU3F3	POU3F3
PRAGMIN	-5.4987885	2.1050636	0	PRAGMIN	PRAGMIN	PRAGMIN
PRR16	7.3717628	0.20836697	0	PRR16	PRR16	PRR16
ROBO1	-2.1733223	1.36044369	0	ROBO1	ROBO1	ROBO1
SORCS3	3.7751832	0.46911502	0	SORCS3	SORCS3	SORCS3
SOX8	2.2105106	0.62278216	0	SOX8	SOX8	NA
ST6GALNAC5	-6.0992162	2.36657776	0	ST6GALNAC!	ST6GALNAC5	ST6GALNAC5
TFAP2D	-3.5078229	3.65801776	0	TFAP2D	TFAP2D	TFAP2D
TRHDE	7.4632306	0.22727706	0	TRHDE	TRHDE	TRHDE
YES1	-3.7572556	1.84409437	0	YES1	YES1	YES1
ZBTB20	-2.2143973	1.32042789	0	ZBTB20	ZBTB20	ZBTB20
ARAP2	2.1194073	0.7517203	0.11329523	ARAP2	ARAP2	ARAP2
ARHGAP15	2.0953211	0.73642574	0.11329523	ARHGAP15	ARHGAP15	ARHGAP15
EFNB2	2.0686762	0.67820706	0.11329523	EFNB2	EFNB2	EFNB2
ELAVL2	-2.000231	1.49945115	0.11329523	ELAVL2	ELAVL2	ELAVL2
FLJ42709	2.0428552	0.71173922	0.11329523	FLJ42709	FLJ42709	FLJ42709
GLIS3	-2.1030173	1.66495303	0.11329523	GLIS3	GLIS3	GLIS3
ASAM	-2.1306034	1.62637404	0.11329523	NA	ASAM	NA
C13orf18	2.0554786	0.66207045	0.11329523	NA	C13orf18	NA
C5orf4	2.0449505	0.75038304	0.11329523	NA	C5orf4	C5orf4
DPF3	-1.9948572	1.32280327	0.11329523	NA	DPF3	DPF3
EIF3E	2.1041583	0.79564492	0.11329523	NA	EIF3E	EIF3E
GGTA1	-2.1053034	1.54639322	0.11329523	NA	GGTA1	GGTA1
GOLGA7B	-2.1550231	1.44318056	0.11329523	NA	GOLGA7B	GOLGA7B
GPAM	2.140471	0.68699697	0.11329523	NA	GPAM	GPAM
GRIK2	-2.1000341	1.32784063	0.11329523	NA	GRIK2	GRIK2
GRM7	-1.9945815	1.33335681	0.11329523	NA	GRM7	GRM7
HSPB3	-2.111997	1.51116191	0.11329523	NA	HSPB3	HSPB3
HTR7	2.0742972	0.71424476	0.11329523	NA	HTR7	HTR7
PLP2	2.1639678	0.71837011	0.11329523	NA	PLP2	NA
SATB2	2.0138674	0.6652304	0.11329523	NA	SATB2	SATB2
SH3BP4	-2.1323932	1.35398485	0.11329523	NA	SH3BP4	SH3BP4
TARBP1	2.0160223	0.77167533	0.11329523	NA	TARBP1	TARBP1
TBX21	-2.0951755	1.50634971	0.11329523	NA	TBX21	TBX21
RPS6KA3	2.0394529	0.74879937	0.11329523	RPS6KA3	RPS6KA3	RPS6KA3
C8orf83	1.9837388	0.75462987	0.19206239	C8orf83	C8orf83	C8orf83
CNTNAP5	1.8672665	0.7789539	0.19206239	CNTNAP5	CNTNAP5	CNTNAP5
ETV1	1.8694001	0.72920593	0.19206239	ETV1	ETV1	ETV1
FAM84A	1.882711	0.74357273	0.19206239	FAM84A	FAM84A	FAM84A
FANCL	1.9582831	0.77641708	0.19206239	FANCL	FANCL	FANCL
FBXL17	1.8906911	0.71481587	0.19206239	FBXL17	FBXL17	FBXL17
GRIA3	1.848451	0.74980662	0.19206239	GRIA3	GRIA3	GRIA3
MGST1	1.8604752	0.70169159	0.19206239	MGST1	MGST1	MGST1
ACVR1	1.9016813	0.74909317	0.19206239	NA	ACVR1	ACVR1
AKAP13	1.8672226	0.77684803	0.19206239	NA	AKAP13	AKAP13
ASS1	-1.8757098	1.36348358	0.19206239	NA	ASS1	ASS1
C9orf45	1.9496523	0.75910403	0.19206239	NA	C9orf45	C9orf45
CCDC110	1.8985224	0.67066792	0.19206239	NA	CCDC110	NA
CYYR1	-1.8344824	1.39509513	0.19206239	NA	CYYR1	CYYR1
DPP3	-1.8794705	1.2852484	0.19206239	NA	DPP3	DPP3
DPYD	1.91158	0.75716218	0.19206239	NA	DPYD	DPYD
HS3ST2	-1.9807592	1.51306092	0.19206239	NA	HS3ST2	HS3ST2
KLHL14	-1.8384008	1.35188201	0.19206239	NA	KLHL14	KLHL14
KLHL21	1.8221708	0.7030475	0.19206239	NA	KLHL21	NA
LOC151009	1.9328236	0.70324145	0.19206239	NA	LOC151009	NA
NGEF	1.9272593	0.79921309	0.19206239	NA	NGEF	NA

NOS1	-1.9311619	1.2985339	0.19206239	NA	NOS1	NOS1
PLOD2	1.8199163	0.63535707	0.19206239	NA	PLOD2	PLOD2
RLIM	1.8984403	0.79797798	0.19206239	NA	RLIM	NA
RSL24D1	1.9607116	0.76287592	0.19206239	NA	RSL24D1	RSL24D1
SLC22A15	-1.8528495	1.3029058	0.19206239	NA	SLC22A15	NA
SNX9	1.8759538	0.72844561	0.19206239	NA	SNX9	SNX9
TMEM111	1.8818937	0.74576381	0.19206239	NA	TMEM111	NA
ZADH2	-1.9388644	1.33605933	0.19206239	NA	ZADH2	ZADH2
ZBTB44	1.92715	0.78897914	0.19206239	NA	ZBTB44	NA
ZNF706	-1.8489992	1.22405684	0.19206239	NA	ZNF706	ZNF706
NLGN1	1.9729994	0.70393173	0.19206239	NLGN1	NLGN1	NLGN1
PARP8	-1.8990028	1.45305744	0.19206239	PARP8	PARP8	PARP8
POU3F1	-1.895519	1.42385159	0.19206239	POU3F1	POU3F1	POU3F1
PRKD1	-1.9272667	1.32258417	0.19206239	PRKD1	PRKD1	PRKD1
SATB1	1.8658506	0.79817306	0.19206239	SATB1	SATB1	SATB1
XYLT1	1.9222051	0.80021924	0.19206239	XYLT1	XYLT1	XYLT1
CA8	-1.7263499	1.23979534	0.56921718	CA8	CA8	CA8
CDH11	-1.6807595	1.26770628	0.56921718	CDH11	CDH11	CDH11
DCTD	-1.7916642	1.25421313	0.56921718	DCTD	DCTD	DCTD
DIO2	-1.6845813	1.34564495	0.56921718	DIO2	DIO2	DIO2
LRRTM1	-1.7425734	1.47680773	0.56921718	LRRTM1	LRRTM1	LRRTM1
AMOTL1	-1.6708045	1.2233893	0.56921718	NA	AMOTL1	AMOTL1
C1orf201	1.6867839	0.76162761	0.56921718	NA	C1orf201	C1orf201
CHGB	1.6889775	0.80786526	0.56921718	NA	CHGB	CHGB
CIDEA	1.6688155	0.73169435	0.56921718	NA	CIDEA	NA
DIAPH3	-1.691133	1.316908	0.56921718	NA	DIAPH3	DIAPH3
DUSP6	-1.6648534	1.34244361	0.56921718	NA	DUSP6	DUSP6
FBXL4	1.7030863	0.74322208	0.56921718	NA	FBXL4	FBXL4
FOSL2	-1.8048882	1.39235042	0.56921718	NA	FOSL2	FOSL2
GRM3	1.7963915	0.79517321	0.56921718	NA	GRM3	GRM3
ID4	-1.714183	1.24941014	0.56921718	NA	ID4	ID4
LRRC4C	1.7723172	0.78380855	0.56921718	NA	LRRC4C	LRRC4C
MAOA	-1.7230175	1.26439033	0.56921718	NA	MAOA	MAOA
MIAT	1.6685052	0.7468721	0.56921718	NA	MIAT	MIAT
MXRA7	-1.7854284	1.28071002	0.56921718	NA	MXRA7	NA
NGB	1.6973064	0.81316304	0.56921718	NA	NGB	NGB
NINJ1	1.6946936	0.79883026	0.56921718	NA	NINJ1	NA
NR2F2	1.6810585	0.78707751	0.56921718	NA	NR2F2	NR2F2
NRXN3	-1.6779785	1.23251032	0.56921718	NA	NRXN3	NRXN3
PLXNC1	-1.7689523	1.29644548	0.56921718	NA	PLXNC1	PLXNC1
RAPGEFL1	1.7176713	0.71074745	0.56921718	NA	RAPGEFL1	RAPGEFL1
RUNX2	-1.6858028	1.28467826	0.56921718	NA	RUNX2	RUNX2
SMAD3	1.7388772	0.78593321	0.56921718	NA	SMAD3	SMAD3
TMIGD2	1.7168483	0.7748928	0.56921718	NA	TMIGD2	NA
ZAK	1.670217	0.71519869	0.56921718	NA	ZAK	ZAK
ZHX2	1.7521996	0.76600304	0.56921718	NA	ZHX2	ZHX2
PTPRG	1.7029704	0.73794978	0.56921718	PTPRG	PTPRG	PTPRG
RAP1GAP	1.7936958	0.72250668	0.56921718	RAP1GAP	RAP1GAP	RAP1GAP
SLC35F4	-1.7080815	1.50640057	0.56921718	SLC35F4	SLC35F4	SLC35F4
VRK2	1.6716374	0.6899739	0.56921718	VRK2	VRK2	VRK2
ZNF703	-1.7219587	1.2649145	0.56921718	ZNF703	ZNF703	ZNF703
CADM1	1.5423172	0.78415892	0.88419955	CADM1	CADM1	CADM1
CADM2	1.6480507	0.78473465	0.88419955	CADM2	CADM2	CADM2
CDC73	1.5686281	0.79791535	0.88419955	CDC73	CDC73	CDC73
CSMD1	-1.6330538	1.2029593	0.88419955	CSMD1	CSMD1	NA
EFNA5	1.6238383	0.80556294	0.88419955	EFNA5	EFNA5	EFNA5
HNMT	1.6412867	0.77996165	0.88419955	HNMT	HNMT	HNMT
KIF16B	1.6187313	0.77976982	0.88419955	KIF16B	KIF16B	KIF16B

LMO1	1.5536779	0.74571502	0.88419955	LMO1	LMO1	LMO1
LOC220930	1.5258504	0.73363307	0.88419955	LOC220930	LOC220930	LOC220930
LRRC37A	1.5081892	0.70175357	0.88419955	LRRC37A	LRRC37A	NA
AUH	1.5088174	0.79367185	0.88419955	NA	AUH	AUH
CDH13	1.5766612	0.71012446	0.88419955	NA	CDH13	CDH13
CLCN4	-1.6496394	1.35400775	0.88419955	NA	CLCN4	CLCN4
CPLX3	1.6128539	0.80616955	0.88419955	NA	CPLX3	NA
CRIPAK	1.5992703	0.7591401	0.88419955	NA	CRIPAK	NA
DCC	-1.5468003	1.25641969	0.88419955	NA	DCC	DCC
DMXL1	1.5936059	0.79464748	0.88419955	NA	DMXL1	DMXL1
DNMT3A	-1.6393412	1.26259462	0.88419955	NA	DNMT3A	DNMT3A
KBTBD8	1.5720203	0.79932808	0.88419955	NA	KBTBD8	KBTBD8
KIAA1958	-1.5262747	1.26307324	0.88419955	NA	KIAA1958	KIAA1958
MARK1	1.5520661	0.80040881	0.88419955	NA	MARK1	MARK1
MDH1	1.6458122	0.7827652	0.88419955	NA	MDH1	MDH1
MITF	1.626352	0.7826945	0.88419955	NA	MITF	MITF
MYO1B	-1.6535742	1.26905342	0.88419955	NA	MYO1B	MYO1B
PREB	-1.5009519	1.19427223	0.88419955	NA	PREB	NA
PRKCB	1.623504	0.7653644	0.88419955	NA	PRKCB	PRKCB
PSRC1	-1.6109387	1.34653818	0.88419955	NA	PSRC1	NA
SCO2	-1.6514943	1.26470566	0.88419955	NA	SCO2	NA
SEPX1	-1.5896316	1.26272394	0.88419955	NA	SEPX1	NA
SH3KBP1	1.6217678	0.81439028	0.88419955	NA	SH3KBP1	SH3KBP1
SMAD7	1.5822772	0.77254442	0.88419955	NA	SMAD7	SMAD7
SPRED2	-1.5160014	1.21935135	0.88419955	NA	SPRED2	SPRED2
STAG2	1.5384991	0.80153576	0.88419955	NA	STAG2	STAG2
SYT13	1.5262234	0.77492133	0.88419955	NA	SYT13	SYT13
TDP1	-1.6031528	1.24968789	0.88419955	NA	TDP1	TDP1
TMEM47	-1.6560851	1.2938101	0.88419955	NA	TMEM47	TMEM47
TMEM5	-1.5773597	1.27996903	0.88419955	NA	TMEM5	TMEM5
TP53BP2	1.5084482	0.80778045	0.88419955	NA	TP53BP2	NA
ZNF395	-1.598808	1.21514445	0.88419955	NA	ZNF395	ZNF395
PTPRD	1.601349	0.82378207	0.88419955	PTPRD	PTPRD	PTPRD
UNC5C	1.5826048	0.82539105	0.88419955	UNC5C	UNC5C	UNC5C

CAR-HAR PFOvsPT q<0.01

SYMBOL	PFOvsPT.d	PFOvsPT.FC	PFOvsPT.q	HAR	CAR	MAR
ACCN1	-2.6307648	1.67979598	0	NA	ACCN1	ACCN1
ADAMTS16	-2.3622011	1.76005451	0	NA	ADAMTS16	ADAMTS16
AK5	2.4672315	0.67898931	0	NA	AK5	AK5
ANKRD32	-5.2441495	2.29550366	0	NA	ANKRD32	ANKRD32
APOO	-2.2526913	1.32642345	0	NA	APOO	APOO
ARNTL	2.5357596	0.65285149	0	NA	ARNTL	ARNTL
CACNA2D3	-5.0183639	2.37311616	0	NA	CACNA2D3	CACNA2D3
CBLN1	-7.7041147	4.80719481	0	NA	CBLN1	CBLN1
CCDC109A	-2.989666	1.63539425	0	NA	CCDC109A	CCDC109A
CDH12	-3.0931634	1.70193601	0	NA	CDH12	CDH12
CDH9	3.3472538	0.50238639	0	NA	CDH9	CDH9
CEP120	-2.8706623	1.55083986	0	NA	CEP120	CEP120
CGRRF1	-4.1255188	2.23860247	0	NA	CGRRF1	CGRRF1
CNTN3	-5.1226552	2.1416389	0	NA	CNTN3	CNTN3
COX7A1	3.6220653	0.49840726	0	NA	COX7A1	COX7A1
CPNE8	-7.9019211	5.45090012	0	NA	CPNE8	CPNE8
CTNNBL1	-5.1701204	2.60508807	0	NA	CTNNBL1	CTNNBL1

CTXN3	-3.1547723	2.02249669	0	NA	CTXN3	CTXN3
CXorf1	3.5295083	0.5214482	0	NA	CXorf1	CXorf1
DACH2	5.3983733	0.39389626	0	NA	DACH2	DACH2
DACT1	3.7612088	0.6514341	0	NA	DACT1	DACT1
DGKI	2.8821568	0.44509385	0	NA	DGKI	DGKI
DLG1	3.1684757	0.60089689	0	NA	DLG1	DLG1
DOK5	3.4689382	0.68277451	0	NA	DOK5	DOK5
DYRK2	2.3302515	0.68671835	0	NA	DYRK2	DYRK2
EPHA4	-2.3288721	1.52144273	0	NA	EPHA4	EPHA4
FAM19A5	-2.2537914	1.36434919	0	NA	FAM19A5	FAM19A5
FAM5B	-2.7524322	1.60087371	0	NA	FAM5B	FAM5B
FGF12	-3.1985688	1.60028385	0	NA	FGF12	FGF12
FGF14	-4.161336	1.89013512	0	NA	FGF14	FGF14
FLJ43390	-9.7499031	5.56194442	0	NA	FLJ43390	FLJ43390
FLRT3	-6.0329061	2.31581298	0	NA	FLRT3	FLRT3
FRMPD4	2.6700364	0.72369361	0	NA	FRMPD4	FRMPD4
GABRA1	-2.4861275	1.5458172	0	NA	GABRA1	GABRA1
GABRA5	4.1635031	0.57212962	0	NA	GABRA5	GABRA5
GABRG2	-2.500529	1.35616898	0	NA	GABRG2	GABRG2
GAS1	-2.8207017	1.43925682	0	NA	GAS1	GAS1
GOLSYN	3.3184939	0.65282491	0	NA	GOLSYN	GOLSYN
GRB14	2.5728727	0.49303609	0	NA	GRB14	GRB14
H2AFY2	-2.909317	1.40106706	0	NA	H2AFY2	H2AFY2
HLF	4.0234794	0.4754812	0	NA	HLF	HLF
ID2	3.8786506	0.44944894	0	NA	ID2	ID2
IGFBP4	2.3828134	0.68418134	0	NA	IGFBP4	IGFBP4
IGFBP5	-2.834741	1.61843461	0	NA	IGFBP5	IGFBP5
IGSF11	-7.5561874	3.09731621	0	NA	IGSF11	IGSF11
KIAA0182	2.8095351	0.6575132	0	NA	KIAA0182	KIAA0182
KLF6	-2.7404503	1.42932138	0	NA	KLF6	KLF6
LGALS8	2.2259948	0.72480252	0	NA	LGALS8	LGALS8
LHFP	3.3336246	0.60532507	0	NA	LHFP	LHFP
LMO3	-2.4423344	1.621965	0	NA	LMO3	LMO3
LMO4	-5.4333042	2.09762329	0	NA	LMO4	LMO4
LOC150568	-4.5808282	2.16132196	0	NA	LOC150568	LOC150568
LOC283392	3.2109194	0.58737246	0	NA	LOC283392	LOC283392
LOC645323	2.2641538	0.74917028	0	NA	LOC645323	LOC645323
LOC653786	2.4826133	0.66960833	0	NA	LOC653786	LOC653786
LRRTM4	5.9239675	0.23561301	0	NA	LRRTM4	LRRTM4
MAMLD1	-2.732106	1.53315954	0	NA	MAMLD1	MAMLD1
MARCH3	-5.561013	1.98423491	0	NA	MARCH3	MARCH3
MAST4	-4.5965819	1.89897414	0	NA	MAST4	MAST4
MC4R	-2.7448307	2.07739068	0	NA	MC4R	MC4R
MDGA1	-2.3368477	1.47049165	0	NA	MDGA1	MDGA1
MID1	3.3365625	0.56004233	0	NA	MID1	MID1
MOSC1	-2.4501897	1.4052336	0	NA	MOSC1	MOSC1
ARHGDIB	-2.6187164	1.37947451	0	NA	ARHGDIB	NA
CACNA1A	-5.7244908	2.02037437	0	NA	CACNA1A	NA
CNKSR3	-5.9495054	2.9706848	0	NA	CNKSR3	NA
CYP27C1	-4.0171215	2.88173718	0	NA	CYP27C1	NA
DCBLD1	2.2063284	0.71430327	0	NA	DCBLD1	NA
DNAH10	-2.8406866	1.5059201	0	NA	DNAH10	NA
FSIP1	3.2066337	0.54366788	0	NA	FSIP1	NA
MEIS3P1	-4.0994783	3.16136077	0	NA	MEIS3P1	NA
PKIG	-2.4710853	1.51923202	0	NA	PKIG	NA
PLAGL1	2.7294518	0.62240269	0	NA	PLAGL1	NA
SLC16A1	3.2925757	0.59571496	0	NA	SLC16A1	NA
TNFAIP8	2.5452211	0.57765687	0	NA	TNFAIP8	NA

NDP	-4.9907327	3.07052035	0	NA	NDP	NDP
NOG	-3.1842631	2.05302024	0	NA	NOG	NOG
NR3C1	3.9886444	0.55590161	0	NA	NR3C1	NR3C1
NR3C2	6.2948111	0.35436521	0	NA	NR3C2	NR3C2
NR4A2	5.3728796	0.31034033	0	NA	NR4A2	NR4A2
NTNG2	5.0387291	0.27228346	0	NA	NTNG2	NTNG2
NXPH3	3.2378358	0.63766383	0	NA	NXPH3	NXPH3
ODZ4	2.4831271	0.71491135	0	NA	ODZ4	ODZ4
OLFM3	-2.3427178	1.60992788	0	NA	OLFM3	OLFM3
PBX3	2.6304097	0.71030199	0	NA	PBX3	PBX3
PCDH17	-5.0671224	2.34152922	0	NA	PCDH17	PCDH17
PCDH18	-2.4106968	1.50798322	0	NA	PCDH18	PCDH18
PCSK9	2.8585448	0.66795293	0	NA	PCSK9	PCSK9
PDE4DIP	2.650362	0.69291976	0	NA	PDE4DIP	PDE4DIP
PDZRN4	-5.4476486	2.63707924	0	NA	PDZRN4	PDZRN4
PHLDA1	-4.0501691	1.65922624	0	NA	PHLDA1	PHLDA1
PIK3AP1	2.7624911	0.64434479	0	NA	PIK3AP1	PIK3AP1
PPARGC1A	2.5259817	0.69605157	0	NA	PPARGC1A	PPARGC1A
PSD2	-2.1802477	1.30380301	0	NA	PSD2	PSD2
PTER	-3.937988	2.21395294	0	NA	PTER	PTER
PTPRK	4.7632103	0.3977537	0	NA	PTPRK	PTPRK
QDPR	-2.1979566	1.33072736	0	NA	QDPR	QDPR
RAB7L1	3.8609759	0.56498331	0	NA	RAB7L1	RAB7L1
RGS20	2.36348	0.71130303	0	NA	RGS20	RGS20
RNF182	4.7106408	0.47962861	0	NA	RNF182	RNF182
ROBO2	5.3895655	0.46474463	0	NA	ROBO2	ROBO2
RPRM	-3.0311581	2.03229418	0	NA	RPRM	RPRM
SFRP1	-3.1715505	1.60973547	0	NA	SFRP1	SFRP1
SFRP2	-2.9745118	1.69062342	0	NA	SFRP2	SFRP2
SLC6A15	2.7910928	0.67529137	0	NA	SLC6A15	SLC6A15
SLCO3A1	-2.3528977	1.73020418	0	NA	SLCO3A1	SLCO3A1
SLIT2	-3.0879381	1.56586266	0	NA	SLIT2	SLIT2
SORCS1	4.3988439	0.48451882	0	NA	SORCS1	SORCS1
SPON1	5.0932677	0.30520412	0	NA	SPON1	SPON1
ST8SIA3	-2.4241874	1.47965344	0	NA	ST8SIA3	ST8SIA3
STXBP6	-7.5596973	3.29939452	0	NA	STXBP6	STXBP6
SULF2	-4.7353515	2.57250946	0	NA	SULF2	SULF2
SYNE1	2.7432808	0.71460998	0	NA	SYNE1	SYNE1
SYNM	-3.2827036	1.63034073	0	NA	SYNM	SYNM
TLE1	9.2333799	0.33331922	0	NA	TLE1	TLE1
TMCC2	2.363984	0.72802771	0	NA	TMCC2	TMCC2
TOX	-6.2470255	3.16050207	0	NA	TOX	TOX
TPBG	-2.3870222	1.57377889	0	NA	TPBG	TPBG
TRH	-4.3485877	1.92544255	0	NA	TRH	TRH
WBSCR17	-3.4505242	1.54274478	0	NA	WBSCR17	WBSCR17
WNT11	-2.185204	1.57874503	0	NA	WNT11	WNT11
ZNF238	2.8390867	0.596913	0	NA	ZNF238	ZNF238
ZNF423	-6.9022481	4.59746511	0	NA	ZNF423	ZNF423
C5orf4	2.0449505	0.75038304	0.11329523	NA	C5orf4	C5orf4
DPF3	-1.9948572	1.32280327	0.11329523	NA	DPF3	DPF3
EIF3E	2.1041583	0.79564492	0.11329523	NA	EIF3E	EIF3E
GGTA1	-2.1053034	1.54639322	0.11329523	NA	GGTA1	GGTA1
GOLGA7B	-2.1550231	1.44318056	0.11329523	NA	GOLGA7B	GOLGA7B
GPAM	2.140471	0.68699697	0.11329523	NA	GPAM	GPAM
GRIK2	-2.1000341	1.32784063	0.11329523	NA	GRIK2	GRIK2
GRM7	-1.9945815	1.33335681	0.11329523	NA	GRM7	GRM7
HSPB3	-2.111997	1.51116191	0.11329523	NA	HSPB3	HSPB3
HTR7	2.0742972	0.71424476	0.11329523	NA	HTR7	HTR7

ASAM	-2.1306034	1.62637404	0.11329523	NA	ASAM	NA
C13orf18	2.0554786	0.66207045	0.11329523	NA	C13orf18	NA
PLP2	2.1639678	0.71837011	0.11329523	NA	PLP2	NA
SATB2	2.0138674	0.6652304	0.11329523	NA	SATB2	SATB2
SH3BP4	-2.1323932	1.35398485	0.11329523	NA	SH3BP4	SH3BP4
TARBP1	2.0160223	0.77167533	0.11329523	NA	TARBP1	TARBP1
TBX21	-2.0951755	1.50634971	0.11329523	NA	TBX21	TBX21
ACVR1	1.9016813	0.74909317	0.19206239	NA	ACVR1	ACVR1
AKAP13	1.8672226	0.77684803	0.19206239	NA	AKAP13	AKAP13
ASS1	-1.8757098	1.36348358	0.19206239	NA	ASS1	ASS1
C9orf45	1.9496523	0.75910403	0.19206239	NA	C9orf45	C9orf45
CYYR1	-1.8344824	1.39509513	0.19206239	NA	CYYR1	CYYR1
DPP3	-1.8794705	1.2852484	0.19206239	NA	DPP3	DPP3
DPYD	1.91158	0.75716218	0.19206239	NA	DPYD	DPYD
HS3ST2	-1.9807592	1.51306092	0.19206239	NA	HS3ST2	HS3ST2
KLHL14	-1.8384008	1.35188201	0.19206239	NA	KLHL14	KLHL14
CCDC110	1.8985224	0.67066792	0.19206239	NA	CCDC110	NA
KLHL21	1.8221708	0.7030475	0.19206239	NA	KLHL21	NA
LOC151009	1.9328236	0.70324145	0.19206239	NA	LOC151009	NA
NGEF	1.9272593	0.79921309	0.19206239	NA	NGEF	NA
RLIM	1.8984403	0.79797798	0.19206239	NA	RLIM	NA
SLC22A15	-1.8528495	1.3029058	0.19206239	NA	SLC22A15	NA
TMEM111	1.8818937	0.74576381	0.19206239	NA	TMEM111	NA
ZBTB44	1.92715	0.78897914	0.19206239	NA	ZBTB44	NA
NOS1	-1.9311619	1.2985339	0.19206239	NA	NOS1	NOS1
PLOD2	1.8199163	0.63535707	0.19206239	NA	PLOD2	PLOD2
RSL24D1	1.9607116	0.76287592	0.19206239	NA	RSL24D1	RSL24D1
SNX9	1.8759538	0.72844561	0.19206239	NA	SNX9	SNX9
ZADH2	-1.9388644	1.33605933	0.19206239	NA	ZADH2	ZADH2
ZNF706	-1.8489992	1.22405684	0.19206239	NA	ZNF706	ZNF706
AMOTL1	-1.6708045	1.2233893	0.56921718	NA	AMOTL1	AMOTL1
C1orf201	1.6867839	0.76162761	0.56921718	NA	C1orf201	C1orf201
CHGB	1.6889775	0.80786526	0.56921718	NA	CHGB	CHGB
DIAPH3	-1.691133	1.316908	0.56921718	NA	DIAPH3	DIAPH3
DUSP6	-1.6648534	1.34244361	0.56921718	NA	DUSP6	DUSP6
FBXL4	1.7030863	0.74322208	0.56921718	NA	FBXL4	FBXL4
FOSL2	-1.8048882	1.39235042	0.56921718	NA	FOSL2	FOSL2
GRM3	1.7963915	0.79517321	0.56921718	NA	GRM3	GRM3
ID4	-1.714183	1.24941014	0.56921718	NA	ID4	ID4
LRRC4C	1.7723172	0.78380855	0.56921718	NA	LRRC4C	LRRC4C
MAOA	-1.7230175	1.26439033	0.56921718	NA	MAOA	MAOA
MIAT	1.6685052	0.7468721	0.56921718	NA	MIAT	MIAT
CIDEA	1.6688155	0.73169435	0.56921718	NA	CIDEA	NA
MXRA7	-1.7854284	1.28071002	0.56921718	NA	MXRA7	NA
NINJ1	1.6946936	0.79883026	0.56921718	NA	NINJ1	NA
TMIGD2	1.7168483	0.7748928	0.56921718	NA	TMIGD2	NA
NGB	1.6973064	0.81316304	0.56921718	NA	NGB	NGB
NR2F2	1.6810585	0.78707751	0.56921718	NA	NR2F2	NR2F2
NRXN3	-1.6779785	1.23251032	0.56921718	NA	NRXN3	NRXN3
PLXNC1	-1.7689523	1.29644548	0.56921718	NA	PLXNC1	PLXNC1
RAPGEFL1	1.7176713	0.71074745	0.56921718	NA	RAPGEFL1	RAPGEFL1
RUNX2	-1.6858028	1.28467826	0.56921718	NA	RUNX2	RUNX2
SMAD3	1.7388772	0.78593321	0.56921718	NA	SMAD3	SMAD3
ZAK	1.670217	0.71519869	0.56921718	NA	ZAK	ZAK
ZHX2	1.7521996	0.76600304	0.56921718	NA	ZHX2	ZHX2
AUH	1.5088174	0.79367185	0.88419955	NA	AUH	AUH
CDH13	1.5766612	0.71012446	0.88419955	NA	CDH13	CDH13
CLCN4	-1.6496394	1.35400775	0.88419955	NA	CLCN4	CLCN4

DCC	-1.5468003	1.25641969	0.88419955	NA	DCC	DCC
DMXL1	1.5936059	0.79464748	0.88419955	NA	DMXL1	DMXL1
DNMT3A	-1.6393412	1.26259462	0.88419955	NA	DNMT3A	DNMT3A
KBTBD8	1.5720203	0.79932808	0.88419955	NA	KBTBD8	KBTBD8
KIAA1958	-1.5262747	1.26307324	0.88419955	NA	KIAA1958	KIAA1958
MARK1	1.5520661	0.80040881	0.88419955	NA	MARK1	MARK1
MDH1	1.6458122	0.7827652	0.88419955	NA	MDH1	MDH1
MITF	1.626352	0.7826945	0.88419955	NA	MITF	MITF
MYO1B	-1.6535742	1.26905342	0.88419955	NA	MYO1B	MYO1B
CPLX3	1.6128539	0.80616955	0.88419955	NA	CPLX3	NA
CRIPAK	1.5992703	0.7591401	0.88419955	NA	CRIPAK	NA
PREB	-1.5009519	1.19427223	0.88419955	NA	PREB	NA
PSRC1	-1.6109387	1.34653818	0.88419955	NA	PSRC1	NA
SCO2	-1.6514943	1.26470566	0.88419955	NA	SCO2	NA
SEPX1	-1.5896316	1.26272394	0.88419955	NA	SEPX1	NA
TP53BP2	1.5084482	0.80778045	0.88419955	NA	TP53BP2	NA
PRKCB	1.623504	0.7653644	0.88419955	NA	PRKCB	PRKCB
SH3KBP1	1.6217678	0.81439028	0.88419955	NA	SH3KBP1	SH3KBP1
SMAD7	1.5822772	0.77254442	0.88419955	NA	SMAD7	SMAD7
SPRED2	-1.5160014	1.21935135	0.88419955	NA	SPRED2	SPRED2
STAG2	1.5384991	0.80153576	0.88419955	NA	STAG2	STAG2
SYT13	1.5262234	0.77492133	0.88419955	NA	SYT13	SYT13
TDP1	-1.6031528	1.24968789	0.88419955	NA	TDP1	TDP1
TMEM47	-1.6560851	1.2938101	0.88419955	NA	TMEM47	TMEM47
TMEM5	-1.5773597	1.27996903	0.88419955	NA	TMEM5	TMEM5
ZNF395	-1.598808	1.21514445	0.88419955	NA	ZNF395	ZNF395

CAR only PFOvsPT q<0.01

SYMBOL	PFOvsPT.d	PFOvsPT.FC	PFOvsPT.q	HAR	CAR	MAR
ARHGDIB	-2.6187164	1.37947451	0	NA	ARHGDIB	NA
CACNA1A	-5.7244908	2.02037437	0	NA	CACNA1A	NA
CNKSR3	-5.9495054	2.9706848	0	NA	CNKSR3	NA
CYP27C1	-4.0171215	2.88173718	0	NA	CYP27C1	NA
DCBLD1	2.2063284	0.71430327	0	NA	DCBLD1	NA
DNAH10	-2.8406866	1.5059201	0	NA	DNAH10	NA
FSIP1	3.2066337	0.54366788	0	NA	FSIP1	NA
MEIS3P1	-4.0994783	3.16136077	0	NA	MEIS3P1	NA
PKIG	-2.4710853	1.51923202	0	NA	PKIG	NA
PLAGL1	2.7294518	0.62240269	0	NA	PLAGL1	NA
SLC16A1	3.2925757	0.59571496	0	NA	SLC16A1	NA
TNFAIP8	2.5452211	0.57765687	0	NA	TNFAIP8	NA
ASAM	-2.1306034	1.62637404	0.11329523	NA	ASAM	NA
C13orf18	2.0554786	0.66207045	0.11329523	NA	C13orf18	NA
PLP2	2.1639678	0.71837011	0.11329523	NA	PLP2	NA
CCDC110	1.8985224	0.67066792	0.19206239	NA	CCDC110	NA
KLHL21	1.8221708	0.7030475	0.19206239	NA	KLHL21	NA
LOC151009	1.9328236	0.70324145	0.19206239	NA	LOC151009	NA
NGEF	1.9272593	0.79921309	0.19206239	NA	NGEF	NA
RLIM	1.8984403	0.79797798	0.19206239	NA	RLIM	NA
SLC22A15	-1.8528495	1.3029058	0.19206239	NA	SLC22A15	NA
TMEM111	1.8818937	0.74576381	0.19206239	NA	TMEM111	NA
ZBTB44	1.92715	0.78897914	0.19206239	NA	ZBTB44	NA
CIDEA	1.6688155	0.73169435	0.56921718	NA	CIDEA	NA

MXRA7	-1.7854284	1.28071002	0.56921718	NA	MXRA7	NA
NINJ1	1.6946936	0.79883026	0.56921718	NA	NINJ1	NA
TMIGD2	1.7168483	0.7748928	0.56921718	NA	TMIGD2	NA
CPLX3	1.6128539	0.80616955	0.88419955	NA	CPLX3	NA
CRIPAK	1.5992703	0.7591401	0.88419955	NA	CRIPAK	NA
PREB	-1.5009519	1.19427223	0.88419955	NA	PREB	NA
PSRC1	-1.6109387	1.34653818	0.88419955	NA	PSRC1	NA
SCO2	-1.6514943	1.26470566	0.88419955	NA	SCO2	NA
SEPX1	-1.5896316	1.26272394	0.88419955	NA	SEPX1	NA
TP53BP2	1.5084482	0.80778045	0.88419955	NA	TP53BP2	NA

MAR PFOvsPT q<0.01

SYMBOL	PFOvsPT.d	PFOvsPT.FC	PFOvsPT.q	HAR	CAR	MAR
ADCYAP1	-3.0970772	1.61592882	0	ADCYAP1	NA	ADCYAP1
ADRA2A	-2.2026569	1.32088762	0	ADRA2A	ADRA2A	ADRA2A
ADRB1	3.2483725	0.62843359	0	ADRB1	NA	ADRB1
AKT3	5.2245195	0.38230327	0	AKT3	AKT3	AKT3
ASTN2	-3.537837	1.8451722	0	ASTN2	ASTN2	ASTN2
BCL11A	2.8875415	0.57742488	0	BCL11A	BCL11A	BCL11A
BMP2	-4.2137159	2.61468581	0	BMP2	BMP2	BMP2
C4orf32	2.3831704	0.76084728	0	C4orf32	C4orf32	C4orf32
CA10	-6.12	5.71221273	0	CA10	CA10	CA10
CBLN4	-2.6478539	1.61876179	0	CBLN4	NA	CBLN4
CDH8	-6.0646864	4.185182	0	CDH8	CDH8	CDH8
CNTN4	-4.2956365	2.05345693	0	CNTN4	CNTN4	CNTN4
CNTN6	-2.8124791	1.62508137	0	CNTN6	CNTN6	CNTN6
CTNNA2	2.3498011	0.72677424	0	CTNNA2	CTNNA2	CTNNA2
CYTIP	2.420378	0.61081395	0	CYTIP	NA	CYTIP
DPP10	2.3546219	0.6215833	0	DPP10	DPP10	DPP10
EGLN3	-2.9967726	1.72101012	0	EGLN3	EGLN3	EGLN3
FAM181B	-2.4483969	1.44723701	0	FAM181B	FAM181B	FAM181B
FAM19A1	4.3485528	0.48800542	0	FAM19A1	NA	FAM19A1
FAM5C	-6.2733541	4.38293974	0	FAM5C	FAM5C	FAM5C
FLJ13197	3.651636	0.51804829	0	FLJ13197	NA	FLJ13197
FST	2.4569874	0.57303	0	FST	FST	FST
GBE1	-3.791506	1.82357854	0	GBE1	GBE1	GBE1
GFRA2	-3.4819788	2.03486324	0	GFRA2	GFRA2	GFRA2
GPR64	4.0661047	0.33444216	0	GPR64	NA	GPR64
GSTA4	3.1060179	0.61447252	0	GSTA4	NA	GSTA4
HS3ST5	-2.6831055	1.77210432	0	HS3ST5	NA	HS3ST5
INHBA	3.8503447	0.53577238	0	INHBA	NA	INHBA
ITGBL1	-2.8160076	1.50576473	0	ITGBL1	NA	ITGBL1
KCNQ5	-3.4225801	1.81548163	0	KCNQ5	NA	KCNQ5
KCNV1	-2.3638489	1.62181689	0	KCNV1	KCNV1	KCNV1
KIAA1797	2.2970172	0.70104402	0	KIAA1797	NA	KIAA1797
L3MBTL4	-7.3135318	2.8540538	0	L3MBTL4	NA	L3MBTL4
LHX2	5.766844	0.4639514	0	LHX2	LHX2	LHX2
LPHN2	4.2270257	0.47033437	0	LPHN2	LPHN2	LPHN2
LRRC8B	-2.8892578	1.4786014	0	LRRC8B	LRRC8B	LRRC8B
LRRN3	3.7923133	0.62994277	0	LRRN3	NA	LRRN3
LYPD1	-2.40434	1.63547339	0	LYPD1	NA	LYPD1
MEIS2	-8.0564215	3.20343535	0	MEIS2	MEIS2	MEIS2
MID1IP1	-3.3184109	1.50260718	0	MID1IP1	MID1IP1	MID1IP1
MN1	-2.3740917	1.37684425	0	MN1	NA	MN1

AADAT	-2.5028615	1.32458137	0	NA	NA	AADAT
ABCA1	-3.0361926	1.58268171	0	NA	NA	ABCA1
ACCN1	-2.6307648	1.67979598	0	NA	ACCN1	ACCN1
ACTN2	5.7230026	0.4636697	0	NA	NA	ACTN2
ACVR1C	4.1708629	0.43979572	0	NA	NA	ACVR1C
ADAMTS16	-2.3622011	1.76005451	0	NA	ADAMTS16	ADAMTS16
ADAMTS3	-2.4132743	1.48483016	0	NA	NA	ADAMTS3
ADCY8	-7.3811571	3.68678064	0	NA	NA	ADCY8
ADI1	2.2650305	0.75310026	0	NA	NA	ADI1
AK3L1	-2.7061578	1.72460433	0	NA	NA	AK3L1
AK5	2.4672315	0.67898931	0	NA	AK5	AK5
ANKH	2.6719111	0.68214739	0	NA	NA	ANKH
ANKRD32	-5.2441495	2.29550366	0	NA	ANKRD32	ANKRD32
ANKRD57	3.0675827	0.64815372	0	NA	NA	ANKRD57
ANKS1B	5.4887345	0.46381882	0	NA	NA	ANKS1B
ANO3	2.3646069	0.48056189	0	NA	NA	ANO3
ANO4	-3.3417516	1.52520784	0	NA	NA	ANO4
ANTXR2	3.9176954	0.58369719	0	NA	NA	ANTXR2
APOO	-2.2526913	1.32642345	0	NA	APOO	APOO
ARHGAP6	-3.195736	1.87980994	0	NA	NA	ARHGAP6
ARHGEF6	2.8277758	0.64287333	0	NA	NA	ARHGEF6
ARL4A	2.511116	0.63345231	0	NA	NA	ARL4A
ARNTL	2.5357596	0.65285149	0	NA	ARNTL	ARNTL
ASAH1	2.5440324	0.73396012	0	NA	NA	ASAH1
ASCL1	4.3720168	0.50310044	0	NA	NA	ASCL1
ATP8A2	-2.771165	1.51469201	0	NA	NA	ATP8A2
B3GALT2	3.1860665	0.64355277	0	NA	NA	B3GALT2
B3GAT2	-2.2564059	1.50607282	0	NA	NA	B3GAT2
B3GNT2	-2.2363781	1.33150078	0	NA	NA	B3GNT2
BAMBI	-2.5486832	1.74619574	0	NA	NA	BAMBI
BARD1	3.8839376	0.39442453	0	NA	NA	BARD1
BATF3	2.2609024	0.66619292	0	NA	NA	BATF3
BDNF	-6.0284835	4.73059426	0	NA	NA	BDNF
BEND6	2.719765	0.6260379	0	NA	NA	BEND6
BHLHE40	2.4061711	0.67549028	0	NA	NA	BHLHE40
C14orf132	4.9873905	0.49423025	0	NA	NA	C14orf132
C16orf61	-4.4408933	1.96458039	0	NA	NA	C16orf61
C18orf1	2.6272125	0.72658286	0	NA	NA	C18orf1
C1orf198	-2.5560951	1.44208311	0	NA	NA	C1orf198
C3orf59	-3.6667364	1.71843955	0	NA	NA	C3orf59
C6orf141	-2.511954	1.54599037	0	NA	NA	C6orf141
CACNA2D3	-5.0183639	2.37311616	0	NA	CACNA2D3	CACNA2D3
CALB1	-10.470768	11.6676061	0	NA	NA	CALB1
CAP2	2.3484789	0.74445997	0	NA	NA	CAP2
CBLN1	-7.7041147	4.80719481	0	NA	CBLN1	CBLN1
CBLN2	-14.554868	11.2565275	0	NA	NA	CBLN2
CCDC109A	-2.989666	1.63539425	0	NA	CCDC109A	CCDC109A
CCDC109B	3.0258479	0.54338818	0	NA	NA	CCDC109B
CDCA7	2.3297026	0.73081932	0	NA	NA	CDCA7
CDCA7L	-3.204917	1.82154463	0	NA	NA	CDCA7L
CDH12	-3.0931634	1.70193601	0	NA	CDH12	CDH12
CDH3	2.8363774	0.6615742	0	NA	NA	CDH3
CDH9	3.3472538	0.50238639	0	NA	CDH9	CDH9
CENPF	-2.9824191	1.57276807	0	NA	NA	CENPF
CEP120	-2.8706623	1.55083986	0	NA	CEP120	CEP120
CGRRF1	-4.1255188	2.23860247	0	NA	CGRRF1	CGRRF1
CHRDL1	-4.5280955	2.8838507	0	NA	NA	CHRDL1
CHST8	2.9652964	0.48824292	0	NA	NA	CHST8

CLDN1	-6.6031156	2.44678317	0	NA	NA	CLDN1
CNIH3	-3.9901337	1.71872752	0	NA	NA	CNIH3
CNTN3	-5.1226552	2.1416389	0	NA	CNTN3	CNTN3
CNTNAP2	-5.1222137	3.34073761	0	NA	NA	CNTNAP2
COL12A1	-2.5600556	1.45817607	0	NA	NA	COL12A1
COMMD2	2.6738365	0.72015488	0	NA	NA	COMMD2
COX7A1	3.6220653	0.49840726	0	NA	COX7A1	COX7A1
CPNE8	-7.9019211	5.45090012	0	NA	CPNE8	CPNE8
CRISPLD1	-2.8583041	1.65098592	0	NA	NA	CRISPLD1
CSRP2	-5.7836768	2.30538505	0	NA	NA	CSRP2
CTNNBL1	-5.1701204	2.60508807	0	NA	CTNNBL1	CTNNBL1
CTXN3	-3.1547723	2.02249669	0	NA	CTXN3	CTXN3
CUX1	8.0750155	0.33967862	0	NA	NA	CUX1
CXCR4	2.4724394	0.73913062	0	NA	NA	CXCR4
CXorf1	3.5295083	0.5214482	0	NA	CXorf1	CXorf1
CXXC4	-3.9002226	1.68344979	0	NA	NA	CXXC4
CXXC5	-5.7185821	2.53493779	0	NA	NA	CXXC5
CYP26A1	-2.9104637	2.07047558	0	NA	NA	CYP26A1
DACH2	5.3983733	0.39389626	0	NA	DACH2	DACH2
DACT1	3.7612088	0.6514341	0	NA	DACT1	DACT1
DERA	-3.2478245	1.90395366	0	NA	NA	DERA
DGKI	2.8821568	0.44509385	0	NA	DGKI	DGKI
DLG1	3.1684757	0.60089689	0	NA	DLG1	DLG1
DNER	2.2646872	0.67573729	0	NA	NA	DNER
DOK5	3.4689382	0.68277451	0	NA	DOK5	DOK5
DUSP10	-4.7462373	1.89223447	0	NA	NA	DUSP10
DUSP4	-4.5331275	2.38044825	0	NA	NA	DUSP4
DYRK2	2.3302515	0.68671835	0	NA	DYRK2	DYRK2
EDIL3	2.4718718	0.71487974	0	NA	NA	EDIL3
EFR3A	-2.5473489	1.48864717	0	NA	NA	EFR3A
EMX2	-3.67376	2.37966598	0	NA	NA	EMX2
EPCAM	3.3607583	0.567492	0	NA	NA	EPCAM
EPHA4	-2.3288721	1.52144273	0	NA	EPHA4	EPHA4
EPHA7	2.3254158	0.64339145	0	NA	NA	EPHA7
EPS15	-2.380353	1.48687855	0	NA	NA	EPS15
F3	-2.1747337	1.73215267	0	NA	NA	F3
FAM19A5	-2.2537914	1.36434919	0	NA	FAM19A5	FAM19A5
FAM59A	-2.4934649	1.78462466	0	NA	NA	FAM59A
FAM5B	-2.7524322	1.60087371	0	NA	FAM5B	FAM5B
FERMT2	4.2632045	0.48073451	0	NA	NA	FERMT2
FGF12	-3.1985688	1.60028385	0	NA	FGF12	FGF12
FGF14	-4.161336	1.89013512	0	NA	FGF14	FGF14
FGFBP3	5.9094274	0.39665838	0	NA	NA	FGFBP3
FHDC1	-6.7100988	2.52361319	0	NA	NA	FHDC1
FJX1	5.5721969	0.39617966	0	NA	NA	FJX1
FLJ11235	2.3242112	0.67035538	0	NA	NA	FLJ11235
FLJ43390	-9.7499031	5.56194442	0	NA	FLJ43390	FLJ43390
FLRT3	-6.0329061	2.31581298	0	NA	FLRT3	FLRT3
FOXP1	2.5735375	0.71489822	0	NA	NA	FOXP1
FRMPD4	2.6700364	0.72369361	0	NA	FRMPD4	FRMPD4
FRZB	2.8764855	0.66639767	0	NA	NA	FRZB
FSTL5	-9.134059	5.14415268	0	NA	NA	FSTL5
FZD4	-2.5504763	1.47882771	0	NA	NA	FZD4
GAB1	2.4348477	0.72772467	0	NA	NA	GAB1
GABRA1	-2.4861275	1.5458172	0	NA	GABRA1	GABRA1
GABRA5	4.1635031	0.57212962	0	NA	GABRA5	GABRA5
GABRG1	3.8878003	0.50753413	0	NA	NA	GABRG1
GABRG2	-2.500529	1.35616898	0	NA	GABRG2	GABRG2

GALNT2	-4.2925877	1.95474348	0	NA	NA	GALNT2
GAS1	-2.8207017	1.43925682	0	NA	GAS1	GAS1
GCG	-4.5083529	2.3913353	0	NA	NA	GCG
GHR	2.8771158	0.43728712	0	NA	NA	GHR
GLT25D2	-3.7023891	1.66361917	0	NA	NA	GLT25D2
GOLSYN	3.3184939	0.65282491	0	NA	GOLSYN	GOLSYN
GPHN	2.5740778	0.72677534	0	NA	NA	GPHN
GPR22	3.3248308	0.47835698	0	NA	NA	GPR22
GPR37	-2.8484793	1.64943831	0	NA	NA	GPR37
GPR88	-3.4255545	2.18844425	0	NA	NA	GPR88
GPSM2	-3.4162766	2.69405811	0	NA	NA	GPSM2
GRB14	2.5728727	0.49303609	0	NA	GRB14	GRB14
GREM1	-2.8864642	2.73409001	0	NA	NA	GREM1
GREM2	-4.9255321	4.01956006	0	NA	NA	GREM2
H2AFY2	-2.909317	1.40106706	0	NA	H2AFY2	H2AFY2
HCRTR2	-2.6419059	1.7646649	0	NA	NA	HCRTR2
HEY1	-3.7913367	1.48994671	0	NA	NA	HEY1
HIP1R	-2.1829824	1.28957466	0	NA	NA	HIP1R
HLF	4.0234794	0.4754812	0	NA	HLF	HLF
HS3ST1	-2.2006599	1.42280822	0	NA	NA	HS3ST1
HS6ST3	-3.6617101	2.5215554	0	NA	NA	HS6ST3
HSPA12A	-4.2211371	2.06573928	0	NA	NA	HSPA12A
HTR1A	3.8667755	0.55382899	0	NA	NA	HTR1A
HUNK	-3.5936681	1.62480292	0	NA	NA	HUNK
ID2	3.8786506	0.44944894	0	NA	ID2	ID2
IGFBP4	2.3828134	0.68418134	0	NA	IGFBP4	IGFBP4
IGFBP5	-2.834741	1.61843461	0	NA	IGFBP5	IGFBP5
IGSF11	-7.5561874	3.09731621	0	NA	IGSF11	IGSF11
IL7	-4.0113273	3.62954247	0	NA	NA	IL7
INTS12	-2.4877879	1.36118535	0	NA	NA	INTS12
IRS2	-3.4481015	1.49754195	0	NA	NA	IRS2
ITGA2	2.7273643	0.64873872	0	NA	NA	ITGA2
ITGA9	-2.8155154	1.48817905	0	NA	NA	ITGA9
ITGAV	2.574707	0.67811483	0	NA	NA	ITGAV
ITPR1	3.6500924	0.6397888	0	NA	NA	ITPR1
JMJD1C	2.5771635	0.67697228	0	NA	NA	JMJD1C
JPH1	-3.2960068	1.56100122	0	NA	NA	JPH1
KCNA5	-5.2899544	2.51664648	0	NA	NA	KCNA5
KCNC2	-2.9617191	1.43705499	0	NA	NA	KCNC2
KCNJ6	-5.6306064	2.58105401	0	NA	NA	KCNJ6
KCNK9	-3.5810556	1.81798244	0	NA	NA	KCNK9
KCNT2	2.3088159	0.53576416	0	NA	NA	KCNT2
KIAA0182	2.8095351	0.6575132	0	NA	KIAA0182	KIAA0182
KIAA0319	3.0140926	0.65502347	0	NA	NA	KIAA0319
KIAA0895	4.4775793	0.41370822	0	NA	NA	KIAA0895
KIAA1239	3.8815334	0.58276574	0	NA	NA	KIAA1239
KIAA1377	-5.0111275	2.24041945	0	NA	NA	KIAA1377
KIAA1804	3.1060298	0.62349667	0	NA	NA	KIAA1804
KIT	-3.1769796	1.63754001	0	NA	NA	KIT
KITLG	2.7996099	0.49657375	0	NA	NA	KITLG
KL	2.5219389	0.67779547	0	NA	NA	KL
KLF6	-2.7404503	1.42932138	0	NA	KLF6	KLF6
KLHL32	5.6726516	0.24577443	0	NA	NA	KLHL32
KLHL8	3.0656515	0.69557702	0	NA	NA	KLHL8
KRT31	-3.8344871	2.18590383	0	NA	NA	KRT31
LDB2	-2.8931194	1.4399603	0	NA	NA	LDB2
LDLRAD3	4.0334391	0.51008571	0	NA	NA	LDLRAD3
LGALS8	2.2259948	0.72480252	0	NA	LGALS8	LGALS8

LHFP	3.3336246	0.60532507	0	NA	LHFP	LHFP
LHFPL2	-4.0089999	1.78865557	0	NA	NA	LHFPL2
LMO3	-2.4423344	1.621965	0	NA	LMO3	LMO3
LMO4	-5.4333042	2.09762329	0	NA	LMO4	LMO4
LNX2	-2.7594468	1.56511014	0	NA	NA	LNX2
LOC150568	-4.5808282	2.16132196	0	NA	LOC150568	LOC150568
LOC283392	3.2109194	0.58737246	0	NA	LOC283392	LOC283392
LOC286002	6.6399202	0.37730405	0	NA	NA	LOC286002
LOC645323	2.2641538	0.74917028	0	NA	LOC645323	LOC645323
LOC653786	2.4826133	0.66960833	0	NA	LOC653786	LOC653786
LOX	4.6566289	0.52134261	0	NA	NA	LOX
LPL	2.7815804	0.71377351	0	NA	NA	LPL
LRRC3B	-4.8019253	1.97409064	0	NA	NA	LRRC3B
LRRTM2	-2.4679681	1.4276446	0	NA	NA	LRRTM2
LRRTM4	5.9239675	0.23561301	0	NA	LRRTM4	LRRTM4
LUZP2	2.5089308	0.46101732	0	NA	NA	LUZP2
MAL2	-2.6502091	1.76772617	0	NA	NA	MAL2
MAMLD1	-2.732106	1.53315954	0	NA	MAMLD1	MAMLD1
MAP3K5	-6.2555867	3.27948624	0	NA	NA	MAP3K5
MARCH3	-5.561013	1.98423491	0	NA	MARCH3	MARCH3
MAST4	-4.5965819	1.89897414	0	NA	MAST4	MAST4
MC4R	-2.7448307	2.07739068	0	NA	MC4R	MC4R
MCHR2	-3.564827	1.90786707	0	NA	NA	MCHR2
MCTP1	3.6104371	0.58045841	0	NA	NA	MCTP1
MDGA1	-2.3368477	1.47049165	0	NA	MDGA1	MDGA1
ME3	-2.3898726	1.31149488	0	NA	NA	ME3
MEGF10	-7.2306862	3.23291631	0	NA	NA	MEGF10
MEGF11	-2.9538663	1.677664	0	NA	NA	MEGF11
MET	3.6942663	0.56200753	0	NA	NA	MET
MID1	3.3365625	0.56004233	0	NA	MID1	MID1
MIPEP	-6.0186078	2.26883941	0	NA	NA	MIPEP
MOCS2	2.6924114	0.68164569	0	NA	NA	MOCS2
MOSC1	-2.4501897	1.4052336	0	NA	MOSC1	MOSC1
MPP6	2.8271169	0.45577482	0	NA	NA	MPP6
MRAP2	2.3949268	0.51337438	0	NA	NA	MRAP2
MTFR1	2.241794	0.74725754	0	NA	NA	MTFR1
MYO16	-3.0542861	1.60213646	0	NA	NA	MYO16
MYO1E	2.4481187	0.67850105	0	NA	NA	MYO1E
NCOA2	2.3244361	0.65543156	0	NA	NA	NCOA2
NDP	-4.9907327	3.07052035	0	NA	NDP	NDP
NEBL	-3.959623	1.81477552	0	NA	NA	NEBL
NEDD9	-2.4947402	1.5534365	0	NA	NA	NEDD9
NEFL	6.1790036	0.31463525	0	NA	NA	NEFL
NEUROD1	-7.5610329	4.91363672	0	NA	NA	NEUROD1
NFE2L3	3.2905816	0.45771202	0	NA	NA	NFE2L3
NME5	-2.2837135	1.57292326	0	NA	NA	NME5
NOG	-3.1842631	2.05302024	0	NA	NOG	NOG
NPAS2	2.4130014	0.7114197	0	NA	NA	NPAS2
NPY	10.487701	0.15260943	0	NA	NA	NPY
NPY1R	-2.8091789	1.55164331	0	NA	NA	NPY1R
NR2F1	4.5333847	0.4904898	0	NA	NA	NR2F1
NR3C1	3.9886444	0.55590161	0	NA	NR3C1	NR3C1
NR3C2	6.2948111	0.35436521	0	NA	NR3C2	NR3C2
NR4A2	5.3728796	0.31034033	0	NA	NR4A2	NR4A2
NRIP1	-3.2856362	1.71512376	0	NA	NA	NRIP1
NRN1	2.645197	0.61308738	0	NA	NA	NRN1
NTN4	-4.3353016	2.12969222	0	NA	NA	NTN4
NTNG1	2.5601591	0.6424647	0	NA	NA	NTNG1

NTNG2	5.0387291	0.27228346	0	NA	NTNG2	NTNG2
NUP93	-3.4552735	1.54554115	0	NA	NA	NUP93
NXPH3	3.2378358	0.63766383	0	NA	NXPH3	NXPH3
ODZ4	2.4831271	0.71491135	0	NA	ODZ4	ODZ4
OLFM3	-2.3427178	1.60992788	0	NA	OLFM3	OLFM3
OSBPL6	2.5945569	0.64613791	0	NA	NA	OSBPL6
OTOR	3.3941304	0.32209121	0	NA	NA	OTOR
P2RY12	2.5446371	0.62162186	0	NA	NA	P2RY12
PACSIN2	-2.7839704	1.44877693	0	NA	NA	PACSIN2
PAICS	3.8589303	0.6145106	0	NA	NA	PAICS
PAK7	4.4807926	0.58162497	0	NA	NA	PAK7
PALMD	-3.1556924	1.82573813	0	NA	NA	PALMD
PAPPA2	3.3547903	0.67821402	0	NA	NA	PAPPA2
PBX3	2.6304097	0.71030199	0	NA	PBX3	PBX3
PCBD1	-2.4006729	1.43434157	0	NA	NA	PCBD1
PCDH17	-5.0671224	2.34152922	0	NA	PCDH17	PCDH17
PCDH18	-2.4106968	1.50798322	0	NA	PCDH18	PCDH18
PCOLCE2	2.5204469	0.58040551	0	NA	NA	PCOLCE2
PCSK2	11.205976	0.12589846	0	NA	NA	PCSK2
PCSK9	2.8585448	0.66795293	0	NA	PCSK9	PCSK9
PDE4DIP	2.650362	0.69291976	0	NA	PDE4DIP	PDE4DIP
PDE8B	-2.9336571	1.67372112	0	NA	NA	PDE8B
PDZRN3	2.3995817	0.66270499	0	NA	NA	PDZRN3
PDZRN4	-5.4476486	2.63707924	0	NA	PDZRN4	PDZRN4
PENK	-6.7368517	6.43723769	0	NA	NA	PENK
PGM1	-2.8866915	1.47795452	0	NA	NA	PGM1
PHLDA1	-4.0501691	1.65922624	0	NA	PHLDA1	PHLDA1
PIK3AP1	2.7624911	0.64434479	0	NA	PIK3AP1	PIK3AP1
PKP4	-2.4639251	1.3398196	0	NA	NA	PKP4
PLCXD3	4.2669402	0.47628416	0	NA	NA	PLCXD3
PLEKHG1	-3.4388593	2.35048104	0	NA	NA	PLEKHG1
POPDC3	-4.034346	2.24011236	0	NA	NA	POPDC3
POU6F2	-2.6805439	1.63663371	0	NA	NA	POU6F2
PPARGC1A	2.5259817	0.69605157	0	NA	PPARGC1A	PPARGC1A
PPEF1	8.0271043	0.3765052	0	NA	NA	PPEF1
PROK2	3.9923299	0.40336252	0	NA	NA	PROK2
PRSS23	-8.5327816	3.48366176	0	NA	NA	PRSS23
PRUNE2	-2.1695295	1.38494176	0	NA	NA	PRUNE2
PSD2	-2.1802477	1.30380301	0	NA	PSD2	PSD2
PTDSS1	3.5101653	0.65010501	0	NA	NA	PTDSS1
PTER	-3.937988	2.21395294	0	NA	PTER	PTER
PTGFR	-4.2996081	3.03066476	0	NA	NA	PTGFR
PTPN13	3.4056784	0.52764387	0	NA	NA	PTPN13
PTPN2	2.2949353	0.72235209	0	NA	NA	PTPN2
PTPRK	4.7632103	0.3977537	0	NA	PTPRK	PTPRK
PTPRT	-2.748046	1.71787085	0	NA	NA	PTPRT
PTX3	-5.0063375	2.69234918	0	NA	NA	PTX3
QDPR	-2.1979566	1.33072736	0	NA	QDPR	QDPR
RAB7L1	3.8609759	0.56498331	0	NA	RAB7L1	RAB7L1
RASGRP1	8.1145198	0.18252844	0	NA	NA	RASGRP1
RASL11B	4.6466211	0.54763951	0	NA	NA	RASL11B
RBP1	-2.2620478	1.66082427	0	NA	NA	RBP1
RCAN1	-3.188018	1.56161412	0	NA	NA	RCAN1
RCAN2	-6.3698221	4.29708113	0	NA	NA	RCAN2
RFK	-3.0766184	1.42806894	0	NA	NA	RFK
RFTN1	-2.4595346	1.37948102	0	NA	NA	RFTN1
RGS20	2.36348	0.71130303	0	NA	RGS20	RGS20
RGS8	-2.4556735	1.75033938	0	NA	NA	RGS8

RGS9	-2.3931161	1.54817406	0	NA	NA	RGS9
RNF182	4.7106408	0.47962861	0	NA	RNF182	RNF182
ROBO2	5.3895655	0.46474463	0	NA	ROBO2	ROBO2
RPL23AP32	2.2281744	0.68807363	0	NA	NA	RPL23AP32
RPRM	-3.0311581	2.03229418	0	NA	RPRM	RPRM
RPRML	3.4553787	0.39643637	0	NA	NA	RPRML
RRAS2	-2.2138059	1.43616073	0	NA	NA	RRAS2
RXFP1	-9.3099821	9.18590416	0	NA	NA	RXFP1
RXRG	-5.5586275	2.9990711	0	NA	NA	RXRG
RYR3	5.4519481	0.38078123	0	NA	NA	RYR3
SAMD13	-3.0569876	1.73761783	0	NA	NA	SAMD13
SAV1	4.2713176	0.49855907	0	NA	NA	SAV1
SDC2	2.240968	0.57465879	0	NA	NA	SDC2
SEMA3E	-2.3056371	1.63520145	0	NA	NA	SEMA3E
SERPINI1	3.7436343	0.60735495	0	NA	NA	SERPINI1
SEZ6L	2.383783	0.70083586	0	NA	NA	SEZ6L
SFRP1	-3.1715505	1.60973547	0	NA	SFRP1	SFRP1
SFRP2	-2.9745118	1.69062342	0	NA	SFRP2	SFRP2
SGCG	-3.5579458	2.88288651	0	NA	NA	SGCG
SGEF	3.0105396	0.51600388	0	NA	NA	SGEF
SGK1	4.5344644	0.54497708	0	NA	NA	SGK1
SH3D19	-2.1801666	1.33741731	0	NA	NA	SH3D19
SHISA2	5.6562868	0.33524399	0	NA	NA	SHISA2
SLC12A2	-2.2112538	1.5342079	0	NA	NA	SLC12A2
SLC17A7	3.8280472	0.65556488	0	NA	NA	SLC17A7
SLC35F2	-2.9960979	1.96965228	0	NA	NA	SLC35F2
SLC6A15	2.7910928	0.67529137	0	NA	SLC6A15	SLC6A15
SLCO3A1	-2.3528977	1.73020418	0	NA	SLCO3A1	SLCO3A1
SLIT2	-3.0879381	1.56586266	0	NA	SLIT2	SLIT2
SLITRK3	-4.2346195	2.044461	0	NA	NA	SLITRK3
SLITRK4	2.2845762	0.74566532	0	NA	NA	SLITRK4
SMC4	3.2304147	0.66233649	0	NA	NA	SMC4
SNCAIP	-2.3999304	1.44456808	0	NA	NA	SNCAIP
SOCS2	-2.3828863	1.62618978	0	NA	NA	SOCS2
SORCS1	4.3988439	0.48451882	0	NA	SORCS1	SORCS1
SOX9	12.91514	0.11641769	0	NA	NA	SOX9
SPIRE1	3.7605242	0.60874536	0	NA	NA	SPIRE1
SPON1	5.0932677	0.30520412	0	NA	SPON1	SPON1
SRI	-2.2418818	1.30176061	0	NA	NA	SRI
SSTR1	2.2225815	0.45025199	0	NA	NA	SSTR1
ST3GAL1	2.3076774	0.65009901	0	NA	NA	ST3GAL1
ST6GAL2	4.5318538	0.44398221	0	NA	NA	ST6GAL2
ST8SIA3	-2.4241874	1.47965344	0	NA	ST8SIA3	ST8SIA3
STARD4	3.0072284	0.63499894	0	NA	NA	STARD4
STK31	3.157706	0.29964372	0	NA	NA	STK31
STXBP6	-7.5596973	3.29939452	0	NA	STXBP6	STXBP6
SULF1	-4.571398	2.76779119	0	NA	NA	SULF1
SULF2	-4.7353515	2.57250946	0	NA	SULF2	SULF2
SVIP	3.1539867	0.63591265	0	NA	NA	SVIP
SYNE1	2.7432808	0.71460998	0	NA	SYNE1	SYNE1
SYNM	-3.2827036	1.63034073	0	NA	SYNM	SYNM
SYNPR	2.2112118	0.6402151	0	NA	NA	SYNPR
TACR2	2.2351101	0.65506084	0	NA	NA	TACR2
TAL2	-2.8474592	1.98116717	0	NA	NA	TAL2
TBC1D8	3.2520082	0.56460283	0	NA	NA	TBC1D8
TCF7L2	-2.7195924	1.46882282	0	NA	NA	TCF7L2
TDRD7	2.4033651	0.71213719	0	NA	NA	TDRD7
TGFA	3.641964	0.59550569	0	NA	NA	TGFA

TGFB2	5.2878672	0.38054757	0	NA	NA	TGFB2
TGFBR1	2.4199176	0.6526334	0	NA	NA	TGFBR1
THSD7A	3.3056219	0.55085256	0	NA	NA	THSD7A
TLE1	9.2333799	0.33331922	0	NA	TLE1	TLE1
TLK1	3.19175	0.6193214	0	NA	NA	TLK1
TMCC2	2.363984	0.72802771	0	NA	TMCC2	TMCC2
TMEM200A	4.5987098	0.48562263	0	NA	NA	TMEM200A
TMEM35	-2.2798528	1.40446853	0	NA	NA	TMEM35
TNFRSF21	-2.2771288	1.33797149	0	NA	NA	TNFRSF21
TNKS2	2.4188874	0.6724906	0	NA	NA	TNKS2
TOX	-6.2470255	3.16050207	0	NA	TOX	TOX
TP53I3	-4.5234967	2.3275304	0	NA	NA	TP53I3
TPBG	-2.3870222	1.57377889	0	NA	TPBG	TPBG
TRH	-4.3485877	1.92544255	0	NA	TRH	TRH
TRPC4	5.6127943	0.39063655	0	NA	NA	TRPC4
TRPC7	3.4270364	0.44069231	0	NA	NA	TRPC7
TSHZ1	-2.7662182	1.5240847	0	NA	NA	TSHZ1
TSHZ3	-4.8185133	2.42675149	0	NA	NA	TSHZ3
UFM1	2.5808817	0.67122313	0	NA	NA	UFM1
UNC13C	5.2100561	0.37864663	0	NA	NA	UNC13C
USP46	2.7529312	0.69716762	0	NA	NA	USP46
UST	-2.3511955	1.63237533	0	NA	NA	UST
WBSCR17	-3.4505242	1.54274478	0	NA	WBSCR17	WBSCR17
WIF1	2.9832351	0.33811108	0	NA	NA	WIF1
WNT11	-2.185204	1.57874503	0	NA	WNT11	WNT11
WNT7B	3.4683119	0.48789458	0	NA	NA	WNT7B
XK	2.2480153	0.63144829	0	NA	NA	XK
ZNF114	-3.8851986	2.8202289	0	NA	NA	ZNF114
ZNF238	2.8390867	0.596913	0	NA	ZNF238	ZNF238
ZNF385B	3.1447634	0.46473021	0	NA	NA	ZNF385B
ZNF423	-6.9022481	4.59746511	0	NA	ZNF423	ZNF423
ZNF503	2.7163132	0.66857216	0	NA	NA	ZNF503
ZNF618	2.4840875	0.73010905	0	NA	NA	ZNF618
NCAM2	3.8690279	0.48696699	0	NCAM2	NCAM2	NCAM2
NDST3	-5.1022264	3.64574782	0	NDST3	NA	NDST3
OPCML	3.7307845	0.52898093	0	OPCML	OPCML	OPCML
PART1	-4.6456628	2.46597251	0	PART1	PART1	PART1
PCDH10	-5.8434513	2.90835591	0	PCDH10	NA	PCDH10
PCDH7	4.9943541	0.52826125	0	PCDH7	PCDH7	PCDH7
PCDH9	5.6119571	0.38338733	0	PCDH9	PCDH9	PCDH9
PLEKHH2	2.7074491	0.60084142	0	PLEKHH2	NA	PLEKHH2
PLK2	-4.1808613	1.84061633	0	PLK2	NA	PLK2
POU3F2	-2.9474857	1.60823013	0	POU3F2	NA	POU3F2
POU3F3	-2.3728614	1.478081	0	POU3F3	POU3F3	POU3F3
PRAGMIN	-5.4987885	2.1050636	0	PRAGMIN	PRAGMIN	PRAGMIN
PRR16	7.3717628	0.20836697	0	PRR16	PRR16	PRR16
RAB23	2.6716488	0.69797553	0	RAB23	NA	RAB23
ROBO1	-2.1733223	1.36044369	0	ROBO1	ROBO1	ROBO1
RSPO2	4.4727342	0.45629497	0	RSPO2	NA	RSPO2
SDK1	-8.517135	2.97888689	0	SDK1	NA	SDK1
SLITRK1	-2.3569568	1.36395396	0	SLITRK1	NA	SLITRK1
SORCS3	3.7751832	0.46911502	0	SORCS3	SORCS3	SORCS3
SPATA18	-2.9348408	1.57045958	0	SPATA18	NA	SPATA18
ST6GALNAC5	-6.0992162	2.36657776	0	ST6GALNAC5	ST6GALNAC5	ST6GALNAC5
TCERG1L	-4.262795	2.66773624	0	TCERG1L	NA	TCERG1L
TFAP2D	-3.5078229	3.65801776	0	TFAP2D	TFAP2D	TFAP2D
THBS1	5.2207093	0.27836758	0	THBS1	NA	THBS1
TMCC3	2.9003394	0.62058748	0	TMCC3	NA	TMCC3

TRHDE	7.4632306	0.22727706	0	TRHDE	TRHDE	TRHDE
VSTM2L	-7.6922484	5.1814054	0	VSTM2L	NA	VSTM2L
YES1	-3.7572556	1.84409437	0	YES1	YES1	YES1
ZBTB20	-2.2143973	1.32042789	0	ZBTB20	ZBTB20	ZBTB20
ARAP2	2.1194073	0.7517203	0.11329523	ARAP2	ARAP2	ARAP2
ARHGAP15	2.0953211	0.73642574	0.11329523	ARHGAP15	ARHGAP15	ARHGAP15
EFNB2	2.0686762	0.67820706	0.11329523	EFNB2	EFNB2	EFNB2
ELAVL2	-2.000231	1.49945115	0.11329523	ELAVL2	ELAVL2	ELAVL2
ETV5	2.0643668	0.73596483	0.11329523	ETV5	NA	ETV5
FLJ42709	2.0428552	0.71173922	0.11329523	FLJ42709	FLJ42709	FLJ42709
GLIS3	-2.1030173	1.66495303	0.11329523	GLIS3	GLIS3	GLIS3
ABHD6	2.0503227	0.76084925	0.11329523	NA	NA	ABHD6
ADIPOR2	2.1669984	0.75632768	0.11329523	NA	NA	ADIPOR2
ADSSL1	-2.024042	1.31061038	0.11329523	NA	NA	ADSSL1
AGFG1	2.1688378	0.76608477	0.11329523	NA	NA	AGFG1
AJAP1	-2.0384512	1.31571584	0.11329523	NA	NA	AJAP1
AKR1C1	-2.1625288	1.35573308	0.11329523	NA	NA	AKR1C1
ANKRD29	-2.0779442	1.53237741	0.11329523	NA	NA	ANKRD29
AP1S2	2.1771745	0.7818732	0.11329523	NA	NA	AP1S2
BVES	-2.0094566	1.44099132	0.11329523	NA	NA	BVES
C13orf36	-2.0945015	2.13159181	0.11329523	NA	NA	C13orf36
C1orf173	2.1605387	0.65197987	0.11329523	NA	NA	C1orf173
C3orf50	-2.036135	1.98550562	0.11329523	NA	NA	C3orf50
C5orf4	2.0449505	0.75038304	0.11329523	NA	C5orf4	C5orf4
C7orf36	-2.157551	1.48566933	0.11329523	NA	NA	C7orf36
CBARA1	-2.0722511	1.36405196	0.11329523	NA	NA	CBARA1
DPF3	-1.9948572	1.32280327	0.11329523	NA	DPF3	DPF3
DRD1	-2.1159006	1.84786237	0.11329523	NA	NA	DRD1
EBPL	-2.0174033	1.31289045	0.11329523	NA	NA	EBPL
EIF3E	2.1041583	0.79564492	0.11329523	NA	EIF3E	EIF3E
EML6	2.0822228	0.71419206	0.11329523	NA	NA	EML6
EMX1	2.0036574	0.73095406	0.11329523	NA	NA	EMX1
FRY	2.1460488	0.74024331	0.11329523	NA	NA	FRY
GGCT	-2.0093063	1.27766155	0.11329523	NA	NA	GGCT
GGTA1	-2.1053034	1.54639322	0.11329523	NA	GGTA1	GGTA1
GOLGA7B	-2.1550231	1.44318056	0.11329523	NA	GOLGA7B	GOLGA7B
GPAM	2.140471	0.68699697	0.11329523	NA	GPAM	GPAM
GRIK1	2.0950291	0.68395149	0.11329523	NA	NA	GRIK1
GRIK2	-2.1000341	1.32784063	0.11329523	NA	GRIK2	GRIK2
GRM5	-2.0873797	1.32209824	0.11329523	NA	NA	GRM5
GRM7	-1.9945815	1.33335681	0.11329523	NA	GRM7	GRM7
HAS2	2.0574799	0.6831462	0.11329523	NA	NA	HAS2
HSPB3	-2.111997	1.51116191	0.11329523	NA	HSPB3	HSPB3
HTR7	2.0742972	0.71424476	0.11329523	NA	HTR7	HTR7
LOC387763	2.0187893	0.76468361	0.11329523	NA	NA	LOC387763
LRRC7	2.1384318	0.73077893	0.11329523	NA	NA	LRRC7
MYF6	-2.0699629	1.36606021	0.11329523	NA	NA	MYF6
NECAB1	2.0109467	0.68407983	0.11329523	NA	NA	NECAB1
NEDD4	2.1359013	0.73156625	0.11329523	NA	NA	NEDD4
NUAK2	2.0193351	0.72768633	0.11329523	NA	NA	NUAK2
OMG	2.0489617	0.72221266	0.11329523	NA	NA	OMG
PDGFD	2.180256	0.67258826	0.11329523	NA	NA	PDGFD
PDZD2	2.1692008	0.69590969	0.11329523	NA	NA	PDZD2
PGBD5	-2.0289311	1.31080757	0.11329523	NA	NA	PGBD5
PTPRZ1	2.1107313	0.75631262	0.11329523	NA	NA	PTPRZ1
RAB11A	-2.0923258	1.24803638	0.11329523	NA	NA	RAB11A
REM2	-2.1261275	1.56858986	0.11329523	NA	NA	REM2
RFC3	2.091419	0.71567047	0.11329523	NA	NA	RFC3

RSPH9	2.0134479	0.74456472	0.11329523	NA	NA	RSPH9
SATB2	2.0138674	0.6652304	0.11329523	NA	SATB2	SATB2
SERPINB6	-2.0431848	1.28622809	0.11329523	NA	NA	SERPINB6
SERTAD2	2.1096883	0.74027596	0.11329523	NA	NA	SERTAD2
SFRS2B	-2.1154615	1.27174603	0.11329523	NA	NA	SFRS2B
SH3BP4	-2.1323932	1.35398485	0.11329523	NA	SH3BP4	SH3BP4
SLC17A5	-2.1683251	1.35369328	0.11329523	NA	NA	SLC17A5
SLC26A8	2.0197541	0.66891371	0.11329523	NA	NA	SLC26A8
SLC27A2	2.1396481	0.7838011	0.11329523	NA	NA	SLC27A2
SMPDL3A	2.1051156	0.710081	0.11329523	NA	NA	SMPDL3A
SNHG8	2.029819	0.76749506	0.11329523	NA	NA	SNHG8
SV2B	-2.1380977	1.40507555	0.11329523	NA	NA	SV2B
TAF4B	2.163867	0.73515042	0.11329523	NA	NA	TAF4B
TAPT1	-2.0881596	1.24815485	0.11329523	NA	NA	TAPT1
TARBP1	2.0160223	0.77167533	0.11329523	NA	TARBP1	TARBP1
TBX21	-2.0951755	1.50634971	0.11329523	NA	TBX21	TBX21
NFIA	-2.0249092	1.54756066	0.11329523	NFIA	NA	NFIA
NR4A3	2.0575626	0.77554613	0.11329523	NR4A3	NA	NR4A3
PMP22	-2.1046144	1.88169418	0.11329523	PMP22	NA	PMP22
RPS6KA3	2.0394529	0.74879937	0.11329523	RPS6KA3	RPS6KA3	RPS6KA3
SNX16	2.0222737	0.73542514	0.11329523	SNX16	NA	SNX16
THRB	-2.1344228	1.6017252	0.11329523	THRB	NA	THRB
C3orf58	1.9392572	0.78454523	0.19206239	C3orf58	NA	C3orf58
C8orf83	1.9837388	0.75462987	0.19206239	C8orf83	C8orf83	C8orf83
CNTNAP5	1.8672665	0.7789539	0.19206239	CNTNAP5	CNTNAP5	CNTNAP5
DNAJC1	1.8836523	0.7871951	0.19206239	DNAJC1	NA	DNAJC1
DOK6	1.968978	0.74194772	0.19206239	DOK6	NA	DOK6
ETV1	1.8694001	0.72920593	0.19206239	ETV1	ETV1	ETV1
FAM84A	1.882711	0.74357273	0.19206239	FAM84A	FAM84A	FAM84A
FANCL	1.9582831	0.77641708	0.19206239	FANCL	FANCL	FANCL
FBXL17	1.8906911	0.71481587	0.19206239	FBXL17	FBXL17	FBXL17
GRIA3	1.848451	0.74980662	0.19206239	GRIA3	GRIA3	GRIA3
KCNJ2	1.8946814	0.65167684	0.19206239	KCNJ2	NA	KCNJ2
KIAA0114	1.8648166	0.75999109	0.19206239	KIAA0114	NA	KIAA0114
LPHN3	-1.935682	1.28725915	0.19206239	LPHN3	NA	LPHN3
MGST1	1.8604752	0.70169159	0.19206239	MGST1	MGST1	MGST1
ACVR1	1.9016813	0.74909317	0.19206239	NA	ACVR1	ACVR1
ADC	1.882217	0.74554495	0.19206239	NA	NA	ADC
ADCY1	1.8937999	0.78767214	0.19206239	NA	NA	ADCY1
AKAP13	1.8672226	0.77684803	0.19206239	NA	AKAP13	AKAP13
AKAP7	-1.9212017	1.33142677	0.19206239	NA	NA	AKAP7
AKR1B1	-1.8873825	1.26669329	0.19206239	NA	NA	AKR1B1
ALDH7A1	-1.9677894	1.56588168	0.19206239	NA	NA	ALDH7A1
ANK2	1.9805793	0.68917144	0.19206239	NA	NA	ANK2
ANKRD6	-1.9501876	1.4522325	0.19206239	NA	NA	ANKRD6
APBB2	-1.9369471	1.33781927	0.19206239	NA	NA	APBB2
ARHGAP12	1.9922063	0.64834482	0.19206239	NA	NA	ARHGAP12
ARHGAP18	1.968179	0.70485716	0.19206239	NA	NA	ARHGAP18
ASS1	-1.8757098	1.36348358	0.19206239	NA	ASS1	ASS1
ATP2C1	1.8198191	0.76723843	0.19206239	NA	NA	ATP2C1
BAI2	1.9038778	0.68664261	0.19206239	NA	NA	BAI2
C10orf58	-1.9142667	1.24940341	0.19206239	NA	NA	C10orf58
C11orf75	-1.9505339	1.68916961	0.19206239	NA	NA	C11orf75
C14orf37	1.9223885	0.66899015	0.19206239	NA	NA	C14orf37
C2orf3	1.8417981	0.75585756	0.19206239	NA	NA	C2orf3
C2orf7	-1.8899315	1.31692006	0.19206239	NA	NA	C2orf7
C9orf45	1.9496523	0.75910403	0.19206239	NA	C9orf45	C9orf45
CAMKV	1.8540275	0.77291741	0.19206239	NA	NA	CAMKV

CAV1	-1.8666365	1.46701097	0.19206239	NA	NA	CAV1
CEP135	1.8777125	0.76959028	0.19206239	NA	NA	CEP135
CYYR1	-1.8344824	1.39509513	0.19206239	NA	CYYR1	CYYR1
DEK	1.8585399	0.81623636	0.19206239	NA	NA	DEK
DENND4C	1.8540265	0.79761758	0.19206239	NA	NA	DENND4C
DPP3	-1.8794705	1.2852484	0.19206239	NA	DPP3	DPP3
DPYD	1.91158	0.75716218	0.19206239	NA	DPYD	DPYD
DYNC2H1	1.9865679	0.71849855	0.19206239	NA	NA	DYNC2H1
EIF3D	1.8358103	0.81949329	0.19206239	NA	NA	EIF3D
EPB41L3	1.819643	0.72224433	0.19206239	NA	NA	EPB41L3
FABP7	1.9955677	0.69389679	0.19206239	NA	NA	FABP7
FAM43A	-1.8784603	1.28227334	0.19206239	NA	NA	FAM43A
FRMD4B	-1.9884055	1.29900971	0.19206239	NA	NA	FRMD4B
GCA	1.9918127	0.6942364	0.19206239	NA	NA	GCA
GHRH	-1.8694463	1.32426279	0.19206239	NA	NA	GHRH
HCN1	1.8768348	0.75954513	0.19206239	NA	NA	HCN1
HS3ST2	-1.9807592	1.51306092	0.19206239	NA	HS3ST2	HS3ST2
IGSF21	1.8198256	0.7643972	0.19206239	NA	NA	IGSF21
IMPDH2	1.8557926	0.80768111	0.19206239	NA	NA	IMPDH2
KCNAB1	-1.9093715	1.39623488	0.19206239	NA	NA	KCNAB1
KCNE1	-1.9868154	1.38190226	0.19206239	NA	NA	KCNE1
KLHL14	-1.8384008	1.35188201	0.19206239	NA	KLHL14	KLHL14
LOC100129550	1.8909605	0.74844148	0.19206239	NA	NA	LOC100129550
LOC100134229	-1.9667124	1.43010266	0.19206239	NA	NA	LOC100134229
LOC151162	-1.8938993	1.26626375	0.19206239	NA	NA	LOC151162
LRRC8C	-1.9658767	1.28337061	0.19206239	NA	NA	LRRC8C
MYB	-1.9590426	1.33546126	0.19206239	NA	NA	MYB
MYC	-1.8492218	1.26090084	0.19206239	NA	NA	MYC
N4BP2L2	1.8287236	0.76511986	0.19206239	NA	NA	N4BP2L2
NAP1L5	-1.8801924	1.29018764	0.19206239	NA	NA	NAP1L5
NOS1	-1.9311619	1.2985339	0.19206239	NA	NOS1	NOS1
NPNT	-1.8799266	1.43839345	0.19206239	NA	NA	NPNT
PAG1	1.9923476	0.75355565	0.19206239	NA	NA	PAG1
PHF17	1.9406617	0.73516516	0.19206239	NA	NA	PHF17
PITPNC1	-1.9241893	1.28031768	0.19206239	NA	NA	PITPNC1
PLCXD2	-1.8698634	1.26021415	0.19206239	NA	NA	PLCXD2
PLOD2	1.8199163	0.63535707	0.19206239	NA	PLOD2	PLOD2
RAB2B	-1.8679581	1.22997804	0.19206239	NA	NA	RAB2B
RHPN2	1.9213888	0.73483556	0.19206239	NA	NA	RHPN2
RIN2	1.9573726	0.76035393	0.19206239	NA	NA	RIN2
RP1-177G6.2	1.9769926	0.56392297	0.19206239	NA	NA	RP1-177G6.2
RPH3A	-1.8374374	1.75038429	0.19206239	NA	NA	RPH3A
RPL37	1.8538167	0.80050686	0.19206239	NA	NA	RPL37
RSL24D1	1.9607116	0.76287592	0.19206239	NA	RSL24D1	RSL24D1
SCNN1G	-1.9659273	1.41568303	0.19206239	NA	NA	SCNN1G
SLC46A3	-1.8978447	1.28379808	0.19206239	NA	NA	SLC46A3
SNAI2	-1.8931277	1.42988906	0.19206239	NA	NA	SNAI2
SNX9	1.8759538	0.72844561	0.19206239	NA	SNX9	SNX9
SUPT3H	-1.8828569	1.41033346	0.19206239	NA	NA	SUPT3H
SYDE2	-1.9700165	1.40108213	0.19206239	NA	NA	SYDE2
TIAM2	1.9183267	0.7270701	0.19206239	NA	NA	TIAM2
TMEM74	1.9308704	0.72485958	0.19206239	NA	NA	TMEM74
TMTC1	1.9325874	0.60091483	0.19206239	NA	NA	TMTC1
TRIM44	1.9809887	0.76321079	0.19206239	NA	NA	TRIM44
TSPAN18	-1.941617	1.23064855	0.19206239	NA	NA	TSPAN18
TSPAN6	-1.9717261	1.54699507	0.19206239	NA	NA	TSPAN6

WNT3	-1.8373978	1.28145237	0.19206239	NA	NA	WNT3
WNT5B	1.9573525	0.78654814	0.19206239	NA	NA	WNT5B
ZADH2	-1.9388644	1.33605933	0.19206239	NA	ZADH2	ZADH2
ZNF438	1.9131199	0.79043807	0.19206239	NA	NA	ZNF438
ZNF706	-1.8489992	1.22405684	0.19206239	NA	ZNF706	ZNF706
NLGN1	1.9729994	0.70393173	0.19206239	NLGN1	NLGN1	NLGN1
PARP8	-1.8990028	1.45305744	0.19206239	PARP8	PARP8	PARP8
PLCB4	-1.9883246	1.38632628	0.19206239	PLCB4	NA	PLCB4
POU3F1	-1.895519	1.42385159	0.19206239	POU3F1	POU3F1	POU3F1
PRKD1	-1.9272667	1.32258417	0.19206239	PRKD1	PRKD1	PRKD1
SATB1	1.8658506	0.79817306	0.19206239	SATB1	SATB1	SATB1
XYLT1	1.9222051	0.80021924	0.19206239	XYLT1	XYLT1	XYLT1
BIN1	-1.7670401	1.42300715	0.56921718	BIN1	NA	BIN1
CA8	-1.7263499	1.23979534	0.56921718	CA8	CA8	CA8
CDH11	-1.6807595	1.26770628	0.56921718	CDH11	CDH11	CDH11
DCTD	-1.7916642	1.25421313	0.56921718	DCTD	DCTD	DCTD
DIO2	-1.6845813	1.34564495	0.56921718	DIO2	DIO2	DIO2
LRRTM1	-1.7425734	1.47680773	0.56921718	LRRTM1	LRRTM1	LRRTM1
ABI2	1.8002732	0.78889966	0.56921718	NA	NA	ABI2
ADD2	1.7072329	0.79782557	0.56921718	NA	NA	ADD2
AHR	1.7311067	0.74526905	0.56921718	NA	NA	AHR
ALS2	1.730133	0.77799375	0.56921718	NA	NA	ALS2
AMMECR1	1.7897814	0.77927582	0.56921718	NA	NA	AMMECR1
AMOTL1	-1.6708045	1.2233893	0.56921718	NA	AMOTL1	AMOTL1
ARHGEF3	-1.6834136	1.22494225	0.56921718	NA	NA	ARHGEF3
ARL4C	-1.7089553	1.21333535	0.56921718	NA	NA	ARL4C
ATP2B1	1.7101363	0.78636865	0.56921718	NA	NA	ATP2B1
B3GALTL	-1.7134436	1.54733497	0.56921718	NA	NA	B3GALTL
BCAP29	1.77772	0.79518995	0.56921718	NA	NA	BCAP29
BCAR3	1.6554876	0.78105918	0.56921718	NA	NA	BCAR3
C17orf58	-1.664373	1.25174985	0.56921718	NA	NA	C17orf58
C1orf201	1.6867839	0.76162761	0.56921718	NA	C1orf201	C1orf201
CABP1	1.7476754	0.72210881	0.56921718	NA	NA	CABP1
CHD3	1.7570376	0.74835474	0.56921718	NA	NA	CHD3
CHGB	1.6889775	0.80786526	0.56921718	NA	CHGB	CHGB
CHRNB1	-1.6878377	1.30251577	0.56921718	NA	NA	CHRNB1
CSMD2	1.6504898	0.78251864	0.56921718	NA	NA	CSMD2
CTGF	-1.7315289	1.50144371	0.56921718	NA	NA	CTGF
DGKH	1.6708511	0.81766292	0.56921718	NA	NA	DGKH
DIAPH3	-1.691133	1.316908	0.56921718	NA	DIAPH3	DIAPH3
DNAJC12	-1.779901	1.33330638	0.56921718	NA	NA	DNAJC12
DUSP6	-1.6648534	1.34244361	0.56921718	NA	DUSP6	DUSP6
E2F3	-1.7467634	1.27894087	0.56921718	NA	NA	E2F3
ECHDC3	1.7663812	0.70254225	0.56921718	NA	NA	ECHDC3
FBXL4	1.7030863	0.74322208	0.56921718	NA	FBXL4	FBXL4
FGFR1	-1.7778665	1.21511311	0.56921718	NA	NA	FGFR1
FNDC3B	1.7206151	0.81477116	0.56921718	NA	NA	FNDC3B
FOSL2	-1.8048882	1.39235042	0.56921718	NA	FOSL2	FOSL2
FTL	-1.6927348	1.23643025	0.56921718	NA	NA	FTL
GNAL	-1.6730574	1.82837731	0.56921718	NA	NA	GNAL
GPR137C	-1.7683416	1.45479059	0.56921718	NA	NA	GPR137C
GPR63	1.7023713	0.67572853	0.56921718	NA	NA	GPR63
GRM3	1.7963915	0.79517321	0.56921718	NA	GRM3	GRM3
HEATR1	1.7673478	0.6703171	0.56921718	NA	NA	HEATR1
HSPA4L	1.7627049	0.69399936	0.56921718	NA	NA	HSPA4L
HYMAI	1.7725019	0.66603623	0.56921718	NA	NA	HYMAI
ID4	-1.714183	1.24941014	0.56921718	NA	ID4	ID4
IPO8	1.7151015	0.7815551	0.56921718	NA	NA	IPO8

JAK2	1.766462	0.78345728	0.56921718	NA	NA	JAK2
KCNK2	-1.6590451	1.31587451	0.56921718	NA	NA	KCNK2
KLHL25	1.6601986	0.75803502	0.56921718	NA	NA	KLHL25
LOC401097	1.7677791	0.6990811	0.56921718	NA	NA	LOC401097
LRRC4C	1.7723172	0.78380855	0.56921718	NA	LRRC4C	LRRC4C
LYSMD3	1.6959089	0.74857584	0.56921718	NA	NA	LYSMD3
MAN2A1	1.7357733	0.68817559	0.56921718	NA	NA	MAN2A1
MAOA	-1.7230175	1.26439033	0.56921718	NA	MAOA	MAOA
MIAT	1.6685052	0.7468721	0.56921718	NA	MIAT	MIAT
NALCN	-1.7134353	1.33890556	0.56921718	NA	NA	NALCN
NEFM	1.8104381	0.76583052	0.56921718	NA	NA	NEFM
NGB	1.6973064	0.81316304	0.56921718	NA	NGB	NGB
NR2F2	1.6810585	0.78707751	0.56921718	NA	NR2F2	NR2F2
NRXN3	-1.6779785	1.23251032	0.56921718	NA	NRXN3	NRXN3
OAT	-1.665461	1.23767024	0.56921718	NA	NA	OAT
PAQR8	-1.6793243	1.3547052	0.56921718	NA	NA	PAQR8
PCOTH	-1.7710008	1.2697666	0.56921718	NA	NA	PCOTH
PLXNC1	-1.7689523	1.29644548	0.56921718	NA	PLXNC1	PLXNC1
PPA2	-1.716176	1.29695608	0.56921718	NA	NA	PPA2
PPP1R3C	-1.7865883	1.27858437	0.56921718	NA	NA	PPP1R3C
PRDM10	1.7214244	0.80448983	0.56921718	NA	NA	PRDM10
QPCT	-1.6989685	1.52267803	0.56921718	NA	NA	QPCT
RAPGEFL1	1.7176713	0.71074745	0.56921718	NA	RAPGEFL1	RAPGEFL1
RBM24	1.7236535	0.77326309	0.56921718	NA	NA	RBM24
RGS16	-1.6671199	1.33345675	0.56921718	NA	NA	RGS16
RUNX2	-1.6858028	1.28467826	0.56921718	NA	RUNX2	RUNX2
SAMD3	1.8075569	0.77995226	0.56921718	NA	NA	SAMD3
SCRN1	1.7554073	0.77450968	0.56921718	NA	NA	SCRN1
SEMA3C	-1.6712737	1.36815885	0.56921718	NA	NA	SEMA3C
SHB	1.7365438	0.81139146	0.56921718	NA	NA	SHB
SIPA1L2	1.7967593	0.81728709	0.56921718	NA	NA	SIPA1L2
SLC17A8	-1.6870861	1.51521774	0.56921718	NA	NA	SLC17A8
SMAD3	1.7388772	0.78593321	0.56921718	NA	SMAD3	SMAD3
SPATA13	-1.6937885	1.32035141	0.56921718	NA	NA	SPATA13
SYT9	-1.7174943	1.24831857	0.56921718	NA	NA	SYT9
TBC1D1	-1.7283841	1.22658604	0.56921718	NA	NA	TBC1D1
TBR1	-1.7852788	1.28154788	0.56921718	NA	NA	TBR1
TNFAIP3	-1.7459981	1.72654034	0.56921718	NA	NA	TNFAIP3
UBP1	1.7354988	0.76318925	0.56921718	NA	NA	UBP1
VEPH1	-1.6686711	1.21287794	0.56921718	NA	NA	VEPH1
WWP2	-1.6644297	1.26294759	0.56921718	NA	NA	WWP2
ZAK	1.670217	0.71519869	0.56921718	NA	ZAK	ZAK
ZHX2	1.7521996	0.76600304	0.56921718	NA	ZHX2	ZHX2
NMNAT3	-1.7568169	1.32096251	0.56921718	NMNAT3	NA	NMNAT3
NTN1	-1.7302086	1.30085813	0.56921718	NTN1	NA	NTN1
PBX1	-1.7364712	1.3212575	0.56921718	PBX1	NA	PBX1
PTPRG	1.7029704	0.73794978	0.56921718	PTPRG	PTPRG	PTPRG
RAP1GAP	1.7936958	0.72250668	0.56921718	RAP1GAP	RAP1GAP	RAP1GAP
SLC35F4	-1.7080815	1.50640057	0.56921718	SLC35F4	SLC35F4	SLC35F4
TCF4	1.7294234	0.77062722	0.56921718	TCF4	NA	TCF4
VRK2	1.6716374	0.6899739	0.56921718	VRK2	VRK2	VRK2
ZNF703	-1.7219587	1.2649145	0.56921718	ZNF703	ZNF703	ZNF703
CADM1	1.5423172	0.78415892	0.88419955	CADM1	CADM1	CADM1
CADM2	1.6480507	0.78473465	0.88419955	CADM2	CADM2	CADM2
CDC73	1.5686281	0.79791535	0.88419955	CDC73	CDC73	CDC73
CSMD3	-1.5303673	1.2347212	0.88419955	CSMD3	NA	CSMD3
EFNA5	1.6238383	0.80556294	0.88419955	EFNA5	EFNA5	EFNA5
ETV6	-1.5174112	1.34083786	0.88419955	ETV6	NA	ETV6

FTSJD1	1.615738	0.73069219	0.88419955	FTSJD1	NA	FTSJD1
HNMT	1.6412867	0.77996165	0.88419955	HNMT	HNMT	HNMT
ITGA4	1.5542557	0.80397129	0.88419955	ITGA4	NA	ITGA4
KIF16B	1.6187313	0.77976982	0.88419955	KIF16B	KIF16B	KIF16B
LMO1	1.5536779	0.74571502	0.88419955	LMO1	LMO1	LMO1
LOC220930	1.5258504	0.73363307	0.88419955	LOC220930	LOC220930	LOC220930
AMIGO2	1.5389169	0.79046747	0.88419955	NA	NA	AMIGO2
ASPH	1.6025921	0.81486279	0.88419955	NA	NA	ASPH
AUH	1.5088174	0.79367185	0.88419955	NA	AUH	AUH
BCL2	1.6262094	0.72497361	0.88419955	NA	NA	BCL2
BRAF	1.6067269	0.77384768	0.88419955	NA	NA	BRAF
C11orf87	-1.5472354	1.41698678	0.88419955	NA	NA	C11orf87
C1GALT1	-1.5028948	1.27063069	0.88419955	NA	NA	C1GALT1
C6orf138	-1.6177315	1.37118745	0.88419955	NA	NA	C6orf138
CAPZA2	1.4915659	0.82907958	0.88419955	NA	NA	CAPZA2
CCDC152	1.5109192	0.81777537	0.88419955	NA	NA	CCDC152
CDH13	1.5766612	0.71012446	0.88419955	NA	CDH13	CDH13
CDK8	1.6055934	0.82417525	0.88419955	NA	NA	CDK8
CLCC1	-1.5448079	1.28666703	0.88419955	NA	NA	CLCC1
CLCN4	-1.6496394	1.35400775	0.88419955	NA	CLCN4	CLCN4
CLCN5	-1.6268313	1.230451	0.88419955	NA	NA	CLCN5
CLGN	-1.6539475	1.5093067	0.88419955	NA	NA	CLGN
CLYBL	1.5408082	0.78965522	0.88419955	NA	NA	CLYBL
CPNE6	-1.6220942	1.36472646	0.88419955	NA	NA	CPNE6
DCC	-1.5468003	1.25641969	0.88419955	NA	DCC	DCC
DDHD1	1.6422988	0.76981857	0.88419955	NA	NA	DDHD1
DLEU2L	1.4927985	0.65515587	0.88419955	NA	NA	DLEU2L
DMXL1	1.5936059	0.79464748	0.88419955	NA	DMXL1	DMXL1
DNMT3A	-1.6393412	1.26259462	0.88419955	NA	DNMT3A	DNMT3A
ELAVL4	-1.588411	1.23423612	0.88419955	NA	NA	ELAVL4
FABP6	-1.6316726	1.23861445	0.88419955	NA	NA	FABP6
GAS2L1	1.5550568	0.79647135	0.88419955	NA	NA	GAS2L1
GAS2L3	1.5920415	0.75218211	0.88419955	NA	NA	GAS2L3
GPD2	1.5940441	0.78962328	0.88419955	NA	NA	GPD2
GPR177	-1.5759886	1.44536871	0.88419955	NA	NA	GPR177
GUCY1A3	-1.5753948	1.37360364	0.88419955	NA	NA	GUCY1A3
IRF2	-1.5233308	1.44229607	0.88419955	NA	NA	IRF2
JUN	-1.6519549	1.47071215	0.88419955	NA	NA	JUN
KBTBD8	1.5720203	0.79932808	0.88419955	NA	KBTBD8	KBTBD8
KEAP1	-1.5697444	1.24489759	0.88419955	NA	NA	KEAP1
KIAA1958	-1.5262747	1.26307324	0.88419955	NA	KIAA1958	KIAA1958
KPNA2	-1.5666428	1.24652842	0.88419955	NA	NA	KPNA2
MAN1C1	1.5184786	0.83719153	0.88419955	NA	NA	MAN1C1
MARK1	1.5520661	0.80040881	0.88419955	NA	MARK1	MARK1
MDH1	1.6458122	0.7827652	0.88419955	NA	MDH1	MDH1
MITF	1.626352	0.7826945	0.88419955	NA	MITF	MITF
MSH2	-1.5195244	1.23633967	0.88419955	NA	NA	MSH2
MYO1B	-1.6535742	1.26905342	0.88419955	NA	MYO1B	MYO1B
NBL1	-1.5567805	1.26174467	0.88419955	NA	NA	NBL1
NPHP3	1.6046245	0.80858105	0.88419955	NA	NA	NPHP3
NRD1	1.5794877	0.77648009	0.88419955	NA	NA	NRD1
NUDCD1	1.5156774	0.76342572	0.88419955	NA	NA	NUDCD1
PACSIN1	1.5554403	0.8116957	0.88419955	NA	NA	PACSIN1
PPAT	1.5750822	0.79892128	0.88419955	NA	NA	PPAT
PPFIA2	1.6049976	0.82402652	0.88419955	NA	NA	PPFIA2
PPP2R5A	1.5226625	0.79373633	0.88419955	NA	NA	PPP2R5A
PRKCB	1.623504	0.7653644	0.88419955	NA	PRKCB	PRKCB
PRSS12	1.5393879	0.7075545	0.88419955	NA	NA	PRSS12

PTGDS	-1.5720961	1.54986996	0.88419955	NA	NA	PTGDS
RCC2	-1.6074405	1.25214465	0.88419955	NA	NA	RCC2
RGMB	1.5259339	0.82087114	0.88419955	NA	NA	RGMB
RHOBTB1	1.5203465	0.80651117	0.88419955	NA	NA	RHOBTB1
RNF169	1.5327919	0.80108988	0.88419955	NA	NA	RNF169
RPL37A	1.5098859	0.80697164	0.88419955	NA	NA	RPL37A
RSPO3	-1.5307645	1.28286727	0.88419955	NA	NA	RSPO3
SCG2	-1.5585077	1.37500787	0.88419955	NA	NA	SCG2
SH3KBP1	1.6217678	0.81439028	0.88419955	NA	SH3KBP1	SH3KBP1
SLC44A5	1.5080185	0.7401177	0.88419955	NA	NA	SLC44A5
SLC5A3	1.6113967	0.81212852	0.88419955	NA	NA	SLC5A3
SMAD7	1.5822772	0.77254442	0.88419955	NA	SMAD7	SMAD7
SNX22	1.6470677	0.7752698	0.88419955	NA	NA	SNX22
SPRED2	-1.5160014	1.21935135	0.88419955	NA	SPRED2	SPRED2
SSBP2	1.4973895	0.83258835	0.88419955	NA	NA	SSBP2
STAG2	1.5384991	0.80153576	0.88419955	NA	STAG2	STAG2
STARD13	1.5338506	0.81861749	0.88419955	NA	NA	STARD13
STRBP	1.5625995	0.76323323	0.88419955	NA	NA	STRBP
SYT13	1.5262234	0.77492133	0.88419955	NA	SYT13	SYT13
TDP1	-1.6031528	1.24968789	0.88419955	NA	TDP1	TDP1
TF	-1.5563084	1.25399026	0.88419955	NA	NA	TF
TGFB3	-1.628569	1.28476102	0.88419955	NA	NA	TGFB3
TLN2	1.6341747	0.79796596	0.88419955	NA	NA	TLN2
TMEM47	-1.6560851	1.2938101	0.88419955	NA	TMEM47	TMEM47
TMEM5	-1.5773597	1.27996903	0.88419955	NA	TMEM5	TMEM5
TNRC6B	1.5843737	0.78946261	0.88419955	NA	NA	TNRC6B
TSEN15	-1.6274658	1.29362016	0.88419955	NA	NA	TSEN15
UBE2L6	-1.6195086	1.21735044	0.88419955	NA	NA	UBE2L6
UVRAG	1.5689983	0.81164414	0.88419955	NA	NA	UVRAG
ZDHHC23	1.5498989	0.74854916	0.88419955	NA	NA	ZDHHC23
ZNF365	1.6080121	0.78220485	0.88419955	NA	NA	ZNF365
ZNF395	-1.598808	1.21514445	0.88419955	NA	ZNF395	ZNF395
NRCAM	-1.5882778	1.23232595	0.88419955	NRCAM	NA	NRCAM
PTPRD	1.601349	0.82378207	0.88419955	PTPRD	PTPRD	PTPRD
SLC7A11	-1.5125867	1.3357056	0.88419955	SLC7A11	NA	SLC7A11
STIM2	1.6017321	0.73637926	0.88419955	STIM2	NA	STIM2
UNC5C	1.5826048	0.82539105	0.88419955	UNC5C	UNC5C	UNC5C

MAR-HAR PFOvsPT q<0.01

SYMBOL	PFOvsPT.d	PFOvsPT.FC	PFOvsPT.q	HAR	CAR	MAR
ACCN1	-2.6307648	1.67979598	0	NA	ACCN1	ACCN1
ADAMTS16	-2.3622011	1.76005451	0	NA	ADAMTS16	ADAMTS16
AK5	2.4672315	0.67898931	0	NA	AK5	AK5
ANKRD32	-5.2441495	2.29550366	0	NA	ANKRD32	ANKRD32
APOO	-2.2526913	1.32642345	0	NA	APOO	APOO
ARNTL	2.5357596	0.65285149	0	NA	ARNTL	ARNTL
CACNA2D3	-5.0183639	2.37311616	0	NA	CACNA2D3	CACNA2D3
CBLN1	-7.7041147	4.80719481	0	NA	CBLN1	CBLN1
CCDC109A	-2.989666	1.63539425	0	NA	CCDC109A	CCDC109A
CDH12	-3.0931634	1.70193601	0	NA	CDH12	CDH12
CDH9	3.3472538	0.50238639	0	NA	CDH9	CDH9
CEP120	-2.8706623	1.55083986	0	NA	CEP120	CEP120
CGRRF1	-4.1255188	2.23860247	0	NA	CGRRF1	CGRRF1

CNTN3	-5.1226552	2.1416389	0	NA	CNTN3	CNTN3
COX7A1	3.6220653	0.49840726	0	NA	COX7A1	COX7A1
CPNE8	-7.9019211	5.45090012	0	NA	CPNE8	CPNE8
CTNNBL1	-5.1701204	2.60508807	0	NA	CTNNBL1	CTNNBL1
CTXN3	-3.1547723	2.02249669	0	NA	CTXN3	CTXN3
CXorf1	3.5295083	0.5214482	0	NA	CXorf1	CXorf1
DACH2	5.3983733	0.39389626	0	NA	DACH2	DACH2
DACT1	3.7612088	0.6514341	0	NA	DACT1	DACT1
DGKI	2.8821568	0.44509385	0	NA	DGKI	DGKI
DLG1	3.1684757	0.60089689	0	NA	DLG1	DLG1
DOK5	3.4689382	0.68277451	0	NA	DOK5	DOK5
DYRK2	2.3302515	0.68671835	0	NA	DYRK2	DYRK2
EPHA4	-2.3288721	1.52144273	0	NA	EPHA4	EPHA4
FAM19A5	-2.2537914	1.36434919	0	NA	FAM19A5	FAM19A5
FAM5B	-2.7524322	1.60087371	0	NA	FAM5B	FAM5B
FGF12	-3.1985688	1.60028385	0	NA	FGF12	FGF12
FGF14	-4.161336	1.89013512	0	NA	FGF14	FGF14
FLJ43390	-9.7499031	5.56194442	0	NA	FLJ43390	FLJ43390
FLRT3	-6.0329061	2.31581298	0	NA	FLRT3	FLRT3
FRMPD4	2.6700364	0.72369361	0	NA	FRMPD4	FRMPD4
GABRA1	-2.4861275	1.5458172	0	NA	GABRA1	GABRA1
GABRA5	4.1635031	0.57212962	0	NA	GABRA5	GABRA5
GABRG2	-2.500529	1.35616898	0	NA	GABRG2	GABRG2
GAS1	-2.8207017	1.43925682	0	NA	GAS1	GAS1
GOLSYN	3.3184939	0.65282491	0	NA	GOLSYN	GOLSYN
GRB14	2.5728727	0.49303609	0	NA	GRB14	GRB14
H2AFY2	-2.909317	1.40106706	0	NA	H2AFY2	H2AFY2
HLF	4.0234794	0.4754812	0	NA	HLF	HLF
ID2	3.8786506	0.44944894	0	NA	ID2	ID2
IGFBP4	2.3828134	0.68418134	0	NA	IGFBP4	IGFBP4
IGFBP5	-2.834741	1.61843461	0	NA	IGFBP5	IGFBP5
IGSF11	-7.5561874	3.09731621	0	NA	IGSF11	IGSF11
KIAA0182	2.8095351	0.6575132	0	NA	KIAA0182	KIAA0182
KLF6	-2.7404503	1.42932138	0	NA	KLF6	KLF6
LGALS8	2.2259948	0.72480252	0	NA	LGALS8	LGALS8
LHFP	3.3336246	0.60532507	0	NA	LHFP	LHFP
LMO3	-2.4423344	1.621965	0	NA	LMO3	LMO3
LMO4	-5.4333042	2.09762329	0	NA	LMO4	LMO4
LOC150568	-4.5808282	2.16132196	0	NA	LOC150568	LOC150568
LOC283392	3.2109194	0.58737246	0	NA	LOC283392	LOC283392
LOC645323	2.2641538	0.74917028	0	NA	LOC645323	LOC645323
LOC653786	2.4826133	0.66960833	0	NA	LOC653786	LOC653786
LRRTM4	5.9239675	0.23561301	0	NA	LRRTM4	LRRTM4
MAMLD1	-2.732106	1.53315954	0	NA	MAMLD1	MAMLD1
MARCH3	-5.561013	1.98423491	0	NA	MARCH3	MARCH3
MAST4	-4.5965819	1.89897414	0	NA	MAST4	MAST4
MC4R	-2.7448307	2.07739068	0	NA	MC4R	MC4R
MDGA1	-2.3368477	1.47049165	0	NA	MDGA1	MDGA1
MID1	3.3365625	0.56004233	0	NA	MID1	MID1
MOSC1	-2.4501897	1.4052336	0	NA	MOSC1	MOSC1
AADAT	-2.5028615	1.32458137	0	NA	NA	AADAT
ABCA1	-3.0361926	1.58268171	0	NA	NA	ABCA1
ACTN2	5.7230026	0.4636697	0	NA	NA	ACTN2
ACVR1C	4.1708629	0.43979572	0	NA	NA	ACVR1C
ADAMTS3	-2.4132743	1.48483016	0	NA	NA	ADAMTS3
ADCY8	-7.3811571	3.68678064	0	NA	NA	ADCY8
ADI1	2.2650305	0.75310026	0	NA	NA	ADI1
AK3L1	-2.7061578	1.72460433	0	NA	NA	AK3L1

ANKH	2.6719111	0.68214739	0	NA	NA	ANKH
ANKRD57	3.0675827	0.64815372	0	NA	NA	ANKRD57
ANKS1B	5.4887345	0.46381882	0	NA	NA	ANKS1B
ANO3	2.3646069	0.48056189	0	NA	NA	ANO3
ANO4	-3.3417516	1.52520784	0	NA	NA	ANO4
ANTXR2	3.9176954	0.58369719	0	NA	NA	ANTXR2
ARHGAP6	-3.195736	1.87980994	0	NA	NA	ARHGAP6
ARHGEF6	2.8277758	0.64287333	0	NA	NA	ARHGEF6
ARL4A	2.511116	0.63345231	0	NA	NA	ARL4A
ASAH1	2.5440324	0.73396012	0	NA	NA	ASAH1
ASCL1	4.3720168	0.50310044	0	NA	NA	ASCL1
ATP8A2	-2.771165	1.51469201	0	NA	NA	ATP8A2
B3GALT2	3.1860665	0.64355277	0	NA	NA	B3GALT2
B3GAT2	-2.2564059	1.50607282	0	NA	NA	B3GAT2
B3GNT2	-2.2363781	1.33150078	0	NA	NA	B3GNT2
BAMBI	-2.5486832	1.74619574	0	NA	NA	BAMBI
BARD1	3.8839376	0.39442453	0	NA	NA	BARD1
BATF3	2.2609024	0.66619292	0	NA	NA	BATF3
BDNF	-6.0284835	4.73059426	0	NA	NA	BDNF
BEND6	2.719765	0.6260379	0	NA	NA	BEND6
BHLHE40	2.4061711	0.67549028	0	NA	NA	BHLHE40
C14orf132	4.9873905	0.49423025	0	NA	NA	C14orf132
C16orf61	-4.4408933	1.96458039	0	NA	NA	C16orf61
C18orf1	2.6272125	0.72658286	0	NA	NA	C18orf1
C1orf198	-2.5560951	1.44208311	0	NA	NA	C1orf198
C3orf59	-3.6667364	1.71843955	0	NA	NA	C3orf59
C6orf141	-2.511954	1.54599037	0	NA	NA	C6orf141
CALB1	-10.470768	11.6676061	0	NA	NA	CALB1
CAP2	2.3484789	0.74445997	0	NA	NA	CAP2
CBLN2	-14.554868	11.2565275	0	NA	NA	CBLN2
CCDC109B	3.0258479	0.54338818	0	NA	NA	CCDC109B
CDCA7	2.3297026	0.73081932	0	NA	NA	CDCA7
CDCA7L	-3.204917	1.82154463	0	NA	NA	CDCA7L
CDH3	2.8363774	0.6615742	0	NA	NA	CDH3
CENPF	-2.9824191	1.57276807	0	NA	NA	CENPF
CHRDL1	-4.5280955	2.8838507	0	NA	NA	CHRDL1
CHST8	2.9652964	0.48824292	0	NA	NA	CHST8
CLDN1	-6.6031156	2.44678317	0	NA	NA	CLDN1
CNIH3	-3.9901337	1.71872752	0	NA	NA	CNIH3
CNTNAP2	-5.1222137	3.34073761	0	NA	NA	CNTNAP2
COL12A1	-2.5600556	1.45817607	0	NA	NA	COL12A1
COMMD2	2.6738365	0.72015488	0	NA	NA	COMMD2
CRISPLD1	-2.8583041	1.65098592	0	NA	NA	CRISPLD1
CSRP2	-5.7836768	2.30538505	0	NA	NA	CSRP2
CUX1	8.0750155	0.33967862	0	NA	NA	CUX1
CXCR4	2.4724394	0.73913062	0	NA	NA	CXCR4
CXXC4	-3.9002226	1.68344979	0	NA	NA	CXXC4
CXXC5	-5.7185821	2.53493779	0	NA	NA	CXXC5
CYP26A1	-2.9104637	2.07047558	0	NA	NA	CYP26A1
DERA	-3.2478245	1.90395366	0	NA	NA	DERA
DNER	2.2646872	0.67573729	0	NA	NA	DNER
DUSP10	-4.7462373	1.89223447	0	NA	NA	DUSP10
DUSP4	-4.5331275	2.38044825	0	NA	NA	DUSP4
EDIL3	2.4718718	0.71487974	0	NA	NA	EDIL3
EFR3A	-2.5473489	1.48864717	0	NA	NA	EFR3A
EMX2	-3.67376	2.37966598	0	NA	NA	EMX2
EPCAM	3.3607583	0.567492	0	NA	NA	EPCAM
EPHA7	2.3254158	0.64339145	0	NA	NA	EPHA7

EPS15	-2.380353	1.48687855	0	NA	NA	EPS15
F3	-2.1747337	1.73215267	0	NA	NA	F3
FAM59A	-2.4934649	1.78462466	0	NA	NA	FAM59A
FERMT2	4.2632045	0.48073451	0	NA	NA	FERMT2
FGFBP3	5.9094274	0.39665838	0	NA	NA	FGFBP3
FHDC1	-6.7100988	2.52361319	0	NA	NA	FHDC1
FJX1	5.5721969	0.39617966	0	NA	NA	FJX1
FLJ11235	2.3242112	0.67035538	0	NA	NA	FLJ11235
FOXP1	2.5735375	0.71489822	0	NA	NA	FOXP1
FRZB	2.8764855	0.66639767	0	NA	NA	FRZB
FSTL5	-9.134059	5.14415268	0	NA	NA	FSTL5
FZD4	-2.5504763	1.47882771	0	NA	NA	FZD4
GAB1	2.4348477	0.72772467	0	NA	NA	GAB1
GABRG1	3.8878003	0.50753413	0	NA	NA	GABRG1
GALNT2	-4.2925877	1.95474348	0	NA	NA	GALNT2
GCG	-4.5083529	2.3913353	0	NA	NA	GCG
GHR	2.8771158	0.43728712	0	NA	NA	GHR
GLT25D2	-3.7023891	1.66361917	0	NA	NA	GLT25D2
GPHN	2.5740778	0.72677534	0	NA	NA	GPHN
GPR22	3.3248308	0.47835698	0	NA	NA	GPR22
GPR37	-2.8484793	1.64943831	0	NA	NA	GPR37
GPR88	-3.4255545	2.18844425	0	NA	NA	GPR88
GPSM2	-3.4162766	2.69405811	0	NA	NA	GPSM2
GREM1	-2.8864642	2.73409001	0	NA	NA	GREM1
GREM2	-4.9255321	4.01956006	0	NA	NA	GREM2
HCRTR2	-2.6419059	1.7646649	0	NA	NA	HCRTR2
HEY1	-3.7913367	1.48994671	0	NA	NA	HEY1
HIP1R	-2.1829824	1.28957466	0	NA	NA	HIP1R
HS3ST1	-2.2006599	1.42280822	0	NA	NA	HS3ST1
HS6ST3	-3.6617101	2.5215554	0	NA	NA	HS6ST3
HSPA12A	-4.2211371	2.06573928	0	NA	NA	HSPA12A
HTR1A	3.8667755	0.55382899	0	NA	NA	HTR1A
HUNK	-3.5936681	1.62480292	0	NA	NA	HUNK
IL7	-4.0113273	3.62954247	0	NA	NA	IL7
INTS12	-2.4877879	1.36118535	0	NA	NA	INTS12
IRS2	-3.4481015	1.49754195	0	NA	NA	IRS2
ITGA2	2.7273643	0.64873872	0	NA	NA	ITGA2
ITGA9	-2.8155154	1.48817905	0	NA	NA	ITGA9
ITGAV	2.574707	0.67811483	0	NA	NA	ITGAV
ITPR1	3.6500924	0.6397888	0	NA	NA	ITPR1
JMJD1C	2.5771635	0.67697228	0	NA	NA	JMJD1C
JPH1	-3.2960068	1.56100122	0	NA	NA	JPH1
KCNA5	-5.2899544	2.51664648	0	NA	NA	KCNA5
KCNC2	-2.9617191	1.43705499	0	NA	NA	KCNC2
KCNJ6	-5.6306064	2.58105401	0	NA	NA	KCNJ6
KCNK9	-3.5810556	1.81798244	0	NA	NA	KCNK9
KCNT2	2.3088159	0.53576416	0	NA	NA	KCNT2
KIAA0319	3.0140926	0.65502347	0	NA	NA	KIAA0319
KIAA0895	4.4775793	0.41370822	0	NA	NA	KIAA0895
KIAA1239	3.8815334	0.58276574	0	NA	NA	KIAA1239
KIAA1377	-5.0111275	2.24041945	0	NA	NA	KIAA1377
KIAA1804	3.1060298	0.62349667	0	NA	NA	KIAA1804
KIT	-3.1769796	1.63754001	0	NA	NA	KIT
KITLG	2.7996099	0.49657375	0	NA	NA	KITLG
KL	2.5219389	0.67779547	0	NA	NA	KL
KLHL32	5.6726516	0.24577443	0	NA	NA	KLHL32
KLHL8	3.0656515	0.69557702	0	NA	NA	KLHL8
KRT31	-3.8344871	2.18590383	0	NA	NA	KRT31

LDB2	-2.8931194	1.4399603	0	NA	NA	LDB2
LDLRAD3	4.0334391	0.51008571	0	NA	NA	LDLRAD3
LHFPL2	-4.0089999	1.78865557	0	NA	NA	LHFPL2
LNX2	-2.7594468	1.56511014	0	NA	NA	LNX2
LOC286002	6.6399202	0.37730405	0	NA	NA	LOC286002
LOX	4.6566289	0.52134261	0	NA	NA	LOX
LPL	2.7815804	0.71377351	0	NA	NA	LPL
LRRC3B	-4.8019253	1.97409064	0	NA	NA	LRRC3B
LRRTM2	-2.4679681	1.4276446	0	NA	NA	LRRTM2
LUZP2	2.5089308	0.46101732	0	NA	NA	LUZP2
MAL2	-2.6502091	1.76772617	0	NA	NA	MAL2
MAP3K5	-6.2555867	3.27948624	0	NA	NA	MAP3K5
MCHR2	-3.564827	1.90786707	0	NA	NA	MCHR2
MCTP1	3.6104371	0.58045841	0	NA	NA	MCTP1
ME3	-2.3898726	1.31149488	0	NA	NA	ME3
MEGF10	-7.2306862	3.23291631	0	NA	NA	MEGF10
MEGF11	-2.9538663	1.677664	0	NA	NA	MEGF11
MET	3.6942663	0.56200753	0	NA	NA	MET
MIPEP	-6.0186078	2.26883941	0	NA	NA	MIPEP
MOCS2	2.6924114	0.68164569	0	NA	NA	MOCS2
MPP6	2.8271169	0.45577482	0	NA	NA	MPP6
MRAP2	2.3949268	0.51337438	0	NA	NA	MRAP2
MTFR1	2.241794	0.74725754	0	NA	NA	MTFR1
MYO16	-3.0542861	1.60213646	0	NA	NA	MYO16
MYO1E	2.4481187	0.67850105	0	NA	NA	MYO1E
NCOA2	2.3244361	0.65543156	0	NA	NA	NCOA2
NEBL	-3.959623	1.81477552	0	NA	NA	NEBL
NEDD9	-2.4947402	1.5534365	0	NA	NA	NEDD9
NEFL	6.1790036	0.31463525	0	NA	NA	NEFL
NEUROD1	-7.5610329	4.91363672	0	NA	NA	NEUROD1
NFE2L3	3.2905816	0.45771202	0	NA	NA	NFE2L3
NME5	-2.2837135	1.57292326	0	NA	NA	NME5
NPAS2	2.4130014	0.7114197	0	NA	NA	NPAS2
NPY	10.487701	0.15260943	0	NA	NA	NPY
NPY1R	-2.8091789	1.55164331	0	NA	NA	NPY1R
NR2F1	4.5333847	0.4904898	0	NA	NA	NR2F1
NRIP1	-3.2856362	1.71512376	0	NA	NA	NRIP1
NRN1	2.645197	0.61308738	0	NA	NA	NRN1
NTN4	-4.3353016	2.12969222	0	NA	NA	NTN4
NTNG1	2.5601591	0.6424647	0	NA	NA	NTNG1
NUP93	-3.4552735	1.54554115	0	NA	NA	NUP93
OSBPL6	2.5945569	0.64613791	0	NA	NA	OSBPL6
OTOR	3.3941304	0.32209121	0	NA	NA	OTOR
P2RY12	2.5446371	0.62162186	0	NA	NA	P2RY12
PACSIN2	-2.7839704	1.44877693	0	NA	NA	PACSIN2
PAICS	3.8589303	0.6145106	0	NA	NA	PAICS
PAK7	4.4807926	0.58162497	0	NA	NA	PAK7
PALMD	-3.1556924	1.82573813	0	NA	NA	PALMD
PAPPA2	3.3547903	0.67821402	0	NA	NA	PAPPA2
PCBD1	-2.4006729	1.43434157	0	NA	NA	PCBD1
PCOLCE2	2.5204469	0.58040551	0	NA	NA	PCOLCE2
PCSK2	11.205976	0.12589846	0	NA	NA	PCSK2
PDE8B	-2.9336571	1.67372112	0	NA	NA	PDE8B
PDZRN3	2.3995817	0.66270499	0	NA	NA	PDZRN3
PENK	-6.7368517	6.43723769	0	NA	NA	PENK
PGM1	-2.8866915	1.47795452	0	NA	NA	PGM1
PKP4	-2.4639251	1.3398196	0	NA	NA	PKP4
PLCXD3	4.2669402	0.47628416	0	NA	NA	PLCXD3

PLEKHG1	-3.4388593	2.35048104	0	NA	NA	PLEKHG1
POPDC3	-4.034346	2.24011236	0	NA	NA	POPDC3
POU6F2	-2.6805439	1.63663371	0	NA	NA	POU6F2
PPEF1	8.0271043	0.3765052	0	NA	NA	PPEF1
PROK2	3.9923299	0.40336252	0	NA	NA	PROK2
PRSS23	-8.5327816	3.48366176	0	NA	NA	PRSS23
PRUNE2	-2.1695295	1.38494176	0	NA	NA	PRUNE2
PTDSS1	3.5101653	0.65010501	0	NA	NA	PTDSS1
PTGFR	-4.2996081	3.03066476	0	NA	NA	PTGFR
PTPN13	3.4056784	0.52764387	0	NA	NA	PTPN13
PTPN2	2.2949353	0.72235209	0	NA	NA	PTPN2
PTPRT	-2.748046	1.71787085	0	NA	NA	PTPRT
PTX3	-5.0063375	2.69234918	0	NA	NA	PTX3
RASGRP1	8.1145198	0.18252844	0	NA	NA	RASGRP1
RASL11B	4.6466211	0.54763951	0	NA	NA	RASL11B
RBP1	-2.2620478	1.66082427	0	NA	NA	RBP1
RCAN1	-3.188018	1.56161412	0	NA	NA	RCAN1
RCAN2	-6.3698221	4.29708113	0	NA	NA	RCAN2
RFK	-3.0766184	1.42806894	0	NA	NA	RFK
RFTN1	-2.4595346	1.37948102	0	NA	NA	RFTN1
RGS8	-2.4556735	1.75033938	0	NA	NA	RGS8
RGS9	-2.3931161	1.54817406	0	NA	NA	RGS9
RPL23AP32	2.2281744	0.68807363	0	NA	NA	RPL23AP32
RPRML	3.4553787	0.39643637	0	NA	NA	RPRML
RRAS2	-2.2138059	1.43616073	0	NA	NA	RRAS2
RXFP1	-9.3099821	9.18590416	0	NA	NA	RXFP1
RXRG	-5.5586275	2.9990711	0	NA	NA	RXRG
RYR3	5.4519481	0.38078123	0	NA	NA	RYR3
SAMD13	-3.0569876	1.73761783	0	NA	NA	SAMD13
SAV1	4.2713176	0.49855907	0	NA	NA	SAV1
SDC2	2.240968	0.57465879	0	NA	NA	SDC2
SEMA3E	-2.3056371	1.63520145	0	NA	NA	SEMA3E
SERPINI1	3.7436343	0.60735495	0	NA	NA	SERPINI1
SEZ6L	2.383783	0.70083586	0	NA	NA	SEZ6L
SGCG	-3.5579458	2.88288651	0	NA	NA	SGCG
SGEF	3.0105396	0.51600388	0	NA	NA	SGEF
SGK1	4.5344644	0.54497708	0	NA	NA	SGK1
SH3D19	-2.1801666	1.33741731	0	NA	NA	SH3D19
SHISA2	5.6562868	0.33524399	0	NA	NA	SHISA2
SLC12A2	-2.2112538	1.5342079	0	NA	NA	SLC12A2
SLC17A7	3.8280472	0.65556488	0	NA	NA	SLC17A7
SLC35F2	-2.9960979	1.96965228	0	NA	NA	SLC35F2
SLITRK3	-4.2346195	2.044461	0	NA	NA	SLITRK3
SLITRK4	2.2845762	0.74566532	0	NA	NA	SLITRK4
SMC4	3.2304147	0.66233649	0	NA	NA	SMC4
SNCAIP	-2.3999304	1.44456808	0	NA	NA	SNCAIP
SOCS2	-2.3828863	1.62618978	0	NA	NA	SOCS2
SOX9	12.91514	0.11641769	0	NA	NA	SOX9
SPIRE1	3.7605242	0.60874536	0	NA	NA	SPIRE1
SRI	-2.2418818	1.30176061	0	NA	NA	SRI
SSTR1	2.2225815	0.45025199	0	NA	NA	SSTR1
ST3GAL1	2.3076774	0.65009901	0	NA	NA	ST3GAL1
ST6GAL2	4.5318538	0.44398221	0	NA	NA	ST6GAL2
STARD4	3.0072284	0.63499894	0	NA	NA	STARD4
STK31	3.157706	0.29964372	0	NA	NA	STK31
SULF1	-4.571398	2.76779119	0	NA	NA	SULF1
SVIP	3.1539867	0.63591265	0	NA	NA	SVIP
SYNPR	2.2112118	0.6402151	0	NA	NA	SYNPR

TACR2	2.2351101	0.65506084	0	NA	NA	TACR2
TAL2	-2.8474592	1.98116717	0	NA	NA	TAL2
TBC1D8	3.2520082	0.56460283	0	NA	NA	TBC1D8
TCF7L2	-2.7195924	1.46882282	0	NA	NA	TCF7L2
TDRD7	2.4033651	0.71213719	0	NA	NA	TDRD7
TGFA	3.641964	0.59550569	0	NA	NA	TGFA
TGFB2	5.2878672	0.38054757	0	NA	NA	TGFB2
TGFBR1	2.4199176	0.6526334	0	NA	NA	TGFBR1
THSD7A	3.3056219	0.55085256	0	NA	NA	THSD7A
TLK1	3.19175	0.6193214	0	NA	NA	TLK1
TMEM200A	4.5987098	0.48562263	0	NA	NA	TMEM200A
TMEM35	-2.2798528	1.40446853	0	NA	NA	TMEM35
TNFRSF21	-2.2771288	1.33797149	0	NA	NA	TNFRSF21
TNKS2	2.4188874	0.6724906	0	NA	NA	TNKS2
TP53I3	-4.5234967	2.3275304	0	NA	NA	TP53I3
TRPC4	5.6127943	0.39063655	0	NA	NA	TRPC4
TRPC7	3.4270364	0.44069231	0	NA	NA	TRPC7
TSHZ1	-2.7662182	1.5240847	0	NA	NA	TSHZ1
TSHZ3	-4.8185133	2.42675149	0	NA	NA	TSHZ3
UFM1	2.5808817	0.67122313	0	NA	NA	UFM1
UNC13C	5.2100561	0.37864663	0	NA	NA	UNC13C
USP46	2.7529312	0.69716762	0	NA	NA	USP46
UST	-2.3511955	1.63237533	0	NA	NA	UST
WIF1	2.9832351	0.33811108	0	NA	NA	WIF1
WNT7B	3.4683119	0.48789458	0	NA	NA	WNT7B
XK	2.2480153	0.63144829	0	NA	NA	XK
ZNF114	-3.8851986	2.8202289	0	NA	NA	ZNF114
ZNF385B	3.1447634	0.46473021	0	NA	NA	ZNF385B
ZNF503	2.7163132	0.66857216	0	NA	NA	ZNF503
ZNF618	2.4840875	0.73010905	0	NA	NA	ZNF618
NDP	-4.9907327	3.07052035	0	NA	NDP	NDP
NOG	-3.1842631	2.05302024	0	NA	NOG	NOG
NR3C1	3.9886444	0.55590161	0	NA	NR3C1	NR3C1
NR3C2	6.2948111	0.35436521	0	NA	NR3C2	NR3C2
NR4A2	5.3728796	0.31034033	0	NA	NR4A2	NR4A2
NTNG2	5.0387291	0.27228346	0	NA	NTNG2	NTNG2
NXPH3	3.2378358	0.63766383	0	NA	NXPH3	NXPH3
ODZ4	2.4831271	0.71491135	0	NA	ODZ4	ODZ4
OLFM3	-2.3427178	1.60992788	0	NA	OLFM3	OLFM3
PBX3	2.6304097	0.71030199	0	NA	PBX3	PBX3
PCDH17	-5.0671224	2.34152922	0	NA	PCDH17	PCDH17
PCDH18	-2.4106968	1.50798322	0	NA	PCDH18	PCDH18
PCSK9	2.8585448	0.66795293	0	NA	PCSK9	PCSK9
PDE4DIP	2.650362	0.69291976	0	NA	PDE4DIP	PDE4DIP
PDZRN4	-5.4476486	2.63707924	0	NA	PDZRN4	PDZRN4
PHLDA1	-4.0501691	1.65922624	0	NA	PHLDA1	PHLDA1
PIK3AP1	2.7624911	0.64434479	0	NA	PIK3AP1	PIK3AP1
PPARGC1A	2.5259817	0.69605157	0	NA	PPARGC1A	PPARGC1A
PSD2	-2.1802477	1.30380301	0	NA	PSD2	PSD2
PTER	-3.937988	2.21395294	0	NA	PTER	PTER
PTPRK	4.7632103	0.3977537	0	NA	PTPRK	PTPRK
QDPR	-2.1979566	1.33072736	0	NA	QDPR	QDPR
RAB7L1	3.8609759	0.56498331	0	NA	RAB7L1	RAB7L1
RGS20	2.36348	0.71130303	0	NA	RGS20	RGS20
RNF182	4.7106408	0.47962861	0	NA	RNF182	RNF182
ROBO2	5.3895655	0.46474463	0	NA	ROBO2	ROBO2
RPRM	-3.0311581	2.03229418	0	NA	RPRM	RPRM
SFRP1	-3.1715505	1.60973547	0	NA	SFRP1	SFRP1

SFRP2	-2.9745118	1.69062342	0	NA	SFRP2	SFRP2
SLC6A15	2.7910928	0.67529137	0	NA	SLC6A15	SLC6A15
SLCO3A1	-2.3528977	1.73020418	0	NA	SLCO3A1	SLCO3A1
SLIT2	-3.0879381	1.56586266	0	NA	SLIT2	SLIT2
SORCS1	4.3988439	0.48451882	0	NA	SORCS1	SORCS1
SPON1	5.0932677	0.30520412	0	NA	SPON1	SPON1
ST8SIA3	-2.4241874	1.47965344	0	NA	ST8SIA3	ST8SIA3
STXBP6	-7.5596973	3.29939452	0	NA	STXBP6	STXBP6
SULF2	-4.7353515	2.57250946	0	NA	SULF2	SULF2
SYNE1	2.7432808	0.71460998	0	NA	SYNE1	SYNE1
SYNM	-3.2827036	1.63034073	0	NA	SYNM	SYNM
TLE1	9.2333799	0.33331922	0	NA	TLE1	TLE1
TMCC2	2.363984	0.72802771	0	NA	TMCC2	TMCC2
TOX	-6.2470255	3.16050207	0	NA	TOX	TOX
TPBG	-2.3870222	1.57377889	0	NA	TPBG	TPBG
TRH	-4.3485877	1.92544255	0	NA	TRH	TRH
WBSCR17	-3.4505242	1.54274478	0	NA	WBSCR17	WBSCR17
WNT11	-2.185204	1.57874503	0	NA	WNT11	WNT11
ZNF238	2.8390867	0.596913	0	NA	ZNF238	ZNF238
ZNF423	-6.9022481	4.59746511	0	NA	ZNF423	ZNF423
C5orf4	2.0449505	0.75038304	0.11329523	NA	C5orf4	C5orf4
DPF3	-1.9948572	1.32280327	0.11329523	NA	DPF3	DPF3
EIF3E	2.1041583	0.79564492	0.11329523	NA	EIF3E	EIF3E
GGTA1	-2.1053034	1.54639322	0.11329523	NA	GGTA1	GGTA1
GOLGA7B	-2.1550231	1.44318056	0.11329523	NA	GOLGA7B	GOLGA7B
GPAM	2.140471	0.68699697	0.11329523	NA	GPAM	GPAM
GRIK2	-2.1000341	1.32784063	0.11329523	NA	GRIK2	GRIK2
GRM7	-1.9945815	1.33335681	0.11329523	NA	GRM7	GRM7
HSPB3	-2.111997	1.51116191	0.11329523	NA	HSPB3	HSPB3
HTR7	2.0742972	0.71424476	0.11329523	NA	HTR7	HTR7
ABHD6	2.0503227	0.76084925	0.11329523	NA	NA	ABHD6
ADIPOR2	2.1669984	0.75632768	0.11329523	NA	NA	ADIPOR2
ADSSL1	-2.024042	1.31061038	0.11329523	NA	NA	ADSSL1
AGFG1	2.1688378	0.76608477	0.11329523	NA	NA	AGFG1
AJAP1	-2.0384512	1.31571584	0.11329523	NA	NA	AJAP1
AKR1C1	-2.1625288	1.35573308	0.11329523	NA	NA	AKR1C1
ANKRD29	-2.0779442	1.53237741	0.11329523	NA	NA	ANKRD29
AP1S2	2.1771745	0.7818732	0.11329523	NA	NA	AP1S2
BVES	-2.0094566	1.44099132	0.11329523	NA	NA	BVES
C13orf36	-2.0945015	2.13159181	0.11329523	NA	NA	C13orf36
C1orf173	2.1605387	0.65197987	0.11329523	NA	NA	C1orf173
C3orf50	-2.036135	1.98550562	0.11329523	NA	NA	C3orf50
C7orf36	-2.157551	1.48566933	0.11329523	NA	NA	C7orf36
CBARA1	-2.0722511	1.36405196	0.11329523	NA	NA	CBARA1
DRD1	-2.1159006	1.84786237	0.11329523	NA	NA	DRD1
EBPL	-2.0174033	1.31289045	0.11329523	NA	NA	EBPL
EML6	2.0822228	0.71419206	0.11329523	NA	NA	EML6
EMX1	2.0036574	0.73095406	0.11329523	NA	NA	EMX1
FRY	2.1460488	0.74024331	0.11329523	NA	NA	FRY
GGCT	-2.0093063	1.27766155	0.11329523	NA	NA	GGCT
GRIK1	2.0950291	0.68395149	0.11329523	NA	NA	GRIK1
GRM5	-2.0873797	1.32209824	0.11329523	NA	NA	GRM5
HAS2	2.0574799	0.6831462	0.11329523	NA	NA	HAS2
LOC387763	2.0187893	0.76468361	0.11329523	NA	NA	LOC387763
LRRC7	2.1384318	0.73077893	0.11329523	NA	NA	LRRC7
MYF6	-2.0699629	1.36606021	0.11329523	NA	NA	MYF6
NECAB1	2.0109467	0.68407983	0.11329523	NA	NA	NECAB1
NEDD4	2.1359013	0.73156625	0.11329523	NA	NA	NEDD4

NUAK2	2.0193351	0.72768633	0.11329523	NA	NA	NUAK2
OMG	2.0489617	0.72221266	0.11329523	NA	NA	OMG
PDGFD	2.180256	0.67258826	0.11329523	NA	NA	PDGFD
PDZD2	2.1692008	0.69590969	0.11329523	NA	NA	PDZD2
PGBD5	-2.0289311	1.31080757	0.11329523	NA	NA	PGBD5
PTPRZ1	2.1107313	0.75631262	0.11329523	NA	NA	PTPRZ1
RAB11A	-2.0923258	1.24803638	0.11329523	NA	NA	RAB11A
REM2	-2.1261275	1.56858986	0.11329523	NA	NA	REM2
RFC3	2.091419	0.71567047	0.11329523	NA	NA	RFC3
RSPH9	2.0134479	0.74456472	0.11329523	NA	NA	RSPH9
SERPINB6	-2.0431848	1.28622809	0.11329523	NA	NA	SERPINB6
SERTAD2	2.1096883	0.74027596	0.11329523	NA	NA	SERTAD2
SFRS2B	-2.1154615	1.27174603	0.11329523	NA	NA	SFRS2B
SLC17A5	-2.1683251	1.35369328	0.11329523	NA	NA	SLC17A5
SLC26A8	2.0197541	0.66891371	0.11329523	NA	NA	SLC26A8
SLC27A2	2.1396481	0.7838011	0.11329523	NA	NA	SLC27A2
SMPDL3A	2.1051156	0.710081	0.11329523	NA	NA	SMPDL3A
SNHG8	2.029819	0.76749506	0.11329523	NA	NA	SNHG8
SV2B	-2.1380977	1.40507555	0.11329523	NA	NA	SV2B
TAF4B	2.163867	0.73515042	0.11329523	NA	NA	TAF4B
TAPT1	-2.0881596	1.24815485	0.11329523	NA	NA	TAPT1
SATB2	2.0138674	0.6652304	0.11329523	NA	SATB2	SATB2
SH3BP4	-2.1323932	1.35398485	0.11329523	NA	SH3BP4	SH3BP4
TARBP1	2.0160223	0.77167533	0.11329523	NA	TARBP1	TARBP1
TBX21	-2.0951755	1.50634971	0.11329523	NA	TBX21	TBX21
ACVR1	1.9016813	0.74909317	0.19206239	NA	ACVR1	ACVR1
AKAP13	1.8672226	0.77684803	0.19206239	NA	AKAP13	AKAP13
ASS1	-1.8757098	1.36348358	0.19206239	NA	ASS1	ASS1
C9orf45	1.9496523	0.75910403	0.19206239	NA	C9orf45	C9orf45
CYYR1	-1.8344824	1.39509513	0.19206239	NA	CYYR1	CYYR1
DPP3	-1.8794705	1.2852484	0.19206239	NA	DPP3	DPP3
DPYD	1.91158	0.75716218	0.19206239	NA	DPYD	DPYD
HS3ST2	-1.9807592	1.51306092	0.19206239	NA	HS3ST2	HS3ST2
KLHL14	-1.8384008	1.35188201	0.19206239	NA	KLHL14	KLHL14
ADC	1.882217	0.74554495	0.19206239	NA	NA	ADC
ADCY1	1.8937999	0.78767214	0.19206239	NA	NA	ADCY1
AKAP7	-1.9212017	1.33142677	0.19206239	NA	NA	AKAP7
AKR1B1	-1.8873825	1.26669329	0.19206239	NA	NA	AKR1B1
ALDH7A1	-1.9677894	1.56588168	0.19206239	NA	NA	ALDH7A1
ANK2	1.9805793	0.68917144	0.19206239	NA	NA	ANK2
ANKRD6	-1.9501876	1.4522325	0.19206239	NA	NA	ANKRD6
APBB2	-1.9369471	1.33781927	0.19206239	NA	NA	APBB2
ARHGAP12	1.9922063	0.64834482	0.19206239	NA	NA	ARHGAP12
ARHGAP18	1.968179	0.70485716	0.19206239	NA	NA	ARHGAP18
ATP2C1	1.8198191	0.76723843	0.19206239	NA	NA	ATP2C1
BAI2	1.9038778	0.68664261	0.19206239	NA	NA	BAI2
C10orf58	-1.9142667	1.24940341	0.19206239	NA	NA	C10orf58
C11orf75	-1.9505339	1.68916961	0.19206239	NA	NA	C11orf75
C14orf37	1.9223885	0.66899015	0.19206239	NA	NA	C14orf37
C2orf3	1.8417981	0.75585756	0.19206239	NA	NA	C2orf3
C2orf7	-1.8899315	1.31692006	0.19206239	NA	NA	C2orf7
CAMKV	1.8540275	0.77291741	0.19206239	NA	NA	CAMKV
CAV1	-1.8666365	1.46701097	0.19206239	NA	NA	CAV1
CEP135	1.8777125	0.76959028	0.19206239	NA	NA	CEP135
DEK	1.8585399	0.81623636	0.19206239	NA	NA	DEK
DENND4C	1.8540265	0.79761758	0.19206239	NA	NA	DENND4C
DYNC2H1	1.9865679	0.71849855	0.19206239	NA	NA	DYNC2H1
EIF3D	1.8358103	0.81949329	0.19206239	NA	NA	EIF3D

EPB41L3	1.819643	0.72224433	0.19206239	NA	NA	EPB41L3
FABP7	1.9955677	0.69389679	0.19206239	NA	NA	FABP7
FAM43A	-1.8784603	1.28227334	0.19206239	NA	NA	FAM43A
FRMD4B	-1.9884055	1.29900971	0.19206239	NA	NA	FRMD4B
GCA	1.9918127	0.6942364	0.19206239	NA	NA	GCA
GHRH	-1.8694463	1.32426279	0.19206239	NA	NA	GHRH
HCN1	1.8768348	0.75954513	0.19206239	NA	NA	HCN1
IGSF21	1.8198256	0.7643972	0.19206239	NA	NA	IGSF21
IMPDH2	1.8557926	0.80768111	0.19206239	NA	NA	IMPDH2
KCNAB1	-1.9093715	1.39623488	0.19206239	NA	NA	KCNAB1
KCNE1	-1.9868154	1.38190226	0.19206239	NA	NA	KCNE1
LOC100129550	1.8909605	0.74844148	0.19206239	NA	NA	LOC10012955 0
LOC100134229	-1.9667124	1.43010266	0.19206239	NA	NA	LOC10013422 9
LOC151162	-1.8938993	1.26626375	0.19206239	NA	NA	LOC151162
LRRC8C	-1.9658767	1.28337061	0.19206239	NA	NA	LRRC8C
MYB	-1.9590426	1.33546126	0.19206239	NA	NA	MYB
MYC	-1.8492218	1.26090084	0.19206239	NA	NA	MYC
N4BP2L2	1.8287236	0.76511986	0.19206239	NA	NA	N4BP2L2
NAP1L5	-1.8801924	1.29018764	0.19206239	NA	NA	NAP1L5
NPNT	-1.8799266	1.43839345	0.19206239	NA	NA	NPNT
PAG1	1.9923476	0.75355565	0.19206239	NA	NA	PAG1
PHF17	1.9406617	0.73516516	0.19206239	NA	NA	PHF17
PITPNC1	-1.9241893	1.28031768	0.19206239	NA	NA	PITPNC1
PLCXD2	-1.8698634	1.26021415	0.19206239	NA	NA	PLCXD2
RAB2B	-1.8679581	1.22997804	0.19206239	NA	NA	RAB2B
RHPN2	1.9213888	0.73483556	0.19206239	NA	NA	RHPN2
RIN2	1.9573726	0.76035393	0.19206239	NA	NA	RIN2
RP1-177G6.2	1.9769926	0.56392297	0.19206239	NA	NA	RP1-177G6.2
RPH3A	-1.8374374	1.75038429	0.19206239	NA	NA	RPH3A
RPL37	1.8538167	0.80050686	0.19206239	NA	NA	RPL37
SCNN1G	-1.9659273	1.41568303	0.19206239	NA	NA	SCNN1G
SLC46A3	-1.8978447	1.28379808	0.19206239	NA	NA	SLC46A3
SNAI2	-1.8931277	1.42988906	0.19206239	NA	NA	SNAI2
SUPT3H	-1.8828569	1.41033346	0.19206239	NA	NA	SUPT3H
SYDE2	-1.9700165	1.40108213	0.19206239	NA	NA	SYDE2
TIAM2	1.9183267	0.7270701	0.19206239	NA	NA	TIAM2
TMEM74	1.9308704	0.72485958	0.19206239	NA	NA	TMEM74
TMTC1	1.9325874	0.60091483	0.19206239	NA	NA	TMTC1
TRIM44	1.9809887	0.76321079	0.19206239	NA	NA	TRIM44
TSPAN18	-1.941617	1.23064855	0.19206239	NA	NA	TSPAN18
TSPAN6	-1.9717261	1.54699507	0.19206239	NA	NA	TSPAN6
WNT3	-1.8373978	1.28145237	0.19206239	NA	NA	WNT3
WNT5B	1.9573525	0.78654814	0.19206239	NA	NA	WNT5B
ZNF438	1.9131199	0.79043807	0.19206239	NA	NA	ZNF438
NOS1	-1.9311619	1.2985339	0.19206239	NA	NOS1	NOS1
PLOD2	1.8199163	0.63535707	0.19206239	NA	PLOD2	PLOD2
RSL24D1	1.9607116	0.76287592	0.19206239	NA	RSL24D1	RSL24D1
SNX9	1.8759538	0.72844561	0.19206239	NA	SNX9	SNX9
ZADH2	-1.9388644	1.33605933	0.19206239	NA	ZADH2	ZADH2
ZNF706	-1.8489992	1.22405684	0.19206239	NA	ZNF706	ZNF706
AMOTL1	-1.6708045	1.2233893	0.56921718	NA	AMOTL1	AMOTL1
C1orf201	1.6867839	0.76162761	0.56921718	NA	C1orf201	C1orf201
CHGB	1.6889775	0.80786526	0.56921718	NA	CHGB	CHGB
DIAPH3	-1.691133	1.316908	0.56921718	NA	DIAPH3	DIAPH3
DUSP6	-1.6648534	1.34244361	0.56921718	NA	DUSP6	DUSP6
FBXL4	1.7030863	0.74322208	0.56921718	NA	FBXL4	FBXL4

FOSL2	-1.8048882	1.39235042	0.56921718	NA	FOSL2	FOSL2
GRM3	1.7963915	0.79517321	0.56921718	NA	GRM3	GRM3
ID4	-1.714183	1.24941014	0.56921718	NA	ID4	ID4
LRRC4C	1.7723172	0.78380855	0.56921718	NA	LRRC4C	LRRC4C
MAOA	-1.7230175	1.26439033	0.56921718	NA	MAOA	MAOA
MIAT	1.6685052	0.7468721	0.56921718	NA	MIAT	MIAT
ABI2	1.8002732	0.78889966	0.56921718	NA	NA	ABI2
ADD2	1.7072329	0.79782557	0.56921718	NA	NA	ADD2
AHR	1.7311067	0.74526905	0.56921718	NA	NA	AHR
ALS2	1.730133	0.77799375	0.56921718	NA	NA	ALS2
AMMECR1	1.7897814	0.77927582	0.56921718	NA	NA	AMMECR1
ARHGEF3	-1.6834136	1.22494225	0.56921718	NA	NA	ARHGEF3
ARL4C	-1.7089553	1.21333535	0.56921718	NA	NA	ARL4C
ATP2B1	1.7101363	0.78636865	0.56921718	NA	NA	ATP2B1
B3GALTL	-1.7134436	1.54733497	0.56921718	NA	NA	B3GALTL
BCAP29	1.77772	0.79518995	0.56921718	NA	NA	BCAP29
BCAR3	1.6554876	0.78105918	0.56921718	NA	NA	BCAR3
C17orf58	-1.664373	1.25174985	0.56921718	NA	NA	C17orf58
CABP1	1.7476754	0.72210881	0.56921718	NA	NA	CABP1
CHD3	1.7570376	0.74835474	0.56921718	NA	NA	CHD3
CHRNB1	-1.6878377	1.30251577	0.56921718	NA	NA	CHRNB1
CSMD2	1.6504898	0.78251864	0.56921718	NA	NA	CSMD2
CTGF	-1.7315289	1.50144371	0.56921718	NA	NA	CTGF
DGKH	1.6708511	0.81766292	0.56921718	NA	NA	DGKH
DNAJC12	-1.779901	1.33330638	0.56921718	NA	NA	DNAJC12
E2F3	-1.7467634	1.27894087	0.56921718	NA	NA	E2F3
ECHDC3	1.7663812	0.70254225	0.56921718	NA	NA	ECHDC3
FGFR1	-1.7778665	1.21511311	0.56921718	NA	NA	FGFR1
FNDC3B	1.7206151	0.81477116	0.56921718	NA	NA	FNDC3B
FTL	-1.6927348	1.23643025	0.56921718	NA	NA	FTL
GNAL	-1.6730574	1.82837731	0.56921718	NA	NA	GNAL
GPR137C	-1.7683416	1.45479059	0.56921718	NA	NA	GPR137C
GPR63	1.7023713	0.67572853	0.56921718	NA	NA	GPR63
HEATR1	1.7673478	0.6703171	0.56921718	NA	NA	HEATR1
HSPA4L	1.7627049	0.69399936	0.56921718	NA	NA	HSPA4L
HYMAI	1.7725019	0.66603623	0.56921718	NA	NA	HYMAI
IPO8	1.7151015	0.7815551	0.56921718	NA	NA	IPO8
JAK2	1.766462	0.78345728	0.56921718	NA	NA	JAK2
KCNK2	-1.6590451	1.31587451	0.56921718	NA	NA	KCNK2
KLHL25	1.6601986	0.75803502	0.56921718	NA	NA	KLHL25
LOC401097	1.7677791	0.6990811	0.56921718	NA	NA	LOC401097
LYSMD3	1.6959089	0.74857584	0.56921718	NA	NA	LYSMD3
MAN2A1	1.7357733	0.68817559	0.56921718	NA	NA	MAN2A1
NALCN	-1.7134353	1.33890556	0.56921718	NA	NA	NALCN
NEFM	1.8104381	0.76583052	0.56921718	NA	NA	NEFM
OAT	-1.665461	1.23767024	0.56921718	NA	NA	OAT
PAQR8	-1.6793243	1.3547052	0.56921718	NA	NA	PAQR8
PCOTH	-1.7710008	1.2697666	0.56921718	NA	NA	PCOTH
PPA2	-1.716176	1.29695608	0.56921718	NA	NA	PPA2
PPP1R3C	-1.7865883	1.27858437	0.56921718	NA	NA	PPP1R3C
PRDM10	1.7214244	0.80448983	0.56921718	NA	NA	PRDM10
QPCT	-1.6989685	1.52267803	0.56921718	NA	NA	QPCT
RBM24	1.7236535	0.77326309	0.56921718	NA	NA	RBM24
RGS16	-1.6671199	1.33345675	0.56921718	NA	NA	RGS16
SAMD3	1.8075569	0.77995226	0.56921718	NA	NA	SAMD3
SCRN1	1.7554073	0.77450968	0.56921718	NA	NA	SCRN1
SEMA3C	-1.6712737	1.36815885	0.56921718	NA	NA	SEMA3C
SHB	1.7365438	0.81139146	0.56921718	NA	NA	SHB

SIPA1L2	1.7967593	0.81728709	0.56921718	NA	NA	SIPA1L2
SLC17A8	-1.6870861	1.51521774	0.56921718	NA	NA	SLC17A8
SPATA13	-1.6937885	1.32035141	0.56921718	NA	NA	SPATA13
SYT9	-1.7174943	1.24831857	0.56921718	NA	NA	SYT9
TBC1D1	-1.7283841	1.22658604	0.56921718	NA	NA	TBC1D1
TBR1	-1.7852788	1.28154788	0.56921718	NA	NA	TBR1
TNFAIP3	-1.7459981	1.72654034	0.56921718	NA	NA	TNFAIP3
UBP1	1.7354988	0.76318925	0.56921718	NA	NA	UBP1
VEPH1	-1.6686711	1.21287794	0.56921718	NA	NA	VEPH1
WWP2	-1.6644297	1.26294759	0.56921718	NA	NA	WWP2
NGB	1.6973064	0.81316304	0.56921718	NA	NGB	NGB
NR2F2	1.6810585	0.78707751	0.56921718	NA	NR2F2	NR2F2
NRXN3	-1.6779785	1.23251032	0.56921718	NA	NRXN3	NRXN3
PLXNC1	-1.7689523	1.29644548	0.56921718	NA	PLXNC1	PLXNC1
RAPGEFL1	1.7176713	0.71074745	0.56921718	NA	RAPGEFL1	RAPGEFL1
RUNX2	-1.6858028	1.28467826	0.56921718	NA	RUNX2	RUNX2
SMAD3	1.7388772	0.78593321	0.56921718	NA	SMAD3	SMAD3
ZAK	1.670217	0.71519869	0.56921718	NA	ZAK	ZAK
ZHX2	1.7521996	0.76600304	0.56921718	NA	ZHX2	ZHX2
AUH	1.5088174	0.79367185	0.88419955	NA	AUH	AUH
CDH13	1.5766612	0.71012446	0.88419955	NA	CDH13	CDH13
CLCN4	-1.6496394	1.35400775	0.88419955	NA	CLCN4	CLCN4
DCC	-1.5468003	1.25641969	0.88419955	NA	DCC	DCC
DMXL1	1.5936059	0.79464748	0.88419955	NA	DMXL1	DMXL1
DNMT3A	-1.6393412	1.26259462	0.88419955	NA	DNMT3A	DNMT3A
KBTBD8	1.5720203	0.79932808	0.88419955	NA	KBTBD8	KBTBD8
KIAA1958	-1.5262747	1.26307324	0.88419955	NA	KIAA1958	KIAA1958
MARK1	1.5520661	0.80040881	0.88419955	NA	MARK1	MARK1
MDH1	1.6458122	0.7827652	0.88419955	NA	MDH1	MDH1
MITF	1.626352	0.7826945	0.88419955	NA	MITF	MITF
MYO1B	-1.6535742	1.26905342	0.88419955	NA	MYO1B	MYO1B
AMIGO2	1.5389169	0.79046747	0.88419955	NA	NA	AMIGO2
ASPH	1.6025921	0.81486279	0.88419955	NA	NA	ASPH
BCL2	1.6262094	0.72497361	0.88419955	NA	NA	BCL2
BRAF	1.6067269	0.77384768	0.88419955	NA	NA	BRAF
C11orf87	-1.5472354	1.41698678	0.88419955	NA	NA	C11orf87
C1GALT1	-1.5028948	1.27063069	0.88419955	NA	NA	C1GALT1
C6orf138	-1.6177315	1.37118745	0.88419955	NA	NA	C6orf138
CAPZA2	1.4915659	0.82907958	0.88419955	NA	NA	CAPZA2
CCDC152	1.5109192	0.81777537	0.88419955	NA	NA	CCDC152
CDK8	1.6055934	0.82417525	0.88419955	NA	NA	CDK8
CLCC1	-1.5448079	1.28666703	0.88419955	NA	NA	CLCC1
CLCN5	-1.6268313	1.230451	0.88419955	NA	NA	CLCN5
CLGN	-1.6539475	1.5093067	0.88419955	NA	NA	CLGN
CLYBL	1.5408082	0.78965522	0.88419955	NA	NA	CLYBL
CPNE6	-1.6220942	1.36472646	0.88419955	NA	NA	CPNE6
DDHD1	1.6422988	0.76981857	0.88419955	NA	NA	DDHD1
DLEU2L	1.4927985	0.65515587	0.88419955	NA	NA	DLEU2L
ELAVL4	-1.588411	1.23423612	0.88419955	NA	NA	ELAVL4
FABP6	-1.6316726	1.23861445	0.88419955	NA	NA	FABP6
GAS2L1	1.5550568	0.79647135	0.88419955	NA	NA	GAS2L1
GAS2L3	1.5920415	0.75218211	0.88419955	NA	NA	GAS2L3
GPD2	1.5940441	0.78962328	0.88419955	NA	NA	GPD2
GPR177	-1.5759886	1.44536871	0.88419955	NA	NA	GPR177
GUCY1A3	-1.5753948	1.37360364	0.88419955	NA	NA	GUCY1A3
IRF2	-1.5233308	1.44229607	0.88419955	NA	NA	IRF2
JUN	-1.6519549	1.47071215	0.88419955	NA	NA	JUN
KEAP1	-1.5697444	1.24489759	0.88419955	NA	NA	KEAP1

KPNA2	-1.5666428	1.24652842	0.88419955	NA	NA	KPNA2
MAN1C1	1.5184786	0.83719153	0.88419955	NA	NA	MAN1C1
MSH2	-1.5195244	1.23633967	0.88419955	NA	NA	MSH2
NBL1	-1.5567805	1.26174467	0.88419955	NA	NA	NBL1
NPHP3	1.6046245	0.80858105	0.88419955	NA	NA	NPHP3
NRD1	1.5794877	0.77648009	0.88419955	NA	NA	NRD1
NUDCD1	1.5156774	0.76342572	0.88419955	NA	NA	NUDCD1
PACSIN1	1.5554403	0.8116957	0.88419955	NA	NA	PACSIN1
PPAT	1.5750822	0.79892128	0.88419955	NA	NA	PPAT
PPFIA2	1.6049976	0.82402652	0.88419955	NA	NA	PPFIA2
PPP2R5A	1.5226625	0.79373633	0.88419955	NA	NA	PPP2R5A
PRSS12	1.5393879	0.7075545	0.88419955	NA	NA	PRSS12
PTGDS	-1.5720961	1.54986996	0.88419955	NA	NA	PTGDS
RCC2	-1.6074405	1.25214465	0.88419955	NA	NA	RCC2
RGMB	1.5259339	0.82087114	0.88419955	NA	NA	RGMB
RHOBTB1	1.5203465	0.80651117	0.88419955	NA	NA	RHOBTB1
RNF169	1.5327919	0.80108988	0.88419955	NA	NA	RNF169
RPL37A	1.5098859	0.80697164	0.88419955	NA	NA	RPL37A
RSPO3	-1.5307645	1.28286727	0.88419955	NA	NA	RSPO3
SCG2	-1.5585077	1.37500787	0.88419955	NA	NA	SCG2
SLC44A5	1.5080185	0.7401177	0.88419955	NA	NA	SLC44A5
SLC5A3	1.6113967	0.81212852	0.88419955	NA	NA	SLC5A3
SNX22	1.6470677	0.7752698	0.88419955	NA	NA	SNX22
SSBP2	1.4973895	0.83258835	0.88419955	NA	NA	SSBP2
STARD13	1.5338506	0.81861749	0.88419955	NA	NA	STARD13
STRBP	1.5625995	0.76323323	0.88419955	NA	NA	STRBP
TF	-1.5563084	1.25399026	0.88419955	NA	NA	TF
TGFB3	-1.628569	1.28476102	0.88419955	NA	NA	TGFB3
TLN2	1.6341747	0.79796596	0.88419955	NA	NA	TLN2
TNRC6B	1.5843737	0.78946261	0.88419955	NA	NA	TNRC6B
TSEN15	-1.6274658	1.29362016	0.88419955	NA	NA	TSEN15
UBE2L6	-1.6195086	1.21735044	0.88419955	NA	NA	UBE2L6
UVRAG	1.5689983	0.81164414	0.88419955	NA	NA	UVRAG
ZDHHC23	1.5498989	0.74854916	0.88419955	NA	NA	ZDHHC23
ZNF365	1.6080121	0.78220485	0.88419955	NA	NA	ZNF365
PRKCB	1.623504	0.7653644	0.88419955	NA	PRKCB	PRKCB
SH3KBP1	1.6217678	0.81439028	0.88419955	NA	SH3KBP1	SH3KBP1
SMAD7	1.5822772	0.77254442	0.88419955	NA	SMAD7	SMAD7
SPRED2	-1.5160014	1.21935135	0.88419955	NA	SPRED2	SPRED2
STAG2	1.5384991	0.80153576	0.88419955	NA	STAG2	STAG2
SYT13	1.5262234	0.77492133	0.88419955	NA	SYT13	SYT13
TDP1	-1.6031528	1.24968789	0.88419955	NA	TDP1	TDP1
TMEM47	-1.6560851	1.2938101	0.88419955	NA	TMEM47	TMEM47
TMEM5	-1.5773597	1.27996903	0.88419955	NA	TMEM5	TMEM5
ZNF395	-1.598808	1.21514445	0.88419955	NA	ZNF395	ZNF395

MAR only PFOvsPT q<0.01

SYMBOL	PFOvsPT.d	PFOvsPT.FC	PFOvsPT.q	HAR	CAR	MAR
AADAT	-2.5028615	0.7549555	0	NA	NA	AADAT
ABCA1	-3.0361926	0.63183898	0	NA	NA	ABCA1
ACTN2	5.7230026	2.15670768	0	NA	NA	ACTN2
ACVR1C	4.1708629	2.27378292	0	NA	NA	ACVR1C
ADAMTS3	-2.4132743	0.6734777	0	NA	NA	ADAMTS3

ADCY8	-7.3811571	0.27123935	0	NA	NA	ADCY8
ADI1	2.2650305	1.32784445	0	NA	NA	ADI1
AK3L1	-2.7061578	0.57984315	0	NA	NA	AK3L1
ANKH	2.6719111	1.46595885	0	NA	NA	ANKH
ANKRD57	3.0675827	1.54284388	0	NA	NA	ANKRD57
ANKS1B	5.4887345	2.15601428	0	NA	NA	ANKS1B
ANO3	2.3646069	2.08089745	0	NA	NA	ANO3
ANO4	-3.3417516	0.65564835	0	NA	NA	ANO4
ANTXR2	3.9176954	1.71321708	0	NA	NA	ANTXR2
ARHGAP6	-3.195736	0.53196867	0	NA	NA	ARHGAP6
ARHGEF6	2.8277758	1.55551638	0	NA	NA	ARHGEF6
ARL4A	2.511116	1.57865082	0	NA	NA	ARL4A
ASAH1	2.5440324	1.36247185	0	NA	NA	ASAH1
ASCL1	4.3720168	1.98767465	0	NA	NA	ASCL1
ATP8A2	-2.771165	0.66020022	0	NA	NA	ATP8A2
B3GALT2	3.1860665	1.55387412	0	NA	NA	B3GALT2
B3GAT2	-2.2564059	0.66397852	0	NA	NA	B3GAT2
B3GNT2	-2.2363781	0.75103223	0	NA	NA	B3GNT2
BAMBI	-2.5486832	0.57267348	0	NA	NA	BAMBI
BARD1	3.8839376	2.53533929	0	NA	NA	BARD1
BATF3	2.2609024	1.50106669	0	NA	NA	BATF3
BDNF	-6.0284835	0.21138993	0	NA	NA	BDNF
BEND6	2.719765	1.59734737	0	NA	NA	BEND6
BHLHE40	2.4061711	1.4804062	0	NA	NA	BHLHE40
C14orf132	4.9873905	2.02334844	0	NA	NA	C14orf132
C16orf61	-4.4408933	0.50901455	0	NA	NA	C16orf61
C18orf1	2.6272125	1.37630553	0	NA	NA	C18orf1
C1orf198	-2.5560951	0.69344131	0	NA	NA	C1orf198
C3orf59	-3.6667364	0.58192329	0	NA	NA	C3orf59
C6orf141	-2.511954	0.64683456	0	NA	NA	C6orf141
CALB1	-10.470768	0.08570738	0	NA	NA	CALB1
CAP2	2.3484789	1.34325557	0	NA	NA	CAP2
CBLN2	-14.554868	0.08883734	0	NA	NA	CBLN2
CCDC109B	3.0258479	1.84030504	0	NA	NA	CCDC109B
CDCA7	2.3297026	1.36832726	0	NA	NA	CDCA7
CDCA7L	-3.204917	0.54898463	0	NA	NA	CDCA7L
CDH3	2.8363774	1.51154625	0	NA	NA	CDH3
CENPF	-2.9824191	0.63582166	0	NA	NA	CENPF
CHRDL1	-4.5280955	0.34675859	0	NA	NA	CHRDL1
CHST8	2.9652964	2.04816076	0	NA	NA	CHST8
CLDN1	-6.6031156	0.40869989	0	NA	NA	CLDN1
CNIH3	-3.9901337	0.58182579	0	NA	NA	CNIH3
CNTNAP2	-5.1222137	0.29933509	0	NA	NA	CNTNAP2
COL12A1	-2.5600556	0.68578824	0	NA	NA	COL12A1
COMMD2	2.6738365	1.38859018	0	NA	NA	COMMD2
CRISPLD1	-2.8583041	0.60569868	0	NA	NA	CRISPLD1
CSRP2	-5.7836768	0.43376702	0	NA	NA	CSRP2
CUX1	8.0750155	2.94395919	0	NA	NA	CUX1
CXCR4	2.4724394	1.35294084	0	NA	NA	CXCR4
CXXC4	-3.9002226	0.59401831	0	NA	NA	CXXC4
CXXC5	-5.7185821	0.394487	0	NA	NA	CXXC5
CYP26A1	-2.9104637	0.48298082	0	NA	NA	CYP26A1
DERA	-3.2478245	0.52522287	0	NA	NA	DERA
DNER	2.2646872	1.47986504	0	NA	NA	DNER
DUSP10	-4.7462373	0.52847573	0	NA	NA	DUSP10
DUSP4	-4.5331275	0.42008895	0	NA	NA	DUSP4
EDIL3	2.4718718	1.39883668	0	NA	NA	EDIL3
EFR3A	-2.5473489	0.67175085	0	NA	NA	EFR3A

EMX2	-3.67376	0.42022704	0	NA	NA	EMX2
EPCAM	3.3607583	1.76213938	0	NA	NA	EPCAM
EPHA7	2.3254158	1.55426373	0	NA	NA	EPHA7
EPS15	-2.380353	0.67254989	0	NA	NA	EPS15
F3	-2.1747337	0.57731632	0	NA	NA	F3
FAM59A	-2.4934649	0.56034191	0	NA	NA	FAM59A
FERMT2	4.2632045	2.08015025	0	NA	NA	FERMT2
FGFBP3	5.9094274	2.52106105	0	NA	NA	FGFBP3
FHDC1	-6.7100988	0.39625724	0	NA	NA	FHDC1
FJX1	5.5721969	2.52410736	0	NA	NA	FJX1
FLJ11235	2.3242112	1.49174606	0	NA	NA	FLJ11235
FOXP1	2.5735375	1.39880052	0	NA	NA	FOXP1
FRZB	2.8764855	1.5006055	0	NA	NA	FRZB
FSTL5	-9.134059	0.19439547	0	NA	NA	FSTL5
FZD4	-2.5504763	0.67621129	0	NA	NA	FZD4
GAB1	2.4348477	1.37414607	0	NA	NA	GAB1
GABRG1	3.8878003	1.97031083	0	NA	NA	GABRG1
GALNT2	-4.2925877	0.51157608	0	NA	NA	GALNT2
GCG	-4.5083529	0.41817641	0	NA	NA	GCG
GHR	2.8771158	2.28682704	0	NA	NA	GHR
GLT25D2	-3.7023891	0.60109911	0	NA	NA	GLT25D2
GPHN	2.5740778	1.37594102	0	NA	NA	GPHN
GPR22	3.3248308	2.09048899	0	NA	NA	GPR22
GPR37	-2.8484793	0.60626699	0	NA	NA	GPR37
GPR88	-3.4255545	0.45694561	0	NA	NA	GPR88
GPSM2	-3.4162766	0.37118724	0	NA	NA	GPSM2
GREM1	-2.8864642	0.36575241	0	NA	NA	GREM1
GREM2	-4.9255321	0.24878345	0	NA	NA	GREM2
HCRTR2	-2.6419059	0.56667983	0	NA	NA	HCRTR2
HEY1	-3.7913367	0.67116494	0	NA	NA	HEY1
HIP1R	-2.1829824	0.77544948	0	NA	NA	HIP1R
HS3ST1	-2.2006599	0.70283541	0	NA	NA	HS3ST1
HS6ST3	-3.6617101	0.39658062	0	NA	NA	HS6ST3
HSPA12A	-4.2211371	0.48408819	0	NA	NA	HSPA12A
HTR1A	3.8667755	1.80561151	0	NA	NA	HTR1A
HUNK	-3.5936681	0.61545926	0	NA	NA	HUNK
IL7	-4.0113273	0.27551682	0	NA	NA	IL7
INTS12	-2.4877879	0.73465381	0	NA	NA	INTS12
IRS2	-3.4481015	0.66776093	0	NA	NA	IRS2
ITGA2	2.7273643	1.54145263	0	NA	NA	ITGA2
ITGA9	-2.8155154	0.67196215	0	NA	NA	ITGA9
ITGAV	2.574707	1.4746765	0	NA	NA	ITGAV
ITPR1	3.6500924	1.5630158	0	NA	NA	ITPR1
JMJD1C	2.5771635	1.47716536	0	NA	NA	JMJD1C
JPH1	-3.2960068	0.64061449	0	NA	NA	JPH1
KCNA5	-5.2899544	0.39735418	0	NA	NA	KCNA5
KCNC2	-2.9617191	0.6958676	0	NA	NA	KCNC2
KCNJ6	-5.6306064	0.38743862	0	NA	NA	KCNJ6
KCNK9	-3.5810556	0.55006032	0	NA	NA	KCNK9
KCNT2	2.3088159	1.8664929	0	NA	NA	KCNT2
KIAA0319	3.0140926	1.52666284	0	NA	NA	KIAA0319
KIAA0895	4.4775793	2.41716249	0	NA	NA	KIAA0895
KIAA1239	3.8815334	1.71595536	0	NA	NA	KIAA1239
KIAA1377	-5.0111275	0.44634499	0	NA	NA	KIAA1377
KIAA1804	3.1060298	1.6038578	0	NA	NA	KIAA1804
KIT	-3.1769796	0.6106721	0	NA	NA	KIT
KITLG	2.7996099	2.01379956	0	NA	NA	KITLG
KL	2.5219389	1.47537132	0	NA	NA	KL

KLHL32	5.6726516	4.06877152	0	NA	NA	KLHL32
KLHL8	3.0656515	1.43765531	0	NA	NA	KLHL8
KRT31	-3.8344871	0.45747667	0	NA	NA	KRT31
LDB2	-2.8931194	0.69446359	0	NA	NA	LDB2
LDLRAD3	4.0334391	1.96045483	0	NA	NA	LDLRAD3
LHFPL2	-4.0089999	0.55907913	0	NA	NA	LHFPL2
LNX2	-2.7594468	0.63893267	0	NA	NA	LNX2
LOC286002	6.6399202	2.6503824	0	NA	NA	LOC286002
LOX	4.6566289	1.91812442	0	NA	NA	LOX
LPL	2.7815804	1.40100464	0	NA	NA	LPL
LRRC3B	-4.8019253	0.50656235	0	NA	NA	LRRC3B
LRRTM2	-2.4679681	0.70045444	0	NA	NA	LRRTM2
LUZP2	2.5089308	2.16911589	0	NA	NA	LUZP2
MAL2	-2.6502091	0.56569847	0	NA	NA	MAL2
MAP3K5	-6.2555867	0.30492581	0	NA	NA	MAP3K5
MCHR2	-3.564827	0.52414553	0	NA	NA	MCHR2
MCTP1	3.6104371	1.7227763	0	NA	NA	MCTP1
ME3	-2.3898726	0.76248868	0	NA	NA	ME3
MEGF10	-7.2306862	0.30931825	0	NA	NA	MEGF10
MEGF11	-2.9538663	0.59606691	0	NA	NA	MEGF11
MET	3.6942663	1.7793356	0	NA	NA	MET
MIPEP	-6.0186078	0.44075398	0	NA	NA	MIPEP
MOCS2	2.6924114	1.4670378	0	NA	NA	MOCS2
MPP6	2.8271169	2.19406594	0	NA	NA	MPP6
MRAP2	2.3949268	1.94789621	0	NA	NA	MRAP2
MTFR1	2.241794	1.3382267	0	NA	NA	MTFR1
MYO16	-3.0542861	0.62416656	0	NA	NA	MYO16
MYO1E	2.4481187	1.47383707	0	NA	NA	MYO1E
NCOA2	2.3244361	1.5257123	0	NA	NA	NCOA2
NEBL	-3.959623	0.55103234	0	NA	NA	NEBL
NEDD9	-2.4947402	0.64373407	0	NA	NA	NEDD9
NEFL	6.1790036	3.17828345	0	NA	NA	NEFL
NEUROD1	-7.5610329	0.20351525	0	NA	NA	NEUROD1
NFE2L3	3.2905816	2.18477987	0	NA	NA	NFE2L3
NME5	-2.2837135	0.63575892	0	NA	NA	NME5
NPAS2	2.4130014	1.40564001	0	NA	NA	NPAS2
NPY	10.487701	6.55267487	0	NA	NA	NPY
NPY1R	-2.8091789	0.64447802	0	NA	NA	NPY1R
NR2F1	4.5333847	2.03877837	0	NA	NA	NR2F1
NRIP1	-3.2856362	0.5830483	0	NA	NA	NRIP1
NRN1	2.645197	1.63108886	0	NA	NA	NRN1
NTN4	-4.3353016	0.46955142	0	NA	NA	NTN4
NTNG1	2.5601591	1.55650575	0	NA	NA	NTNG1
NUP93	-3.4552735	0.64702256	0	NA	NA	NUP93
OSBPL6	2.5945569	1.54765722	0	NA	NA	OSBPL6
OTOR	3.3941304	3.10471059	0	NA	NA	OTOR
P2RY12	2.5446371	1.60869503	0	NA	NA	P2RY12
PACSIN2	-2.7839704	0.69023739	0	NA	NA	PACSIN2
PAICS	3.8589303	1.62731124	0	NA	NA	PAICS
PAK7	4.4807926	1.71932096	0	NA	NA	PAK7
PALMD	-3.1556924	0.54772368	0	NA	NA	PALMD
PAPPA2	3.3547903	1.47446083	0	NA	NA	PAPPA2
PCBD1	-2.4006729	0.69718401	0	NA	NA	PCBD1
PCOLCE2	2.5204469	1.72293333	0	NA	NA	PCOLCE2
PCSK2	11.205976	7.94290912	0	NA	NA	PCSK2
PDE8B	-2.9336571	0.5974711	0	NA	NA	PDE8B
PDZRN3	2.3995817	1.50896706	0	NA	NA	PDZRN3
PENK	-6.7368517	0.15534614	0	NA	NA	PENK

PGM1	-2.8866915	0.67661081	0	NA	NA	PGM1
PKP4	-2.4639251	0.74636914	0	NA	NA	PKP4
PLCXD3	4.2669402	2.09958696	0	NA	NA	PLCXD3
PLEKHG1	-3.4388593	0.42544483	0	NA	NA	PLEKHG1
POPDC3	-4.034346	0.44640618	0	NA	NA	POPDC3
POU6F2	-2.6805439	0.61101027	0	NA	NA	POU6F2
PPEF1	8.0271043	2.65600578	0	NA	NA	PPEF1
PROK2	3.9923299	2.47915943	0	NA	NA	PROK2
PRSS23	-8.5327816	0.28705427	0	NA	NA	PRSS23
PRUNE2	-2.1695295	0.72205202	0	NA	NA	PRUNE2
PTDSS1	3.5101653	1.53821303	0	NA	NA	PTDSS1
PTGFR	-4.2996081	0.32996061	0	NA	NA	PTGFR
PTPN13	3.4056784	1.89521768	0	NA	NA	PTPN13
PTPN2	2.2949353	1.38436645	0	NA	NA	PTPN2
PTPRT	-2.748046	0.58211594	0	NA	NA	PTPRT
PTX3	-5.0063375	0.37142285	0	NA	NA	PTX3
RASGRP1	8.1145198	5.47859839	0	NA	NA	RASGRP1
RASL11B	4.6466211	1.82601874	0	NA	NA	RASL11B
RBP1	-2.2620478	0.60211066	0	NA	NA	RBP1
RCAN1	-3.188018	0.64036306	0	NA	NA	RCAN1
RCAN2	-6.3698221	0.23271611	0	NA	NA	RCAN2
RFK	-3.0766184	0.70024631	0	NA	NA	RFK
RFTN1	-2.4595346	0.7249103	0	NA	NA	RFTN1
RGS8	-2.4556735	0.57131777	0	NA	NA	RGS8
RGS9	-2.3931161	0.6459222	0	NA	NA	RGS9
RPL23AP32	2.2281744	1.45333283	0	NA	NA	RPL23AP32
RPRML	3.4553787	2.52247291	0	NA	NA	RPRML
RRAS2	-2.2138059	0.69630089	0	NA	NA	RRAS2
RXFP1	-9.3099821	0.10886245	0	NA	NA	RXFP1
RXRG	-5.5586275	0.33343658	0	NA	NA	RXRG
RYR3	5.4519481	2.62617986	0	NA	NA	RYR3
SAMD13	-3.0569876	0.57550054	0	NA	NA	SAMD13
SAV1	4.2713176	2.00578038	0	NA	NA	SAV1
SDC2	2.240968	1.74016307	0	NA	NA	SDC2
SEMA3E	-2.3056371	0.61154545	0	NA	NA	SEMA3E
SERPINI1	3.7436343	1.64648367	0	NA	NA	SERPINI1
SEZ6L	2.383783	1.42686764	0	NA	NA	SEZ6L
SGCG	-3.5579458	0.34687456	0	NA	NA	SGCG
SGEF	3.0105396	1.93796991	0	NA	NA	SGEF
SGK1	4.5344644	1.83493955	0	NA	NA	SGK1
SH3D19	-2.1801666	0.74770978	0	NA	NA	SH3D19
SHISA2	5.6562868	2.9829021	0	NA	NA	SHISA2
SLC12A2	-2.2112538	0.65180215	0	NA	NA	SLC12A2
SLC17A7	3.8280472	1.52540202	0	NA	NA	SLC17A7
SLC35F2	-2.9960979	0.50770383	0	NA	NA	SLC35F2
SLITRK3	-4.2346195	0.48912647	0	NA	NA	SLITRK3
SLITRK4	2.2845762	1.34108423	0	NA	NA	SLITRK4
SMC4	3.2304147	1.50980659	0	NA	NA	SMC4
SNCAIP	-2.3999304	0.69224844	0	NA	NA	SNCAIP
SOCS2	-2.3828863	0.61493438	0	NA	NA	SOCS2
SOX9	12.91514	8.5897599	0	NA	NA	SOX9
SPIRE1	3.7605242	1.64272299	0	NA	NA	SPIRE1
SRI	-2.2418818	0.7681904	0	NA	NA	SRI
SSTR1	2.2225815	2.22097851	0	NA	NA	SSTR1
ST3GAL1	2.3076774	1.53822723	0	NA	NA	ST3GAL1
ST6GAL2	4.5318538	2.25234251	0	NA	NA	ST6GAL2
STARD4	3.0072284	1.57480578	0	NA	NA	STARD4
STK31	3.157706	3.3372967	0	NA	NA	STK31

SULF1	-4.571398	0.36129893	0	NA	NA	SULF1
SVIP	3.1539867	1.57254303	0	NA	NA	SVIP
SYNPR	2.2112118	1.56197503	0	NA	NA	SYNPR
TACR2	2.2351101	1.52657575	0	NA	NA	TACR2
TAL2	-2.8474592	0.50475296	0	NA	NA	TAL2
TBC1D8	3.2520082	1.77115654	0	NA	NA	TBC1D8
TCF7L2	-2.7195924	0.68081731	0	NA	NA	TCF7L2
TDRD7	2.4033651	1.40422382	0	NA	NA	TDRD7
TGFA	3.641964	1.67924509	0	NA	NA	TGFA
TGFB2	5.2878672	2.62779239	0	NA	NA	TGFB2
TGFBR1	2.4199176	1.53225379	0	NA	NA	TGFBR1
THSD7A	3.3056219	1.81536778	0	NA	NA	THSD7A
TLK1	3.19175	1.61467051	0	NA	NA	TLK1
TMEM200A	4.5987098	2.05921209	0	NA	NA	TMEM200A
TMEM35	-2.2798528	0.7120131	0	NA	NA	TMEM35
TNFRSF21	-2.2771288	0.74740008	0	NA	NA	TNFRSF21
TNKS2	2.4188874	1.48700963	0	NA	NA	TNKS2
TP53I3	-4.5234967	0.42963993	0	NA	NA	TP53I3
TRPC4	5.6127943	2.55992429	0	NA	NA	TRPC4
TRPC7	3.4270364	2.26915688	0	NA	NA	TRPC7
TSHZ1	-2.7662182	0.65613151	0	NA	NA	TSHZ1
TSHZ3	-4.8185133	0.41207351	0	NA	NA	TSHZ3
UFM1	2.5808817	1.48981756	0	NA	NA	UFM1
UNC13C	5.2100561	2.64098482	0	NA	NA	UNC13C
USP46	2.7529312	1.43437528	0	NA	NA	USP46
UST	-2.3511955	0.61260421	0	NA	NA	UST
WIF1	2.9832351	2.95760793	0	NA	NA	WIF1
WNT7B	3.4683119	2.04962309	0	NA	NA	WNT7B
XK	2.2480153	1.58366096	0	NA	NA	XK
ZNF114	-3.8851986	0.35458115	0	NA	NA	ZNF114
ZNF385B	3.1447634	2.1517861	0	NA	NA	ZNF385B
ZNF503	2.7163132	1.49572486	0	NA	NA	ZNF503
ZNF618	2.4840875	1.36965841	0	NA	NA	ZNF618
ABHD6	2.0503227	1.3143208	0.11329523	NA	NA	ABHD6
ADIPOR2	2.1669984	1.32217825	0.11329523	NA	NA	ADIPOR2
ADSSL1	-2.024042	0.76300326	0.11329523	NA	NA	ADSSL1
AGFG1	2.1688378	1.30533858	0.11329523	NA	NA	AGFG1
AJAP1	-2.0384512	0.76004253	0.11329523	NA	NA	AJAP1
AKR1C1	-2.1625288	0.73760832	0.11329523	NA	NA	AKR1C1
ANKRD29	-2.0779442	0.65258075	0.11329523	NA	NA	ANKRD29
AP1S2	2.1771745	1.27897977	0.11329523	NA	NA	AP1S2
BVES	-2.0094566	0.6939667	0.11329523	NA	NA	BVES
C13orf36	-2.0945015	0.46913297	0.11329523	NA	NA	C13orf36
C1orf173	2.1605387	1.53378968	0.11329523	NA	NA	C1orf173
C3orf50	-2.036135	0.50365005	0.11329523	NA	NA	C3orf50
C7orf36	-2.157551	0.67309729	0.11329523	NA	NA	C7orf36
CBARA1	-2.0722511	0.7331099	0.11329523	NA	NA	CBARA1
DRD1	-2.1159006	0.54116584	0.11329523	NA	NA	DRD1
EBPL	-2.0174033	0.76167817	0.11329523	NA	NA	EBPL
EML6	2.0822228	1.40018359	0.11329523	NA	NA	EML6
EMX1	2.0036574	1.36807504	0.11329523	NA	NA	EMX1
FRY	2.1460488	1.35090718	0.11329523	NA	NA	FRY
GGCT	-2.0093063	0.78267989	0.11329523	NA	NA	GGCT
GRIK1	2.0950291	1.462092	0.11329523	NA	NA	GRIK1
GRM5	-2.0873797	0.75637345	0.11329523	NA	NA	GRM5
HAS2	2.0574799	1.4638155	0.11329523	NA	NA	HAS2
LOC387763	2.0187893	1.30773039	0.11329523	NA	NA	LOC387763
LRRC7	2.1384318	1.36840288	0.11329523	NA	NA	LRRC7

MYF6	-2.0699629	0.73203216	0.11329523	NA	NA	MYF6
NECAB1	2.0109467	1.4618177	0.11329523	NA	NA	NECAB1
NEDD4	2.1359013	1.3669302	0.11329523	NA	NA	NEDD4
NUAK2	2.0193351	1.37421848	0.11329523	NA	NA	NUAK2
OMG	2.0489617	1.38463372	0.11329523	NA	NA	OMG
PDGFD	2.180256	1.48679372	0.11329523	NA	NA	PDGFD
PDZD2	2.1692008	1.43696806	0.11329523	NA	NA	PDZD2
PGBD5	-2.0289311	0.76288849	0.11329523	NA	NA	PGBD5
PTPRZ1	2.1107313	1.32220457	0.11329523	NA	NA	PTPRZ1
RAB11A	-2.0923258	0.80125869	0.11329523	NA	NA	RAB11A
REM2	-2.1261275	0.63751528	0.11329523	NA	NA	REM2
RFC3	2.091419	1.39729113	0.11329523	NA	NA	RFC3
RSPH9	2.0134479	1.3430666	0.11329523	NA	NA	RSPH9
SERPINB6	-2.0431848	0.77746708	0.11329523	NA	NA	SERPINB6
SERTAD2	2.1096883	1.35084759	0.11329523	NA	NA	SERTAD2
SFRS2B	-2.1154615	0.78632052	0.11329523	NA	NA	SFRS2B
SLC17A5	-2.1683251	0.73871978	0.11329523	NA	NA	SLC17A5
SLC26A8	2.0197541	1.49496114	0.11329523	NA	NA	SLC26A8
SLC27A2	2.1396481	1.27583389	0.11329523	NA	NA	SLC27A2
SMPDL3A	2.1051156	1.40829005	0.11329523	NA	NA	SMPDL3A
SNHG8	2.029819	1.30293998	0.11329523	NA	NA	SNHG8
SV2B	-2.1380977	0.7117055	0.11329523	NA	NA	SV2B
TAF4B	2.163867	1.36026583	0.11329523	NA	NA	TAF4B
TAPT1	-2.0881596	0.80118264	0.11329523	NA	NA	TAPT1
ADC	1.882217	1.34130076	0.19206239	NA	NA	ADC
ADCY1	1.8937999	1.26956376	0.19206239	NA	NA	ADCY1
AKAP7	-1.9212017	0.75107398	0.19206239	NA	NA	AKAP7
AKR1B1	-1.8873825	0.78945709	0.19206239	NA	NA	AKR1B1
ALDH7A1	-1.9677894	0.63861785	0.19206239	NA	NA	ALDH7A1
ANK2	1.9805793	1.45101776	0.19206239	NA	NA	ANK2
ANKRD6	-1.9501876	0.68859497	0.19206239	NA	NA	ANKRD6
APBB2	-1.9369471	0.74748512	0.19206239	NA	NA	APBB2
ARHGAP12	1.9922063	1.54238912	0.19206239	NA	NA	ARHGAP12
ARHGAP18	1.968179	1.41872716	0.19206239	NA	NA	ARHGAP18
ATP2C1	1.8198191	1.30337579	0.19206239	NA	NA	ATP2C1
BAI2	1.9038778	1.45636169	0.19206239	NA	NA	BAI2
C10orf58	-1.9142667	0.800382	0.19206239	NA	NA	C10orf58
C11orf75	-1.9505339	0.59200686	0.19206239	NA	NA	C11orf75
C14orf37	1.9223885	1.49479032	0.19206239	NA	NA	C14orf37
C2orf3	1.8417981	1.32300059	0.19206239	NA	NA	C2orf3
C2orf7	-1.8899315	0.75934753	0.19206239	NA	NA	C2orf7
CAMKV	1.8540275	1.2937993	0.19206239	NA	NA	CAMKV
CAV1	-1.8666365	0.68165816	0.19206239	NA	NA	CAV1
CEP135	1.8777125	1.29939271	0.19206239	NA	NA	CEP135
DEK	1.8585399	1.22513532	0.19206239	NA	NA	DEK
DENND4C	1.8540265	1.25373365	0.19206239	NA	NA	DENND4C
DYNC2H1	1.9865679	1.39179126	0.19206239	NA	NA	DYNC2H1
EIF3D	1.8358103	1.22026625	0.19206239	NA	NA	EIF3D
EPB41L3	1.819643	1.38457301	0.19206239	NA	NA	EPB41L3
FABP7	1.9955677	1.44113651	0.19206239	NA	NA	FABP7
FAM43A	-1.8784603	0.77986492	0.19206239	NA	NA	FAM43A
FRMD4B	-1.9884055	0.76981719	0.19206239	NA	NA	FRMD4B
GCA	1.9918127	1.44043152	0.19206239	NA	NA	GCA
GHRH	-1.8694463	0.75513713	0.19206239	NA	NA	GHRH
HCN1	1.8768348	1.31657746	0.19206239	NA	NA	HCN1
IGSF21	1.8198256	1.30822039	0.19206239	NA	NA	IGSF21
IMPDH2	1.8557926	1.23811241	0.19206239	NA	NA	IMPDH2
KCNAB1	-1.9093715	0.71621188	0.19206239	NA	NA	KCNAB1

KCNE1	-1.9868154	0.72364018	0.19206239	NA	NA	KCNE1
LOC100129550	1.8909605	1.3361098	0.19206239	NA	NA	LOC100129550
LOC100134229	-1.9667124	0.6992505	0.19206239	NA	NA	LOC100134229
LOC151162	-1.8938993	0.78972489	0.19206239	NA	NA	LOC151162
LRRC8C	-1.9658767	0.77919815	0.19206239	NA	NA	LRRC8C
MYB	-1.9590426	0.74880495	0.19206239	NA	NA	MYB
MYC	-1.8492218	0.79308378	0.19206239	NA	NA	MYC
N4BP2L2	1.8287236	1.30698477	0.19206239	NA	NA	N4BP2L2
NAP1L5	-1.8801924	0.77508106	0.19206239	NA	NA	NAP1L5
NPNT	-1.8799266	0.69522007	0.19206239	NA	NA	NPNT
PAG1	1.9923476	1.327042	0.19206239	NA	NA	PAG1
PHF17	1.9406617	1.36023855	0.19206239	NA	NA	PHF17
PITPNC1	-1.9241893	0.78105615	0.19206239	NA	NA	PITPNC1
PLCXD2	-1.8698634	0.79351593	0.19206239	NA	NA	PLCXD2
RAB2B	-1.8679581	0.81302264	0.19206239	NA	NA	RAB2B
RHPN2	1.9213888	1.36084867	0.19206239	NA	NA	RHPN2
RIN2	1.9573726	1.31517701	0.19206239	NA	NA	RIN2
RP1-177G6.2	1.9769926	1.77329184	0.19206239	NA	NA	RP1-177G6.2
RPH3A	-1.8374374	0.57130312	0.19206239	NA	NA	RPH3A
RPL37	1.8538167	1.24920853	0.19206239	NA	NA	RPL37
SCNN1G	-1.9659273	0.70637281	0.19206239	NA	NA	SCNN1G
SLC46A3	-1.8978447	0.77893869	0.19206239	NA	NA	SLC46A3
SNAI2	-1.8931277	0.69935496	0.19206239	NA	NA	SNAI2
SUPT3H	-1.8828569	0.70905217	0.19206239	NA	NA	SUPT3H
SYDE2	-1.9700165	0.71373403	0.19206239	NA	NA	SYDE2
TIAM2	1.9183267	1.37538321	0.19206239	NA	NA	TIAM2
TMEM74	1.9308704	1.37957754	0.19206239	NA	NA	TMEM74
TMTC1	1.9325874	1.66412935	0.19206239	NA	NA	TMTC1
TRIM44	1.9809887	1.31025402	0.19206239	NA	NA	TRIM44
TSPAN18	-1.941617	0.81257968	0.19206239	NA	NA	TSPAN18
TSPAN6	-1.9717261	0.64641447	0.19206239	NA	NA	TSPAN6
WNT3	-1.8373978	0.78036455	0.19206239	NA	NA	WNT3
WNT5B	1.9573525	1.271378	0.19206239	NA	NA	WNT5B
ZNF438	1.9131199	1.26512126	0.19206239	NA	NA	ZNF438
ABI2	1.8002732	1.26758833	0.56921718	NA	NA	ABI2
ADD2	1.7072329	1.25340681	0.56921718	NA	NA	ADD2
AHR	1.7311067	1.3417973	0.56921718	NA	NA	AHR
ALS2	1.730133	1.28535736	0.56921718	NA	NA	ALS2
AMMECR1	1.7897814	1.28324269	0.56921718	NA	NA	AMMECR1
ARHGEF3	-1.6834136	0.81636501	0.56921718	NA	NA	ARHGEF3
ARL4C	-1.7089553	0.82417446	0.56921718	NA	NA	ARL4C
ATP2B1	1.7101363	1.2716682	0.56921718	NA	NA	ATP2B1
B3GALTL	-1.7134436	0.64627247	0.56921718	NA	NA	B3GALTL
BCAP29	1.77772	1.25756116	0.56921718	NA	NA	BCAP29
BCAR3	1.6554876	1.28031272	0.56921718	NA	NA	BCAR3
C17orf58	-1.664373	0.79888166	0.56921718	NA	NA	C17orf58
CABP1	1.7476754	1.38483284	0.56921718	NA	NA	CABP1
CHD3	1.7570376	1.33626467	0.56921718	NA	NA	CHD3
CHRNB1	-1.6878377	0.76774502	0.56921718	NA	NA	CHRNB1
CSMD2	1.6504898	1.27792483	0.56921718	NA	NA	CSMD2
CTGF	-1.7315289	0.66602563	0.56921718	NA	NA	CTGF
DGKH	1.6708511	1.22299786	0.56921718	NA	NA	DGKH
DNAJC12	-1.779901	0.75001516	0.56921718	NA	NA	DNAJC12
E2F3	-1.7467634	0.78189698	0.56921718	NA	NA	E2F3
ECHDC3	1.7663812	1.42340193	0.56921718	NA	NA	ECHDC3
FGFR1	-1.7778665	0.82296865	0.56921718	NA	NA	FGFR1

FNDC3B	1.7206151	1.22733849	0.56921718	NA	NA	FNDC3B
FTL	-1.6927348	0.80877996	0.56921718	NA	NA	FTL
GNAL	-1.6730574	0.54693306	0.56921718	NA	NA	GNAL
GPR137C	-1.7683416	0.68738416	0.56921718	NA	NA	GPR137C
GPR63	1.7023713	1.47988423	0.56921718	NA	NA	GPR63
HEATR1	1.7673478	1.49183125	0.56921718	NA	NA	HEATR1
HSPA4L	1.7627049	1.44092352	0.56921718	NA	NA	HSPA4L
HYMAI	1.7725019	1.50141984	0.56921718	NA	NA	HYMAI
IPO8	1.7151015	1.27950032	0.56921718	NA	NA	IPO8
JAK2	1.766462	1.27639377	0.56921718	NA	NA	JAK2
KCNK2	-1.6590451	0.75995088	0.56921718	NA	NA	KCNK2
KLHL25	1.6601986	1.31920026	0.56921718	NA	NA	KLHL25
LOC401097	1.7677791	1.4304492	0.56921718	NA	NA	LOC401097
LYSMD3	1.6959089	1.33586999	0.56921718	NA	NA	LYSMD3
MAN2A1	1.7357733	1.45311751	0.56921718	NA	NA	MAN2A1
NALCN	-1.7134353	0.74687867	0.56921718	NA	NA	NALCN
NEFM	1.8104381	1.30577194	0.56921718	NA	NA	NEFM
OAT	-1.665461	0.80796965	0.56921718	NA	NA	OAT
PAQR8	-1.6793243	0.73816798	0.56921718	NA	NA	PAQR8
PCOTH	-1.7710008	0.78754631	0.56921718	NA	NA	PCOTH
PPA2	-1.716176	0.77103613	0.56921718	NA	NA	PPA2
PPP1R3C	-1.7865883	0.78211499	0.56921718	NA	NA	PPP1R3C
PRDM10	1.7214244	1.24302379	0.56921718	NA	NA	PRDM10
QPCT	-1.6989685	0.65673766	0.56921718	NA	NA	QPCT
RBM24	1.7236535	1.29322092	0.56921718	NA	NA	RBM24
RGS16	-1.6671199	0.74993059	0.56921718	NA	NA	RGS16
SAMD3	1.8075569	1.28212975	0.56921718	NA	NA	SAMD3
SCRN1	1.7554073	1.29113944	0.56921718	NA	NA	SCRN1
SEMA3C	-1.6712737	0.73090928	0.56921718	NA	NA	SEMA3C
SHB	1.7365438	1.23245074	0.56921718	NA	NA	SHB
SIPA1L2	1.7967593	1.22356026	0.56921718	NA	NA	SIPA1L2
SLC17A8	-1.6870861	0.65997115	0.56921718	NA	NA	SLC17A8
SPATA13	-1.6937885	0.75737413	0.56921718	NA	NA	SPATA13
SYT9	-1.7174943	0.80107756	0.56921718	NA	NA	SYT9
TBC1D1	-1.7283841	0.81527098	0.56921718	NA	NA	TBC1D1
TBR1	-1.7852788	0.78030639	0.56921718	NA	NA	TBR1
TNFAIP3	-1.7459981	0.57919295	0.56921718	NA	NA	TNFAIP3
UBP1	1.7354988	1.310291	0.56921718	NA	NA	UBP1
VEPH1	-1.6686711	0.82448527	0.56921718	NA	NA	VEPH1
WWP2	-1.6644297	0.7917985	0.56921718	NA	NA	WWP2
AMIGO2	1.5389169	1.2650742	0.88419955	NA	NA	AMIGO2
ASPH	1.6025921	1.22720047	0.88419955	NA	NA	ASPH
BCL2	1.6262094	1.37936056	0.88419955	NA	NA	BCL2
BRAF	1.6067269	1.29224398	0.88419955	NA	NA	BRAF
C11orf87	-1.5472354	0.70572288	0.88419955	NA	NA	C11orf87
C1GALT1	-1.5028948	0.78701074	0.88419955	NA	NA	C1GALT1
C6orf138	-1.6177315	0.72929489	0.88419955	NA	NA	C6orf138
CAPZA2	1.4915659	1.20615683	0.88419955	NA	NA	CAPZA2
CCDC152	1.5109192	1.22282968	0.88419955	NA	NA	CCDC152
CDK8	1.6055934	1.21333418	0.88419955	NA	NA	CDK8
CLCC1	-1.5448079	0.77720185	0.88419955	NA	NA	CLCC1
CLCN5	-1.6268313	0.81271014	0.88419955	NA	NA	CLCN5
CLGN	-1.6539475	0.66255586	0.88419955	NA	NA	CLGN
CLYBL	1.5408082	1.26637546	0.88419955	NA	NA	CLYBL
CPNE6	-1.6220942	0.73274757	0.88419955	NA	NA	CPNE6
DDHD1	1.6422988	1.29900738	0.88419955	NA	NA	DDHD1
DLEU2L	1.4927985	1.52635434	0.88419955	NA	NA	DLEU2L
ELAVL4	-1.588411	0.81021774	0.88419955	NA	NA	ELAVL4

FABP6	-1.6316726	0.80735373	0.88419955	NA	NA	FABP6
GAS2L1	1.5550568	1.25553795	0.88419955	NA	NA	GAS2L1
GAS2L3	1.5920415	1.32946527	0.88419955	NA	NA	GAS2L3
GPD2	1.5940441	1.26642669	0.88419955	NA	NA	GPD2
GPR177	-1.5759886	0.69186499	0.88419955	NA	NA	GPR177
GUCY1A3	-1.5753948	0.72801205	0.88419955	NA	NA	GUCY1A3
IRF2	-1.5233308	0.69333892	0.88419955	NA	NA	IRF2
JUN	-1.6519549	0.67994271	0.88419955	NA	NA	JUN
KEAP1	-1.5697444	0.80327893	0.88419955	NA	NA	KEAP1
KPNA2	-1.5666428	0.802228	0.88419955	NA	NA	KPNA2
MAN1C1	1.5184786	1.1944698	0.88419955	NA	NA	MAN1C1
MSH2	-1.5195244	0.80883921	0.88419955	NA	NA	MSH2
NBL1	-1.5567805	0.79255338	0.88419955	NA	NA	NBL1
NPHP3	1.6046245	1.2367344	0.88419955	NA	NA	NPHP3
NRD1	1.5794877	1.28786303	0.88419955	NA	NA	NRD1
NUDCD1	1.5156774	1.30988513	0.88419955	NA	NA	NUDCD1
PACSIN1	1.5554403	1.23198878	0.88419955	NA	NA	PACSIN1
PPAT	1.5750822	1.25168778	0.88419955	NA	NA	PPAT
PPFIA2	1.6049976	1.21355317	0.88419955	NA	NA	PPFIA2
PPP2R5A	1.5226625	1.25986421	0.88419955	NA	NA	PPP2R5A
PRSS12	1.5393879	1.41331869	0.88419955	NA	NA	PRSS12
PTGDS	-1.5720961	0.64521542	0.88419955	NA	NA	PTGDS
RCC2	-1.6074405	0.79862977	0.88419955	NA	NA	RCC2
RGMB	1.5259339	1.218218	0.88419955	NA	NA	RGMB
RHOBTB1	1.5203465	1.23990843	0.88419955	NA	NA	RHOBTB1
RNF169	1.5327919	1.24829937	0.88419955	NA	NA	RNF169
RPL37A	1.5098859	1.23920092	0.88419955	NA	NA	RPL37A
RSPO3	-1.5307645	0.77950387	0.88419955	NA	NA	RSPO3
SCG2	-1.5585077	0.72726856	0.88419955	NA	NA	SCG2
SLC44A5	1.5080185	1.35113644	0.88419955	NA	NA	SLC44A5
SLC5A3	1.6113967	1.23133221	0.88419955	NA	NA	SLC5A3
SNX22	1.6470677	1.28987355	0.88419955	NA	NA	SNX22
SSBP2	1.4973895	1.20107373	0.88419955	NA	NA	SSBP2
STARD13	1.5338506	1.22157176	0.88419955	NA	NA	STARD13
STRBP	1.5625995	1.31021548	0.88419955	NA	NA	STRBP
TF	-1.5563084	0.79745436	0.88419955	NA	NA	TF
TGFB3	-1.628569	0.77835487	0.88419955	NA	NA	TGFB3
TLN2	1.6341747	1.25318628	0.88419955	NA	NA	TLN2
TNRC6B	1.5843737	1.26668444	0.88419955	NA	NA	TNRC6B
TSEN15	-1.6274658	0.77302444	0.88419955	NA	NA	TSEN15
UBE2L6	-1.6195086	0.82145615	0.88419955	NA	NA	UBE2L6
UVRAG	1.5689983	1.23206705	0.88419955	NA	NA	UVRAG
ZDHHC23	1.5498989	1.3359176	0.88419955	NA	NA	ZDHHC23
ZNF365	1.6080121	1.27843748	0.88419955	NA	NA	ZNF365

HAR 17vs19 q<0.01

SYMBOL	17vs19.d	17vs19.FC	17vs19.q	HAR	CAR	MAR
ALG10	-4.3960045	1.61044113	0	ALG10	ALG10	ALG10
CYorf15A	4.8826982	0.33984058	0	CYorf15A	CYorf15A	NA
DAZ3	3.1560883	0.66021176	0	DAZ3	DAZ3	NA
FAM27A	3.7260472	0.66240645	0	FAM27A	FAM27A	FAM27A
FRG1B	4.9790263	0.53342964	0	FRG1B	FRG1B	NA
GPC6	-2.9574624	1.70632811	0	GPC6	GPC6	GPC6
KGFLP1	-6.0498232	1.92952432	0	KGFLP1	KGFLP1	KGFLP1

LOC645166	2.9873503	0.70416815	0	LOC645166	LOC645166	NA
LOC654342	4.2238118	0.61249671	0	LOC654342	LOC654342	NA
MPDZ	-3.8035766	1.38811298	0	MPDZ	MPDZ	MPDZ
MYOM2	3.9185099	0.60750334	0	MYOM2	MYOM2	MYOM2
AFAP1L2	-3.1976804	1.61900834	0	AFAP1L2	NA	AFAP1L2
CDR2	-4.3926751	1.59802619	0	CDR2	NA	CDR2
DAD1	-3.4090834	1.42475318	0	DAD1	NA	DAD1
DKK2	-2.7972101	1.54347974	0	DKK2	NA	DKK2
FAM19A2	3.0791036	0.75560504	0	FAM19A2	NA	FAM19A2
FOXC1	-3.4526707	1.89779589	0	FOXC1	NA	FOXC1
GSTT2	4.7937351	0.46999547	0	GSTT2	NA	NA
KLF5	-3.3245929	1.31204257	0	KLF5	NA	KLF5
LOC654433	6.1800862	0.38815097	0	LOC654433	NA	LOC654433
LOC80154	4.1659068	0.69723599	0	LOC80154	NA	NA
NANOS1	3.0195092	0.76802038	0	NANOS1	NA	NANOS1
NIPSNAP3A	6.4560253	0.48119602	0	NIPSNAP3A	NA	NA
NLGN4Y	11.059543	0.23828453	0	NLGN4Y	NA	NA
PITPNM3	3.1659088	0.71006956	0	PITPNM3	NA	PITPNM3
SRR	-4.3326417	1.52243837	0	SRR	NA	SRR
SSR1	-3.1579583	1.33434048	0	SSR1	NA	SSR1
STAG3L4	-5.9270751	1.80174807	0	STAG3L4	NA	STAG3L4
TBC1D15	-2.7400071	1.3902345	0	TBC1D15	NA	TBC1D15
VIP	3.1452416	0.72815259	0	VIP	NA	VIP
ZFR	-2.6851191	1.38190809	0	ZFR	NA	NA
ZNF385D	-2.6964259	1.68972635	0	ZNF385D	NA	NA
SDPR	-3.040839	1.4477601	0	SDPR	SDPR	SDPR
TTTY15	16.499994	0.04287293	0	TTTY15	TTTY15	TTTY15
C7orf28B	-2.5054821	1.33288865	0.15408684	C7orf28B	C7orf28B	NA
DAZ1	2.8255536	0.75360375	0.15408684	DAZ1	DAZ1	NA
EDNRA	-2.5143927	1.50640502	0.15408684	EDNRA	EDNRA	EDNRA
ITIH5	-2.3664665	1.25547418	0.15408684	ITIH5	ITIH5	ITIH5
MAP3K7	-2.3125599	1.23669912	0.15408684	MAP3K7	MAP3K7	MAP3K7
APCDD1	-2.375738	1.25099685	0.15408684	APCDD1	NA	APCDD1
BPGM	-2.4073412	1.32814895	0.15408684	BPGM	NA	BPGM
C9orf72	-2.3188655	1.25802647	0.15408684	C9orf72	NA	C9orf72
DAZ4	2.859064	0.57365678	0.15408684	DAZ4	NA	DAZ4
LOC400657	-2.6144841	1.26930781	0.15408684	LOC400657	NA	LOC400657
PRKAB2	-2.4229614	1.32794586	0.15408684	PRKAB2	NA	PRKAB2
PZP	2.7303672	0.75805215	0.15408684	PZP	NA	NA
TMSB4Y	2.7244112	0.74036259	0.15408684	TMSB4Y	NA	NA
TOP1	-2.5863571	1.42133876	0.15408684	TOP1	NA	TOP1
NFU1	2.5576059	0.78910103	0.15408684	NFU1	NFU1	NFU1
NKAIN3	2.9072777	0.80136794	0.15408684	NKAIN3	NKAIN3	NKAIN3
PKN2	-2.5086486	1.30100005	0.15408684	PKN2	PKN2	PKN2
PSAT1	-2.3191814	1.22755029	0.15408684	PSAT1	PSAT1	PSAT1
TARSL2	2.4999564	0.80142314	0.15408684	TARSL2	TARSL2	NA
WSB1	-2.3133712	1.38965777	0.15408684	WSB1	WSB1	WSB1
ATOH1	2.1058924	0.72609592	0.52040198	ATOH1	ATOH1	ATOH1
EFCAB4B	2.4987579	0.8501656	0.52040198	EFCAB4B	EFCAB4B	EFCAB4B
FAM96A	-1.9027157	1.18994558	0.52040198	FAM96A	FAM96A	FAM96A
FAT1	-2.1651627	1.28255083	0.52040198	FAT1	FAT1	FAT1
FRMD6	-2.2175814	1.30253868	0.52040198	FRMD6	FRMD6	FRMD6
KCTD12	-1.9045931	1.28329693	0.52040198	KCTD12	KCTD12	KCTD12
KDR	-2.1881049	1.32116515	0.52040198	KDR	KDR	KDR
LOC100132832	2.3888489	0.7146981	0.52040198	LOC1001328	LOC100132832	NA
LOC400891	-1.9715033	1.18672323	0.52040198	LOC400891	LOC400891	NA
ADPGK	-2.0753836	1.21126407	0.52040198	ADPGK	NA	ADPGK
ALDH6A1	-1.9990247	1.22934956	0.52040198	ALDH6A1	NA	ALDH6A1

CA9	2.0772529	0.71449784	0.52040198	CA9	NA	CA9
DULLARD	2.3121778	0.81341173	0.52040198	DULLARD	NA	NA
FCGR3B	-2.1865778	1.17965061	0.52040198	FCGR3B	NA	NA
FLJ46321	2.44914	0.76711204	0.52040198	FLJ46321	NA	FLJ46321
GH1	2.1085009	0.82681608	0.52040198	GH1	NA	NA
HLA-DRB1	2.1210444	0.68540221	0.52040198	HLA-DRB1	NA	NA
IFLTD1	2.4490002	0.75215324	0.52040198	IFLTD1	NA	IFLTD1
LOC541471	-2.1699253	1.20290734	0.52040198	LOC541471	NA	LOC541471
MGST2	-2.1789181	1.41626241	0.52040198	MGST2	NA	MGST2
NEK8	2.0962729	0.78434452	0.52040198	NEK8	NA	NA
ORC2L	-1.9637817	1.46302166	0.52040198	ORC2L	NA	ORC2L
PARP4	-2.0216843	1.56362627	0.52040198	PARP4	NA	NA
PAX8	2.1322173	0.81230924	0.52040198	PAX8	NA	PAX8
PCDHA3	2.3164964	0.74777155	0.52040198	PCDHA3	NA	NA
SPA17	2.2711195	0.77403425	0.52040198	SPA17	NA	NA
TCEA3	2.0861038	0.79924679	0.52040198	TCEA3	NA	TCEA3
TMTC2	-2.1768285	1.45902545	0.52040198	TMTC2	NA	TMTC2
TUBG2	2.2009254	0.7412214	0.52040198	TUBG2	NA	NA
TXNL1	-1.9519404	1.29571028	0.52040198	TXNL1	NA	TXNL1
ZBBX	2.4536853	0.74870382	0.52040198	ZBBX	NA	ZBBX
NHLH2	-1.9479143	1.31275162	0.52040198	NHLH2	NHLH2	NA
PLXNA2	-1.9743795	1.4904532	0.52040198	PLXNA2	PLXNA2	PLXNA2
TLE4	-2.2209082	1.42075177	0.52040198	TLE4	TLE4	TLE4
ZNF200	-2.1070827	1.20967709	0.52040198	ZNF200	ZNF200	NA

HAR-CAR 17vs19 q<0.01

SYMBOL	17vs19.d	17vs19.FC	17vs19.q	HAR	CAR	MAR
AFAP1L2	-3.1976804	1.61900834	0	AFAP1L2	NA	AFAP1L2
CDR2	-4.3926751	1.59802619	0	CDR2	NA	CDR2
DAD1	-3.4090834	1.42475318	0	DAD1	NA	DAD1
DKK2	-2.7972101	1.54347974	0	DKK2	NA	DKK2
FAM19A2	3.0791036	0.75560504	0	FAM19A2	NA	FAM19A2
FOXC1	-3.4526707	1.89779589	0	FOXC1	NA	FOXC1
KLF5	-3.3245929	1.31204257	0	KLF5	NA	KLF5
LOC654433	6.1800862	0.38815097	0	LOC654433	NA	LOC654433
GSTT2	4.7937351	0.46999547	0	GSTT2	NA	NA
LOC80154	4.1659068	0.69723599	0	LOC80154	NA	NA
NIPSNAP3A	6.4560253	0.48119602	0	NIPSNAP3A	NA	NA
NLGN4Y	11.059543	0.23828453	0	NLGN4Y	NA	NA
ZFR	-2.6851191	1.38190809	0	ZFR	NA	NA
ZNF385D	-2.6964259	1.68972635	0	ZNF385D	NA	NA
NANOS1	3.0195092	0.76802038	0	NANOS1	NA	NANOS1
PITPNM3	3.1659088	0.71006956	0	PITPNM3	NA	PITPNM3
SRR	-4.3326417	1.52243837	0	SRR	NA	SRR
SSR1	-3.1579583	1.33434048	0	SSR1	NA	SSR1
STAG3L4	-5.9270751	1.80174807	0	STAG3L4	NA	STAG3L4
TBC1D15	-2.7400071	1.3902345	0	TBC1D15	NA	TBC1D15
VIP	3.1452416	0.72815259	0	VIP	NA	VIP
APCDD1	-2.375738	1.25099685	0.15408684	APCDD1	NA	APCDD1
BPGM	-2.4073412	1.32814895	0.15408684	BPGM	NA	BPGM
C9orf72	-2.3188655	1.25802647	0.15408684	C9orf72	NA	C9orf72
DAZ4	2.859064	0.57365678	0.15408684	DAZ4	NA	DAZ4
LOC400657	-2.6144841	1.26930781	0.15408684	LOC400657	NA	LOC400657
PZP	2.7303672	0.75805215	0.15408684	PZP	NA	NA

TMSB4Y	2.7244112	0.74036259	0.15408684	TMSB4Y	NA	NA
PRKAB2	-2.4229614	1.32794586	0.15408684	PRKAB2	NA	PRKAB2
TOP1	-2.5863571	1.42133876	0.15408684	TOP1	NA	TOP1
ADPGK	-2.0753836	1.21126407	0.52040198	ADPGK	NA	ADPGK
ALDH6A1	-1.9990247	1.22934956	0.52040198	ALDH6A1	NA	ALDH6A1
CA9	2.0772529	0.71449784	0.52040198	CA9	NA	CA9
FLJ46321	2.44914	0.76711204	0.52040198	FLJ46321	NA	FLJ46321
IFLTD1	2.4490002	0.75215324	0.52040198	IFLTD1	NA	IFLTD1
LOC541471	-2.1699253	1.20290734	0.52040198	LOC541471	NA	LOC541471
MGST2	-2.1789181	1.41626241	0.52040198	MGST2	NA	MGST2
DULLARD	2.3121778	0.81341173	0.52040198	DULLARD	NA	NA
FCGR3B	-2.1865778	1.17965061	0.52040198	FCGR3B	NA	NA
GH1	2.1085009	0.82681608	0.52040198	GH1	NA	NA
HLA-DRB1	2.1210444	0.68540221	0.52040198	HLA-DRB1	NA	NA
NEK8	2.0962729	0.78434452	0.52040198	NEK8	NA	NA
PARP4	-2.0216843	1.56362627	0.52040198	PARP4	NA	NA
PCDHA3	2.3164964	0.74777155	0.52040198	PCDHA3	NA	NA
SPA17	2.2711195	0.77403425	0.52040198	SPA17	NA	NA
TUBG2	2.2009254	0.7412214	0.52040198	TUBG2	NA	NA
ORC2L	-1.9637817	1.46302166	0.52040198	ORC2L	NA	ORC2L
PAX8	2.1322173	0.81230924	0.52040198	PAX8	NA	PAX8
TCEA3	2.0861038	0.79924679	0.52040198	TCEA3	NA	TCEA3
TMTC2	-2.1768285	1.45902545	0.52040198	TMTC2	NA	TMTC2
TXNL1	-1.9519404	1.29571028	0.52040198	TXNL1	NA	TXNL1
ZBBX	2.4536853	0.74870382	0.52040198	ZBBX	NA	ZBBX

HAR only 17vs19 q<0.01

SYMBOL	17vs19.d	17vs19.FC	17vs19.q	HAR	CAR	MAR
GSTT2	4.7937351	0.46999547	0	GSTT2	NA	NA
LOC80154	4.1659068	0.69723599	0	LOC80154	NA	NA
NIPSNAP3A	6.4560253	0.48119602	0	NIPSNAP3A	NA	NA
NLGN4Y	11.059543	0.23828453	0	NLGN4Y	NA	NA
ZFR	-2.6851191	1.38190809	0	ZFR	NA	NA
ZNF385D	-2.6964259	1.68972635	0	ZNF385D	NA	NA
PZP	2.7303672	0.75805215	0.15408684	PZP	NA	NA
TMSB4Y	2.7244112	0.74036259	0.15408684	TMSB4Y	NA	NA
DULLARD	2.3121778	0.81341173	0.52040198	DULLARD	NA	NA
FCGR3B	-2.1865778	1.17965061	0.52040198	FCGR3B	NA	NA
GH1	2.1085009	0.82681608	0.52040198	GH1	NA	NA
HLA-DRB1	2.1210444	0.68540221	0.52040198	HLA-DRB1	NA	NA
NEK8	2.0962729	0.78434452	0.52040198	NEK8	NA	NA
PARP4	-2.0216843	1.56362627	0.52040198	PARP4	NA	NA
PCDHA3	2.3164964	0.74777155	0.52040198	PCDHA3	NA	NA
SPA17	2.2711195	0.77403425	0.52040198	SPA17	NA	NA
TUBG2	2.2009254	0.7412214	0.52040198	TUBG2	NA	NA

CAR 17vs19 q<0.01

SYMBOL	17vs19.d	17vs19.FC	17vs19.q	HAR	CAR	MAR
ALG10	-4.3960045	1.61044113	0	ALG10	ALG10	ALG10
CYorf15A	4.8826982	0.33984058	0	CYorf15A	CYorf15A	NA
DAZ3	3.1560883	0.66021176	0	DAZ3	DAZ3	NA
FAM27A	3.7260472	0.66240645	0	FAM27A	FAM27A	FAM27A
FRG1B	4.9790263	0.53342964	0	FRG1B	FRG1B	NA
GPC6	-2.9574624	1.70632811	0	GPC6	GPC6	GPC6
KGFLP1	-6.0498232	1.92952432	0	KGFLP1	KGFLP1	KGFLP1
LOC645166	2.9873503	0.70416815	0	LOC645166	LOC645166	NA
LOC654342	4.2238118	0.61249671	0	LOC654342	LOC654342	NA
MPDZ	-3.8035766	1.38811298	0	MPDZ	MPDZ	MPDZ
MYOM2	3.9185099	0.60750334	0	MYOM2	MYOM2	MYOM2
AHCY	3.8216127	0.6616698	0	NA	AHCY	AHCY
AK3	-3.9337709	1.62834195	0	NA	AK3	NA
ALDH1A2	-2.9144595	1.37007936	0	NA	ALDH1A2	ALDH1A2
ANXA3	-3.0609158	1.75438391	0	NA	ANXA3	ANXA3
ATM	-2.7124409	1.28498678	0	NA	ATM	NA
BAG5	-3.0836664	1.41818198	0	NA	BAG5	NA
BAT2	3.885736	0.67498407	0	NA	BAT2	NA
C17orf97	-3.4256608	1.89055616	0	NA	C17orf97	C17orf97
C20orf111	-3.2423874	1.39343486	0	NA	C20orf111	NA
CCL25	3.0998658	0.73888725	0	NA	CCL25	NA
CDH5	-2.9995755	1.29949249	0	NA	CDH5	CDH5
CLPTM1	3.4364856	0.68939056	0	NA	CLPTM1	NA
CTXN1	3.0496447	0.7261724	0	NA	CTXN1	NA
DDX12	3.2465719	0.69927974	0	NA	DDX12	NA
DDX3X	-2.9328472	1.28671605	0	NA	DDX3X	NA
DDX3Y	16.876416	0.17718856	0	NA	DDX3Y	DDX3Y
DNAJC14	-2.9630926	1.36956381	0	NA	DNAJC14	NA
FABP2	-3.1328576	1.50995037	0	NA	FABP2	FABP2
FMNL2	-2.7567558	1.43286398	0	NA	FMNL2	FMNL2
GJA1	-3.1733784	2.19155709	0	NA	GJA1	GJA1
GPR137B	-2.6931058	1.61477917	0	NA	GPR137B	NA
HBA1	-4.1478687	2.17084113	0	NA	HBA1	HBA1
HBM	-3.4403808	2.1078457	0	NA	HBM	HBM
HEATR3	-3.6981394	1.7535573	0	NA	HEATR3	HEATR3
LBH	-3.1891558	1.3292535	0	NA	LBH	LBH
LMNB1	-3.4886148	1.61615901	0	NA	LMNB1	LMNB1
LOC728411	3.7763355	0.60805245	0	NA	LOC728411	LOC728411
LRP1	3.4631467	0.73756441	0	NA	LRP1	LRP1
MAP9	-5.3005398	1.91553803	0	NA	MAP9	MAP9
MCM4	-2.715579	1.29650881	0	NA	MCM4	MCM4
NPEPPS	-2.8764258	1.23059526	0	NA	NPEPPS	NA
OLFML2B	-2.7124295	1.40929775	0	NA	OLFML2B	OLFML2B
ORMDL1	-5.0598578	1.60157912	0	NA	ORMDL1	NA
PCDH11Y	3.3331052	0.66928933	0	NA	PCDH11Y	PCDH11Y
PCDHA9	-9.0447639	4.22978343	0	NA	PCDHA9	NA
PCDHB2	-3.4170174	1.79354433	0	NA	PCDHB2	PCDHB2
PEX13	-4.0700533	1.51194967	0	NA	PEX13	PEX13
PKD1	2.9515426	0.77083577	0	NA	PKD1	NA
RASGEF1C	4.6942403	0.6096328	0	NA	RASGEF1C	NA
RPN2	-2.6682219	1.21872076	0	NA	RPN2	NA
RPS15	-4.1443443	1.46645326	0	NA	RPS15	NA
SBDSP	-2.6680322	1.32347965	0	NA	SBDSP	NA

SIGIRR	5.8151826	0.32670657	0	NA	SIGIRR	NA
SLC44A1	-3.7981917	1.47477665	0	NA	SLC44A1	SLC44A1
SNRNP40	-3.2647062	1.38873481	0	NA	SNRNP40	NA
SOX21	3.5374339	0.65163046	0	NA	SOX21	SOX21
SVIL	-2.9796019	1.44575569	0	NA	SVIL	SVIL
SYN1	3.6477506	0.66202785	0	NA	SYN1	NA
UTY	4.7598996	0.58514359	0	NA	UTY	NA
VAMP3	-3.8246818	1.30583534	0	NA	VAMP3	VAMP3
WDR86	3.2488551	0.69935269	0	NA	WDR86	WDR86
WNK3	-3.2041129	1.50150119	0	NA	WNK3	WNK3
ZBTB43	-2.9867948	1.28892076	0	NA	ZBTB43	NA
ZC3H4	-2.9450088	1.30078298	0	NA	ZC3H4	NA
ZFX	-2.982037	1.46067025	0	NA	ZFX	ZFX
ZNF217	-3.8102481	1.65942746	0	NA	ZNF217	ZNF217
ZNF580	3.355492	0.70024099	0	NA	ZNF580	NA
ZNF600	-2.9584113	1.45679969	0	NA	ZNF600	NA
SDPR	-3.040839	1.4477601	0	SDPR	SDPR	SDPR
TTTY15	16.499994	0.04287293	0	TTTY15	TTTY15	TTTY15
C7orf28B	-2.5054821	1.33288865	0.15408684	C7orf28B	C7orf28B	NA
DAZ1	2.8255536	0.75360375	0.15408684	DAZ1	DAZ1	NA
EDNRA	-2.5143927	1.50640502	0.15408684	EDNRA	EDNRA	EDNRA
ITIH5	-2.3664665	1.25547418	0.15408684	ITIH5	ITIH5	ITIH5
MAP3K7	-2.3125599	1.23669912	0.15408684	MAP3K7	MAP3K7	MAP3K7
AKAP12	-2.2786081	1.22442061	0.15408684	NA	AKAP12	AKAP12
BGN	-2.5541739	1.24956941	0.15408684	NA	BGN	NA
BMPR1B	2.6068905	0.57627958	0.15408684	NA	BMPR1B	BMPR1B
C6orf225	-2.4203083	1.2979177	0.15408684	NA	C6orf225	C6orf225
C7orf27	2.6845877	0.79922567	0.15408684	NA	C7orf27	NA
CCL2	-2.4253942	1.60984817	0.15408684	NA	CCL2	CCL2
CTNNB1	-2.3310371	1.55618518	0.15408684	NA	CTNNB1	CTNNB1
CUL2	2.8363592	0.7865667	0.15408684	NA	CUL2	CUL2
DGKZ	2.6512931	0.72415923	0.15408684	NA	DGKZ	NA
DPY19L2	-2.3058242	1.26838212	0.15408684	NA	DPY19L2	DPY19L2
EIF1AX	-2.2607281	1.24915554	0.15408684	NA	EIF1AX	EIF1AX
EMCN	-2.4724299	1.31793197	0.15408684	NA	EMCN	EMCN
EPB41L1	-2.5552412	1.24069766	0.15408684	NA	EPB41L1	EPB41L1
ESCO1	-2.5615491	1.28333929	0.15408684	NA	ESCO1	ESCO1
FASTKD2	-2.439101	1.25346273	0.15408684	NA	FASTKD2	FASTKD2
GLG1	-2.5581961	1.37991218	0.15408684	NA	GLG1	NA
GLRB	-2.5792542	1.28960532	0.15408684	NA	GLRB	GLRB
HCCS	-2.2828589	1.20536856	0.15408684	NA	HCCS	HCCS
HDDC2	2.513864	0.71840442	0.15408684	NA	HDDC2	HDDC2
HNRNPH1	-2.281457	1.32281047	0.15408684	NA	HNRNPH1	NA
HYI	-2.5821409	1.26010427	0.15408684	NA	HYI	HYI
KHDRBS2	-2.310516	1.28131427	0.15408684	NA	KHDRBS2	KHDRBS2
LHX3	2.5309149	0.71468102	0.15408684	NA	LHX3	NA
LOC100170939	-2.5776111	1.46453322	0.15408684	NA	LOC100170939	NA
LOC338758	-2.632777	1.41919053	0.15408684	NA	LOC338758	LOC338758
LOC723809	2.8695723	0.71704125	0.15408684	NA	LOC723809	LOC723809
MAPK8IP2	2.5229928	0.8036034	0.15408684	NA	MAPK8IP2	NA
NKAIN2	-2.4988171	1.40672174	0.15408684	NA	NKAIN2	NKAIN2
NOVA2	2.6609281	0.74595138	0.15408684	NA	NOVA2	NA
OTUB2	2.5639288	0.82291476	0.15408684	NA	OTUB2	NA
PDSS1	-2.5214532	1.28278209	0.15408684	NA	PDSS1	NA
PHB	-2.3630554	1.18715953	0.15408684	NA	PHB	PHB
PHF2	2.7253249	0.72802401	0.15408684	NA	PHF2	PHF2
PRPH2	2.8264552	0.70562354	0.15408684	NA	PRPH2	PRPH2
PTCHD2	2.5046776	0.7152472	0.15408684	NA	PTCHD2	NA

RP9	-2.5188738	1.24645567	0.15408684	NA	RP9	RP9
SSR3	-2.5990322	1.35165952	0.15408684	NA	SSR3	SSR3
SYPL2	2.7515259	0.65328867	0.15408684	NA	SYPL2	SYPL2
TM7SF4	2.6204674	0.77172555	0.15408684	NA	TM7SF4	TM7SF4
ZNF326	-2.3209629	1.23916508	0.15408684	NA	ZNF326	ZNF326
ZNF644	-2.5407901	1.55442586	0.15408684	NA	ZNF644	ZNF644
NFU1	2.5576059	0.78910103	0.15408684	NFU1	NFU1	NFU1
NKAIN3	2.9072777	0.80136794	0.15408684	NKAIN3	NKAIN3	NKAIN3
PKN2	-2.5086486	1.30100005	0.15408684	PKN2	PKN2	PKN2
PSAT1	-2.3191814	1.22755029	0.15408684	PSAT1	PSAT1	PSAT1
TARSL2	2.4999564	0.80142314	0.15408684	TARSL2	TARSL2	NA
WSB1	-2.3133712	1.38965777	0.15408684	WSB1	WSB1	WSB1
ATOH1	2.1058924	0.72609592	0.52040198	ATOH1	ATOH1	ATOH1
EFCAB4B	2.4987579	0.8501656	0.52040198	EFCAB4B	EFCAB4B	EFCAB4B
FAM96A	-1.9027157	1.18994558	0.52040198	FAM96A	FAM96A	FAM96A
FAT1	-2.1651627	1.28255083	0.52040198	FAT1	FAT1	FAT1
FRMD6	-2.2175814	1.30253868	0.52040198	FRMD6	FRMD6	FRMD6
KCTD12	-1.9045931	1.28329693	0.52040198	KCTD12	KCTD12	KCTD12
KDR	-2.1881049	1.32116515	0.52040198	KDR	KDR	KDR
LOC100132832	2.3888489	0.7146981	0.52040198	LOC1001328	LOC100132832	NA
LOC400891	-1.9715033	1.18672323	0.52040198	LOC400891	LOC400891	NA
ABCA17P	2.3343513	0.73624021	0.52040198	NA	ABCA17P	ABCA17P
ACTRT2	2.4225188	0.66197595	0.52040198	NA	ACTRT2	NA
APOL1	-1.9121602	1.38224102	0.52040198	NA	APOL1	APOL1
ARC	2.4962207	0.71036984	0.52040198	NA	ARC	NA
BMP10	2.4111758	0.67500925	0.52040198	NA	BMP10	NA
C11orf71	-2.0942639	1.19735443	0.52040198	NA	C11orf71	NA
C14orf178	2.1092287	0.78711472	0.52040198	NA	C14orf178	C14orf178
C14orf28	-1.9367375	1.22619677	0.52040198	NA	C14orf28	NA
C17orf44	2.3507042	0.66310725	0.52040198	NA	C17orf44	NA
C20orf152	2.0690105	0.81899879	0.52040198	NA	C20orf152	NA
C3orf17	-1.9128026	1.21356318	0.52040198	NA	C3orf17	C3orf17
C9orf69	2.3143464	0.81078734	0.52040198	NA	C9orf69	NA
CBLN3	2.0833671	0.78763001	0.52040198	NA	CBLN3	NA
CD47	-1.9775249	1.21294482	0.52040198	NA	CD47	CD47
CDC42	2.1906733	0.81494459	0.52040198	NA	CDC42	CDC42
CLDN15	2.1697741	0.83185557	0.52040198	NA	CLDN15	NA
CLN5	-1.9063071	1.27256393	0.52040198	NA	CLN5	CLN5
COL4A1	-2.0671428	1.22169476	0.52040198	NA	COL4A1	COL4A1
COL4A2	-2.2049807	1.18443796	0.52040198	NA	COL4A2	NA
COX7B	-2.1427705	1.31349944	0.52040198	NA	COX7B	NA
DEXI	2.1112593	0.83873045	0.52040198	NA	DEXI	DEXI
EIF2S3	-1.9601947	1.16509672	0.52040198	NA	EIF2S3	EIF2S3
FAM154B	-2.1218522	1.31465336	0.52040198	NA	FAM154B	NA
FAM184B	-2.240609	1.19153921	0.52040198	NA	FAM184B	NA
FLJ39653	-2.0964844	1.20766404	0.52040198	NA	FLJ39653	FLJ39653
GDE1	-2.1606586	1.20437995	0.52040198	NA	GDE1	NA
GJA4	-2.0965789	1.16984231	0.52040198	NA	GJA4	NA
GNL3L	-2.0766874	1.24010248	0.52040198	NA	GNL3L	NA
GPR144	-2.1131044	1.34267311	0.52040198	NA	GPR144	NA
HMG20A	-2.1217093	1.21264922	0.52040198	NA	HMG20A	NA
HRC	2.4465437	0.81970352	0.52040198	NA	HRC	NA
HS3ST4	-1.889094	1.23721883	0.52040198	NA	HS3ST4	HS3ST4
HSDL2	-1.8965978	1.29385895	0.52040198	NA	HSDL2	HSDL2
ICOS	2.4927746	0.75229582	0.52040198	NA	ICOS	ICOS
IDH2	-2.0983052	1.21860617	0.52040198	NA	IDH2	IDH2
IL13RA2	2.4185608	0.56894165	0.52040198	NA	IL13RA2	NA
KCNA2	2.1389919	0.81668435	0.52040198	NA	KCNA2	NA

KIAA0831	-2.1327187	1.19270868	0.52040198	NA	KIAA0831	KIAA0831
KRAS	-2.1983385	1.18710244	0.52040198	NA	KRAS	NA
LOC100192378	2.4134955	0.79027925	0.52040198	NA	LOC100192378	LOC100192378
LOC284805	2.363168	0.70057426	0.52040198	NA	LOC284805	NA
MCPH1	-2.1621202	1.1819172	0.52040198	NA	MCPH1	MCPH1
MDK	-1.9152052	1.32885191	0.52040198	NA	MDK	NA
MKKS	2.3111227	0.77454001	0.52040198	NA	MKKS	MKKS
MPPED1	-2.0119839	1.21751657	0.52040198	NA	MPPED1	MPPED1
MTHFS	-1.9253965	1.30214111	0.52040198	NA	MTHFS	NA
MXRA5	-1.9816178	1.41499961	0.52040198	NA	MXRA5	NA
NPBWR2	2.2708493	0.75492006	0.52040198	NA	NPBWR2	NA
NPDC1	2.3806903	0.76341094	0.52040198	NA	NPDC1	NA
NPIP	2.1970509	0.81624528	0.52040198	NA	NPIP	NA
PAFAH1B2	-1.8806275	1.17625486	0.52040198	NA	PAFAH1B2	PAFAH1B2
PCDH11X	2.1516949	0.84514434	0.52040198	NA	PCDH11X	PCDH11X
PCNXL2	2.0737151	0.79867141	0.52040198	NA	PCNXL2	PCNXL2
PDPK1	-1.8887228	1.13758817	0.52040198	NA	PDPK1	NA
PEX5L	-2.1640812	1.24539707	0.52040198	NA	PEX5L	PEX5L
PREP	-1.984032	1.275789	0.52040198	NA	PREP	PREP
PSMC3	-2.0114918	1.21585774	0.52040198	NA	PSMC3	NA
PTPN23	2.3028472	0.82481612	0.52040198	NA	PTPN23	NA
RAD1	-2.1804543	1.21625387	0.52040198	NA	RAD1	NA
RALGDS	2.1173299	0.82805109	0.52040198	NA	RALGDS	NA
RARA	2.487077	0.76487883	0.52040198	NA	RARA	RARA
RBM42	2.2347514	0.77108967	0.52040198	NA	RBM42	NA
RLTPR	2.3650698	0.79681502	0.52040198	NA	RLTPR	RLTPR
ROBO3	2.3069616	0.74089878	0.52040198	NA	ROBO3	ROBO3
RTN3	-1.953676	1.15159896	0.52040198	NA	RTN3	NA
RUFY3	-1.9157856	1.19877545	0.52040198	NA	RUFY3	RUFY3
SCNN1D	2.2105416	0.78447523	0.52040198	NA	SCNN1D	NA
SCRN3	-2.102817	1.23760316	0.52040198	NA	SCRN3	SCRN3
SFTPD	2.3522799	0.8057268	0.52040198	NA	SFTPD	NA
SIPA1L1	-2.0250012	1.19199125	0.52040198	NA	SIPA1L1	SIPA1L1
SLC16A7	-2.1144529	1.4193732	0.52040198	NA	SLC16A7	SLC16A7
SLC29A3	2.3957502	0.77489056	0.52040198	NA	SLC29A3	SLC29A3
SS18	-1.9681358	1.22695495	0.52040198	NA	SS18	SS18
SSR4	-1.9712245	1.19889846	0.52040198	NA	SSR4	NA
SUMF1	-2.1038277	1.21929102	0.52040198	NA	SUMF1	SUMF1
SYCE2	2.2061146	0.75924301	0.52040198	NA	SYCE2	NA
TCAP	-1.9631978	1.25541931	0.52040198	NA	TCAP	NA
TEX28	2.2745937	0.75567848	0.52040198	NA	TEX28	NA
TFDP3	2.2307515	0.76327553	0.52040198	NA	TFDP3	TFDP3
TIAF1	2.059115	0.83706419	0.52040198	NA	TIAF1	NA
TM4SF18	-1.8990657	1.3332277	0.52040198	NA	TM4SF18	NA
TMCC1	-1.975072	1.16245899	0.52040198	NA	TMCC1	TMCC1
TMEM106B	-2.0505189	1.30611139	0.52040198	NA	TMEM106B	TMEM106B
TMEM187	-1.9812326	1.30434616	0.52040198	NA	TMEM187	NA
TTC39B	-2.1480051	1.21699674	0.52040198	NA	TTC39B	TTC39B
USP39	-2.1130153	1.22255532	0.52040198	NA	USP39	NA
VGLL3	-1.8927667	1.23418995	0.52040198	NA	VGLL3	VGLL3
VRK1	-2.0220907	1.19558435	0.52040198	NA	VRK1	VRK1
WDR6	2.2784428	0.79289306	0.52040198	NA	WDR6	NA
XIAP	-1.9443922	1.16880732	0.52040198	NA	XIAP	XIAP
ZNF155	-2.0373414	1.22108449	0.52040198	NA	ZNF155	NA
NHLH2	-1.9479143	1.31275162	0.52040198	NHLH2	NHLH2	NA
PLXNA2	-1.9743795	1.4904532	0.52040198	PLXNA2	PLXNA2	PLXNA2
TLE4	-2.2209082	1.42075177	0.52040198	TLE4	TLE4	TLE4

ZNF200	-2.1070827	1.20967709	0.52040198	ZNF200	ZNF200	NA

CAR-HAR 17vs19 q<0.01

SYMBOL	17vs19.d	17vs19.FC	17vs19.q	HAR	CAR	MAR
AHCY	3.8216127	0.6616698	0	NA	AHCY	AHCY
ALDH1A2	-2.9144595	1.37007936	0	NA	ALDH1A2	ALDH1A2
ANXA3	-3.0609158	1.75438391	0	NA	ANXA3	ANXA3
C17orf97	-3.4256608	1.89055616	0	NA	C17orf97	C17orf97
CDH5	-2.9995755	1.29949249	0	NA	CDH5	CDH5
DDX3Y	16.876416	0.17718856	0	NA	DDX3Y	DDX3Y
FABP2	-3.1328576	1.50995037	0	NA	FABP2	FABP2
FMNL2	-2.7567558	1.43286398	0	NA	FMNL2	FMNL2
GJA1	-3.1733784	2.19155709	0	NA	GJA1	GJA1
HBA1	-4.1478687	2.17084113	0	NA	HBA1	HBA1
HBM	-3.4403808	2.1078457	0	NA	HBM	HBM
HEATR3	-3.6981394	1.7535573	0	NA	HEATR3	HEATR3
LBH	-3.1891558	1.3292535	0	NA	LBH	LBH
LMNB1	-3.4886148	1.61615901	0	NA	LMNB1	LMNB1
LOC728411	3.7763355	0.60805245	0	NA	LOC728411	LOC728411
LRP1	3.4631467	0.73756441	0	NA	LRP1	LRP1
MAP9	-5.3005398	1.91553803	0	NA	MAP9	MAP9
MCM4	-2.715579	1.29650881	0	NA	MCM4	MCM4
AK3	-3.9337709	1.62834195	0	NA	AK3	NA
ATM	-2.7124409	1.28498678	0	NA	ATM	NA
BAG5	-3.0836664	1.41818198	0	NA	BAG5	NA
BAT2	3.885736	0.67498407	0	NA	BAT2	NA
C20orf111	-3.2423874	1.39343486	0	NA	C20orf111	NA
CCL25	3.0998658	0.73888725	0	NA	CCL25	NA
CLPTM1	3.4364856	0.68939056	0	NA	CLPTM1	NA
CTXN1	3.0496447	0.7261724	0	NA	CTXN1	NA
DDX12	3.2465719	0.69927974	0	NA	DDX12	NA
DDX3X	-2.9328472	1.28671605	0	NA	DDX3X	NA
DNAJC14	-2.9630926	1.36956381	0	NA	DNAJC14	NA
GPR137B	-2.6931058	1.61477917	0	NA	GPR137B	NA
NPEPPS	-2.8764258	1.23059526	0	NA	NPEPPS	NA
ORMDL1	-5.0598578	1.60157912	0	NA	ORMDL1	NA
PCDHA9	-9.0447639	4.22978343	0	NA	PCDHA9	NA
PKD1	2.9515426	0.77083577	0	NA	PKD1	NA
RASGEF1C	4.6942403	0.6096328	0	NA	RASGEF1C	NA
RPN2	-2.6682219	1.21872076	0	NA	RPN2	NA
RPS15	-4.1443443	1.46645326	0	NA	RPS15	NA
SBDSP	-2.6680322	1.32347965	0	NA	SBDSP	NA
SIGIRR	5.8151826	0.32670657	0	NA	SIGIRR	NA
SNRNP40	-3.2647062	1.38873481	0	NA	SNRNP40	NA
SYN1	3.6477506	0.66202785	0	NA	SYN1	NA
UTY	4.7598996	0.58514359	0	NA	UTY	NA
ZBTB43	-2.9867948	1.28892076	0	NA	ZBTB43	NA
ZC3H4	-2.9450088	1.30078298	0	NA	ZC3H4	NA
ZNF580	3.355492	0.70024099	0	NA	ZNF580	NA
ZNF600	-2.9584113	1.45679969	0	NA	ZNF600	NA
OLFML2B	-2.7124295	1.40929775	0	NA	OLFML2B	OLFML2B
PCDH11Y	3.3331052	0.66928933	0	NA	PCDH11Y	PCDH11Y
PCDHB2	-3.4170174	1.79354433	0	NA	PCDHB2	PCDHB2

PEX13	-4.0700533	1.51194967	0	NA	PEX13	PEX13
SLC44A1	-3.7981917	1.47477665	0	NA	SLC44A1	SLC44A1
SOX21	3.5374339	0.65163046	0	NA	SOX21	SOX21
SVIL	-2.9796019	1.44575569	0	NA	SVIL	SVIL
VAMP3	-3.8246818	1.30583534	0	NA	VAMP3	VAMP3
WDR86	3.2488551	0.69935269	0	NA	WDR86	WDR86
WNK3	-3.2041129	1.50150119	0	NA	WNK3	WNK3
ZFX	-2.982037	1.46067025	0	NA	ZFX	ZFX
ZNF217	-3.8102481	1.65942746	0	NA	ZNF217	ZNF217
AKAP12	-2.2786081	1.22442061	0.15408684	NA	AKAP12	AKAP12
BMPR1B	2.6068905	0.57627958	0.15408684	NA	BMPR1B	BMPR1B
C6orf225	-2.4203083	1.2979177	0.15408684	NA	C6orf225	C6orf225
CCL2	-2.4253942	1.60984817	0.15408684	NA	CCL2	CCL2
CTNNB1	-2.3310371	1.55618518	0.15408684	NA	CTNNB1	CTNNB1
CUL2	2.8363592	0.7865667	0.15408684	NA	CUL2	CUL2
DPY19L2	-2.3058242	1.26838212	0.15408684	NA	DPY19L2	DPY19L2
EIF1AX	-2.2607281	1.24915554	0.15408684	NA	EIF1AX	EIF1AX
EMCN	-2.4724299	1.31793197	0.15408684	NA	EMCN	EMCN
EPB41L1	-2.5552412	1.24069766	0.15408684	NA	EPB41L1	EPB41L1
ESCO1	-2.5615491	1.28333929	0.15408684	NA	ESCO1	ESCO1
FASTKD2	-2.439101	1.25346273	0.15408684	NA	FASTKD2	FASTKD2
GLRB	-2.5792542	1.28960532	0.15408684	NA	GLRB	GLRB
HCCS	-2.2828589	1.20536856	0.15408684	NA	HCCS	HCCS
HDDC2	2.513864	0.71840442	0.15408684	NA	HDDC2	HDDC2
HYI	-2.5821409	1.26010427	0.15408684	NA	HYI	HYI
KHDRBS2	-2.310516	1.28131427	0.15408684	NA	KHDRBS2	KHDRBS2
LOC338758	-2.632777	1.41919053	0.15408684	NA	LOC338758	LOC338758
LOC723809	2.8695723	0.71704125	0.15408684	NA	LOC723809	LOC723809
BGN	-2.5541739	1.24956941	0.15408684	NA	BGN	NA
C7orf27	2.6845877	0.79922567	0.15408684	NA	C7orf27	NA
DGKZ	2.6512931	0.72415923	0.15408684	NA	DGKZ	NA
GLG1	-2.5581961	1.37991218	0.15408684	NA	GLG1	NA
HNRNPH1	-2.281457	1.32281047	0.15408684	NA	HNRNPH1	NA
LHX3	2.5309149	0.71468102	0.15408684	NA	LHX3	NA
LOC100170939	-2.5776111	1.46453322	0.15408684	NA	LOC100170939	NA
MAPK8IP2	2.5229928	0.8036034	0.15408684	NA	MAPK8IP2	NA
NOVA2	2.6609281	0.74595138	0.15408684	NA	NOVA2	NA
OTUB2	2.5639288	0.82291476	0.15408684	NA	OTUB2	NA
PDSS1	-2.5214532	1.28278209	0.15408684	NA	PDSS1	NA
PTCHD2	2.5046776	0.7152472	0.15408684	NA	PTCHD2	NA
NKAIN2	-2.4988171	1.40672174	0.15408684	NA	NKAIN2	NKAIN2
PHB	-2.3630554	1.18715953	0.15408684	NA	PHB	PHB
PHF2	2.7253249	0.72802401	0.15408684	NA	PHF2	PHF2
PRPH2	2.8264552	0.70562354	0.15408684	NA	PRPH2	PRPH2
RP9	-2.5188738	1.24645567	0.15408684	NA	RP9	RP9
SSR3	-2.5990322	1.35165952	0.15408684	NA	SSR3	SSR3
SYPL2	2.7515259	0.65328867	0.15408684	NA	SYPL2	SYPL2
TM7SF4	2.6204674	0.77172555	0.15408684	NA	TM7SF4	TM7SF4
ZNF326	-2.3209629	1.23916508	0.15408684	NA	ZNF326	ZNF326
ZNF644	-2.5407901	1.55442586	0.15408684	NA	ZNF644	ZNF644
ABCA17P	2.3343513	0.73624021	0.52040198	NA	ABCA17P	ABCA17P
APOL1	-1.9121602	1.38224102	0.52040198	NA	APOL1	APOL1
C14orf178	2.1092287	0.78711472	0.52040198	NA	C14orf178	C14orf178
C3orf17	-1.9128026	1.21356318	0.52040198	NA	C3orf17	C3orf17
CD47	-1.9775249	1.21294482	0.52040198	NA	CD47	CD47
CDC42	2.1906733	0.81494459	0.52040198	NA	CDC42	CDC42
CLN5	-1.9063071	1.27256393	0.52040198	NA	CLN5	CLN5
COL4A1	-2.0671428	1.22169476	0.52040198	NA	COL4A1	COL4A1

DEXI	2.1112593	0.83873045	0.52040198	NA	DEXI	DEXI
EIF2S3	-1.9601947	1.16509672	0.52040198	NA	EIF2S3	EIF2S3
FLJ39653	-2.0964844	1.20766404	0.52040198	NA	FLJ39653	FLJ39653
HS3ST4	-1.889094	1.23721883	0.52040198	NA	HS3ST4	HS3ST4
HSDL2	-1.8965978	1.29385895	0.52040198	NA	HSDL2	HSDL2
ICOS	2.4927746	0.75229582	0.52040198	NA	ICOS	ICOS
IDH2	-2.0983052	1.21860617	0.52040198	NA	IDH2	IDH2
KIAA0831	-2.1327187	1.19270868	0.52040198	NA	KIAA0831	KIAA0831
LOC100192378	2.4134955	0.79027925	0.52040198	NA	LOC10019237<br>8	LOC10019237<br>8
MCPH1	-2.1621202	1.1819172	0.52040198	NA	MCPH1	MCPH1
MKKS	2.3111227	0.77454001	0.52040198	NA	MKKS	MKKS
MPPED1	-2.0119839	1.21751657	0.52040198	NA	MPPED1	MPPED1
ACTRT2	2.4225188	0.66197595	0.52040198	NA	ACTRT2	NA
ARC	2.4962207	0.71036984	0.52040198	NA	ARC	NA
BMP10	2.4111758	0.67500925	0.52040198	NA	BMP10	NA
C11orf71	-2.0942639	1.19735443	0.52040198	NA	C11orf71	NA
C14orf28	-1.9367375	1.22619677	0.52040198	NA	C14orf28	NA
C17orf44	2.3507042	0.66310725	0.52040198	NA	C17orf44	NA
C20orf152	2.0690105	0.81899879	0.52040198	NA	C20orf152	NA
C9orf69	2.3143464	0.81078734	0.52040198	NA	C9orf69	NA
CBLN3	2.0833671	0.78763001	0.52040198	NA	CBLN3	NA
CLDN15	2.1697741	0.83185557	0.52040198	NA	CLDN15	NA
COL4A2	-2.2049807	1.18443796	0.52040198	NA	COL4A2	NA
COX7B	-2.1427705	1.31349944	0.52040198	NA	COX7B	NA
FAM154B	-2.1218522	1.31465336	0.52040198	NA	FAM154B	NA
FAM184B	-2.240609	1.19153921	0.52040198	NA	FAM184B	NA
GDE1	-2.1606586	1.20437995	0.52040198	NA	GDE1	NA
GJA4	-2.0965789	1.16984231	0.52040198	NA	GJA4	NA
GNL3L	-2.0766874	1.24010248	0.52040198	NA	GNL3L	NA
GPR144	-2.1131044	1.34267311	0.52040198	NA	GPR144	NA
HMG20A	-2.1217093	1.21264922	0.52040198	NA	HMG20A	NA
HRC	2.4465437	0.81970352	0.52040198	NA	HRC	NA
IL13RA2	2.4185608	0.56894165	0.52040198	NA	IL13RA2	NA
KCNA2	2.1389919	0.81668435	0.52040198	NA	KCNA2	NA
KRAS	-2.1983385	1.18710244	0.52040198	NA	KRAS	NA
LOC284805	2.363168	0.70057426	0.52040198	NA	LOC284805	NA
MDK	-1.9152052	1.32885191	0.52040198	NA	MDK	NA
MTHFS	-1.9253965	1.30214111	0.52040198	NA	MTHFS	NA
MXRA5	-1.9816178	1.41499961	0.52040198	NA	MXRA5	NA
NPBWR2	2.2708493	0.75492006	0.52040198	NA	NPBWR2	NA
NPDC1	2.3806903	0.76341094	0.52040198	NA	NPDC1	NA
NPIP	2.1970509	0.81624528	0.52040198	NA	NPIP	NA
PDPK1	-1.8887228	1.13758817	0.52040198	NA	PDPK1	NA
PSMC3	-2.0114918	1.21585774	0.52040198	NA	PSMC3	NA
PTPN23	2.3028472	0.82481612	0.52040198	NA	PTPN23	NA
RAD1	-2.1804543	1.21625387	0.52040198	NA	RAD1	NA
RALGDS	2.1173299	0.82805109	0.52040198	NA	RALGDS	NA
RBM42	2.2347514	0.77108967	0.52040198	NA	RBM42	NA
RTN3	-1.953676	1.15159896	0.52040198	NA	RTN3	NA
SCNN1D	2.2105416	0.78447523	0.52040198	NA	SCNN1D	NA
SFTPD	2.3522799	0.8057268	0.52040198	NA	SFTPD	NA
SSR4	-1.9712245	1.19889846	0.52040198	NA	SSR4	NA
SYCE2	2.2061146	0.75924301	0.52040198	NA	SYCE2	NA
TCAP	-1.9631978	1.25541931	0.52040198	NA	TCAP	NA
TEX28	2.2745937	0.75567848	0.52040198	NA	TEX28	NA
TIAF1	2.059115	0.83706419	0.52040198	NA	TIAF1	NA
TM4SF18	-1.8990657	1.3332277	0.52040198	NA	TM4SF18	NA

TMEM187	-1.9812326	1.30434616	0.52040198	NA	TMEM187	NA
USP39	-2.1130153	1.22255532	0.52040198	NA	USP39	NA
WDR6	2.2784428	0.79289306	0.52040198	NA	WDR6	NA
ZNF155	-2.0373414	1.22108449	0.52040198	NA	ZNF155	NA
PAFAH1B2	-1.8806275	1.17625486	0.52040198	NA	PAFAH1B2	PAFAH1B2
PCDH11X	2.1516949	0.84514434	0.52040198	NA	PCDH11X	PCDH11X
PCNXL2	2.0737151	0.79867141	0.52040198	NA	PCNXL2	PCNXL2
PEX5L	-2.1640812	1.24539707	0.52040198	NA	PEX5L	PEX5L
PREP	-1.984032	1.275789	0.52040198	NA	PREP	PREP
RARA	2.487077	0.76487883	0.52040198	NA	RARA	RARA
RLTPR	2.3650698	0.79681502	0.52040198	NA	RLTPR	RLTPR
ROBO3	2.3069616	0.74089878	0.52040198	NA	ROBO3	ROBO3
RUFY3	-1.9157856	1.19877545	0.52040198	NA	RUFY3	RUFY3
SCRN3	-2.102817	1.23760316	0.52040198	NA	SCRN3	SCRN3
SIPA1L1	-2.0250012	1.19199125	0.52040198	NA	SIPA1L1	SIPA1L1
SLC16A7	-2.1144529	1.4193732	0.52040198	NA	SLC16A7	SLC16A7
SLC29A3	2.3957502	0.77489056	0.52040198	NA	SLC29A3	SLC29A3
SS18	-1.9681358	1.22695495	0.52040198	NA	SS18	SS18
SUMF1	-2.1038277	1.21929102	0.52040198	NA	SUMF1	SUMF1
TFDP3	2.2307515	0.76327553	0.52040198	NA	TFDP3	TFDP3
TMCC1	-1.975072	1.16245899	0.52040198	NA	TMCC1	TMCC1
TMEM106B	-2.0505189	1.30611139	0.52040198	NA	TMEM106B	TMEM106B
TTC39B	-2.1480051	1.21699674	0.52040198	NA	TTC39B	TTC39B
VGLL3	-1.8927667	1.23418995	0.52040198	NA	VGLL3	VGLL3
VRK1	-2.0220907	1.19558435	0.52040198	NA	VRK1	VRK1
XIAP	-1.9443922	1.16880732	0.52040198	NA	XIAP	XIAP

CAR only 17vs19 q<0.01

SYMBOL	17vs19.d	17vs19.FC	17vs19.q	HAR	CAR	MAR
AK3	-3.9337709	1.62834195	0	NA	AK3	NA
ATM	-2.7124409	1.28498678	0	NA	ATM	NA
BAG5	-3.0836664	1.41818198	0	NA	BAG5	NA
BAT2	3.885736	0.67498407	0	NA	BAT2	NA
C20orf111	-3.2423874	1.39343486	0	NA	C20orf111	NA
CCL25	3.0998658	0.73888725	0	NA	CCL25	NA
CLPTM1	3.4364856	0.68939056	0	NA	CLPTM1	NA
CTXN1	3.0496447	0.7261724	0	NA	CTXN1	NA
DDX12	3.2465719	0.69927974	0	NA	DDX12	NA
DDX3X	-2.9328472	1.28671605	0	NA	DDX3X	NA
DNAJC14	-2.9630926	1.36956381	0	NA	DNAJC14	NA
GPR137B	-2.6931058	1.61477917	0	NA	GPR137B	NA
NPEPPS	-2.8764258	1.23059526	0	NA	NPEPPS	NA
ORMDL1	-5.0598578	1.60157912	0	NA	ORMDL1	NA
PCDHA9	-9.0447639	4.22978343	0	NA	PCDHA9	NA
PKD1	2.9515426	0.77083577	0	NA	PKD1	NA
RASGEF1C	4.6942403	0.6096328	0	NA	RASGEF1C	NA
RPN2	-2.6682219	1.21872076	0	NA	RPN2	NA
RPS15	-4.1443443	1.46645326	0	NA	RPS15	NA
SBDSP	-2.6680322	1.32347965	0	NA	SBDSP	NA
SIGIRR	5.8151826	0.32670657	0	NA	SIGIRR	NA
SNRNP40	-3.2647062	1.38873481	0	NA	SNRNP40	NA
SYN1	3.6477506	0.66202785	0	NA	SYN1	NA
UTY	4.7598996	0.58514359	0	NA	UTY	NA

ZBTB43	-2.9867948	1.28892076	0	NA	ZBTB43	NA
ZC3H4	-2.9450088	1.30078298	0	NA	ZC3H4	NA
ZNF580	3.355492	0.70024099	0	NA	ZNF580	NA
ZNF600	-2.9584113	1.45679969	0	NA	ZNF600	NA
BGN	-2.5541739	1.24956941	0.15408684	NA	BGN	NA
C7orf27	2.6845877	0.79922567	0.15408684	NA	C7orf27	NA
DGKZ	2.6512931	0.72415923	0.15408684	NA	DGKZ	NA
GLG1	-2.5581961	1.37991218	0.15408684	NA	GLG1	NA
HNRNPH1	-2.281457	1.32281047	0.15408684	NA	HNRNPH1	NA
LHX3	2.5309149	0.71468102	0.15408684	NA	LHX3	NA
LOC100170939	-2.5776111	1.46453322	0.15408684	NA	LOC100170939	NA
MAPK8IP2	2.5229928	0.8036034	0.15408684	NA	MAPK8IP2	NA
NOVA2	2.6609281	0.74595138	0.15408684	NA	NOVA2	NA
OTUB2	2.5639288	0.82291476	0.15408684	NA	OTUB2	NA
PDSS1	-2.5214532	1.28278209	0.15408684	NA	PDSS1	NA
PTCHD2	2.5046776	0.7152472	0.15408684	NA	PTCHD2	NA
ACTRT2	2.4225188	0.66197595	0.52040198	NA	ACTRT2	NA
ARC	2.4962207	0.71036984	0.52040198	NA	ARC	NA
BMP10	2.4111758	0.67500925	0.52040198	NA	BMP10	NA
C11orf71	-2.0942639	1.19735443	0.52040198	NA	C11orf71	NA
C14orf28	-1.9367375	1.22619677	0.52040198	NA	C14orf28	NA
C17orf44	2.3507042	0.66310725	0.52040198	NA	C17orf44	NA
C20orf152	2.0690105	0.81899879	0.52040198	NA	C20orf152	NA
C9orf69	2.3143464	0.81078734	0.52040198	NA	C9orf69	NA
CBLN3	2.0833671	0.78763001	0.52040198	NA	CBLN3	NA
CLDN15	2.1697741	0.83185557	0.52040198	NA	CLDN15	NA
COL4A2	-2.2049807	1.18443796	0.52040198	NA	COL4A2	NA
COX7B	-2.1427705	1.31349944	0.52040198	NA	COX7B	NA
FAM154B	-2.1218522	1.31465336	0.52040198	NA	FAM154B	NA
FAM184B	-2.240609	1.19153921	0.52040198	NA	FAM184B	NA
GDE1	-2.1606586	1.20437995	0.52040198	NA	GDE1	NA
GJA4	-2.0965789	1.16984231	0.52040198	NA	GJA4	NA
GNL3L	-2.0766874	1.24010248	0.52040198	NA	GNL3L	NA
GPR144	-2.1131044	1.34267311	0.52040198	NA	GPR144	NA
HMG20A	-2.1217093	1.21264922	0.52040198	NA	HMG20A	NA
HRC	2.4465437	0.81970352	0.52040198	NA	HRC	NA
IL13RA2	2.4185608	0.56894165	0.52040198	NA	IL13RA2	NA
KCNA2	2.1389919	0.81668435	0.52040198	NA	KCNA2	NA
KRAS	-2.1983385	1.18710244	0.52040198	NA	KRAS	NA
LOC284805	2.363168	0.70057426	0.52040198	NA	LOC284805	NA
MDK	-1.9152052	1.32885191	0.52040198	NA	MDK	NA
MTHFS	-1.9253965	1.30214111	0.52040198	NA	MTHFS	NA
MXRA5	-1.9816178	1.41499961	0.52040198	NA	MXRA5	NA
NPBWR2	2.2708493	0.75492006	0.52040198	NA	NPBWR2	NA
NPDC1	2.3806903	0.76341094	0.52040198	NA	NPDC1	NA
NPIP	2.1970509	0.81624528	0.52040198	NA	NPIP	NA
PDPK1	-1.8887228	1.13758817	0.52040198	NA	PDPK1	NA
PSMC3	-2.0114918	1.21585774	0.52040198	NA	PSMC3	NA
PTPN23	2.3028472	0.82481612	0.52040198	NA	PTPN23	NA
RAD1	-2.1804543	1.21625387	0.52040198	NA	RAD1	NA
RALGDS	2.1173299	0.82805109	0.52040198	NA	RALGDS	NA
RBM42	2.2347514	0.77108967	0.52040198	NA	RBM42	NA
RTN3	-1.953676	1.15159896	0.52040198	NA	RTN3	NA
SCNN1D	2.2105416	0.78447523	0.52040198	NA	SCNN1D	NA
SFTPD	2.3522799	0.8057268	0.52040198	NA	SFTPD	NA
SSR4	-1.9712245	1.19889846	0.52040198	NA	SSR4	NA
SYCE2	2.2061146	0.75924301	0.52040198	NA	SYCE2	NA
TCAP	-1.9631978	1.25541931	0.52040198	NA	TCAP	NA

TEX28	2.2745937	0.75567848	0.52040198	NA	TEX28	NA
TIAF1	2.059115	0.83706419	0.52040198	NA	TIAF1	NA
TM4SF18	-1.8990657	1.3332277	0.52040198	NA	TM4SF18	NA
TMEM187	-1.9812326	1.30434616	0.52040198	NA	TMEM187	NA
USP39	-2.1130153	1.22255532	0.52040198	NA	USP39	NA
WDR6	2.2784428	0.79289306	0.52040198	NA	WDR6	NA
ZNF155	-2.0373414	1.22108449	0.52040198	NA	ZNF155	NA

MAR 17vs19 q<0.01

SYMBOL	17vs19.d	17vs19.FC	17vs19.q	HAR	CAR	MAR
AFAP1L2	-3.1976804	1.61900834	0	AFAP1L2	NA	AFAP1L2
ALG10	-4.3960045	1.61044113	0	ALG10	ALG10	ALG10
CDR2	-4.3926751	1.59802619	0	CDR2	NA	CDR2
DAD1	-3.4090834	1.42475318	0	DAD1	NA	DAD1
DKK2	-2.7972101	1.54347974	0	DKK2	NA	DKK2
FAM19A2	3.0791036	0.75560504	0	FAM19A2	NA	FAM19A2
FAM27A	3.7260472	0.66240645	0	FAM27A	FAM27A	FAM27A
FOXC1	-3.4526707	1.89779589	0	FOXC1	NA	FOXC1
GPC6	-2.9574624	1.70632811	0	GPC6	GPC6	GPC6
KGFLP1	-6.0498232	1.92952432	0	KGFLP1	KGFLP1	KGFLP1
KLF5	-3.3245929	1.31204257	0	KLF5	NA	KLF5
LOC654433	6.1800862	0.38815097	0	LOC654433	NA	LOC654433
MPDZ	-3.8035766	1.38811298	0	MPDZ	MPDZ	MPDZ
MYOM2	3.9185099	0.60750334	0	MYOM2	MYOM2	MYOM2
ACP6	4.0312994	0.65072026	0	NA	NA	ACP6
AHCY	3.8216127	0.6616698	0	NA	AHCY	AHCY
ALDH1A2	-2.9144595	1.37007936	0	NA	ALDH1A2	ALDH1A2
AMFR	-8.7147112	2.11275886	0	NA	NA	AMFR
ANXA3	-3.0609158	1.75438391	0	NA	ANXA3	ANXA3
ASAH2B	3.1456685	0.69224484	0	NA	NA	ASAH2B
ATG12	-2.7573778	1.34585972	0	NA	NA	ATG12
B4GALT5	-4.2003741	1.4674269	0	NA	NA	B4GALT5
BBX	-2.725413	1.50335278	0	NA	NA	BBX
BCL11B	-3.0643644	1.39138997	0	NA	NA	BCL11B
BEND3	-2.9661982	1.24150406	0	NA	NA	BEND3
BOC	-2.8068475	1.27656318	0	NA	NA	BOC
C12orf75	3.5206713	0.67780015	0	NA	NA	C12orf75
C14orf101	-3.3229101	1.38652116	0	NA	NA	C14orf101
C17orf75	-2.9644849	1.77741209	0	NA	NA	C17orf75
C17orf82	3.0510839	0.71348687	0	NA	NA	C17orf82
C17orf97	-3.4256608	1.89055616	0	NA	C17orf97	C17orf97
C18orf19	-3.0628472	1.35442251	0	NA	NA	C18orf19
C1orf156	-4.0044376	1.62573154	0	NA	NA	C1orf156
C20orf94	-2.8759556	1.3876158	0	NA	NA	C20orf94
C21orf57	7.4867579	0.47059106	0	NA	NA	C21orf57
C2orf74	-6.9874199	1.97745048	0	NA	NA	C2orf74
C3orf70	-3.7496884	1.67548683	0	NA	NA	C3orf70
C8orf44	-2.7352321	1.43097518	0	NA	NA	C8orf44
C8orf59	-2.7116814	1.29717691	0	NA	NA	C8orf59
CACNB4	-8.4974559	2.93779503	0	NA	NA	CACNB4
CCDC59	-3.0452841	1.3867423	0	NA	NA	CCDC59
CCND1	-4.7674023	1.57837942	0	NA	NA	CCND1
CCNJ	-3.0257177	1.38453169	0	NA	NA	CCNJ
CDH1	-3.9085644	1.52767902	0	NA	NA	CDH1

CDH5	-2.9995755	1.29949249	0	NA	CDH5	CDH5
CDKAL1	-6.8380691	1.93235063	0	NA	NA	CDKAL1
CLDN11	-2.8676812	1.53788098	0	NA	NA	CLDN11
COL3A1	-3.5343766	1.53911674	0	NA	NA	COL3A1
CRBN	-4.4936872	1.31982838	0	NA	NA	CRBN
CRH	3.942527	0.52240886	0	NA	NA	CRH
DARC	4.2546692	0.60437945	0	NA	NA	DARC
DCN	-2.7933295	1.41786959	0	NA	NA	DCN
DDX17	-15.817588	9.77010381	0	NA	NA	DDX17
DDX3Y	16.876416	0.17718856	0	NA	DDX3Y	DDX3Y
DEM1	-4.1108629	1.39193028	0	NA	NA	DEM1
DKK1	-3.6500275	1.68388797	0	NA	NA	DKK1
DOCK7	-5.7660623	1.62898159	0	NA	NA	DOCK7
DSP	-2.7089628	1.75689966	0	NA	NA	DSP
EGR1	-3.2586722	1.70147338	0	NA	NA	EGR1
EXT1	-2.7939417	1.33590226	0	NA	NA	EXT1
EYA1	-3.2278247	1.50514894	0	NA	NA	EYA1
FABP2	-3.1328576	1.50995037	0	NA	FABP2	FABP2
FAM110B	-2.71985	1.25767091	0	NA	NA	FAM110B
FAM129A	-3.7403437	1.4648489	0	NA	NA	FAM129A
FAM178B	3.3666818	0.67561962	0	NA	NA	FAM178B
FAM46C	-6.0599677	2.33991468	0	NA	NA	FAM46C
FAM60A	-2.842808	1.5858925	0	NA	NA	FAM60A
FBLN5	-2.8513505	1.67224398	0	NA	NA	FBLN5
FLJ32063	3.4212591	0.74778534	0	NA	NA	FLJ32063
FMNL2	-2.7567558	1.43286398	0	NA	FMNL2	FMNL2
FOS	-3.3201611	2.17313695	0	NA	NA	FOS
FUT11	-3.36033	1.34288763	0	NA	NA	FUT11
FZD3	-4.9729828	2.70223052	0	NA	NA	FZD3
GAS2	-3.0464526	1.65391062	0	NA	NA	GAS2
GATC	-4.9569503	1.5386214	0	NA	NA	GATC
GJA1	-3.1733784	2.19155709	0	NA	GJA1	GJA1
GJC1	-2.8228101	1.41752178	0	NA	NA	GJC1
GK3P	-3.041541	1.40839884	0	NA	NA	GK3P
GLCE	-3.297914	1.36107722	0	NA	NA	GLCE
GNG11	-4.6550523	1.60596159	0	NA	NA	GNG11
GNG2	3.3049392	0.67064371	0	NA	NA	GNG2
GOLGA1	-2.8694446	1.38471146	0	NA	NA	GOLGA1
GPR155	-2.9358654	1.32266431	0	NA	NA	GPR155
GRIN2B	-2.7893494	1.86405719	0	NA	NA	GRIN2B
GSTT1	14.567224	0.29536778	0	NA	NA	GSTT1
GYPA	-3.3398883	1.50730896	0	NA	NA	GYPA
HADHB	-3.5500149	1.30255086	0	NA	NA	HADHB
HAS3	-2.7170941	1.34573993	0	NA	NA	HAS3
HBA1	-4.1478687	2.17084113	0	NA	HBA1	HBA1
HBB	-6.0753265	3.07303904	0	NA	NA	HBB
HBG1	-5.0919514	2.4485439	0	NA	NA	HBG1
HBM	-3.4403808	2.1078457	0	NA	HBM	HBM
HCG11	-2.6850125	1.46858808	0	NA	NA	HCG11
HEATR3	-3.6981394	1.7535573	0	NA	HEATR3	HEATR3
HEMGN	-3.7746287	2.04436448	0	NA	NA	HEMGN
HLA-DMA	4.852325	0.47355534	0	NA	NA	HLA-DMA
HMBOX1	9.4320641	0.24538978	0	NA	NA	HMBOX1
HNRNPA0	-2.8345414	1.2364776	0	NA	NA	HNRNPA0
HSPA2	-3.2032388	1.45747749	0	NA	NA	HSPA2
HTR2A	-8.5978236	3.18105929	0	NA	NA	HTR2A
IMMP1L	-3.1167623	1.36790945	0	NA	NA	IMMP1L
ITGB3BP	3.7077098	0.66980147	0	NA	NA	ITGB3BP

ITPRIP	-4.8085233	1.38297881	0	NA	NA	ITPRIP
KGFLP2	-10.258703	2.72238842	0	NA	NA	KGFLP2
KIF3A	-3.0737407	1.52727468	0	NA	NA	KIF3A
KLHDC8A	4.5112655	0.68928407	0	NA	NA	KLHDC8A
KPNB1	-2.7311651	1.28255063	0	NA	NA	KPNB1
LBH	-3.1891558	1.3292535	0	NA	LBH	LBH
LCLAT1	-4.0539244	1.40814788	0	NA	NA	LCLAT1
LMNB1	-3.4886148	1.61615901	0	NA	LMNB1	LMNB1
LMO7	-2.8636254	1.393642	0	NA	NA	LMO7
LOC100129387	-3.3914122	1.61354421	0	NA	NA	LOC10012938 7
LOC144486	-2.7714156	1.27120705	0	NA	NA	LOC144486
LOC221710	-2.9769436	1.70109375	0	NA	NA	LOC221710
LOC728411	3.7763355	0.60805245	0	NA	LOC728411	LOC728411
LOC728554	3.1656053	0.78126995	0	NA	NA	LOC728554
LRCH1	-2.9817491	1.48754686	0	NA	NA	LRCH1
LRP1	3.4631467	0.73756441	0	NA	LRP1	LRP1
LRRC37A4	-3.3698976	1.68280214	0	NA	NA	LRRC37A4
LSM11	-3.2322088	1.71262628	0	NA	NA	LSM11
LSM2	-3.2913556	1.47098338	0	NA	NA	LSM2
LTF	2.9664019	0.72724447	0	NA	NA	LTF
LUM	-3.2260897	1.95091755	0	NA	NA	LUM
MAMDC2	-3.215659	1.43587342	0	NA	NA	MAMDC2
MAP9	-5.3005398	1.91553803	0	NA	MAP9	MAP9
MCM4	-2.715579	1.29650881	0	NA	MCM4	MCM4
MDM4	3.0196712	0.78043155	0	NA	NA	MDM4
MED6	-3.3017157	1.47041287	0	NA	NA	MED6
METT10D	-2.7754442	1.3206454	0	NA	NA	METT10D
METTL10	3.7397373	0.69287372	0	NA	NA	METTL10
MFN1	-4.2921499	1.39514778	0	NA	NA	MFN1
MLH3	-3.3077975	1.27531588	0	NA	NA	MLH3
MRPS10	-2.8214155	1.36885454	0	NA	NA	MRPS10
MRPS16	-3.3590271	1.35298982	0	NA	NA	MRPS16
MYOCD	-3.082473	1.36241313	0	NA	NA	MYOCD
NAV2	-4.3314651	1.32226567	0	NA	NA	NAV2
NHSL1	-3.5147792	1.30160174	0	NA	NA	NHSL1
NID2	-3.5619483	1.75737721	0	NA	NA	NID2
NOV	-2.847625	1.70505348	0	NA	NA	NOV
OCLN	-3.4971851	1.51101582	0	NA	NA	OCLN
ODF1	4.6347296	0.55370102	0	NA	NA	ODF1
OLFML2B	-2.7124295	1.40929775	0	NA	OLFML2B	OLFML2B
PCDH11Y	3.3331052	0.66928933	0	NA	PCDH11Y	PCDH11Y
PCDHA10	-2.865924	1.36392247	0	NA	NA	PCDHA10
PCDHB2	-3.4170174	1.79354433	0	NA	PCDHB2	PCDHB2
PCSK5	-3.0316199	1.39730482	0	NA	NA	PCSK5
PDGFC	-4.4359191	1.58063729	0	NA	NA	PDGFC
PDGFRL	-3.5086878	2.13452466	0	NA	NA	PDGFRL
PEX13	-4.0700533	1.51194967	0	NA	PEX13	PEX13
PEX7	-2.8320448	1.4155305	0	NA	NA	PEX7
PFDN5	-4.0513292	1.36869283	0	NA	NA	PFDN5
PGCP	-2.6868551	1.3440617	0	NA	NA	PGCP
POLR1A	-3.0997891	1.34305056	0	NA	NA	POLR1A
POLR3E	-3.0437648	1.34474236	0	NA	NA	POLR3E
POMZP3	10.36362	0.3317839	0	NA	NA	POMZP3
PPIG	-3.1038205	1.48329993	0	NA	NA	PPIG
PROCR	-3.6552633	1.42352553	0	NA	NA	PROCR
PRSS35	-2.7653919	1.76424926	0	NA	NA	PRSS35
PSMB5	-3.5088104	1.32463521	0	NA	NA	PSMB5

PTN	-3.5390284	1.42543178	0	NA	NA	PTN
PTPRS	-3.3876319	1.37662535	0	NA	NA	PTPRS
RCL1	-3.8249932	1.39883248	0	NA	NA	RCL1
RCSD1	-2.889604	1.52154846	0	NA	NA	RCSD1
RHOBTB2	3.0460716	0.75270106	0	NA	NA	RHOBTB2
RPL14	-3.225496	1.3377925	0	NA	NA	RPL14
RPL27A	-4.8315189	1.57672313	0	NA	NA	RPL27A
RPL38	-3.7755266	1.3614783	0	NA	NA	RPL38
RPS23	5.6691597	0.65934831	0	NA	NA	RPS23
RPS4Y1	34.031146	0.01044102	0	NA	NA	RPS4Y1
RWDD2A	-2.843736	1.3185053	0	NA	NA	RWDD2A
SAR1A	-11.355276	3.05639008	0	NA	NA	SAR1A
SDC3	3.9075778	0.68806994	0	NA	NA	SDC3
SERINC3	-3.5661646	1.45310724	0	NA	NA	SERINC3
SERPINH1	-3.5150056	1.42815054	0	NA	NA	SERPINH1
SETD6	-3.1832995	1.57052209	0	NA	NA	SETD6
SLC20A2	-2.8595998	1.4429525	0	NA	NA	SLC20A2
SLC41A2	-3.4222912	1.39333258	0	NA	NA	SLC41A2
SLC44A1	-3.7981917	1.47477665	0	NA	SLC44A1	SLC44A1
SNAP29	-6.3300364	1.64923889	0	NA	NA	SNAP29
SOX21	3.5374339	0.65163046	0	NA	SOX21	SOX21
SPARC	-3.0910308	1.34124545	0	NA	NA	SPARC
SPATA5	-3.2914178	1.4958473	0	NA	NA	SPATA5
SVIL	-2.9796019	1.44575569	0	NA	SVIL	SVIL
TBX15	-3.2246332	1.53301564	0	NA	NA	TBX15
TERF1	-3.228146	1.340048	0	NA	NA	TERF1
TMEM100	-3.0007249	1.60591256	0	NA	NA	TMEM100
TMEM106C	-2.9400017	1.50954861	0	NA	NA	TMEM106C
TMEM159	-5.3443917	2.63754195	0	NA	NA	TMEM159
TMEM169	-2.7655447	1.25706554	0	NA	NA	TMEM169
TMEM178	-2.6904621	1.39217609	0	NA	NA	TMEM178
TMEM33	-2.6658069	1.35762943	0	NA	NA	TMEM33
TNNI3K	4.7634285	0.57583172	0	NA	NA	TNNI3K
TTC32	-6.837454	1.87150878	0	NA	NA	TTC32
TWSG1	-2.8596631	1.33741803	0	NA	NA	TWSG1
TXN2	-3.1741458	1.30516754	0	NA	NA	TXN2
UBE2H	-2.9120245	1.29370931	0	NA	NA	UBE2H
ULBP2	3.235594	0.65035909	0	NA	NA	ULBP2
UPF3A	-2.8879641	1.4299963	0	NA	NA	UPF3A
USP7	-3.4224056	1.48698682	0	NA	NA	USP7
USP9Y	24.930165	0.03392756	0	NA	NA	USP9Y
VAMP3	-3.8246818	1.30583534	0	NA	VAMP3	VAMP3
VGLL4	3.9162699	0.49989022	0	NA	NA	VGLL4
WDR86	3.2488551	0.69935269	0	NA	WDR86	WDR86
WNK3	-3.2041129	1.50150119	0	NA	WNK3	WNK3
ZFPM2	-4.5503508	1.52778901	0	NA	NA	ZFPM2
ZFX	-2.982037	1.46067025	0	NA	ZFX	ZFX
ZNF217	-3.8102481	1.65942746	0	NA	ZNF217	ZNF217
ZNF281	-3.4402922	1.32854507	0	NA	NA	ZNF281
ZNF33B	3.2726387	0.53745074	0	NA	NA	ZNF33B
ZNF410	-2.9582169	1.30315211	0	NA	NA	ZNF410
ZNF673	-2.8295142	1.35068033	0	NA	NA	ZNF673
ZNF678	-7.0487526	3.15095782	0	NA	NA	ZNF678
ZNHIT6	-2.8517864	1.32338501	0	NA	NA	ZNHIT6
NANOS1	3.0195092	0.76802038	0	NANOS1	NA	NANOS1
PITPNM3	3.1659088	0.71006956	0	PITPNM3	NA	PITPNM3
SDPR	-3.040839	1.4477601	0	SDPR	SDPR	SDPR
SRR	-4.3326417	1.52243837	0	SRR	NA	SRR

SSR1	-3.1579583	1.33434048	0	SSR1	NA	SSR1
STAG3L4	-5.9270751	1.80174807	0	STAG3L4	NA	STAG3L4
TBC1D15	-2.7400071	1.3902345	0	TBC1D15	NA	TBC1D15
TTTY15	16.499994	0.04287293	0	TTTY15	TTTY15	TTTY15
VIP	3.1452416	0.72815259	0	VIP	NA	VIP
APCDD1	-2.375738	1.25099685	0.15408684	APCDD1	NA	APCDD1
BPGM	-2.4073412	1.32814895	0.15408684	BPGM	NA	BPGM
C9orf72	-2.3188655	1.25802647	0.15408684	C9orf72	NA	C9orf72
DAZ4	2.859064	0.57365678	0.15408684	DAZ4	NA	DAZ4
EDNRA	-2.5143927	1.50640502	0.15408684	EDNRA	EDNRA	EDNRA
ITIH5	-2.3664665	1.25547418	0.15408684	ITIH5	ITIH5	ITIH5
LOC400657	-2.6144841	1.26930781	0.15408684	LOC400657	NA	LOC400657
MAP3K7	-2.3125599	1.23669912	0.15408684	MAP3K7	MAP3K7	MAP3K7
AACS	2.7772252	0.81928872	0.15408684	NA	NA	AACS
AASDH	-2.2837131	1.33839499	0.15408684	NA	NA	AASDH
AASS	-2.3766554	1.24422141	0.15408684	NA	NA	AASS
ADAMTS5	-2.5578755	1.32124927	0.15408684	NA	NA	ADAMTS5
ADORA2B	-2.6464446	1.52409042	0.15408684	NA	NA	ADORA2B
AKAP12	-2.2786081	1.22442061	0.15408684	NA	AKAP12	AKAP12
AKAP5	-2.4516203	1.43557392	0.15408684	NA	NA	AKAP5
ALKBH3	-2.4735768	1.26430568	0.15408684	NA	NA	ALKBH3
ANGPT2	-2.3641777	1.44208847	0.15408684	NA	NA	ANGPT2
ANKRD13A	-2.609523	1.27601125	0.15408684	NA	NA	ANKRD13A
ARFGEF2	-2.3904299	1.20561488	0.15408684	NA	NA	ARFGEF2
BMPR1B	2.6068905	0.57627958	0.15408684	NA	BMPR1B	BMPR1B
BPNT1	-2.6408925	1.2100756	0.15408684	NA	NA	BPNT1
BUB3	-2.4370156	1.20337506	0.15408684	NA	NA	BUB3
C10orf78	-2.3176742	1.3305325	0.15408684	NA	NA	C10orf78
C14orf118	-2.3531121	1.33016407	0.15408684	NA	NA	C14orf118
C21orf119	2.844505	0.67810524	0.15408684	NA	NA	C21orf119
C6orf225	-2.4203083	1.2979177	0.15408684	NA	C6orf225	C6orf225
C6orf52	2.7647041	0.73088546	0.15408684	NA	NA	C6orf52
CASC3	-2.6647051	1.30076511	0.15408684	NA	NA	CASC3
CAST	-2.4073902	1.23782379	0.15408684	NA	NA	CAST
CBX5	-2.3490675	1.38954567	0.15408684	NA	NA	CBX5
CCL2	-2.4253942	1.60984817	0.15408684	NA	CCL2	CCL2
CCNT1	-2.4365363	1.58989758	0.15408684	NA	NA	CCNT1
CD302	-2.5345817	1.43298482	0.15408684	NA	NA	CD302
CLSTN2	-2.4636842	1.6788568	0.15408684	NA	NA	CLSTN2
CNIH4	-2.4647397	1.24346588	0.15408684	NA	NA	CNIH4
COL11A1	-2.4263028	1.58502373	0.15408684	NA	NA	COL11A1
COL1A2	-2.5247003	1.53757227	0.15408684	NA	NA	COL1A2
COL21A1	-2.2715263	1.39844564	0.15408684	NA	NA	COL21A1
CPEB3	-2.3418384	1.29947995	0.15408684	NA	NA	CPEB3
CRB1	2.7218365	0.77834041	0.15408684	NA	NA	CRB1
CSRNP3	-2.6324874	1.77680543	0.15408684	NA	NA	CSRNP3
CTNNB1	-2.3310371	1.55618518	0.15408684	NA	CTNNB1	CTNNB1
CTSL1	-2.5466408	1.46599936	0.15408684	NA	NA	CTSL1
CUL2	2.8363592	0.7865667	0.15408684	NA	CUL2	CUL2
CYP1B1	-2.5728104	1.59423958	0.15408684	NA	NA	CYP1B1
DAPK1	2.8189297	0.76157288	0.15408684	NA	NA	DAPK1
DGAT2	2.9155871	0.76247906	0.15408684	NA	NA	DGAT2
DGKA	2.6749752	0.76261627	0.15408684	NA	NA	DGKA
DHRS11	-2.3061322	1.45353867	0.15408684	NA	NA	DHRS11
DPY19L2	-2.3058242	1.26838212	0.15408684	NA	DPY19L2	DPY19L2
EFEMP1	-2.2601246	1.512079	0.15408684	NA	NA	EFEMP1
EIF1AX	-2.2607281	1.24915554	0.15408684	NA	EIF1AX	EIF1AX
EMCN	-2.4724299	1.31793197	0.15408684	NA	EMCN	EMCN

ENDOD1	-2.6652223	1.23841843	0.15408684	NA	NA	ENDOD1
ENTPD4	-2.2639859	1.26243617	0.15408684	NA	NA	ENTPD4
EPB41L1	-2.5552412	1.24069766	0.15408684	NA	EPB41L1	EPB41L1
EPC1	-2.5084281	1.27380607	0.15408684	NA	NA	EPC1
ERCC8	-2.3758432	1.20989691	0.15408684	NA	NA	ERCC8
ESCO1	-2.5615491	1.28333929	0.15408684	NA	ESCO1	ESCO1
EXOSC2	-2.507613	1.19153459	0.15408684	NA	NA	EXOSC2
FASTKD2	-2.439101	1.25346273	0.15408684	NA	FASTKD2	FASTKD2
FBXW10	2.5525607	0.7559772	0.15408684	NA	NA	FBXW10
GABRB1	2.6685844	0.59317539	0.15408684	NA	NA	GABRB1
GALNT1	-2.5505422	1.2497617	0.15408684	NA	NA	GALNT1
GLRB	-2.5792542	1.28960532	0.15408684	NA	GLRB	GLRB
GSK3B	-2.3125855	1.8209692	0.15408684	NA	NA	GSK3B
HCCS	-2.2828589	1.20536856	0.15408684	NA	HCCS	HCCS
HDDC2	2.513864	0.71840442	0.15408684	NA	HDDC2	HDDC2
HHAT	-2.6642639	1.37203725	0.15408684	NA	NA	HHAT
HHLA3	2.5531852	0.84325886	0.15408684	NA	NA	HHLA3
HYI	-2.5821409	1.26010427	0.15408684	NA	HYI	HYI
ITIH2	-2.5172604	1.57287272	0.15408684	NA	NA	ITIH2
ITPRIPL2	-2.2714985	1.18882915	0.15408684	NA	NA	ITPRIPL2
KATNA1	-2.4099145	1.20178113	0.15408684	NA	NA	KATNA1
KCNG2	-2.3943031	1.31400163	0.15408684	NA	NA	KCNG2
KHDRBS2	-2.310516	1.28131427	0.15408684	NA	KHDRBS2	KHDRBS2
KIAA1033	-2.5258399	1.4238757	0.15408684	NA	NA	KIAA1033
KIF21A	-2.5343021	1.31543011	0.15408684	NA	NA	KIF21A
KLHL5	-2.5304512	1.28779043	0.15408684	NA	NA	KLHL5
KRT75	2.5310171	0.83537088	0.15408684	NA	NA	KRT75
LDHAL6A	2.7683764	0.7212885	0.15408684	NA	NA	LDHAL6A
LOC253039	-2.2687965	1.31648364	0.15408684	NA	NA	LOC253039
LOC338758	-2.632777	1.41919053	0.15408684	NA	LOC338758	LOC338758
LOC344595	-2.3850471	1.28620698	0.15408684	NA	NA	LOC344595
LOC723809	2.8695723	0.71704125	0.15408684	NA	LOC723809	LOC723809
LYVE1	-2.2907557	1.9875599	0.15408684	NA	NA	LYVE1
MALAT1	-2.6059227	1.84262573	0.15408684	NA	NA	MALAT1
MAML3	-2.2695581	1.18278914	0.15408684	NA	NA	MAML3
MAN1A1	-2.3485979	1.47403349	0.15408684	NA	NA	MAN1A1
MARS2	-2.5238092	1.38801805	0.15408684	NA	NA	MARS2
MED1	-2.6381891	1.36884522	0.15408684	NA	NA	MED1
MMGT1	-2.3779571	1.23820151	0.15408684	NA	NA	MMGT1
MRC2	-2.6443084	1.27734229	0.15408684	NA	NA	MRC2
NCAPG	-2.3366229	1.38654065	0.15408684	NA	NA	NCAPG
NEK10	-2.2733071	1.3437932	0.15408684	NA	NA	NEK10
NEK11	2.6425471	0.83268837	0.15408684	NA	NA	NEK11
NKAIN2	-2.4988171	1.40672174	0.15408684	NA	NKAIN2	NKAIN2
OLFML3	-2.5775999	1.68013647	0.15408684	NA	NA	OLFML3
PBLD	-2.3296979	1.38976503	0.15408684	NA	NA	PBLD
PEX3	-2.3367843	1.24747703	0.15408684	NA	NA	PEX3
PHAX	-2.6002356	1.26474595	0.15408684	NA	NA	PHAX
PHB	-2.3630554	1.18715953	0.15408684	NA	PHB	PHB
PHF2	2.7253249	0.72802401	0.15408684	NA	PHF2	PHF2
PIGF	-2.5599996	1.22772039	0.15408684	NA	NA	PIGF
PITRM1	2.7774431	0.78930237	0.15408684	NA	NA	PITRM1
PNOC	2.5737188	0.75599796	0.15408684	NA	NA	PNOC
PNPLA8	-2.3946008	1.24409588	0.15408684	NA	NA	PNPLA8
PPIB	-2.3549659	1.28657741	0.15408684	NA	NA	PPIB
PRMT8	-2.3041096	1.2286679	0.15408684	NA	NA	PRMT8
PRPF39	-2.3193488	1.47045554	0.15408684	NA	NA	PRPF39
PRPH2	2.8264552	0.70562354	0.15408684	NA	PRPH2	PRPH2

PTCD3	-2.3290937	1.20928246	0.15408684	NA	NA	PTCD3
RASA2	-2.3064891	1.37965524	0.15408684	NA	NA	RASA2
RBMS3	-2.5382434	1.17434759	0.15408684	NA	NA	RBMS3
RP9	-2.5188738	1.24645567	0.15408684	NA	RP9	RP9
RPGRIP1L	-2.2612711	1.2759607	0.15408684	NA	NA	RPGRIP1L
RPP25	2.9209582	0.75889774	0.15408684	NA	NA	RPP25
SCARNA2	2.7205174	0.69920881	0.15408684	NA	NA	SCARNA2
SEC31A	-2.4401949	1.27613832	0.15408684	NA	NA	SEC31A
SENP8	-2.3818281	1.17602863	0.15408684	NA	NA	SENP8
SFRS12IP1	-2.3118187	1.28184605	0.15408684	NA	NA	SFRS12IP1
SIAH3	-2.5901526	1.8345651	0.15408684	NA	NA	SIAH3
SLC35B3	-2.2959573	1.31271787	0.15408684	NA	NA	SLC35B3
SMURF2	-2.263931	1.37802813	0.15408684	NA	NA	SMURF2
SOAT2	2.5770608	0.73759794	0.15408684	NA	NA	SOAT2
SRL	-2.5935564	1.28325817	0.15408684	NA	NA	SRL
SSR3	-2.5990322	1.35165952	0.15408684	NA	SSR3	SSR3
SYPL2	2.7515259	0.65328867	0.15408684	NA	SYPL2	SYPL2
TAOK1	-2.4294969	1.56100579	0.15408684	NA	NA	TAOK1
TCL1A	2.7836999	0.79808427	0.15408684	NA	NA	TCL1A
TM7SF4	2.6204674	0.77172555	0.15408684	NA	TM7SF4	TM7SF4
TRAPPC5	-2.4614767	1.30937375	0.15408684	NA	NA	TRAPPC5
TRIM69	-2.3625574	1.46343493	0.15408684	NA	NA	TRIM69
TUBB6	-2.3299468	1.45598407	0.15408684	NA	NA	TUBB6
UHRF1	-2.5674501	1.3798915	0.15408684	NA	NA	UHRF1
VDAC3	-2.4883873	1.21200174	0.15408684	NA	NA	VDAC3
VPS29	-2.4892411	1.22026069	0.15408684	NA	NA	VPS29
WDFY1	-2.3148448	1.31487986	0.15408684	NA	NA	WDFY1
ZCCHC6	-2.4770813	1.23305412	0.15408684	NA	NA	ZCCHC6
ZEB1	-2.4675301	1.41869147	0.15408684	NA	NA	ZEB1
ZIC2	-2.5628214	1.55072803	0.15408684	NA	NA	ZIC2
ZNF2	-2.3970363	1.41933831	0.15408684	NA	NA	ZNF2
ZNF326	-2.3209629	1.23916508	0.15408684	NA	ZNF326	ZNF326
ZNF568	-2.5273151	1.28010059	0.15408684	NA	NA	ZNF568
ZNF644	-2.5407901	1.55442586	0.15408684	NA	ZNF644	ZNF644
ZNF646	-2.3111948	1.33040038	0.15408684	NA	NA	ZNF646
ZNF827	-2.4277283	1.4249109	0.15408684	NA	NA	ZNF827
NFU1	2.5576059	0.78910103	0.15408684	NFU1	NFU1	NFU1
NKAIN3	2.9072777	0.80136794	0.15408684	NKAIN3	NKAIN3	NKAIN3
PKN2	-2.5086486	1.30100005	0.15408684	PKN2	PKN2	PKN2
PRKAB2	-2.4229614	1.32794586	0.15408684	PRKAB2	NA	PRKAB2
PSAT1	-2.3191814	1.22755029	0.15408684	PSAT1	PSAT1	PSAT1
TOP1	-2.5863571	1.42133876	0.15408684	TOP1	NA	TOP1
WSB1	-2.3133712	1.38965777	0.15408684	WSB1	WSB1	WSB1
ADPGK	-2.0753836	1.21126407	0.52040198	ADPGK	NA	ADPGK
ALDH6A1	-1.9990247	1.22934956	0.52040198	ALDH6A1	NA	ALDH6A1
ATOH1	2.1058924	0.72609592	0.52040198	ATOH1	ATOH1	ATOH1
CA9	2.0772529	0.71449784	0.52040198	CA9	NA	CA9
EFCAB4B	2.4987579	0.8501656	0.52040198	EFCAB4B	EFCAB4B	EFCAB4B
FAM96A	-1.9027157	1.18994558	0.52040198	FAM96A	FAM96A	FAM96A
FAT1	-2.1651627	1.28255083	0.52040198	FAT1	FAT1	FAT1
FLJ46321	2.44914	0.76711204	0.52040198	FLJ46321	NA	FLJ46321
FRMD6	-2.2175814	1.30253868	0.52040198	FRMD6	FRMD6	FRMD6
IFLTD1	2.4490002	0.75215324	0.52040198	IFLTD1	NA	IFLTD1
KCTD12	-1.9045931	1.28329693	0.52040198	KCTD12	KCTD12	KCTD12
KDR	-2.1881049	1.32116515	0.52040198	KDR	KDR	KDR
LOC541471	-2.1699253	1.20290734	0.52040198	LOC541471	NA	LOC541471
MGST2	-2.1789181	1.41626241	0.52040198	MGST2	NA	MGST2
ABCA17P	2.3343513	0.73624021	0.52040198	NA	ABCA17P	ABCA17P

ABCC8	2.4328791	0.76046742	0.52040198	NA	NA	ABCC8
ADK	-2.1302733	1.31924955	0.52040198	NA	NA	ADK
AHI1	-1.9220662	1.18209537	0.52040198	NA	NA	AHI1
ANKRD42	2.2749257	0.82264758	0.52040198	NA	NA	ANKRD42
ANP32A	-2.0033225	1.22966807	0.52040198	NA	NA	ANP32A
ANXA2	-1.9997924	1.45329543	0.52040198	NA	NA	ANXA2
APOD	2.2826264	0.73716617	0.52040198	NA	NA	APOD
APOL1	-1.9121602	1.38224102	0.52040198	NA	APOL1	APOL1
ARFIP1	-2.1744556	1.30306661	0.52040198	NA	NA	ARFIP1
ARID2	-2.1601992	1.33696186	0.52040198	NA	NA	ARID2
ATPAF2	-2.0593733	1.23084647	0.52040198	NA	NA	ATPAF2
BAG3	-1.9384694	1.31468397	0.52040198	NA	NA	BAG3
BCAS1	-2.084448	1.20803661	0.52040198	NA	NA	BCAS1
BFSP2	2.1901177	0.82295316	0.52040198	NA	NA	BFSP2
BMP2K	-1.9532441	1.16821622	0.52040198	NA	NA	BMP2K
BMP8A	2.1058632	0.73990316	0.52040198	NA	NA	BMP8A
BOK	2.3721644	0.76793487	0.52040198	NA	NA	BOK
BRAP	-2.1352878	1.2534199	0.52040198	NA	NA	BRAP
C11orf57	-2.0509347	1.18894566	0.52040198	NA	NA	C11orf57
C14orf178	2.1092287	0.78711472	0.52040198	NA	C14orf178	C14orf178
C15orf61	-1.9751662	1.23347746	0.52040198	NA	NA	C15orf61
C17orf86	-2.0339511	1.26658073	0.52040198	NA	NA	C17orf86
C1orf27	-1.974642	1.30984575	0.52040198	NA	NA	C1orf27
C21orf49	2.4976927	0.79183242	0.52040198	NA	NA	C21orf49
C2orf42	-1.8927023	1.20108286	0.52040198	NA	NA	C2orf42
C3orf17	-1.9128026	1.21356318	0.52040198	NA	C3orf17	C3orf17
C3orf57	-1.8893237	1.19816256	0.52040198	NA	NA	C3orf57
C7orf51	-2.1071945	1.33640561	0.52040198	NA	NA	C7orf51
CACNG2	2.4539067	0.79018397	0.52040198	NA	NA	CACNG2
CAMK2N1	-2.068048	1.26749433	0.52040198	NA	NA	CAMK2N1
CAPN8	2.062243	0.73588244	0.52040198	NA	NA	CAPN8
CC2D2B	2.3528731	0.65284613	0.52040198	NA	NA	CC2D2B
CCDC125	-2.1515828	1.23519787	0.52040198	NA	NA	CCDC125
CCDC140	2.1053809	0.80331706	0.52040198	NA	NA	CCDC140
CCDC28A	-2.1309696	1.26417516	0.52040198	NA	NA	CCDC28A
CD47	-1.9775249	1.21294482	0.52040198	NA	CD47	CD47
CDC42	2.1906733	0.81494459	0.52040198	NA	CDC42	CDC42
CECR1	2.2627415	0.76025114	0.52040198	NA	NA	CECR1
CEP110	-2.1125622	1.19484619	0.52040198	NA	NA	CEP110
CHEK2	2.114179	0.78587628	0.52040198	NA	NA	CHEK2
CLDN16	2.3310751	0.79347685	0.52040198	NA	NA	CLDN16
CLN5	-1.9063071	1.27256393	0.52040198	NA	CLN5	CLN5
COL4A1	-2.0671428	1.22169476	0.52040198	NA	COL4A1	COL4A1
COL9A2	2.1659016	0.70015242	0.52040198	NA	NA	COL9A2
COX15	-1.9051802	1.2085638	0.52040198	NA	NA	COX15
CPXM2	2.1325825	0.86026774	0.52040198	NA	NA	CPXM2
CST7	2.3033608	0.78005281	0.52040198	NA	NA	CST7
CTSC	-2.1849962	1.37608816	0.52040198	NA	NA	CTSC
CTTNBP2	-2.1819356	1.25458024	0.52040198	NA	NA	CTTNBP2
CXorf38	-2.0975988	1.39567315	0.52040198	NA	NA	CXorf38
CXorf59	2.0504571	0.78277683	0.52040198	NA	NA	CXorf59
DCST1	2.1424342	0.71034187	0.52040198	NA	NA	DCST1
DEGS1	-2.072779	1.1858773	0.52040198	NA	NA	DEGS1
DEXI	2.1112593	0.83873045	0.52040198	NA	DEXI	DEXI
DHTKD1	-2.0802506	1.22301036	0.52040198	NA	NA	DHTKD1
DNAH7	2.41911	0.70773173	0.52040198	NA	NA	DNAH7
DNAJC5G	2.0911647	0.71262761	0.52040198	NA	NA	DNAJC5G
EFHB	-1.9809965	1.37724656	0.52040198	NA	NA	EFHB

EIF2C3	-2.0435392	1.34298354	0.52040198	NA	NA	EIF2C3
EIF2S3	-1.9601947	1.16509672	0.52040198	NA	EIF2S3	EIF2S3
EIF3G	-2.2129915	1.18242727	0.52040198	NA	NA	EIF3G
EIF5B	-2.0811152	1.23309634	0.52040198	NA	NA	EIF5B
EMILIN3	2.2222533	0.73846615	0.52040198	NA	NA	EMILIN3
ERAP2	-2.1155597	1.16759146	0.52040198	NA	NA	ERAP2
ERP44	-2.024124	1.18241014	0.52040198	NA	NA	ERP44
FAM122A	-1.9726457	1.17223446	0.52040198	NA	NA	FAM122A
FAM131C	2.1503249	0.82193538	0.52040198	NA	NA	FAM131C
FAM150B	2.0544744	0.70944555	0.52040198	NA	NA	FAM150B
FAM186B	2.1947687	0.78534665	0.52040198	NA	NA	FAM186B
FAM35A	-2.1911292	1.24569496	0.52040198	NA	NA	FAM35A
FARSB	-2.0363338	1.38382939	0.52040198	NA	NA	FARSB
FBN1	-2.2223297	1.35000795	0.52040198	NA	NA	FBN1
FER	-2.0464847	1.36356216	0.52040198	NA	NA	FER
FGL2	-2.0653139	1.59204968	0.52040198	NA	NA	FGL2
FLJ39653	-2.0964844	1.20766404	0.52040198	NA	FLJ39653	FLJ39653
GABRB2	2.371876	0.68809537	0.52040198	NA	NA	GABRB2
GAR1	-2.1861567	1.23255098	0.52040198	NA	NA	GAR1
GATAD1	-2.0920612	1.22377274	0.52040198	NA	NA	GATAD1
GEMIN8	-2.1429039	1.22834499	0.52040198	NA	NA	GEMIN8
GHRHR	2.0826463	0.74904975	0.52040198	NA	NA	GHRHR
GIMAP2	-2.0266119	1.33596833	0.52040198	NA	NA	GIMAP2
GLT8D2	-1.8940871	1.25220948	0.52040198	NA	NA	GLT8D2
GLTP	-1.9432979	1.25181329	0.52040198	NA	NA	GLTP
GNAL	-2.0080944	1.86789974	0.52040198	NA	NA	GNAL
GNG10	-2.0686584	1.27356848	0.52040198	NA	NA	GNG10
GNPDA1	-2.0278047	1.20704513	0.52040198	NA	NA	GNPDA1
GNPNAT1	-2.125466	1.17206348	0.52040198	NA	NA	GNPNAT1
GPC1	2.1876605	0.78278178	0.52040198	NA	NA	GPC1
GPR83	-1.9193755	1.41579111	0.52040198	NA	NA	GPR83
GPR89B	-2.0351575	1.17692447	0.52040198	NA	NA	GPR89B
GRB7	2.1377094	0.7432128	0.52040198	NA	NA	GRB7
GSTO2	2.2568222	0.69059817	0.52040198	NA	NA	GSTO2
GTF2H1	-1.8994516	1.16105061	0.52040198	NA	NA	GTF2H1
HBP1	-1.9735302	1.26538923	0.52040198	NA	NA	HBP1
HEATR5A	-1.902767	1.34983554	0.52040198	NA	NA	HEATR5A
HIBADH	-1.9814384	1.21161046	0.52040198	NA	NA	HIBADH
HINT3	-1.9441384	1.60393052	0.52040198	NA	NA	HINT3
HMGCR	2.0532203	0.86415929	0.52040198	NA	NA	HMGCR
HS3ST4	-1.889094	1.23721883	0.52040198	NA	HS3ST4	HS3ST4
HSD11B2	2.2925167	0.72482175	0.52040198	NA	NA	HSD11B2
HSDL2	-1.8965978	1.29385895	0.52040198	NA	HSDL2	HSDL2
HTATIP2	2.0700273	0.79864545	0.52040198	NA	NA	HTATIP2
ICOS	2.4927746	0.75229582	0.52040198	NA	ICOS	ICOS
IDH2	-2.0983052	1.21860617	0.52040198	NA	IDH2	IDH2
INTS9	-2.0402097	1.16961044	0.52040198	NA	NA	INTS9
ISLR2	-1.9758955	1.33074964	0.52040198	NA	NA	ISLR2
ISOC1	2.4713551	0.77933133	0.52040198	NA	NA	ISOC1
KCTD16	2.354395	0.60252427	0.52040198	NA	NA	KCTD16
KIAA0090	-2.1808647	1.23180295	0.52040198	NA	NA	KIAA0090
KIAA0831	-2.1327187	1.19270868	0.52040198	NA	KIAA0831	KIAA0831
KIAA1586	-2.1571613	1.28117498	0.52040198	NA	NA	KIAA1586
KIF5A	-2.0342757	1.35590469	0.52040198	NA	NA	KIF5A
KRT74	2.1239248	0.77422916	0.52040198	NA	NA	KRT74
KRT82	2.4586007	0.73755047	0.52040198	NA	NA	KRT82
KTELC1	-2.1984993	1.29330049	0.52040198	NA	NA	KTELC1
LAPTM4B	-1.9216933	1.34113928	0.52040198	NA	NA	LAPTM4B

LHB	-2.0833171	1.28891523	0.52040198	NA	NA	LHB
LLPH	-1.999817	1.1937156	0.52040198	NA	NA	LLPH
LMX1A	2.4550773	0.77172307	0.52040198	NA	NA	LMX1A
LOC100192378	2.4134955	0.79027925	0.52040198	NA	LOC100192378	LOC100192378
LOC144438	-2.0132641	1.26333967	0.52040198	NA	NA	LOC144438
LOC153328	2.4023878	0.77768044	0.52040198	NA	NA	LOC153328
LOC283174	-2.1237479	1.27073276	0.52040198	NA	NA	LOC283174
LOC340357	2.3057571	0.73874396	0.52040198	NA	NA	LOC340357
LOC374491	-1.9806138	1.2798857	0.52040198	NA	NA	LOC374491
LRRC17	-1.9927652	1.27770681	0.52040198	NA	NA	LRRC17
LRRC37A2	2.1760499	0.82621777	0.52040198	NA	NA	LRRC37A2
LRRC57	-2.0589527	1.30361128	0.52040198	NA	NA	LRRC57
LSG1	-1.8904563	1.20921704	0.52040198	NA	NA	LSG1
MAP3K12	2.1973903	0.81938356	0.52040198	NA	NA	MAP3K12
MAPK3	2.0914245	0.85292565	0.52040198	NA	NA	MAPK3
MAS1	2.0962005	0.72382428	0.52040198	NA	NA	MAS1
MB	2.27274	0.71881396	0.52040198	NA	NA	MB
MCC	-2.1187613	1.29070636	0.52040198	NA	NA	MCC
MCPH1	-2.1621202	1.1819172	0.52040198	NA	MCPH1	MCPH1
MFSD6L	2.2174655	0.7702194	0.52040198	NA	NA	MFSD6L
MKKS	2.3111227	0.77454001	0.52040198	NA	MKKS	MKKS
MMP16	-1.9949701	1.25948912	0.52040198	NA	NA	MMP16
MPPED1	-2.0119839	1.21751657	0.52040198	NA	MPPED1	MPPED1
MRC1	-2.0059435	2.02300477	0.52040198	NA	NA	MRC1
MRPL2	-1.902676	1.21328046	0.52040198	NA	NA	MRPL2
MSL2	-1.9960172	1.29359364	0.52040198	NA	NA	MSL2
MTERF	-2.1988882	1.20543362	0.52040198	NA	NA	MTERF
MYH10	-1.9686168	1.30885144	0.52040198	NA	NA	MYH10
NCDN	2.4662698	0.81990466	0.52040198	NA	NA	NCDN
NDC80	-2.0028732	1.29395268	0.52040198	NA	NA	NDC80
NDUFS5	2.2017389	0.83524493	0.52040198	NA	NA	NDUFS5
NLN	-2.1851909	1.1969421	0.52040198	NA	NA	NLN
NOSTRIN	-2.1178711	1.29669248	0.52040198	NA	NA	NOSTRIN
NSL1	-2.0645267	1.2104069	0.52040198	NA	NA	NSL1
OMD	-2.2479875	1.87282089	0.52040198	NA	NA	OMD
ORMDL2	-1.9860724	1.19784337	0.52040198	NA	NA	ORMDL2
PAFAH1B2	-1.8806275	1.17625486	0.52040198	NA	PAFAH1B2	PAFAH1B2
PAIP2B	2.1305932	0.79107342	0.52040198	NA	NA	PAIP2B
PAPSS2	-2.2111151	1.2575037	0.52040198	NA	NA	PAPSS2
PCDH11X	2.1516949	0.84514434	0.52040198	NA	PCDH11X	PCDH11X
PCDH8	2.4071771	0.71515048	0.52040198	NA	NA	PCDH8
PCDHGA1	2.307887	0.79195605	0.52040198	NA	NA	PCDHGA1
PCGF6	-1.885943	1.27052744	0.52040198	NA	NA	PCGF6
PCLO	2.1715754	0.80619704	0.52040198	NA	NA	PCLO
PCNXL2	2.0737151	0.79867141	0.52040198	NA	PCNXL2	PCNXL2
PCSK6	2.4986726	0.84049685	0.52040198	NA	NA	PCSK6
PDE1A	-2.2262416	1.28671827	0.52040198	NA	NA	PDE1A
PEX26	-2.1140121	1.21066874	0.52040198	NA	NA	PEX26
PEX5L	-2.1640812	1.24539707	0.52040198	NA	PEX5L	PEX5L
PGM3	-2.0435103	1.26859256	0.52040198	NA	NA	PGM3
PLAG1	-1.9965254	1.30066752	0.52040198	NA	NA	PLAG1
PON1	2.0833775	0.77710618	0.52040198	NA	NA	PON1
POP5	-2.0889906	1.233827	0.52040198	NA	NA	POP5
POU2F2	-1.9716952	1.24277587	0.52040198	NA	NA	POU2F2
PPM1D	-2.141691	1.23000435	0.52040198	NA	NA	PPM1D
PPPDE1	-2.2219879	1.26451279	0.52040198	NA	NA	PPPDE1
PREP	-1.984032	1.275789	0.52040198	NA	PREP	PREP

PRIMA1	2.2221399	0.74154802	0.52040198	NA	NA	PRIMA1
PRRX1	-2.2172145	1.3321327	0.52040198	NA	NA	PRRX1
PSAPL1	2.1745227	0.79341248	0.52040198	NA	NA	PSAPL1
PSD3	2.261628	0.7178862	0.52040198	NA	NA	PSD3
PSIP1	-1.991087	1.24723292	0.52040198	NA	NA	PSIP1
QTRTD1	-1.9218234	1.17645254	0.52040198	NA	NA	QTRTD1
R3HDM2	-1.8986794	1.27911761	0.52040198	NA	NA	R3HDM2
RAI14	-1.9661168	1.3050944	0.52040198	NA	NA	RAI14
RANBP3L	-1.8840796	1.21065335	0.52040198	NA	NA	RANBP3L
RARA	2.487077	0.76487883	0.52040198	NA	RARA	RARA
RBKS	-1.9427115	1.24363587	0.52040198	NA	NA	RBKS
RBM27	-1.8993699	1.21577946	0.52040198	NA	NA	RBM27
RBMS1	-2.1281534	1.34581448	0.52040198	NA	NA	RBMS1
RDX	-2.0118485	1.22780659	0.52040198	NA	NA	RDX
REG3A	2.1035545	0.83289156	0.52040198	NA	NA	REG3A
RGS22	2.1723	0.76006672	0.52040198	NA	NA	RGS22
RLTPR	2.3650698	0.79681502	0.52040198	NA	RLTPR	RLTPR
RNF115	-2.2382884	1.31117258	0.52040198	NA	NA	RNF115
ROBO3	2.3069616	0.74089878	0.52040198	NA	ROBO3	ROBO3
ROD1	-2.0930953	1.32272881	0.52040198	NA	NA	ROD1
RPP14	-2.1327202	1.32992645	0.52040198	NA	NA	RPP14
RPS4X	-2.1992759	1.1435872	0.52040198	NA	NA	RPS4X
RSBN1	-2.1458921	1.19759535	0.52040198	NA	NA	RSBN1
RSF1	-2.0162309	1.31937541	0.52040198	NA	NA	RSF1
RUFY3	-1.9157856	1.19877545	0.52040198	NA	RUFY3	RUFY3
RYK	-2.1729138	1.28586957	0.52040198	NA	NA	RYK
S100A11	-2.1809246	1.37936479	0.52040198	NA	NA	S100A11
SAG	2.3658024	0.75387657	0.52040198	NA	NA	SAG
SAMD9	-1.962769	1.22384576	0.52040198	NA	NA	SAMD9
SCRN3	-2.102817	1.23760316	0.52040198	NA	SCRN3	SCRN3
SDCCAG1	-2.0260314	1.17062402	0.52040198	NA	NA	SDCCAG1
SERPINB8	2.1704807	0.7920756	0.52040198	NA	NA	SERPINB8
SETD1A	-2.0067014	1.23141286	0.52040198	NA	NA	SETD1A
SHFM1	-2.0231735	1.21727741	0.52040198	NA	NA	SHFM1
SHROOM4	-2.1155196	1.19954957	0.52040198	NA	NA	SHROOM4
SIPA1L1	-2.0250012	1.19199125	0.52040198	NA	SIPA1L1	SIPA1L1
SLA	-2.0851739	1.25234599	0.52040198	NA	NA	SLA
SLC12A5	2.2462063	0.81191818	0.52040198	NA	NA	SLC12A5
SLC16A7	-2.1144529	1.4193732	0.52040198	NA	SLC16A7	SLC16A7
SLC25A14	-2.1189443	1.20191495	0.52040198	NA	NA	SLC25A14
SLC29A3	2.3957502	0.77489056	0.52040198	NA	SLC29A3	SLC29A3
SLC31A2	-1.974387	1.32182763	0.52040198	NA	NA	SLC31A2
SLC39A10	2.2087944	0.83672299	0.52040198	NA	NA	SLC39A10
SLC4A7	-2.1984199	1.31180157	0.52040198	NA	NA	SLC4A7
SNORA57	2.2363773	0.75239695	0.52040198	NA	NA	SNORA57
SNRNP27	-2.0354463	1.18932417	0.52040198	NA	NA	SNRNP27
SOSTDC1	2.3612779	0.72786663	0.52040198	NA	NA	SOSTDC1
SPNS3	2.1222548	0.70307546	0.52040198	NA	NA	SPNS3
SS18	-1.9681358	1.22695495	0.52040198	NA	SS18	SS18
STAM	2.279432	0.84229943	0.52040198	NA	NA	STAM
STRN3	-2.1987479	1.27517342	0.52040198	NA	NA	STRN3
SUMF1	-2.1038277	1.21929102	0.52040198	NA	SUMF1	SUMF1
TAF13	-2.0862826	1.39718788	0.52040198	NA	NA	TAF13
TFAM	-2.2124488	1.29583364	0.52040198	NA	NA	TFAM
TFDP3	2.2307515	0.76327553	0.52040198	NA	TFDP3	TFDP3
TFPI2	-1.9400506	1.14045646	0.52040198	NA	NA	TFPI2
TIMP3	-2.0849439	1.2966405	0.52040198	NA	NA	TIMP3
TJP1	-1.9522857	1.19967294	0.52040198	NA	NA	TJP1

TLR2	2.109616	0.7260496	0.52040198	NA	NA	TLR2
TLX3	2.0933695	0.74356216	0.52040198	NA	NA	TLX3
TMCC1	-1.975072	1.16245899	0.52040198	NA	TMCC1	TMCC1
TMEM106B	-2.0505189	1.30611139	0.52040198	NA	TMEM106B	TMEM106B
TMEM184C	-1.9276261	1.22844115	0.52040198	NA	NA	TMEM184C
TMEM220	-1.9798549	1.27831435	0.52040198	NA	NA	TMEM220
TMEM39A	-2.0776092	1.22157921	0.52040198	NA	NA	TMEM39A
TNIK	-1.9259299	1.24052773	0.52040198	NA	NA	TNIK
TOE1	-2.007117	1.23124864	0.52040198	NA	NA	TOE1
TOR1AIP1	-1.9418099	1.27535724	0.52040198	NA	NA	TOR1AIP1
TRPM7	-2.1178918	1.19694633	0.52040198	NA	NA	TRPM7
TSPAN3	-1.9084854	1.12209829	0.52040198	NA	NA	TSPAN3
TTC21A	2.1685433	0.81430899	0.52040198	NA	NA	TTC21A
TTC39B	-2.1480051	1.21699674	0.52040198	NA	TTC39B	TTC39B
TWIST1	-2.1784754	1.38715615	0.52040198	NA	NA	TWIST1
TWISTNB	-1.9593813	1.23898705	0.52040198	NA	NA	TWISTNB
UBE2N	-1.971055	1.15218407	0.52040198	NA	NA	UBE2N
UBQLN2	2.0551659	0.83870165	0.52040198	NA	NA	UBQLN2
UBXN7	-2.1792546	1.33298596	0.52040198	NA	NA	UBXN7
UTP23	-2.0043301	1.44435443	0.52040198	NA	NA	UTP23
VGLL3	-1.8927667	1.23418995	0.52040198	NA	VGLL3	VGLL3
VRK1	-2.0220907	1.19558435	0.52040198	NA	VRK1	VRK1
WARS2	-1.9882524	1.16512945	0.52040198	NA	NA	WARS2
WDR12	-2.0742806	1.17417307	0.52040198	NA	NA	WDR12
WIT1	2.0830366	0.7403525	0.52040198	NA	NA	WIT1
XIAP	-1.9443922	1.16880732	0.52040198	NA	XIAP	XIAP
YTHDF2	-2.1610003	1.14729333	0.52040198	NA	NA	YTHDF2
ZBTB25	2.4548182	0.67193386	0.52040198	NA	NA	ZBTB25
ZBTB8OS	-1.889675	1.37315545	0.52040198	NA	NA	ZBTB8OS
ZC3HAV1	-2.1859277	1.27529601	0.52040198	NA	NA	ZC3HAV1
ZCCHC2	-1.8957973	1.26380685	0.52040198	NA	NA	ZCCHC2
ZCCHC24	-2.0265927	1.24020817	0.52040198	NA	NA	ZCCHC24
ZFP106	-1.9961314	1.17420263	0.52040198	NA	NA	ZFP106
ZFP90	-1.9415611	1.17520105	0.52040198	NA	NA	ZFP90
ZIC1	-2.0479031	1.37387861	0.52040198	NA	NA	ZIC1
ZKSCAN1	-1.9608204	1.19686149	0.52040198	NA	NA	ZKSCAN1
ZMYM5	-1.9307096	1.27978372	0.52040198	NA	NA	ZMYM5
ZNF226	-1.9817975	1.24942263	0.52040198	NA	NA	ZNF226
ZNF461	-2.0113456	1.32811005	0.52040198	NA	NA	ZNF461
ZNF485	-1.8866719	1.31103526	0.52040198	NA	NA	ZNF485
ZNF567	-2.1117909	1.29733533	0.52040198	NA	NA	ZNF567
ZNF8	-1.9349992	1.18664382	0.52040198	NA	NA	ZNF8
ORC2L	-1.9637817	1.46302166	0.52040198	ORC2L	NA	ORC2L
PAX8	2.1322173	0.81230924	0.52040198	PAX8	NA	PAX8
PLXNA2	-1.9743795	1.4904532	0.52040198	PLXNA2	PLXNA2	PLXNA2
TCEA3	2.0861038	0.79924679	0.52040198	TCEA3	NA	TCEA3
TLE4	-2.2209082	1.42075177	0.52040198	TLE4	TLE4	TLE4
TMTC2	-2.1768285	1.45902545	0.52040198	TMTC2	NA	TMTC2
TXNL1	-1.9519404	1.29571028	0.52040198	TXNL1	NA	TXNL1
ZBBX	2.4536853	0.74870382	0.52040198	ZBBX	NA	ZBBX

MAR-HAR 17vs19 q<0.01

SYMBOL	17vs19.d	17vs19.FC	17vs19.q	HAR	CAR	MAR
AHCY	3.8216127	0.6616698	0	NA	AHCY	AHCY

ALDH1A2	-2.9144595	1.37007936	0	NA	ALDH1A2	ALDH1A2
ANXA3	-3.0609158	1.75438391	0	NA	ANXA3	ANXA3
C17orf97	-3.4256608	1.89055616	0	NA	C17orf97	C17orf97
CDH5	-2.9995755	1.29949249	0	NA	CDH5	CDH5
DDX3Y	16.876416	0.17718856	0	NA	DDX3Y	DDX3Y
FABP2	-3.1328576	1.50995037	0	NA	FABP2	FABP2
FMNL2	-2.7567558	1.43286398	0	NA	FMNL2	FMNL2
GJA1	-3.1733784	2.19155709	0	NA	GJA1	GJA1
HBA1	-4.1478687	2.17084113	0	NA	HBA1	HBA1
HBM	-3.4403808	2.1078457	0	NA	HBM	HBM
HEATR3	-3.6981394	1.7535573	0	NA	HEATR3	HEATR3
LBH	-3.1891558	1.3292535	0	NA	LBH	LBH
LMNB1	-3.4886148	1.61615901	0	NA	LMNB1	LMNB1
LOC728411	3.7763355	0.60805245	0	NA	LOC728411	LOC728411
LRP1	3.4631467	0.73756441	0	NA	LRP1	LRP1
MAP9	-5.3005398	1.91553803	0	NA	MAP9	MAP9
MCM4	-2.715579	1.29650881	0	NA	MCM4	MCM4
ACP6	4.0312994	0.65072026	0	NA	NA	ACP6
AMFR	-8.7147112	2.11275886	0	NA	NA	AMFR
ASAH2B	3.1456685	0.69224484	0	NA	NA	ASAH2B
ATG12	-2.7573778	1.34585972	0	NA	NA	ATG12
B4GALT5	-4.2003741	1.4674269	0	NA	NA	B4GALT5
BBX	-2.725413	1.50335278	0	NA	NA	BBX
BCL11B	-3.0643644	1.39138997	0	NA	NA	BCL11B
BEND3	-2.9661982	1.24150406	0	NA	NA	BEND3
BOC	-2.8068475	1.27656318	0	NA	NA	BOC
C12orf75	3.5206713	0.67780015	0	NA	NA	C12orf75
C14orf101	-3.3229101	1.38652116	0	NA	NA	C14orf101
C17orf75	-2.9644849	1.77741209	0	NA	NA	C17orf75
C17orf82	3.0510839	0.71348687	0	NA	NA	C17orf82
C18orf19	-3.0628472	1.35442251	0	NA	NA	C18orf19
C1orf156	-4.0044376	1.62573154	0	NA	NA	C1orf156
C20orf94	-2.8759556	1.3876158	0	NA	NA	C20orf94
C21orf57	7.4867579	0.47059106	0	NA	NA	C21orf57
C2orf74	-6.9874199	1.97745048	0	NA	NA	C2orf74
C3orf70	-3.7496884	1.67548683	0	NA	NA	C3orf70
C8orf44	-2.7352321	1.43097518	0	NA	NA	C8orf44
C8orf59	-2.7116814	1.29717691	0	NA	NA	C8orf59
CACNB4	-8.4974559	2.93779503	0	NA	NA	CACNB4
CCDC59	-3.0452841	1.3867423	0	NA	NA	CCDC59
CCND1	-4.7674023	1.57837942	0	NA	NA	CCND1
CCNJ	-3.0257177	1.38453169	0	NA	NA	CCNJ
CDH1	-3.9085644	1.52767902	0	NA	NA	CDH1
CDKAL1	-6.8380691	1.93235063	0	NA	NA	CDKAL1
CLDN11	-2.8676812	1.53788098	0	NA	NA	CLDN11
COL3A1	-3.5343766	1.53911674	0	NA	NA	COL3A1
CRBN	-4.4936872	1.31982838	0	NA	NA	CRBN
CRH	3.942527	0.52240886	0	NA	NA	CRH
DARC	4.2546692	0.60437945	0	NA	NA	DARC
DCN	-2.7933295	1.41786959	0	NA	NA	DCN
DDX17	-15.817588	9.77010381	0	NA	NA	DDX17
DEM1	-4.1108629	1.39193028	0	NA	NA	DEM1
DKK1	-3.6500275	1.68388797	0	NA	NA	DKK1
DOCK7	-5.7660623	1.62898159	0	NA	NA	DOCK7
DSP	-2.7089628	1.75689966	0	NA	NA	DSP
EGR1	-3.2586722	1.70147338	0	NA	NA	EGR1
EXT1	-2.7939417	1.33590226	0	NA	NA	EXT1
EYA1	-3.2278247	1.50514894	0	NA	NA	EYA1

FAM110B	-2.71985	1.25767091	0	NA	NA	FAM110B
FAM129A	-3.7403437	1.4648489	0	NA	NA	FAM129A
FAM178B	3.3666818	0.67561962	0	NA	NA	FAM178B
FAM46C	-6.0599677	2.33991468	0	NA	NA	FAM46C
FAM60A	-2.842808	1.5858925	0	NA	NA	FAM60A
FBLN5	-2.8513505	1.67224398	0	NA	NA	FBLN5
FLJ32063	3.4212591	0.74778534	0	NA	NA	FLJ32063
FOS	-3.3201611	2.17313695	0	NA	NA	FOS
FUT11	-3.36033	1.34288763	0	NA	NA	FUT11
FZD3	-4.9729828	2.70223052	0	NA	NA	FZD3
GAS2	-3.0464526	1.65391062	0	NA	NA	GAS2
GATC	-4.9569503	1.5386214	0	NA	NA	GATC
GJC1	-2.8228101	1.41752178	0	NA	NA	GJC1
GK3P	-3.041541	1.40839884	0	NA	NA	GK3P
GLCE	-3.297914	1.36107722	0	NA	NA	GLCE
GNG11	-4.6550523	1.60596159	0	NA	NA	GNG11
GNG2	3.3049392	0.67064371	0	NA	NA	GNG2
GOLGA1	-2.8694446	1.38471146	0	NA	NA	GOLGA1
GPR155	-2.9358654	1.32266431	0	NA	NA	GPR155
GRIN2B	-2.7893494	1.86405719	0	NA	NA	GRIN2B
GSTT1	14.567224	0.29536778	0	NA	NA	GSTT1
GYPA	-3.3398883	1.50730896	0	NA	NA	GYPA
HADHB	-3.5500149	1.30255086	0	NA	NA	HADHB
HAS3	-2.7170941	1.34573993	0	NA	NA	HAS3
HBB	-6.0753265	3.07303904	0	NA	NA	HBB
HBG1	-5.0919514	2.4485439	0	NA	NA	HBG1
HCG11	-2.6850125	1.46858808	0	NA	NA	HCG11
HEMGN	-3.7746287	2.04436448	0	NA	NA	HEMGN
HLA-DMA	4.852325	0.47355534	0	NA	NA	HLA-DMA
HMBOX1	9.4320641	0.24538978	0	NA	NA	HMBOX1
HNRNPA0	-2.8345414	1.2364776	0	NA	NA	HNRNPA0
HSPA2	-3.2032388	1.45747749	0	NA	NA	HSPA2
HTR2A	-8.5978236	3.18105929	0	NA	NA	HTR2A
IMMP1L	-3.1167623	1.36790945	0	NA	NA	IMMP1L
ITGB3BP	3.7077098	0.66980147	0	NA	NA	ITGB3BP
ITPRIP	-4.8085233	1.38297881	0	NA	NA	ITPRIP
KGFLP2	-10.258703	2.72238842	0	NA	NA	KGFLP2
KIF3A	-3.0737407	1.52727468	0	NA	NA	KIF3A
KLHDC8A	4.5112655	0.68928407	0	NA	NA	KLHDC8A
KPNB1	-2.7311651	1.28255063	0	NA	NA	KPNB1
LCLAT1	-4.0539244	1.40814788	0	NA	NA	LCLAT1
LMO7	-2.8636254	1.393642	0	NA	NA	LMO7
LOC100129387	-3.3914122	1.61354421	0	NA	NA	LOC100129387
LOC144486	-2.7714156	1.27120705	0	NA	NA	LOC144486
LOC221710	-2.9769436	1.70109375	0	NA	NA	LOC221710
LOC728554	3.1656053	0.78126995	0	NA	NA	LOC728554
LRCH1	-2.9817491	1.48754686	0	NA	NA	LRCH1
LRRC37A4	-3.3698976	1.68280214	0	NA	NA	LRRC37A4
LSM11	-3.2322088	1.71262628	0	NA	NA	LSM11
LSM2	-3.2913556	1.47098338	0	NA	NA	LSM2
LTF	2.9664019	0.72724447	0	NA	NA	LTF
LUM	-3.2260897	1.95091755	0	NA	NA	LUM
MAMDC2	-3.215659	1.43587342	0	NA	NA	MAMDC2
MDM4	3.0196712	0.78043155	0	NA	NA	MDM4
MED6	-3.3017157	1.47041287	0	NA	NA	MED6
METT10D	-2.7754442	1.3206454	0	NA	NA	METT10D
METTL10	3.7397373	0.69287372	0	NA	NA	METTL10

MFN1	-4.2921499	1.39514778	0	NA	NA	MFN1
MLH3	-3.3077975	1.27531588	0	NA	NA	MLH3
MRPS10	-2.8214155	1.36885454	0	NA	NA	MRPS10
MRPS16	-3.3590271	1.35298982	0	NA	NA	MRPS16
MYOCD	-3.082473	1.36241313	0	NA	NA	MYOCD
NAV2	-4.3314651	1.32226567	0	NA	NA	NAV2
NHSL1	-3.5147792	1.30160174	0	NA	NA	NHSL1
NID2	-3.5619483	1.75737721	0	NA	NA	NID2
NOV	-2.847625	1.70505348	0	NA	NA	NOV
OCLN	-3.4971851	1.51101582	0	NA	NA	OCLN
ODF1	4.6347296	0.55370102	0	NA	NA	ODF1
PCDHA10	-2.865924	1.36392247	0	NA	NA	PCDHA10
PCSK5	-3.0316199	1.39730482	0	NA	NA	PCSK5
PDGFC	-4.4359191	1.58063729	0	NA	NA	PDGFC
PDGFRL	-3.5086878	2.13452466	0	NA	NA	PDGFRL
PEX7	-2.8320448	1.4155305	0	NA	NA	PEX7
PFDN5	-4.0513292	1.36869283	0	NA	NA	PFDN5
PGCP	-2.6868551	1.3440617	0	NA	NA	PGCP
POLR1A	-3.0997891	1.34305056	0	NA	NA	POLR1A
POLR3E	-3.0437648	1.34474236	0	NA	NA	POLR3E
POMZP3	10.36362	0.3317839	0	NA	NA	POMZP3
PPIG	-3.1038205	1.48329993	0	NA	NA	PPIG
PROCR	-3.6552633	1.42352553	0	NA	NA	PROCR
PRSS35	-2.7653919	1.76424926	0	NA	NA	PRSS35
PSMB5	-3.5088104	1.32463521	0	NA	NA	PSMB5
PTN	-3.5390284	1.42543178	0	NA	NA	PTN
PTPRS	-3.3876319	1.37662535	0	NA	NA	PTPRS
RCL1	-3.8249932	1.39883248	0	NA	NA	RCL1
RCSD1	-2.889604	1.52154846	0	NA	NA	RCSD1
RHOBTB2	3.0460716	0.75270106	0	NA	NA	RHOBTB2
RPL14	-3.225496	1.3377925	0	NA	NA	RPL14
RPL27A	-4.8315189	1.57672313	0	NA	NA	RPL27A
RPL38	-3.7755266	1.3614783	0	NA	NA	RPL38
RPS23	5.6691597	0.65934831	0	NA	NA	RPS23
RPS4Y1	34.031146	0.01044102	0	NA	NA	RPS4Y1
RWDD2A	-2.843736	1.3185053	0	NA	NA	RWDD2A
SAR1A	-11.355276	3.05639008	0	NA	NA	SAR1A
SDC3	3.9075778	0.68806994	0	NA	NA	SDC3
SERINC3	-3.5661646	1.45310724	0	NA	NA	SERINC3
SERPINH1	-3.5150056	1.42815054	0	NA	NA	SERPINH1
SETD6	-3.1832995	1.57052209	0	NA	NA	SETD6
SLC20A2	-2.8595998	1.4429525	0	NA	NA	SLC20A2
SLC41A2	-3.4222912	1.39333258	0	NA	NA	SLC41A2
SNAP29	-6.3300364	1.64923889	0	NA	NA	SNAP29
SPARC	-3.0910308	1.34124545	0	NA	NA	SPARC
SPATA5	-3.2914178	1.4958473	0	NA	NA	SPATA5
TBX15	-3.2246332	1.53301564	0	NA	NA	TBX15
TERF1	-3.228146	1.340048	0	NA	NA	TERF1
TMEM100	-3.0007249	1.60591256	0	NA	NA	TMEM100
TMEM106C	-2.9400017	1.50954861	0	NA	NA	TMEM106C
TMEM159	-5.3443917	2.63754195	0	NA	NA	TMEM159
TMEM169	-2.7655447	1.25706554	0	NA	NA	TMEM169
TMEM178	-2.6904621	1.39217609	0	NA	NA	TMEM178
TMEM33	-2.6658069	1.35762943	0	NA	NA	TMEM33
TNNI3K	4.7634285	0.57583172	0	NA	NA	TNNI3K
TTC32	-6.837454	1.87150878	0	NA	NA	TTC32
TWSG1	-2.8596631	1.33741803	0	NA	NA	TWSG1
TXN2	-3.1741458	1.30516754	0	NA	NA	TXN2

UBE2H	-2.9120245	1.29370931	0	NA	NA	UBE2H
ULBP2	3.235594	0.65035909	0	NA	NA	ULBP2
UPF3A	-2.8879641	1.4299963	0	NA	NA	UPF3A
USP7	-3.4224056	1.48698682	0	NA	NA	USP7
USP9Y	24.930165	0.03392756	0	NA	NA	USP9Y
VGLL4	3.9162699	0.49989022	0	NA	NA	VGLL4
ZFPM2	-4.5503508	1.52778901	0	NA	NA	ZFPM2
ZNF281	-3.4402922	1.32854507	0	NA	NA	ZNF281
ZNF33B	3.2726387	0.53745074	0	NA	NA	ZNF33B
ZNF410	-2.9582169	1.30315211	0	NA	NA	ZNF410
ZNF673	-2.8295142	1.35068033	0	NA	NA	ZNF673
ZNF678	-7.0487526	3.15095782	0	NA	NA	ZNF678
ZNHIT6	-2.8517864	1.32338501	0	NA	NA	ZNHIT6
OLFML2B	-2.7124295	1.40929775	0	NA	OLFML2B	OLFML2B
PCDH11Y	3.3331052	0.66928933	0	NA	PCDH11Y	PCDH11Y
PCDHB2	-3.4170174	1.79354433	0	NA	PCDHB2	PCDHB2
PEX13	-4.0700533	1.51194967	0	NA	PEX13	PEX13
SLC44A1	-3.7981917	1.47477665	0	NA	SLC44A1	SLC44A1
SOX21	3.5374339	0.65163046	0	NA	SOX21	SOX21
SVIL	-2.9796019	1.44575569	0	NA	SVIL	SVIL
VAMP3	-3.8246818	1.30583534	0	NA	VAMP3	VAMP3
WDR86	3.2488551	0.69935269	0	NA	WDR86	WDR86
WNK3	-3.2041129	1.50150119	0	NA	WNK3	WNK3
ZFX	-2.982037	1.46067025	0	NA	ZFX	ZFX
ZNF217	-3.8102481	1.65942746	0	NA	ZNF217	ZNF217
AKAP12	-2.2786081	1.22442061	0.15408684	NA	AKAP12	AKAP12
BMPR1B	2.6068905	0.57627958	0.15408684	NA	BMPR1B	BMPR1B
C6orf225	-2.4203083	1.2979177	0.15408684	NA	C6orf225	C6orf225
CCL2	-2.4253942	1.60984817	0.15408684	NA	CCL2	CCL2
CTNNB1	-2.3310371	1.55618518	0.15408684	NA	CTNNB1	CTNNB1
CUL2	2.8363592	0.7865667	0.15408684	NA	CUL2	CUL2
DPY19L2	-2.3058242	1.26838212	0.15408684	NA	DPY19L2	DPY19L2
EIF1AX	-2.2607281	1.24915554	0.15408684	NA	EIF1AX	EIF1AX
EMCN	-2.4724299	1.31793197	0.15408684	NA	EMCN	EMCN
EPB41L1	-2.5552412	1.24069766	0.15408684	NA	EPB41L1	EPB41L1
ESCO1	-2.5615491	1.28333929	0.15408684	NA	ESCO1	ESCO1
FASTKD2	-2.439101	1.25346273	0.15408684	NA	FASTKD2	FASTKD2
GLRB	-2.5792542	1.28960532	0.15408684	NA	GLRB	GLRB
HCCS	-2.2828589	1.20536856	0.15408684	NA	HCCS	HCCS
HDDC2	2.513864	0.71840442	0.15408684	NA	HDDC2	HDDC2
HYI	-2.5821409	1.26010427	0.15408684	NA	HYI	HYI
KHDRBS2	-2.310516	1.28131427	0.15408684	NA	KHDRBS2	KHDRBS2
LOC338758	-2.632777	1.41919053	0.15408684	NA	LOC338758	LOC338758
LOC723809	2.8695723	0.71704125	0.15408684	NA	LOC723809	LOC723809
AACS	2.7772252	0.81928872	0.15408684	NA	NA	AACS
AASDH	-2.2837131	1.33839499	0.15408684	NA	NA	AASDH
AASS	-2.3766554	1.24422141	0.15408684	NA	NA	AASS
ADAMTS5	-2.5578755	1.32124927	0.15408684	NA	NA	ADAMTS5
ADORA2B	-2.6464446	1.52409042	0.15408684	NA	NA	ADORA2B
AKAP5	-2.4516203	1.43557392	0.15408684	NA	NA	AKAP5
ALKBH3	-2.4735768	1.26430568	0.15408684	NA	NA	ALKBH3
ANGPT2	-2.3641777	1.44208847	0.15408684	NA	NA	ANGPT2
ANKRD13A	-2.609523	1.27601125	0.15408684	NA	NA	ANKRD13A
ARFGEF2	-2.3904299	1.20561488	0.15408684	NA	NA	ARFGEF2
BPNT1	-2.6408925	1.2100756	0.15408684	NA	NA	BPNT1
BUB3	-2.4370156	1.20337506	0.15408684	NA	NA	BUB3
C10orf78	-2.3176742	1.3305325	0.15408684	NA	NA	C10orf78
C14orf118	-2.3531121	1.33016407	0.15408684	NA	NA	C14orf118

C21orf119	2.844505	0.67810524	0.15408684	NA	NA	C21orf119
C6orf52	2.7647041	0.73088546	0.15408684	NA	NA	C6orf52
CASC3	-2.6647051	1.30076511	0.15408684	NA	NA	CASC3
CAST	-2.4073902	1.23782379	0.15408684	NA	NA	CAST
CBX5	-2.3490675	1.38954567	0.15408684	NA	NA	CBX5
CCNT1	-2.4365363	1.58989758	0.15408684	NA	NA	CCNT1
CD302	-2.5345817	1.43298482	0.15408684	NA	NA	CD302
CLSTN2	-2.4636842	1.6788568	0.15408684	NA	NA	CLSTN2
CNIH4	-2.4647397	1.24346588	0.15408684	NA	NA	CNIH4
COL11A1	-2.4263028	1.58502373	0.15408684	NA	NA	COL11A1
COL1A2	-2.5247003	1.53757227	0.15408684	NA	NA	COL1A2
COL21A1	-2.2715263	1.39844564	0.15408684	NA	NA	COL21A1
CPEB3	-2.3418384	1.29947995	0.15408684	NA	NA	CPEB3
CRB1	2.7218365	0.77834041	0.15408684	NA	NA	CRB1
CSRNP3	-2.6324874	1.77680543	0.15408684	NA	NA	CSRNP3
CTSL1	-2.5466408	1.46599936	0.15408684	NA	NA	CTSL1
CYP1B1	-2.5728104	1.59423958	0.15408684	NA	NA	CYP1B1
DAPK1	2.8189297	0.76157288	0.15408684	NA	NA	DAPK1
DGAT2	2.9155871	0.76247906	0.15408684	NA	NA	DGAT2
DGKA	2.6749752	0.76261627	0.15408684	NA	NA	DGKA
DHRS11	-2.3061322	1.45353867	0.15408684	NA	NA	DHRS11
EFEMP1	-2.2601246	1.512079	0.15408684	NA	NA	EFEMP1
ENDOD1	-2.6652223	1.23841843	0.15408684	NA	NA	ENDOD1
ENTPD4	-2.2639859	1.26243617	0.15408684	NA	NA	ENTPD4
EPC1	-2.5084281	1.27380607	0.15408684	NA	NA	EPC1
ERCC8	-2.3758432	1.20989691	0.15408684	NA	NA	ERCC8
EXOSC2	-2.507613	1.19153459	0.15408684	NA	NA	EXOSC2
FBXW10	2.5525607	0.7559772	0.15408684	NA	NA	FBXW10
GABRB1	2.6685844	0.59317539	0.15408684	NA	NA	GABRB1
GALNT1	-2.5505422	1.2497617	0.15408684	NA	NA	GALNT1
GSK3B	-2.3125855	1.8209692	0.15408684	NA	NA	GSK3B
HHAT	-2.6642639	1.37203725	0.15408684	NA	NA	HHAT
HHLA3	2.5531852	0.84325886	0.15408684	NA	NA	HHLA3
ITIH2	-2.5172604	1.57287272	0.15408684	NA	NA	ITIH2
ITPRIPL2	-2.2714985	1.18882915	0.15408684	NA	NA	ITPRIPL2
KATNA1	-2.4099145	1.20178113	0.15408684	NA	NA	KATNA1
KCNG2	-2.3943031	1.31400163	0.15408684	NA	NA	KCNG2
KIAA1033	-2.5258399	1.4238757	0.15408684	NA	NA	KIAA1033
KIF21A	-2.5343021	1.31543011	0.15408684	NA	NA	KIF21A
KLHL5	-2.5304512	1.28779043	0.15408684	NA	NA	KLHL5
KRT75	2.5310171	0.83537088	0.15408684	NA	NA	KRT75
LDHAL6A	2.7683764	0.7212885	0.15408684	NA	NA	LDHAL6A
LOC253039	-2.2687965	1.31648364	0.15408684	NA	NA	LOC253039
LOC344595	-2.3850471	1.28620698	0.15408684	NA	NA	LOC344595
LYVE1	-2.2907557	1.9875599	0.15408684	NA	NA	LYVE1
MALAT1	-2.6059227	1.84262573	0.15408684	NA	NA	MALAT1
MAML3	-2.2695581	1.18278914	0.15408684	NA	NA	MAML3
MAN1A1	-2.3485979	1.47403349	0.15408684	NA	NA	MAN1A1
MARS2	-2.5238092	1.38801805	0.15408684	NA	NA	MARS2
MED1	-2.6381891	1.36884522	0.15408684	NA	NA	MED1
MMGT1	-2.3779571	1.23820151	0.15408684	NA	NA	MMGT1
MRC2	-2.6443084	1.27734229	0.15408684	NA	NA	MRC2
NCAPG	-2.3366229	1.38654065	0.15408684	NA	NA	NCAPG
NEK10	-2.2733071	1.3437932	0.15408684	NA	NA	NEK10
NEK11	2.6425471	0.83268837	0.15408684	NA	NA	NEK11
OLFML3	-2.5775999	1.68013647	0.15408684	NA	NA	OLFML3
PBLD	-2.3296979	1.38976503	0.15408684	NA	NA	PBLD
PEX3	-2.3367843	1.24747703	0.15408684	NA	NA	PEX3

PHAX	-2.6002356	1.26474595	0.15408684	NA	NA	PHAX
PIGF	-2.5599996	1.22772039	0.15408684	NA	NA	PIGF
PITRM1	2.7774431	0.78930237	0.15408684	NA	NA	PITRM1
PNOC	2.5737188	0.75599796	0.15408684	NA	NA	PNOC
PNPLA8	-2.3946008	1.24409588	0.15408684	NA	NA	PNPLA8
PPIB	-2.3549659	1.28657741	0.15408684	NA	NA	PPIB
PRMT8	-2.3041096	1.2286679	0.15408684	NA	NA	PRMT8
PRPF39	-2.3193488	1.47045554	0.15408684	NA	NA	PRPF39
PTCD3	-2.3290937	1.20928246	0.15408684	NA	NA	PTCD3
RASA2	-2.3064891	1.37965524	0.15408684	NA	NA	RASA2
RBMS3	-2.5382434	1.17434759	0.15408684	NA	NA	RBMS3
RPGRIP1L	-2.2612711	1.2759607	0.15408684	NA	NA	RPGRIP1L
RPP25	2.9209582	0.75889774	0.15408684	NA	NA	RPP25
SCARNA2	2.7205174	0.69920881	0.15408684	NA	NA	SCARNA2
SEC31A	-2.4401949	1.27613832	0.15408684	NA	NA	SEC31A
SENP8	-2.3818281	1.17602863	0.15408684	NA	NA	SENP8
SFRS12IP1	-2.3118187	1.28184605	0.15408684	NA	NA	SFRS12IP1
SIAH3	-2.5901526	1.8345651	0.15408684	NA	NA	SIAH3
SLC35B3	-2.2959573	1.31271787	0.15408684	NA	NA	SLC35B3
SMURF2	-2.263931	1.37802813	0.15408684	NA	NA	SMURF2
SOAT2	2.5770608	0.73759794	0.15408684	NA	NA	SOAT2
SRL	-2.5935564	1.28325817	0.15408684	NA	NA	SRL
TAOK1	-2.4294969	1.56100579	0.15408684	NA	NA	TAOK1
TCL1A	2.7836999	0.79808427	0.15408684	NA	NA	TCL1A
TRAPPC5	-2.4614767	1.30937375	0.15408684	NA	NA	TRAPPC5
TRIM69	-2.3625574	1.46343493	0.15408684	NA	NA	TRIM69
TUBB6	-2.3299468	1.45598407	0.15408684	NA	NA	TUBB6
UHRF1	-2.5674501	1.3798915	0.15408684	NA	NA	UHRF1
VDAC3	-2.4883873	1.21200174	0.15408684	NA	NA	VDAC3
VPS29	-2.4892411	1.22026069	0.15408684	NA	NA	VPS29
WDFY1	-2.3148448	1.31487986	0.15408684	NA	NA	WDFY1
ZCCHC6	-2.4770813	1.23305412	0.15408684	NA	NA	ZCCHC6
ZEB1	-2.4675301	1.41869147	0.15408684	NA	NA	ZEB1
ZIC2	-2.5628214	1.55072803	0.15408684	NA	NA	ZIC2
ZNF2	-2.3970363	1.41933831	0.15408684	NA	NA	ZNF2
ZNF568	-2.5273151	1.28010059	0.15408684	NA	NA	ZNF568
ZNF646	-2.3111948	1.33040038	0.15408684	NA	NA	ZNF646
ZNF827	-2.4277283	1.4249109	0.15408684	NA	NA	ZNF827
NKAIN2	-2.4988171	1.40672174	0.15408684	NA	NKAIN2	NKAIN2
PHB	-2.3630554	1.18715953	0.15408684	NA	PHB	PHB
PHF2	2.7253249	0.72802401	0.15408684	NA	PHF2	PHF2
PRPH2	2.8264552	0.70562354	0.15408684	NA	PRPH2	PRPH2
RP9	-2.5188738	1.24645567	0.15408684	NA	RP9	RP9
SSR3	-2.5990322	1.35165952	0.15408684	NA	SSR3	SSR3
SYPL2	2.7515259	0.65328867	0.15408684	NA	SYPL2	SYPL2
TM7SF4	2.6204674	0.77172555	0.15408684	NA	TM7SF4	TM7SF4
ZNF326	-2.3209629	1.23916508	0.15408684	NA	ZNF326	ZNF326
ZNF644	-2.5407901	1.55442586	0.15408684	NA	ZNF644	ZNF644
ABCA17P	2.3343513	0.73624021	0.52040198	NA	ABCA17P	ABCA17P
APOL1	-1.9121602	1.38224102	0.52040198	NA	APOL1	APOL1
C14orf178	2.1092287	0.78711472	0.52040198	NA	C14orf178	C14orf178
C3orf17	-1.9128026	1.21356318	0.52040198	NA	C3orf17	C3orf17
CD47	-1.9775249	1.21294482	0.52040198	NA	CD47	CD47
CDC42	2.1906733	0.81494459	0.52040198	NA	CDC42	CDC42
CLN5	-1.9063071	1.27256393	0.52040198	NA	CLN5	CLN5
COL4A1	-2.0671428	1.22169476	0.52040198	NA	COL4A1	COL4A1
DEXI	2.1112593	0.83873045	0.52040198	NA	DEXI	DEXI
EIF2S3	-1.9601947	1.16509672	0.52040198	NA	EIF2S3	EIF2S3

FLJ39653	-2.0964844	1.20766404	0.52040198	NA	FLJ39653	FLJ39653
HS3ST4	-1.889094	1.23721883	0.52040198	NA	HS3ST4	HS3ST4
HSDL2	-1.8965978	1.29385895	0.52040198	NA	HSDL2	HSDL2
ICOS	2.4927746	0.75229582	0.52040198	NA	ICOS	ICOS
IDH2	-2.0983052	1.21860617	0.52040198	NA	IDH2	IDH2
KIAA0831	-2.1327187	1.19270868	0.52040198	NA	KIAA0831	KIAA0831
LOC100192378	2.4134955	0.79027925	0.52040198	NA	LOC10019237 8	LOC10019237 8
MCPH1	-2.1621202	1.1819172	0.52040198	NA	MCPH1	MCPH1
MKKS	2.3111227	0.77454001	0.52040198	NA	MKKS	MKKS
MPPED1	-2.0119839	1.21751657	0.52040198	NA	MPPED1	MPPED1
ABCC8	2.4328791	0.76046742	0.52040198	NA	NA	ABCC8
ADK	-2.1302733	1.31924955	0.52040198	NA	NA	ADK
AHI1	-1.9220662	1.18209537	0.52040198	NA	NA	AHI1
ANKRD42	2.2749257	0.82264758	0.52040198	NA	NA	ANKRD42
ANP32A	-2.0033225	1.22966807	0.52040198	NA	NA	ANP32A
ANXA2	-1.9997924	1.45329543	0.52040198	NA	NA	ANXA2
APOD	2.2826264	0.73716617	0.52040198	NA	NA	APOD
ARFIP1	-2.1744556	1.30306661	0.52040198	NA	NA	ARFIP1
ARID2	-2.1601992	1.33696186	0.52040198	NA	NA	ARID2
ATPAF2	-2.0593733	1.23084647	0.52040198	NA	NA	ATPAF2
BAG3	-1.9384694	1.31468397	0.52040198	NA	NA	BAG3
BCAS1	-2.084448	1.20803661	0.52040198	NA	NA	BCAS1
BFSP2	2.1901177	0.82295316	0.52040198	NA	NA	BFSP2
BMP2K	-1.9532441	1.16821622	0.52040198	NA	NA	BMP2K
BMP8A	2.1058632	0.73990316	0.52040198	NA	NA	BMP8A
BOK	2.3721644	0.76793487	0.52040198	NA	NA	BOK
BRAP	-2.1352878	1.2534199	0.52040198	NA	NA	BRAP
C11orf57	-2.0509347	1.18894566	0.52040198	NA	NA	C11orf57
C15orf61	-1.9751662	1.23347746	0.52040198	NA	NA	C15orf61
C17orf86	-2.0339511	1.26658073	0.52040198	NA	NA	C17orf86
C1orf27	-1.974642	1.30984575	0.52040198	NA	NA	C1orf27
C21orf49	2.4976927	0.79183242	0.52040198	NA	NA	C21orf49
C2orf42	-1.8927023	1.20108286	0.52040198	NA	NA	C2orf42
C3orf57	-1.8893237	1.19816256	0.52040198	NA	NA	C3orf57
C7orf51	-2.1071945	1.33640561	0.52040198	NA	NA	C7orf51
CACNG2	2.4539067	0.79018397	0.52040198	NA	NA	CACNG2
CAMK2N1	-2.068048	1.26749433	0.52040198	NA	NA	CAMK2N1
CAPN8	2.062243	0.73588244	0.52040198	NA	NA	CAPN8
CC2D2B	2.3528731	0.65284613	0.52040198	NA	NA	CC2D2B
CCDC125	-2.1515828	1.23519787	0.52040198	NA	NA	CCDC125
CCDC140	2.1053809	0.80331706	0.52040198	NA	NA	CCDC140
CCDC28A	-2.1309696	1.26417516	0.52040198	NA	NA	CCDC28A
CECR1	2.2627415	0.76025114	0.52040198	NA	NA	CECR1
CEP110	-2.1125622	1.19484619	0.52040198	NA	NA	CEP110
CHEK2	2.114179	0.78587628	0.52040198	NA	NA	CHEK2
CLDN16	2.3310751	0.79347685	0.52040198	NA	NA	CLDN16
COL9A2	2.1659016	0.70015242	0.52040198	NA	NA	COL9A2
COX15	-1.9051802	1.2085638	0.52040198	NA	NA	COX15
CPXM2	2.1325825	0.86026774	0.52040198	NA	NA	CPXM2
CST7	2.3033608	0.78005281	0.52040198	NA	NA	CST7
CTSC	-2.1849962	1.37608816	0.52040198	NA	NA	CTSC
CTTNBP2	-2.1819356	1.25458024	0.52040198	NA	NA	CTTNBP2
CXorf38	-2.0975988	1.39567315	0.52040198	NA	NA	CXorf38
CXorf59	2.0504571	0.78277683	0.52040198	NA	NA	CXorf59
DCST1	2.1424342	0.71034187	0.52040198	NA	NA	DCST1
DEGS1	-2.072779	1.1858773	0.52040198	NA	NA	DEGS1
DHTKD1	-2.0802506	1.22301036	0.52040198	NA	NA	DHTKD1

DNAH7	2.41911	0.70773173	0.52040198	NA	NA	DNAH7
DNAJC5G	2.0911647	0.71262761	0.52040198	NA	NA	DNAJC5G
EFHB	-1.9809965	1.37724656	0.52040198	NA	NA	EFHB
EIF2C3	-2.0435392	1.34298354	0.52040198	NA	NA	EIF2C3
EIF3G	-2.2129915	1.18242727	0.52040198	NA	NA	EIF3G
EIF5B	-2.0811152	1.23309634	0.52040198	NA	NA	EIF5B
EMILIN3	2.2222533	0.73846615	0.52040198	NA	NA	EMILIN3
ERAP2	-2.1155597	1.16759146	0.52040198	NA	NA	ERAP2
ERP44	-2.024124	1.18241014	0.52040198	NA	NA	ERP44
FAM122A	-1.9726457	1.17223446	0.52040198	NA	NA	FAM122A
FAM131C	2.1503249	0.82193538	0.52040198	NA	NA	FAM131C
FAM150B	2.0544744	0.70944555	0.52040198	NA	NA	FAM150B
FAM186B	2.1947687	0.78534665	0.52040198	NA	NA	FAM186B
FAM35A	-2.1911292	1.24569496	0.52040198	NA	NA	FAM35A
FARSB	-2.0363338	1.38382939	0.52040198	NA	NA	FARSB
FBN1	-2.2223297	1.35000795	0.52040198	NA	NA	FBN1
FER	-2.0464847	1.36356216	0.52040198	NA	NA	FER
FGL2	-2.0653139	1.59204968	0.52040198	NA	NA	FGL2
GABRB2	2.371876	0.68809537	0.52040198	NA	NA	GABRB2
GAR1	-2.1861567	1.23255098	0.52040198	NA	NA	GAR1
GATAD1	-2.0920612	1.22377274	0.52040198	NA	NA	GATAD1
GEMIN8	-2.1429039	1.22834499	0.52040198	NA	NA	GEMIN8
GHRHR	2.0826463	0.74904975	0.52040198	NA	NA	GHRHR
GIMAP2	-2.0266119	1.33596833	0.52040198	NA	NA	GIMAP2
GLT8D2	-1.8940871	1.25220948	0.52040198	NA	NA	GLT8D2
GLTP	-1.9432979	1.25181329	0.52040198	NA	NA	GLTP
GNAL	-2.0080944	1.86789974	0.52040198	NA	NA	GNAL
GNG10	-2.0686584	1.27356848	0.52040198	NA	NA	GNG10
GNPDA1	-2.0278047	1.20704513	0.52040198	NA	NA	GNPDA1
GNPNAT1	-2.125466	1.17206348	0.52040198	NA	NA	GNPNAT1
GPC1	2.1876605	0.78278178	0.52040198	NA	NA	GPC1
GPR83	-1.9193755	1.41579111	0.52040198	NA	NA	GPR83
GPR89B	-2.0351575	1.17692447	0.52040198	NA	NA	GPR89B
GRB7	2.1377094	0.7432128	0.52040198	NA	NA	GRB7
GSTO2	2.2568222	0.69059817	0.52040198	NA	NA	GSTO2
GTF2H1	-1.8994516	1.16105061	0.52040198	NA	NA	GTF2H1
HBP1	-1.9735302	1.26538923	0.52040198	NA	NA	HBP1
HEATR5A	-1.902767	1.34983554	0.52040198	NA	NA	HEATR5A
HIBADH	-1.9814384	1.21161046	0.52040198	NA	NA	HIBADH
HINT3	-1.9441384	1.60393052	0.52040198	NA	NA	HINT3
HMGCR	2.0532203	0.86415929	0.52040198	NA	NA	HMGCR
HSD11B2	2.2925167	0.72482175	0.52040198	NA	NA	HSD11B2
HTATIP2	2.0700273	0.79864545	0.52040198	NA	NA	HTATIP2
INTS9	-2.0402097	1.16961044	0.52040198	NA	NA	INTS9
ISLR2	-1.9758955	1.33074964	0.52040198	NA	NA	ISLR2
ISOC1	2.4713551	0.77933133	0.52040198	NA	NA	ISOC1
KCTD16	2.354395	0.60252427	0.52040198	NA	NA	KCTD16
KIAA0090	-2.1808647	1.23180295	0.52040198	NA	NA	KIAA0090
KIAA1586	-2.1571613	1.28117498	0.52040198	NA	NA	KIAA1586
KIF5A	-2.0342757	1.35590469	0.52040198	NA	NA	KIF5A
KRT74	2.1239248	0.77422916	0.52040198	NA	NA	KRT74
KRT82	2.4586007	0.73755047	0.52040198	NA	NA	KRT82
KTELC1	-2.1984993	1.29330049	0.52040198	NA	NA	KTELC1
LAPTM4B	-1.9216933	1.34113928	0.52040198	NA	NA	LAPTM4B
LHB	-2.0833171	1.28891523	0.52040198	NA	NA	LHB
LLPH	-1.999817	1.1937156	0.52040198	NA	NA	LLPH
LMX1A	2.4550773	0.77172307	0.52040198	NA	NA	LMX1A
LOC144438	-2.0132641	1.26333967	0.52040198	NA	NA	LOC144438

LOC153328	2.4023878	0.77768044	0.52040198	NA	NA	LOC153328
LOC283174	-2.1237479	1.27073276	0.52040198	NA	NA	LOC283174
LOC340357	2.3057571	0.73874396	0.52040198	NA	NA	LOC340357
LOC374491	-1.9806138	1.2798857	0.52040198	NA	NA	LOC374491
LRRC17	-1.9927652	1.27770681	0.52040198	NA	NA	LRRC17
LRRC37A2	2.1760499	0.82621777	0.52040198	NA	NA	LRRC37A2
LRRC57	-2.0589527	1.30361128	0.52040198	NA	NA	LRRC57
LSG1	-1.8904563	1.20921704	0.52040198	NA	NA	LSG1
MAP3K12	2.1973903	0.81938356	0.52040198	NA	NA	MAP3K12
MAPK3	2.0914245	0.85292565	0.52040198	NA	NA	MAPK3
MAS1	2.0962005	0.72382428	0.52040198	NA	NA	MAS1
MB	2.27274	0.71881396	0.52040198	NA	NA	MB
MCC	-2.1187613	1.29070636	0.52040198	NA	NA	MCC
MFSD6L	2.2174655	0.7702194	0.52040198	NA	NA	MFSD6L
MMP16	-1.9949701	1.25948912	0.52040198	NA	NA	MMP16
MRC1	-2.0059435	2.02300477	0.52040198	NA	NA	MRC1
MRPL2	-1.902676	1.21328046	0.52040198	NA	NA	MRPL2
MSL2	-1.9960172	1.29359364	0.52040198	NA	NA	MSL2
MTERF	-2.1988882	1.20543362	0.52040198	NA	NA	MTERF
MYH10	-1.9686168	1.30885144	0.52040198	NA	NA	MYH10
NCDN	2.4662698	0.81990466	0.52040198	NA	NA	NCDN
NDC80	-2.0028732	1.29395268	0.52040198	NA	NA	NDC80
NDUFS5	2.2017389	0.83524493	0.52040198	NA	NA	NDUFS5
NLN	-2.1851909	1.1969421	0.52040198	NA	NA	NLN
NOSTRIN	-2.1178711	1.29669248	0.52040198	NA	NA	NOSTRIN
NSL1	-2.0645267	1.2104069	0.52040198	NA	NA	NSL1
OMD	-2.2479875	1.87282089	0.52040198	NA	NA	OMD
ORMDL2	-1.9860724	1.19784337	0.52040198	NA	NA	ORMDL2
PAIP2B	2.1305932	0.79107342	0.52040198	NA	NA	PAIP2B
PAPSS2	-2.2111151	1.2575037	0.52040198	NA	NA	PAPSS2
PCDH8	2.4071771	0.71515048	0.52040198	NA	NA	PCDH8
PCDHGA1	2.307887	0.79195605	0.52040198	NA	NA	PCDHGA1
PCGF6	-1.885943	1.27052744	0.52040198	NA	NA	PCGF6
PCLO	2.1715754	0.80619704	0.52040198	NA	NA	PCLO
PCSK6	2.4986726	0.84049685	0.52040198	NA	NA	PCSK6
PDE1A	-2.2262416	1.28671827	0.52040198	NA	NA	PDE1A
PEX26	-2.1140121	1.21066874	0.52040198	NA	NA	PEX26
PGM3	-2.0435103	1.26859256	0.52040198	NA	NA	PGM3
PLAG1	-1.9965254	1.30066752	0.52040198	NA	NA	PLAG1
PON1	2.0833775	0.77710618	0.52040198	NA	NA	PON1
POP5	-2.0889906	1.233827	0.52040198	NA	NA	POP5
POU2F2	-1.9716952	1.24277587	0.52040198	NA	NA	POU2F2
PPM1D	-2.141691	1.23000435	0.52040198	NA	NA	PPM1D
PPPDE1	-2.2219879	1.26451279	0.52040198	NA	NA	PPPDE1
PRIMA1	2.2221399	0.74154802	0.52040198	NA	NA	PRIMA1
PRRX1	-2.2172145	1.3321327	0.52040198	NA	NA	PRRX1
PSAPL1	2.1745227	0.79341248	0.52040198	NA	NA	PSAPL1
PSD3	2.261628	0.7178862	0.52040198	NA	NA	PSD3
PSIP1	-1.991087	1.24723292	0.52040198	NA	NA	PSIP1
QTRTD1	-1.9218234	1.17645254	0.52040198	NA	NA	QTRTD1
R3HDM2	-1.8986794	1.27911761	0.52040198	NA	NA	R3HDM2
RAI14	-1.9661168	1.3050944	0.52040198	NA	NA	RAI14
RANBP3L	-1.8840796	1.21065335	0.52040198	NA	NA	RANBP3L
RBKS	-1.9427115	1.24363587	0.52040198	NA	NA	RBKS
RBM27	-1.8993699	1.21577946	0.52040198	NA	NA	RBM27
RBMS1	-2.1281534	1.34581448	0.52040198	NA	NA	RBMS1
RDX	-2.0118485	1.22780659	0.52040198	NA	NA	RDX
REG3A	2.1035545	0.83289156	0.52040198	NA	NA	REG3A

RGS22	2.1723	0.76006672	0.52040198	NA	NA	RGS22
RNF115	-2.2382884	1.31117258	0.52040198	NA	NA	RNF115
ROD1	-2.0930953	1.32272881	0.52040198	NA	NA	ROD1
RPP14	-2.1327202	1.32992645	0.52040198	NA	NA	RPP14
RPS4X	-2.1992759	1.1435872	0.52040198	NA	NA	RPS4X
RSBN1	-2.1458921	1.19759535	0.52040198	NA	NA	RSBN1
RSF1	-2.0162309	1.31937541	0.52040198	NA	NA	RSF1
RYK	-2.1729138	1.28586957	0.52040198	NA	NA	RYK
S100A11	-2.1809246	1.37936479	0.52040198	NA	NA	S100A11
SAG	2.3658024	0.75387657	0.52040198	NA	NA	SAG
SAMD9	-1.962769	1.22384576	0.52040198	NA	NA	SAMD9
SDCCAG1	-2.0260314	1.17062402	0.52040198	NA	NA	SDCCAG1
SERPINB8	2.1704807	0.7920756	0.52040198	NA	NA	SERPINB8
SETD1A	-2.0067014	1.23141286	0.52040198	NA	NA	SETD1A
SHFM1	-2.0231735	1.21727741	0.52040198	NA	NA	SHFM1
SHROOM4	-2.1155196	1.19954957	0.52040198	NA	NA	SHROOM4
SLA	-2.0851739	1.25234599	0.52040198	NA	NA	SLA
SLC12A5	2.2462063	0.81191818	0.52040198	NA	NA	SLC12A5
SLC25A14	-2.1189443	1.20191495	0.52040198	NA	NA	SLC25A14
SLC31A2	-1.974387	1.32182763	0.52040198	NA	NA	SLC31A2
SLC39A10	2.2087944	0.83672299	0.52040198	NA	NA	SLC39A10
SLC4A7	-2.1984199	1.31180157	0.52040198	NA	NA	SLC4A7
SNORA57	2.2363773	0.75239695	0.52040198	NA	NA	SNORA57
SNRNP27	-2.0354463	1.18932417	0.52040198	NA	NA	SNRNP27
SOSTDC1	2.3612779	0.72786663	0.52040198	NA	NA	SOSTDC1
SPNS3	2.1222548	0.70307546	0.52040198	NA	NA	SPNS3
STAM	2.279432	0.84229943	0.52040198	NA	NA	STAM
STRN3	-2.1987479	1.27517342	0.52040198	NA	NA	STRN3
TAF13	-2.0862826	1.39718788	0.52040198	NA	NA	TAF13
TFAM	-2.2124488	1.29583364	0.52040198	NA	NA	TFAM
TFPI2	-1.9400506	1.14045646	0.52040198	NA	NA	TFPI2
TIMP3	-2.0849439	1.2966405	0.52040198	NA	NA	TIMP3
TJP1	-1.9522857	1.19967294	0.52040198	NA	NA	TJP1
TLR2	2.109616	0.7260496	0.52040198	NA	NA	TLR2
TLX3	2.0933695	0.74356216	0.52040198	NA	NA	TLX3
TMEM184C	-1.9276261	1.22844115	0.52040198	NA	NA	TMEM184C
TMEM220	-1.9798549	1.27831435	0.52040198	NA	NA	TMEM220
TMEM39A	-2.0776092	1.22157921	0.52040198	NA	NA	TMEM39A
TNIK	-1.9259299	1.24052773	0.52040198	NA	NA	TNIK
TOE1	-2.007117	1.23124864	0.52040198	NA	NA	TOE1
TOR1AIP1	-1.9418099	1.27535724	0.52040198	NA	NA	TOR1AIP1
TRPM7	-2.1178918	1.19694633	0.52040198	NA	NA	TRPM7
TSPAN3	-1.9084854	1.12209829	0.52040198	NA	NA	TSPAN3
TTC21A	2.1685433	0.81430899	0.52040198	NA	NA	TTC21A
TWIST1	-2.1784754	1.38715615	0.52040198	NA	NA	TWIST1
TWISTNB	-1.9593813	1.23898705	0.52040198	NA	NA	TWISTNB
UBE2N	-1.971055	1.15218407	0.52040198	NA	NA	UBE2N
UBQLN2	2.0551659	0.83870165	0.52040198	NA	NA	UBQLN2
UBXN7	-2.1792546	1.33298596	0.52040198	NA	NA	UBXN7
UTP23	-2.0043301	1.44435443	0.52040198	NA	NA	UTP23
WARS2	-1.9882524	1.16512945	0.52040198	NA	NA	WARS2
WDR12	-2.0742806	1.17417307	0.52040198	NA	NA	WDR12
WIT1	2.0830366	0.7403525	0.52040198	NA	NA	WIT1
YTHDF2	-2.1610003	1.14729333	0.52040198	NA	NA	YTHDF2
ZBTB25	2.4548182	0.67193386	0.52040198	NA	NA	ZBTB25
ZBTB8OS	-1.889675	1.37315545	0.52040198	NA	NA	ZBTB8OS
ZC3HAV1	-2.1859277	1.27529601	0.52040198	NA	NA	ZC3HAV1
ZCCHC2	-1.8957973	1.26380685	0.52040198	NA	NA	ZCCHC2

ZCCHC24	-2.0265927	1.24020817	0.52040198	NA	NA	ZCCHC24
ZFP106	-1.9961314	1.17420263	0.52040198	NA	NA	ZFP106
ZFP90	-1.9415611	1.17520105	0.52040198	NA	NA	ZFP90
ZIC1	-2.0479031	1.37387861	0.52040198	NA	NA	ZIC1
ZKSCAN1	-1.9608204	1.19686149	0.52040198	NA	NA	ZKSCAN1
ZMYM5	-1.9307096	1.27978372	0.52040198	NA	NA	ZMYM5
ZNF226	-1.9817975	1.24942263	0.52040198	NA	NA	ZNF226
ZNF461	-2.0113456	1.32811005	0.52040198	NA	NA	ZNF461
ZNF485	-1.8866719	1.31103526	0.52040198	NA	NA	ZNF485
ZNF567	-2.1117909	1.29733533	0.52040198	NA	NA	ZNF567
ZNF8	-1.9349992	1.18664382	0.52040198	NA	NA	ZNF8
PAFAH1B2	-1.8806275	1.17625486	0.52040198	NA	PAFAH1B2	PAFAH1B2
PCDH11X	2.1516949	0.84514434	0.52040198	NA	PCDH11X	PCDH11X
PCNXL2	2.0737151	0.79867141	0.52040198	NA	PCNXL2	PCNXL2
PEX5L	-2.1640812	1.24539707	0.52040198	NA	PEX5L	PEX5L
PREP	-1.984032	1.275789	0.52040198	NA	PREP	PREP
RARA	2.487077	0.76487883	0.52040198	NA	RARA	RARA
RLTPR	2.3650698	0.79681502	0.52040198	NA	RLTPR	RLTPR
ROBO3	2.3069616	0.74089878	0.52040198	NA	ROBO3	ROBO3
RUFY3	-1.9157856	1.19877545	0.52040198	NA	RUFY3	RUFY3
SCRN3	-2.102817	1.23760316	0.52040198	NA	SCRN3	SCRN3
SIPA1L1	-2.0250012	1.19199125	0.52040198	NA	SIPA1L1	SIPA1L1
SLC16A7	-2.1144529	1.4193732	0.52040198	NA	SLC16A7	SLC16A7
SLC29A3	2.3957502	0.77489056	0.52040198	NA	SLC29A3	SLC29A3
SS18	-1.9681358	1.22695495	0.52040198	NA	SS18	SS18
SUMF1	-2.1038277	1.21929102	0.52040198	NA	SUMF1	SUMF1
TFDP3	2.2307515	0.76327553	0.52040198	NA	TFDP3	TFDP3
TMCC1	-1.975072	1.16245899	0.52040198	NA	TMCC1	TMCC1
TMEM106B	-2.0505189	1.30611139	0.52040198	NA	TMEM106B	TMEM106B
TTC39B	-2.1480051	1.21699674	0.52040198	NA	TTC39B	TTC39B
VGLL3	-1.8927667	1.23418995	0.52040198	NA	VGLL3	VGLL3
VRK1	-2.0220907	1.19558435	0.52040198	NA	VRK1	VRK1
XIAP	-1.9443922	1.16880732	0.52040198	NA	XIAP	XIAP

MAR only 17vs19 q<0.01

SYMBOL	17vs19.d	17vs19.FC	17vs19.q	HAR	CAR	MAR
ACP6	4.0312994	0.65072026	0	NA	NA	ACP6
AMFR	-8.7147112	2.11275886	0	NA	NA	AMFR
ASAH2B	3.1456685	0.69224484	0	NA	NA	ASAH2B
ATG12	-2.7573778	1.34585972	0	NA	NA	ATG12
B4GALT5	-4.2003741	1.4674269	0	NA	NA	B4GALT5
BBX	-2.725413	1.50335278	0	NA	NA	BBX
BCL11B	-3.0643644	1.39138997	0	NA	NA	BCL11B
BEND3	-2.9661982	1.24150406	0	NA	NA	BEND3
BOC	-2.8068475	1.27656318	0	NA	NA	BOC
C12orf75	3.5206713	0.67780015	0	NA	NA	C12orf75
C14orf101	-3.3229101	1.38652116	0	NA	NA	C14orf101
C17orf75	-2.9644849	1.77741209	0	NA	NA	C17orf75
C17orf82	3.0510839	0.71348687	0	NA	NA	C17orf82
C18orf19	-3.0628472	1.35442251	0	NA	NA	C18orf19
C1orf156	-4.0044376	1.62573154	0	NA	NA	C1orf156
C20orf94	-2.8759556	1.3876158	0	NA	NA	C20orf94
C21orf57	7.4867579	0.47059106	0	NA	NA	C21orf57
C2orf74	-6.9874199	1.97745048	0	NA	NA	C2orf74

C3orf70	-3.7496884	1.67548683	0	NA	NA	C3orf70
C8orf44	-2.7352321	1.43097518	0	NA	NA	C8orf44
C8orf59	-2.7116814	1.29717691	0	NA	NA	C8orf59
CACNB4	-8.4974559	2.93779503	0	NA	NA	CACNB4
CCDC59	-3.0452841	1.3867423	0	NA	NA	CCDC59
CCND1	-4.7674023	1.57837942	0	NA	NA	CCND1
CCNJ	-3.0257177	1.38453169	0	NA	NA	CCNJ
CDH1	-3.9085644	1.52767902	0	NA	NA	CDH1
CDKAL1	-6.8380691	1.93235063	0	NA	NA	CDKAL1
CLDN11	-2.8676812	1.53788098	0	NA	NA	CLDN11
COL3A1	-3.5343766	1.53911674	0	NA	NA	COL3A1
CRBN	-4.4936872	1.31982838	0	NA	NA	CRBN
CRH	3.942527	0.52240886	0	NA	NA	CRH
DARC	4.2546692	0.60437945	0	NA	NA	DARC
DCN	-2.7933295	1.41786959	0	NA	NA	DCN
DDX17	-15.817588	9.77010381	0	NA	NA	DDX17
DEM1	-4.1108629	1.39193028	0	NA	NA	DEM1
DKK1	-3.6500275	1.68388797	0	NA	NA	DKK1
DOCK7	-5.7660623	1.62898159	0	NA	NA	DOCK7
DSP	-2.7089628	1.75689966	0	NA	NA	DSP
EGR1	-3.2586722	1.70147338	0	NA	NA	EGR1
EXT1	-2.7939417	1.33590226	0	NA	NA	EXT1
EYA1	-3.2278247	1.50514894	0	NA	NA	EYA1
FAM110B	-2.71985	1.25767091	0	NA	NA	FAM110B
FAM129A	-3.7403437	1.4648489	0	NA	NA	FAM129A
FAM178B	3.3666818	0.67561962	0	NA	NA	FAM178B
FAM46C	-6.0599677	2.33991468	0	NA	NA	FAM46C
FAM60A	-2.842808	1.5858925	0	NA	NA	FAM60A
FBLN5	-2.8513505	1.67224398	0	NA	NA	FBLN5
FLJ32063	3.4212591	0.74778534	0	NA	NA	FLJ32063
FOS	-3.3201611	2.17313695	0	NA	NA	FOS
FUT11	-3.36033	1.34288763	0	NA	NA	FUT11
FZD3	-4.9729828	2.70223052	0	NA	NA	FZD3
GAS2	-3.0464526	1.65391062	0	NA	NA	GAS2
GATC	-4.9569503	1.5386214	0	NA	NA	GATC
GJC1	-2.8228101	1.41752178	0	NA	NA	GJC1
GK3P	-3.041541	1.40839884	0	NA	NA	GK3P
GLCE	-3.297914	1.36107722	0	NA	NA	GLCE
GNG11	-4.6550523	1.60596159	0	NA	NA	GNG11
GNG2	3.3049392	0.67064371	0	NA	NA	GNG2
GOLGA1	-2.8694446	1.38471146	0	NA	NA	GOLGA1
GPR155	-2.9358654	1.32266431	0	NA	NA	GPR155
GRIN2B	-2.7893494	1.86405719	0	NA	NA	GRIN2B
GSTT1	14.567224	0.29536778	0	NA	NA	GSTT1
GYPA	-3.3398883	1.50730896	0	NA	NA	GYPA
HADHB	-3.5500149	1.30255086	0	NA	NA	HADHB
HAS3	-2.7170941	1.34573993	0	NA	NA	HAS3
HBB	-6.0753265	3.07303904	0	NA	NA	HBB
HBG1	-5.0919514	2.4485439	0	NA	NA	HBG1
HCG11	-2.6850125	1.46858808	0	NA	NA	HCG11
HEMGN	-3.7746287	2.04436448	0	NA	NA	HEMGN
HLA-DMA	4.852325	0.47355534	0	NA	NA	HLA-DMA
HMBOX1	9.4320641	0.24538978	0	NA	NA	HMBOX1
HNRNPA0	-2.8345414	1.2364776	0	NA	NA	HNRNPA0
HSPA2	-3.2032388	1.45747749	0	NA	NA	HSPA2
HTR2A	-8.5978236	3.18105929	0	NA	NA	HTR2A
IMMP1L	-3.1167623	1.36790945	0	NA	NA	IMMP1L
ITGB3BP	3.7077098	0.66980147	0	NA	NA	ITGB3BP

ITPRIP	-4.8085233	1.38297881	0	NA	NA	ITPRIP
KGFLP2	-10.258703	2.72238842	0	NA	NA	KGFLP2
KIF3A	-3.0737407	1.52727468	0	NA	NA	KIF3A
KLHDC8A	4.5112655	0.68928407	0	NA	NA	KLHDC8A
KPNB1	-2.7311651	1.28255063	0	NA	NA	KPNB1
LCLAT1	-4.0539244	1.40814788	0	NA	NA	LCLAT1
LMO7	-2.8636254	1.393642	0	NA	NA	LMO7
LOC100129387	-3.3914122	1.61354421	0	NA	NA	LOC10012938<br>7
LOC144486	-2.7714156	1.27120705	0	NA	NA	LOC144486
LOC221710	-2.9769436	1.70109375	0	NA	NA	LOC221710
LOC728554	3.1656053	0.78126995	0	NA	NA	LOC728554
LRCH1	-2.9817491	1.48754686	0	NA	NA	LRCH1
LRRC37A4	-3.3698976	1.68280214	0	NA	NA	LRRC37A4
LSM11	-3.2322088	1.71262628	0	NA	NA	LSM11
LSM2	-3.2913556	1.47098338	0	NA	NA	LSM2
LTF	2.9664019	0.72724447	0	NA	NA	LTF
LUM	-3.2260897	1.95091755	0	NA	NA	LUM
MAMDC2	-3.215659	1.43587342	0	NA	NA	MAMDC2
MDM4	3.0196712	0.78043155	0	NA	NA	MDM4
MED6	-3.3017157	1.47041287	0	NA	NA	MED6
METT10D	-2.7754442	1.3206454	0	NA	NA	METT10D
METTL10	3.7397373	0.69287372	0	NA	NA	METTL10
MFN1	-4.2921499	1.39514778	0	NA	NA	MFN1
MLH3	-3.3077975	1.27531588	0	NA	NA	MLH3
MRPS10	-2.8214155	1.36885454	0	NA	NA	MRPS10
MRPS16	-3.3590271	1.35298982	0	NA	NA	MRPS16
MYOCD	-3.082473	1.36241313	0	NA	NA	MYOCD
NAV2	-4.3314651	1.32226567	0	NA	NA	NAV2
NHSL1	-3.5147792	1.30160174	0	NA	NA	NHSL1
NID2	-3.5619483	1.75737721	0	NA	NA	NID2
NOV	-2.847625	1.70505348	0	NA	NA	NOV
OCLN	-3.4971851	1.51101582	0	NA	NA	OCLN
ODF1	4.6347296	0.55370102	0	NA	NA	ODF1
PCDHA10	-2.865924	1.36392247	0	NA	NA	PCDHA10
PCSK5	-3.0316199	1.39730482	0	NA	NA	PCSK5
PDGFC	-4.4359191	1.58063729	0	NA	NA	PDGFC
PDGFRL	-3.5086878	2.13452466	0	NA	NA	PDGFRL
PEX7	-2.8320448	1.4155305	0	NA	NA	PEX7
PFDN5	-4.0513292	1.36869283	0	NA	NA	PFDN5
PGCP	-2.6868551	1.3440617	0	NA	NA	PGCP
POLR1A	-3.0997891	1.34305056	0	NA	NA	POLR1A
POLR3E	-3.0437648	1.34474236	0	NA	NA	POLR3E
POMZP3	10.36362	0.3317839	0	NA	NA	POMZP3
PPIG	-3.1038205	1.48329993	0	NA	NA	PPIG
PROCR	-3.6552633	1.42352553	0	NA	NA	PROCR
PRSS35	-2.7653919	1.76424926	0	NA	NA	PRSS35
PSMB5	-3.5088104	1.32463521	0	NA	NA	PSMB5
PTN	-3.5390284	1.42543178	0	NA	NA	PTN
PTPRS	-3.3876319	1.37662535	0	NA	NA	PTPRS
RCL1	-3.8249932	1.39883248	0	NA	NA	RCL1
RCSD1	-2.889604	1.52154846	0	NA	NA	RCSD1
RHOBTB2	3.0460716	0.75270106	0	NA	NA	RHOBTB2
RPL14	-3.225496	1.3377925	0	NA	NA	RPL14
RPL27A	-4.8315189	1.57672313	0	NA	NA	RPL27A
RPL38	-3.7755266	1.3614783	0	NA	NA	RPL38
RPS23	5.6691597	0.65934831	0	NA	NA	RPS23
RPS4Y1	34.031146	0.01044102	0	NA	NA	RPS4Y1

RWDD2A	-2.843736	1.3185053	0	NA	NA	RWDD2A
SAR1A	-11.355276	3.05639008	0	NA	NA	SAR1A
SDC3	3.9075778	0.68806994	0	NA	NA	SDC3
SERINC3	-3.5661646	1.45310724	0	NA	NA	SERINC3
SERPINH1	-3.5150056	1.42815054	0	NA	NA	SERPINH1
SETD6	-3.1832995	1.57052209	0	NA	NA	SETD6
SLC20A2	-2.8595998	1.4429525	0	NA	NA	SLC20A2
SLC41A2	-3.4222912	1.39333258	0	NA	NA	SLC41A2
SNAP29	-6.3300364	1.64923889	0	NA	NA	SNAP29
SPARC	-3.0910308	1.34124545	0	NA	NA	SPARC
SPATA5	-3.2914178	1.4958473	0	NA	NA	SPATA5
TBX15	-3.2246332	1.53301564	0	NA	NA	TBX15
TERF1	-3.228146	1.340048	0	NA	NA	TERF1
TMEM100	-3.0007249	1.60591256	0	NA	NA	TMEM100
TMEM106C	-2.9400017	1.50954861	0	NA	NA	TMEM106C
TMEM159	-5.3443917	2.63754195	0	NA	NA	TMEM159
TMEM169	-2.7655447	1.25706554	0	NA	NA	TMEM169
TMEM178	-2.6904621	1.39217609	0	NA	NA	TMEM178
TMEM33	-2.6658069	1.35762943	0	NA	NA	TMEM33
TNNI3K	4.7634285	0.57583172	0	NA	NA	TNNI3K
TTC32	-6.837454	1.87150878	0	NA	NA	TTC32
TWSG1	-2.8596631	1.33741803	0	NA	NA	TWSG1
TXN2	-3.1741458	1.30516754	0	NA	NA	TXN2
UBE2H	-2.9120245	1.29370931	0	NA	NA	UBE2H
ULBP2	3.235594	0.65035909	0	NA	NA	ULBP2
UPF3A	-2.8879641	1.4299963	0	NA	NA	UPF3A
USP7	-3.4224056	1.48698682	0	NA	NA	USP7
USP9Y	24.930165	0.03392756	0	NA	NA	USP9Y
VGLL4	3.9162699	0.49989022	0	NA	NA	VGLL4
ZFPM2	-4.5503508	1.52778901	0	NA	NA	ZFPM2
ZNF281	-3.4402922	1.32854507	0	NA	NA	ZNF281
ZNF33B	3.2726387	0.53745074	0	NA	NA	ZNF33B
ZNF410	-2.9582169	1.30315211	0	NA	NA	ZNF410
ZNF673	-2.8295142	1.35068033	0	NA	NA	ZNF673
ZNF678	-7.0487526	3.15095782	0	NA	NA	ZNF678
ZNHIT6	-2.8517864	1.32338501	0	NA	NA	ZNHIT6
AACS	2.7772252	0.81928872	0.15408684	NA	NA	AACS
AASDH	-2.2837131	1.33839499	0.15408684	NA	NA	AASDH
AASS	-2.3766554	1.24422141	0.15408684	NA	NA	AASS
ADAMTS5	-2.5578755	1.32124927	0.15408684	NA	NA	ADAMTS5
ADORA2B	-2.6464446	1.52409042	0.15408684	NA	NA	ADORA2B
AKAP5	-2.4516203	1.43557392	0.15408684	NA	NA	AKAP5
ALKBH3	-2.4735768	1.26430568	0.15408684	NA	NA	ALKBH3
ANGPT2	-2.3641777	1.44208847	0.15408684	NA	NA	ANGPT2
ANKRD13A	-2.609523	1.27601125	0.15408684	NA	NA	ANKRD13A
ARFGEF2	-2.3904299	1.20561488	0.15408684	NA	NA	ARFGEF2
BPNT1	-2.6408925	1.2100756	0.15408684	NA	NA	BPNT1
BUB3	-2.4370156	1.20337506	0.15408684	NA	NA	BUB3
C10orf78	-2.3176742	1.3305325	0.15408684	NA	NA	C10orf78
C14orf118	-2.3531121	1.33016407	0.15408684	NA	NA	C14orf118
C21orf119	2.844505	0.67810524	0.15408684	NA	NA	C21orf119
C6orf52	2.7647041	0.73088546	0.15408684	NA	NA	C6orf52
CASC3	-2.6647051	1.30076511	0.15408684	NA	NA	CASC3
CAST	-2.4073902	1.23782379	0.15408684	NA	NA	CAST
CBX5	-2.3490675	1.38954567	0.15408684	NA	NA	CBX5
CCNT1	-2.4365363	1.58989758	0.15408684	NA	NA	CCNT1
CD302	-2.5345817	1.43298482	0.15408684	NA	NA	CD302
CLSTN2	-2.4636842	1.6788568	0.15408684	NA	NA	CLSTN2

CNIH4	-2.4647397	1.24346588	0.15408684	NA	NA	CNIH4
COL11A1	-2.4263028	1.58502373	0.15408684	NA	NA	COL11A1
COL1A2	-2.5247003	1.53757227	0.15408684	NA	NA	COL1A2
COL21A1	-2.2715263	1.39844564	0.15408684	NA	NA	COL21A1
CPEB3	-2.3418384	1.29947995	0.15408684	NA	NA	CPEB3
CRB1	2.7218365	0.77834041	0.15408684	NA	NA	CRB1
CSRNP3	-2.6324874	1.77680543	0.15408684	NA	NA	CSRNP3
CTSL1	-2.5466408	1.46599936	0.15408684	NA	NA	CTSL1
CYP1B1	-2.5728104	1.59423958	0.15408684	NA	NA	CYP1B1
DAPK1	2.8189297	0.76157288	0.15408684	NA	NA	DAPK1
DGAT2	2.9155871	0.76247906	0.15408684	NA	NA	DGAT2
DGKA	2.6749752	0.76261627	0.15408684	NA	NA	DGKA
DHRS11	-2.3061322	1.45353867	0.15408684	NA	NA	DHRS11
EFEMP1	-2.2601246	1.512079	0.15408684	NA	NA	EFEMP1
ENDOD1	-2.6652223	1.23841843	0.15408684	NA	NA	ENDOD1
ENTPD4	-2.2639859	1.26243617	0.15408684	NA	NA	ENTPD4
EPC1	-2.5084281	1.27380607	0.15408684	NA	NA	EPC1
ERCC8	-2.3758432	1.20989691	0.15408684	NA	NA	ERCC8
EXOSC2	-2.507613	1.19153459	0.15408684	NA	NA	EXOSC2
FBXW10	2.5525607	0.7559772	0.15408684	NA	NA	FBXW10
GABRB1	2.6685844	0.59317539	0.15408684	NA	NA	GABRB1
GALNT1	-2.5505422	1.2497617	0.15408684	NA	NA	GALNT1
GSK3B	-2.3125855	1.8209692	0.15408684	NA	NA	GSK3B
HHAT	-2.6642639	1.37203725	0.15408684	NA	NA	HHAT
HHLA3	2.5531852	0.84325886	0.15408684	NA	NA	HHLA3
ITIH2	-2.5172604	1.57287272	0.15408684	NA	NA	ITIH2
ITPRIPL2	-2.2714985	1.18882915	0.15408684	NA	NA	ITPRIPL2
KATNA1	-2.4099145	1.20178113	0.15408684	NA	NA	KATNA1
KCNG2	-2.3943031	1.31400163	0.15408684	NA	NA	KCNG2
KIAA1033	-2.5258399	1.4238757	0.15408684	NA	NA	KIAA1033
KIF21A	-2.5343021	1.31543011	0.15408684	NA	NA	KIF21A
KLHL5	-2.5304512	1.28779043	0.15408684	NA	NA	KLHL5
KRT75	2.5310171	0.83537088	0.15408684	NA	NA	KRT75
LDHAL6A	2.7683764	0.7212885	0.15408684	NA	NA	LDHAL6A
LOC253039	-2.2687965	1.31648364	0.15408684	NA	NA	LOC253039
LOC344595	-2.3850471	1.28620698	0.15408684	NA	NA	LOC344595
LYVE1	-2.2907557	1.9875599	0.15408684	NA	NA	LYVE1
MALAT1	-2.6059227	1.84262573	0.15408684	NA	NA	MALAT1
MAML3	-2.2695581	1.18278914	0.15408684	NA	NA	MAML3
MAN1A1	-2.3485979	1.47403349	0.15408684	NA	NA	MAN1A1
MARS2	-2.5238092	1.38801805	0.15408684	NA	NA	MARS2
MED1	-2.6381891	1.36884522	0.15408684	NA	NA	MED1
MMGT1	-2.3779571	1.23820151	0.15408684	NA	NA	MMGT1
MRC2	-2.6443084	1.27734229	0.15408684	NA	NA	MRC2
NCAPG	-2.3366229	1.38654065	0.15408684	NA	NA	NCAPG
NEK10	-2.2733071	1.3437932	0.15408684	NA	NA	NEK10
NEK11	2.6425471	0.83268837	0.15408684	NA	NA	NEK11
OLFML3	-2.5775999	1.68013647	0.15408684	NA	NA	OLFML3
PBLD	-2.3296979	1.38976503	0.15408684	NA	NA	PBLD
PEX3	-2.3367843	1.24747703	0.15408684	NA	NA	PEX3
PHAX	-2.6002356	1.26474595	0.15408684	NA	NA	PHAX
PIGF	-2.5599996	1.22772039	0.15408684	NA	NA	PIGF
PITRM1	2.7774431	0.78930237	0.15408684	NA	NA	PITRM1
PNOC	2.5737188	0.75599796	0.15408684	NA	NA	PNOC
PNPLA8	-2.3946008	1.24409588	0.15408684	NA	NA	PNPLA8
PPIB	-2.3549659	1.28657741	0.15408684	NA	NA	PPIB
PRMT8	-2.3041096	1.2286679	0.15408684	NA	NA	PRMT8
PRPF39	-2.3193488	1.47045554	0.15408684	NA	NA	PRPF39

PTCD3	-2.3290937	1.20928246	0.15408684	NA	NA	PTCD3
RASA2	-2.3064891	1.37965524	0.15408684	NA	NA	RASA2
RBMS3	-2.5382434	1.17434759	0.15408684	NA	NA	RBMS3
RPGRIP1L	-2.2612711	1.2759607	0.15408684	NA	NA	RPGRIP1L
RPP25	2.9209582	0.75889774	0.15408684	NA	NA	RPP25
SCARNA2	2.7205174	0.69920881	0.15408684	NA	NA	SCARNA2
SEC31A	-2.4401949	1.27613832	0.15408684	NA	NA	SEC31A
SENP8	-2.3818281	1.17602863	0.15408684	NA	NA	SENP8
SFRS12IP1	-2.3118187	1.28184605	0.15408684	NA	NA	SFRS12IP1
SIAH3	-2.5901526	1.8345651	0.15408684	NA	NA	SIAH3
SLC35B3	-2.2959573	1.31271787	0.15408684	NA	NA	SLC35B3
SMURF2	-2.263931	1.37802813	0.15408684	NA	NA	SMURF2
SOAT2	2.5770608	0.73759794	0.15408684	NA	NA	SOAT2
SRL	-2.5935564	1.28325817	0.15408684	NA	NA	SRL
TAOK1	-2.4294969	1.56100579	0.15408684	NA	NA	TAOK1
TCL1A	2.7836999	0.79808427	0.15408684	NA	NA	TCL1A
TRAPPC5	-2.4614767	1.30937375	0.15408684	NA	NA	TRAPPC5
TRIM69	-2.3625574	1.46343493	0.15408684	NA	NA	TRIM69
TUBB6	-2.3299468	1.45598407	0.15408684	NA	NA	TUBB6
UHRF1	-2.5674501	1.3798915	0.15408684	NA	NA	UHRF1
VDAC3	-2.4883873	1.21200174	0.15408684	NA	NA	VDAC3
VPS29	-2.4892411	1.22026069	0.15408684	NA	NA	VPS29
WDFY1	-2.3148448	1.31487986	0.15408684	NA	NA	WDFY1
ZCCHC6	-2.4770813	1.23305412	0.15408684	NA	NA	ZCCHC6
ZEB1	-2.4675301	1.41869147	0.15408684	NA	NA	ZEB1
ZIC2	-2.5628214	1.55072803	0.15408684	NA	NA	ZIC2
ZNF2	-2.3970363	1.41933831	0.15408684	NA	NA	ZNF2
ZNF568	-2.5273151	1.28010059	0.15408684	NA	NA	ZNF568
ZNF646	-2.3111948	1.33040038	0.15408684	NA	NA	ZNF646
ZNF827	-2.4277283	1.4249109	0.15408684	NA	NA	ZNF827
ABCC8	2.4328791	0.76046742	0.52040198	NA	NA	ABCC8
ADK	-2.1302733	1.31924955	0.52040198	NA	NA	ADK
AHI1	-1.9220662	1.18209537	0.52040198	NA	NA	AHI1
ANKRD42	2.2749257	0.82264758	0.52040198	NA	NA	ANKRD42
ANP32A	-2.0033225	1.22966807	0.52040198	NA	NA	ANP32A
ANXA2	-1.9997924	1.45329543	0.52040198	NA	NA	ANXA2
APOD	2.2826264	0.73716617	0.52040198	NA	NA	APOD
ARFIP1	-2.1744556	1.30306661	0.52040198	NA	NA	ARFIP1
ARID2	-2.1601992	1.33696186	0.52040198	NA	NA	ARID2
ATPAF2	-2.0593733	1.23084647	0.52040198	NA	NA	ATPAF2
BAG3	-1.9384694	1.31468397	0.52040198	NA	NA	BAG3
BCAS1	-2.084448	1.20803661	0.52040198	NA	NA	BCAS1
BFSP2	2.1901177	0.82295316	0.52040198	NA	NA	BFSP2
BMP2K	-1.9532441	1.16821622	0.52040198	NA	NA	BMP2K
BMP8A	2.1058632	0.73990316	0.52040198	NA	NA	BMP8A
BOK	2.3721644	0.76793487	0.52040198	NA	NA	BOK
BRAP	-2.1352878	1.2534199	0.52040198	NA	NA	BRAP
C11orf57	-2.0509347	1.18894566	0.52040198	NA	NA	C11orf57
C15orf61	-1.9751662	1.23347746	0.52040198	NA	NA	C15orf61
C17orf86	-2.0339511	1.26658073	0.52040198	NA	NA	C17orf86
C1orf27	-1.974642	1.30984575	0.52040198	NA	NA	C1orf27
C21orf49	2.4976927	0.79183242	0.52040198	NA	NA	C21orf49
C2orf42	-1.8927023	1.20108286	0.52040198	NA	NA	C2orf42
C3orf57	-1.8893237	1.19816256	0.52040198	NA	NA	C3orf57
C7orf51	-2.1071945	1.33640561	0.52040198	NA	NA	C7orf51
CACNG2	2.4539067	0.79018397	0.52040198	NA	NA	CACNG2
CAMK2N1	-2.068048	1.26749433	0.52040198	NA	NA	CAMK2N1
CAPN8	2.062243	0.73588244	0.52040198	NA	NA	CAPN8

CC2D2B	2.3528731	0.65284613	0.52040198	NA	NA	CC2D2B
CCDC125	-2.1515828	1.23519787	0.52040198	NA	NA	CCDC125
CCDC140	2.1053809	0.80331706	0.52040198	NA	NA	CCDC140
CCDC28A	-2.1309696	1.26417516	0.52040198	NA	NA	CCDC28A
CECR1	2.2627415	0.76025114	0.52040198	NA	NA	CECR1
CEP110	-2.1125622	1.19484619	0.52040198	NA	NA	CEP110
CHEK2	2.114179	0.78587628	0.52040198	NA	NA	CHEK2
CLDN16	2.3310751	0.79347685	0.52040198	NA	NA	CLDN16
COL9A2	2.1659016	0.70015242	0.52040198	NA	NA	COL9A2
COX15	-1.9051802	1.2085638	0.52040198	NA	NA	COX15
CPXM2	2.1325825	0.86026774	0.52040198	NA	NA	CPXM2
CST7	2.3033608	0.78005281	0.52040198	NA	NA	CST7
CTSC	-2.1849962	1.37608816	0.52040198	NA	NA	CTSC
CTTNBP2	-2.1819356	1.25458024	0.52040198	NA	NA	CTTNBP2
CXorf38	-2.0975988	1.39567315	0.52040198	NA	NA	CXorf38
CXorf59	2.0504571	0.78277683	0.52040198	NA	NA	CXorf59
DCST1	2.1424342	0.71034187	0.52040198	NA	NA	DCST1
DEGS1	-2.072779	1.1858773	0.52040198	NA	NA	DEGS1
DHTKD1	-2.0802506	1.22301036	0.52040198	NA	NA	DHTKD1
DNAH7	2.41911	0.70773173	0.52040198	NA	NA	DNAH7
DNAJC5G	2.0911647	0.71262761	0.52040198	NA	NA	DNAJC5G
EFHB	-1.9809965	1.37724656	0.52040198	NA	NA	EFHB
EIF2C3	-2.0435392	1.34298354	0.52040198	NA	NA	EIF2C3
EIF3G	-2.2129915	1.18242727	0.52040198	NA	NA	EIF3G
EIF5B	-2.0811152	1.23309634	0.52040198	NA	NA	EIF5B
EMILIN3	2.2222533	0.73846615	0.52040198	NA	NA	EMILIN3
ERAP2	-2.1155597	1.16759146	0.52040198	NA	NA	ERAP2
ERP44	-2.024124	1.18241014	0.52040198	NA	NA	ERP44
FAM122A	-1.9726457	1.17223446	0.52040198	NA	NA	FAM122A
FAM131C	2.1503249	0.82193538	0.52040198	NA	NA	FAM131C
FAM150B	2.0544744	0.70944555	0.52040198	NA	NA	FAM150B
FAM186B	2.1947687	0.78534665	0.52040198	NA	NA	FAM186B
FAM35A	-2.1911292	1.24569496	0.52040198	NA	NA	FAM35A
FARSB	-2.0363338	1.38382939	0.52040198	NA	NA	FARSB
FBN1	-2.2223297	1.35000795	0.52040198	NA	NA	FBN1
FER	-2.0464847	1.36356216	0.52040198	NA	NA	FER
FGL2	-2.0653139	1.59204968	0.52040198	NA	NA	FGL2
GABRB2	2.371876	0.68809537	0.52040198	NA	NA	GABRB2
GAR1	-2.1861567	1.23255098	0.52040198	NA	NA	GAR1
GATAD1	-2.0920612	1.22377274	0.52040198	NA	NA	GATAD1
GEMIN8	-2.1429039	1.22834499	0.52040198	NA	NA	GEMIN8
GHRHR	2.0826463	0.74904975	0.52040198	NA	NA	GHRHR
GIMAP2	-2.0266119	1.33596833	0.52040198	NA	NA	GIMAP2
GLT8D2	-1.8940871	1.25220948	0.52040198	NA	NA	GLT8D2
GLTP	-1.9432979	1.25181329	0.52040198	NA	NA	GLTP
GNAL	-2.0080944	1.86789974	0.52040198	NA	NA	GNAL
GNG10	-2.0686584	1.27356848	0.52040198	NA	NA	GNG10
GNPDA1	-2.0278047	1.20704513	0.52040198	NA	NA	GNPDA1
GNPNAT1	-2.125466	1.17206348	0.52040198	NA	NA	GNPNAT1
GPC1	2.1876605	0.78278178	0.52040198	NA	NA	GPC1
GPR83	-1.9193755	1.41579111	0.52040198	NA	NA	GPR83
GPR89B	-2.0351575	1.17692447	0.52040198	NA	NA	GPR89B
GRB7	2.1377094	0.7432128	0.52040198	NA	NA	GRB7
GSTO2	2.2568222	0.69059817	0.52040198	NA	NA	GSTO2
GTF2H1	-1.8994516	1.16105061	0.52040198	NA	NA	GTF2H1
HBP1	-1.9735302	1.26538923	0.52040198	NA	NA	HBP1
HEATR5A	-1.902767	1.34983554	0.52040198	NA	NA	HEATR5A
HIBADH	-1.9814384	1.21161046	0.52040198	NA	NA	HIBADH

HINT3	-1.9441384	1.60393052	0.52040198	NA	NA	HINT3
HMGCR	2.0532203	0.86415929	0.52040198	NA	NA	HMGCR
HSD11B2	2.2925167	0.72482175	0.52040198	NA	NA	HSD11B2
HTATIP2	2.0700273	0.79864545	0.52040198	NA	NA	HTATIP2
INTS9	-2.0402097	1.16961044	0.52040198	NA	NA	INTS9
ISLR2	-1.9758955	1.33074964	0.52040198	NA	NA	ISLR2
ISOC1	2.4713551	0.77933133	0.52040198	NA	NA	ISOC1
KCTD16	2.354395	0.60252427	0.52040198	NA	NA	KCTD16
KIAA0090	-2.1808647	1.23180295	0.52040198	NA	NA	KIAA0090
KIAA1586	-2.1571613	1.28117498	0.52040198	NA	NA	KIAA1586
KIF5A	-2.0342757	1.35590469	0.52040198	NA	NA	KIF5A
KRT74	2.1239248	0.77422916	0.52040198	NA	NA	KRT74
KRT82	2.4586007	0.73755047	0.52040198	NA	NA	KRT82
KTELC1	-2.1984993	1.29330049	0.52040198	NA	NA	KTELC1
LAPTM4B	-1.9216933	1.34113928	0.52040198	NA	NA	LAPTM4B
LHB	-2.0833171	1.28891523	0.52040198	NA	NA	LHB
LLPH	-1.999817	1.1937156	0.52040198	NA	NA	LLPH
LMX1A	2.4550773	0.77172307	0.52040198	NA	NA	LMX1A
LOC144438	-2.0132641	1.26333967	0.52040198	NA	NA	LOC144438
LOC153328	2.4023878	0.77768044	0.52040198	NA	NA	LOC153328
LOC283174	-2.1237479	1.27073276	0.52040198	NA	NA	LOC283174
LOC340357	2.3057571	0.73874396	0.52040198	NA	NA	LOC340357
LOC374491	-1.9806138	1.2798857	0.52040198	NA	NA	LOC374491
LRRC17	-1.9927652	1.27770681	0.52040198	NA	NA	LRRC17
LRRC37A2	2.1760499	0.82621777	0.52040198	NA	NA	LRRC37A2
LRRC57	-2.0589527	1.30361128	0.52040198	NA	NA	LRRC57
LSG1	-1.8904563	1.20921704	0.52040198	NA	NA	LSG1
MAP3K12	2.1973903	0.81938356	0.52040198	NA	NA	MAP3K12
MAPK3	2.0914245	0.85292565	0.52040198	NA	NA	MAPK3
MAS1	2.0962005	0.72382428	0.52040198	NA	NA	MAS1
MB	2.27274	0.71881396	0.52040198	NA	NA	MB
MCC	-2.1187613	1.29070636	0.52040198	NA	NA	MCC
MFSD6L	2.2174655	0.7702194	0.52040198	NA	NA	MFSD6L
MMP16	-1.9949701	1.25948912	0.52040198	NA	NA	MMP16
MRC1	-2.0059435	2.02300477	0.52040198	NA	NA	MRC1
MRPL2	-1.902676	1.21328046	0.52040198	NA	NA	MRPL2
MSL2	-1.9960172	1.29359364	0.52040198	NA	NA	MSL2
MTERF	-2.1988882	1.20543362	0.52040198	NA	NA	MTERF
MYH10	-1.9686168	1.30885144	0.52040198	NA	NA	MYH10
NCDN	2.4662698	0.81990466	0.52040198	NA	NA	NCDN
NDC80	-2.0028732	1.29395268	0.52040198	NA	NA	NDC80
NDUFS5	2.2017389	0.83524493	0.52040198	NA	NA	NDUFS5
NLN	-2.1851909	1.1969421	0.52040198	NA	NA	NLN
NOSTRIN	-2.1178711	1.29669248	0.52040198	NA	NA	NOSTRIN
NSL1	-2.0645267	1.2104069	0.52040198	NA	NA	NSL1
OMD	-2.2479875	1.87282089	0.52040198	NA	NA	OMD
ORMDL2	-1.9860724	1.19784337	0.52040198	NA	NA	ORMDL2
PAIP2B	2.1305932	0.79107342	0.52040198	NA	NA	PAIP2B
PAPSS2	-2.2111151	1.2575037	0.52040198	NA	NA	PAPSS2
PCDH8	2.4071771	0.71515048	0.52040198	NA	NA	PCDH8
PCDHGA1	2.307887	0.79195605	0.52040198	NA	NA	PCDHGA1
PCGF6	-1.885943	1.27052744	0.52040198	NA	NA	PCGF6
PCLO	2.1715754	0.80619704	0.52040198	NA	NA	PCLO
PCSK6	2.4986726	0.84049685	0.52040198	NA	NA	PCSK6
PDE1A	-2.2262416	1.28671827	0.52040198	NA	NA	PDE1A
PEX26	-2.1140121	1.21066874	0.52040198	NA	NA	PEX26
PGM3	-2.0435103	1.26859256	0.52040198	NA	NA	PGM3
PLAG1	-1.9965254	1.30066752	0.52040198	NA	NA	PLAG1

PON1	2.0833775	0.77710618	0.52040198	NA	NA	PON1
POP5	-2.0889906	1.233827	0.52040198	NA	NA	POP5
POU2F2	-1.9716952	1.24277587	0.52040198	NA	NA	POU2F2
PPM1D	-2.141691	1.23000435	0.52040198	NA	NA	PPM1D
PPPDE1	-2.2219879	1.26451279	0.52040198	NA	NA	PPPDE1
PRIMA1	2.2221399	0.74154802	0.52040198	NA	NA	PRIMA1
PRRX1	-2.2172145	1.3321327	0.52040198	NA	NA	PRRX1
PSAPL1	2.1745227	0.79341248	0.52040198	NA	NA	PSAPL1
PSD3	2.261628	0.7178862	0.52040198	NA	NA	PSD3
PSIP1	-1.991087	1.24723292	0.52040198	NA	NA	PSIP1
QTRTD1	-1.9218234	1.17645254	0.52040198	NA	NA	QTRTD1
R3HDM2	-1.8986794	1.27911761	0.52040198	NA	NA	R3HDM2
RAI14	-1.9661168	1.3050944	0.52040198	NA	NA	RAI14
RANBP3L	-1.8840796	1.21065335	0.52040198	NA	NA	RANBP3L
RBKS	-1.9427115	1.24363587	0.52040198	NA	NA	RBKS
RBM27	-1.8993699	1.21577946	0.52040198	NA	NA	RBM27
RBMS1	-2.1281534	1.34581448	0.52040198	NA	NA	RBMS1
RDX	-2.0118485	1.22780659	0.52040198	NA	NA	RDX
REG3A	2.1035545	0.83289156	0.52040198	NA	NA	REG3A
RGS22	2.1723	0.76006672	0.52040198	NA	NA	RGS22
RNF115	-2.2382884	1.31117258	0.52040198	NA	NA	RNF115
ROD1	-2.0930953	1.32272881	0.52040198	NA	NA	ROD1
RPP14	-2.1327202	1.32992645	0.52040198	NA	NA	RPP14
RPS4X	-2.1992759	1.1435872	0.52040198	NA	NA	RPS4X
RSBN1	-2.1458921	1.19759535	0.52040198	NA	NA	RSBN1
RSF1	-2.0162309	1.31937541	0.52040198	NA	NA	RSF1
RYK	-2.1729138	1.28586957	0.52040198	NA	NA	RYK
S100A11	-2.1809246	1.37936479	0.52040198	NA	NA	S100A11
SAG	2.3658024	0.75387657	0.52040198	NA	NA	SAG
SAMD9	-1.962769	1.22384576	0.52040198	NA	NA	SAMD9
SDCCAG1	-2.0260314	1.17062402	0.52040198	NA	NA	SDCCAG1
SERPINB8	2.1704807	0.7920756	0.52040198	NA	NA	SERPINB8
SETD1A	-2.0067014	1.23141286	0.52040198	NA	NA	SETD1A
SHFM1	-2.0231735	1.21727741	0.52040198	NA	NA	SHFM1
SHROOM4	-2.1155196	1.19954957	0.52040198	NA	NA	SHROOM4
SLA	-2.0851739	1.25234599	0.52040198	NA	NA	SLA
SLC12A5	2.2462063	0.81191818	0.52040198	NA	NA	SLC12A5
SLC25A14	-2.1189443	1.20191495	0.52040198	NA	NA	SLC25A14
SLC31A2	-1.974387	1.32182763	0.52040198	NA	NA	SLC31A2
SLC39A10	2.2087944	0.83672299	0.52040198	NA	NA	SLC39A10
SLC4A7	-2.1984199	1.31180157	0.52040198	NA	NA	SLC4A7
SNORA57	2.2363773	0.75239695	0.52040198	NA	NA	SNORA57
SNRNP27	-2.0354463	1.18932417	0.52040198	NA	NA	SNRNP27
SOSTDC1	2.3612779	0.72786663	0.52040198	NA	NA	SOSTDC1
SPNS3	2.1222548	0.70307546	0.52040198	NA	NA	SPNS3
STAM	2.279432	0.84229943	0.52040198	NA	NA	STAM
STRN3	-2.1987479	1.27517342	0.52040198	NA	NA	STRN3
TAF13	-2.0862826	1.39718788	0.52040198	NA	NA	TAF13
TFAM	-2.2124488	1.29583364	0.52040198	NA	NA	TFAM
TFPI2	-1.9400506	1.14045646	0.52040198	NA	NA	TFPI2
TIMP3	-2.0849439	1.2966405	0.52040198	NA	NA	TIMP3
TJP1	-1.9522857	1.19967294	0.52040198	NA	NA	TJP1
TLR2	2.109616	0.7260496	0.52040198	NA	NA	TLR2
TLX3	2.0933695	0.74356216	0.52040198	NA	NA	TLX3
TMEM184C	-1.9276261	1.22844115	0.52040198	NA	NA	TMEM184C
TMEM220	-1.9798549	1.27831435	0.52040198	NA	NA	TMEM220
TMEM39A	-2.0776092	1.22157921	0.52040198	NA	NA	TMEM39A
TNIK	-1.9259299	1.24052773	0.52040198	NA	NA	TNIK

TOE1	-2.007117	1.23124864	0.52040198	NA	NA	TOE1
TOR1AIP1	-1.9418099	1.27535724	0.52040198	NA	NA	TOR1AIP1
TRPM7	-2.1178918	1.19694633	0.52040198	NA	NA	TRPM7
TSPAN3	-1.9084854	1.12209829	0.52040198	NA	NA	TSPAN3
TTC21A	2.1685433	0.81430899	0.52040198	NA	NA	TTC21A
TWIST1	-2.1784754	1.38715615	0.52040198	NA	NA	TWIST1
TWISTNB	-1.9593813	1.23898705	0.52040198	NA	NA	TWISTNB
UBE2N	-1.971055	1.15218407	0.52040198	NA	NA	UBE2N
UBQLN2	2.0551659	0.83870165	0.52040198	NA	NA	UBQLN2
UBXN7	-2.1792546	1.33298596	0.52040198	NA	NA	UBXN7
UTP23	-2.0043301	1.44435443	0.52040198	NA	NA	UTP23
WARS2	-1.9882524	1.16512945	0.52040198	NA	NA	WARS2
WDR12	-2.0742806	1.17417307	0.52040198	NA	NA	WDR12
WIT1	2.0830366	0.7403525	0.52040198	NA	NA	WIT1
YTHDF2	-2.1610003	1.14729333	0.52040198	NA	NA	YTHDF2
ZBTB25	2.4548182	0.67193386	0.52040198	NA	NA	ZBTB25
ZBTB8OS	-1.889675	1.37315545	0.52040198	NA	NA	ZBTB8OS
ZC3HAV1	-2.1859277	1.27529601	0.52040198	NA	NA	ZC3HAV1
ZCCHC2	-1.8957973	1.26380685	0.52040198	NA	NA	ZCCHC2
ZCCHC24	-2.0265927	1.24020817	0.52040198	NA	NA	ZCCHC24
ZFP106	-1.9961314	1.17420263	0.52040198	NA	NA	ZFP106
ZFP90	-1.9415611	1.17520105	0.52040198	NA	NA	ZFP90
ZIC1	-2.0479031	1.37387861	0.52040198	NA	NA	ZIC1
ZKSCAN1	-1.9608204	1.19686149	0.52040198	NA	NA	ZKSCAN1
ZMYM5	-1.9307096	1.27978372	0.52040198	NA	NA	ZMYM5
ZNF226	-1.9817975	1.24942263	0.52040198	NA	NA	ZNF226
ZNF461	-2.0113456	1.32811005	0.52040198	NA	NA	ZNF461
ZNF485	-1.8866719	1.31103526	0.52040198	NA	NA	ZNF485
ZNF567	-2.1117909	1.29733533	0.52040198	NA	NA	ZNF567
ZNF8	-1.9349992	1.18664382	0.52040198	NA	NA	ZNF8

Supplementary table 3

Genes Nearby a TFBS with a significant turnover

SYMBOL	TF	PFOvsPT.q	PFOvsPT.FC	17vs19.q	17vs19.FC
**"CA10"**	MA0089.1.NFE2L1::MafG|MA0122.1.Nkx3-2|MA0068.1.Pax4|MA0033.1.FOXL1|MA0154.1.EBF1|MA0032.1.FOXC1|MA0027.1.En1	**0**	5.712212733	**37.305898**	0.680951788
**"COL12A1"**	MA0033.1.FOXL1|MA0006.1.Arnt::**Ahr**	**0**	1.458176067	**45.966351**	1.074272474
**"FLJ41649"**	MA0009.1.T|MA0037.1.GATA3	**0**	0.18001333	**50.945784**	1.127531418
**"MDGA1"**	MA0114.1.HNF4A	**0**	1.470491648	**24.919109**	0.881719509
**"NFE2L3"**	MA0073.1.RREB1	**0**	0.457712017	**24.919109**	0.742823848
**"OCA2"**	MA0037.1.GATA3|MA0056.1.MZF1_1-4|MA0073.1.RREB1|MA0079.2.SP1|MA0105.1.NFKB1|MA0057.1.MZF1_5-13|MA0068.1.Pax4	**0**	0.339901245	**53.049636**	1.045841429
**"PDE4DIP"**	MA0156.1.FEV|MA0055.1.Myf	**0**	0.692919758	**37.305898**	0.91679345
**"PTPN13"**	MA0035.2.Gata1|MA0050.1.IRF1|MA0051.1.**IRF2**|MA0037.1.GATA3|MA0063.1.Nkx2-5|MA0067.1.Pax2|MA0055.1.**Myf**|MA0018.2.CREB1|MA0111.1.Spz1|MA0117.1.Mafb|MA0133.1.BRCA1|MA0100.1.**Myb**|MA0089.1.NFE2L1::MafG	**0**	0.527643875	**24.919109**	0.84712375
**"SORCS1"**	MA0046.1.HNF1A	**0**	0.484518816	**45.966351**	1.142124228
**"WBSCR17"**	MA0146.1.Zfx	**0**	1.542744782	**53.049636**	1.01216026
**"ELAVL2"**	MA0163.1.PLAG1	**0.1132952**	1.499451152	**14.05416**	1.244444473

**"LPHN3"**	MA0028.1.ELK1|MA0088.1.znf143|MA0090.1.TEAD1|MA0136.1.ELF5|MA0149.1.EWSR1-FLI1|MA0081.1.SPIB|MA0079.2.SP1	**0.1920624**	1.287259152	**50.945784**	0.989684833
**"NLGN1"**	MA0115.1.NR1H2::RXRA	**0.1920624**	0.70393173	**45.966351**	1.05921267
**"PARP8"**	MA0162.1.Egr1|MA0006.1.Arnt::**Ahr**|MA0042.1.FOXI1	**0.1920624**	1.453057443	**37.305898**	0.912318071
**"RPH3A"**	MA0042.1.FOXI1|MA0073.1.RREB1|MA0084.1.SRY	**0.1920624**	1.750384294	**5.7461843**	0.650129372
**"XYLT1"**	MA0151.1.ARID3A	**0.1920624**	0.80021924	**45.966351**	0.972022235
**"ZNF438"**	MA0073.1.RREB1|MA0006.1.Arnt::**Ahr**|MA0042.1.FOXI1|MA0068.1.Pax4|MA0162.1.Egr1	**0.1920624**	0.790438068	**50.945784**	1.025841266
**"ZNF488"**	MA0061.1.NF-kappaB|MA0068.1.Pax4|MA0073.1.RREB1|MA0107.1.RELA|MA0133.1.BRCA1|MA0142.1.Pou5f1|MA0158.1.HOXA5	**0.5692172**	0.69467208	**5.7461843**	0.769449566

**"ANKRD18A**	MA0002.2.RUNX1|MA0028.1.ELK1|MA0027.1.En1|MA0063.1.Nkx2-5|MA0014.1.Pax5|MA0017.1.**NR2F1**|MA	**0.8841996**	0.700121216	**14.05416**	0.80894258
**"EFNA5"**	MA0070.1.**PBX1**|MA0038.1.Gfi	**0.8841996**	0.805562936	**45.966351**	1.057912259
**"LOC440570**	MA0019.1.Ddit3::Cebpa|MA0122.1.Nkx3-2	**0.8841996**	1.523432007	**1.6684098**	1.442728927
**"RSPO3"**	MA0091.1.TAL1::TCF3	**0.8841996**	1.282867266	**14.05416**	1.174306977
"FAM36A"	MA0073.1.RREB1	**2.3573161**	0.852819971	**53.049636**	1.00508389
"FAT3"	MA0052.1.MEF2A	**2.3573161**	0.79278441	**45.966351**	1.056480815
"NFIB"	MA0162.1.Egr1	**2.3573161**	0.736933547	**1.6684098**	1.352237816
"NIPBL"	MA0092.1.Hand1::Tcfe2a	**5.5878899**	0.745246255	**1.6684098**	1.366650823
"PDE4D"	MA0061.1.NF-kappaB|MA0113.1.**NR3C1**|MA0164.1.Nr2e3|MA0101.1.REL|MA0107.1.RELA	**5.5878899**	1.211239512	**45.966351**	1.057117174
"SYT3"	MA0027.1.En1|MA0062.2.GABPA	**5.5878899**	1.223613257	**24.919109**	0.919808153
"ATP2B4"	MA0137.2.STAT1	**10.827679**	0.858145422	**53.049636**	1.003120724

"NUBPL"	MA0024.1.E2F1|MA0031.1.FOXD1|MA0122.1.Nkx3-2|MA0124.1.NKX3-1|MA0063.1.Nkx2-5|MA0103.1.ZEB1|MA0145.1.Tcfcp2l1	**10.827679**	0.875619426	**45.966351**	0.973167718
"SHANK2"	MA0066.1.PPARG	**10.827679**	0.868594144	**24.919109**	0.928273354
"TAC1"	MA0006.1.Arnt::**Ahr**|MA0162.1.Egr1	**10.827679**	1.395738793	**24.919109**	1.19542118
"ZCCHC11"	MA0061.1.NF-kappaB|MA0105.1.NFKB1	**10.827679**	0.805590514	**5.7461843**	1.231333928
"ANXA2"	MA0073.1.RREB1	**16.669199**	1.285928472	**0.520402**	1.453295433
"IGSF9"	MA0162.1.Egr1|MA0037.1.GATA3	**16.669199**	1.28268069	**0.520402**	0.686088877
"MSRA"	MA0042.1.FOXI1|MA0162.1.Egr1|MA0006.1.Arnt::**Ahr**|MA0037.1.GATA3	**16.669199**	0.819260057	**1.6684098**	1.280609447
"PTRF"	MA0068.1.Pax4|MA0101.1.REL|MA0119.1.TLX1::NFIC|MA0161.1.NFIC	**16.669199**	1.136054582	**24.919109**	1.096944237
"SALL3"	MA0137.2.STAT1|MA0144.1.Stat3	**16.669199**	1.14900178	**24.919109**	0.928252714
"SLIT3"	MA0042.1.FOXI1|MA0006.1.Arnt::**Ahr**|MA0079.2.SP1|MA0162.1.Egr1	**16.669199**	1.14686011	**45.966351**	0.975503032
"TRPS1"	MA0070.1.**PBX1**	**16.669199**	0.882009935	**53.049636**	1.005130483
"ADORA2B"	MA0058.1.MAX|MA0036.1.GATA2|MA0056.1.MZF1_1-4	**24.934882**	1.239787852	**0.1540868**	1.524090418
"CCDC101"	MA0056.1.MZF1_1-4	**24.934882**	1.133362534	**24.919109**	1.085245461
"ISX"	MA0070.1.**PBX1**	**24.934882**	1.173648551	**45.966351**	0.958390386
"LYRM5"	MA0006.1.Arnt::**Ahr**|MA0042.1.FOXI1|MA0259.1.HIF1A::ARNT|MA0073.1.RREB1|MA0162.1.Egr1	**24.934882**	0.886598396	**45.966351**	0.983837695
"MON1B"	MA0052.1.MEF2A	**24.934882**	1.120449736	**37.305898**	0.948907644
"MUC2"	MA0033.1.FOXL1|MA0048.1.NHLH1|MA0002.2.RUNX1|MA0091.1.TAL1::TCF3|MA0109.1.Hltf|MA0111.1.Spz1|MA0116.1.Zfp423|MA0130.1.ZNF354C|MA0133.1.BRCA1|MA0154.1.EBF1|MA0163.1.PLAG1|MA0055.1.Myf|MA0050.1.IRF1	**24.934882**	1.140321231	**14.05416**	0.879418624

"NRP1"	MA0024.1.E2F1|MA0028.1.ELK1|MA0035.2.Gata1|MA0073.1.RREB1|MA0079.2.SP1|MA0080.2.SPI1|MA0037.1.GATA3|MA0098.1.ETS1|MA0136.1.ELF5|MA0149.1.EWSR1-FLI1|MA0081.1.SPIB|MA0014.1.Pax5|MA0442.1.SOX10|MA0152.1.NFATC2|MA0156.1.FEV	**24.934882**	1.169871902	**5.7461843**	0.833834891
"ODZ2"	MA0105.1.NFKB1	**24.934882**	0.865604228	**50.945784**	0.995839252
"PLXNA4"	MA0073.1.RREB1	**24.934882**	0.816235773	**1.6684098**	1.326671121
"TBC1D2B"	MA0029.1.Evi1	**24.934882**	1.098241784	**37.305898**	0.95475188
"ATP8B3"	MA0056.1.MZF1_1-4|MA0149.1.EWSR1-FLI1	**35.800528**	1.110581209	**24.919109**	0.933824721
"CDH6"	MA0073.1.RREB1|MA0041.1.Foxd3|MA0068.1.Pax4	**35.800528**	1.109747131	**37.305898**	1.055872685
"COPS4"	MA0061.1.NF-kappaB	**35.800528**	1.062116264	**53.049636**	1.003464593
"DSCR3"	MA0098.1.ETS1|MA0130.1.ZNF354C|MA0095.1.YY1|MA0070.1.**PBX**1|MA0069.1.Pax6	**35.800528**	1.082491502	**24.919109**	1.077327951
"HLA-DRB4"	MA0061.1.NF-kappaB	**35.800528**	1.107078032	**5.7461843**	0.845651436
"HTT"	MA0072.1.RORA_2|MA0065.2.PPARG::RXRA|MA0043.1.**HLF**|MA0152.1.NFATC2|MA0059.1.MYC::MAX|MA0098.1.ETS1|MA0151.1.ARID3A	**35.800528**	0.892065091	**24.919109**	0.916263387
"IGLV4-60"	MA0039.2.Klf4	**35.800528**	0.895473292	**24.919109**	1.103378096
"ITGB5"	MA0041.1.Foxd3|MA0042.1.FOXI1|MA0146.1.Zfx	**35.800528**	1.099186866	**24.919109**	0.948160345
"LGR6"	MA0068.1.Pax4	**35.800528**	1.093999929	**14.05416**	1.108708334
"LOC645166"	MA0002.2.RUNX1|MA0061.1.NF-kappaB|MA0105.1.NFKB1|MA0107.1.RELA|MA0081.1.SPIB|MA0101.1.REL|MA0162.1.Egr1	**35.800528**	1.148773544	**0**	0.704168146
"MBP"	MA0147.1.Myc	**35.800528**	1.090236767	**50.945784**	1.022693683
"MYO10"	MA0105.1.NFKB1	**35.800528**	1.107035439	**24.919109**	1.100168026

"PUS7L"	MA0046.1.HNF1A	**35.800528**	0.914512663	**53.049636**	1.005902807
"RHCG"	MA0083.1.SRF	**35.800528**	1.096496599	**37.305898**	1.054226013
"TUSC5"	MA0091.1.TAL1::TCF3	**35.800528**	1.197800122	**45.966351**	1.075037446
"WSB1"	MA0106.1.TP53	**35.800528**	0.840343285	**0.1540868**	1.389657773
"ZNF100"	MA0048.1.NHLH1|MA0055.1.Myf|MA0058.1.MAX|MA0079.2.SP1|MA0093.1.USF1|MA0057.1.MZF1_5-13|MA0113.1.**NR3C**1|MA0130.1.ZNF354C|MA0147.1.Myc|MA0100.1.**Myb**|MA0004.1.Arnt|MA0039.2.Klf4|MA0160.1.**NR4A2**	**35.800528**	1.094009682	**37.305898**	1.045941503
"ATP6V1E1"	MA0025.1.NFIL3	**49.792314**	1.045345697	**24.919109**	1.080822353
"C2orf37"	MA0144.1.Stat3	**49.792314**	0.901416142	**37.305898**	1.077171322
"C5orf42"	MA0042.1.FOXI1|MA0073.1.RREB1|MA0006.1.Arnt::**Ahr**|MA0162.1.Egr1	**49.792314**	1.065263289	**45.966351**	1.039777428
"CHD1L"	MA0061.1.NF-kappaB|MA0067.1.Pax2|MA0093.1.USF1|MA0098.1.ETS1|MA0081.1.SPIB|MA0122.1.Nkx3-2|MA0068.1.Pax4|MA0004.1.Arnt|MA0019.1.Ddit3::Cebpa|MA0101.1.REL	**49.792314**	1.086234334	**1.6684098**	0.847156212

"COL3A1"	MA0032.1.FOXC1|MA0036.1.GATA2|MA0031.1.FOXD1|MA0048.1.NHLH1|MA0050.1.IRF1|MA0037.1.GATA3|MA0040.1.Foxq1|MA0056.1.MZF1_1-4|MA0014.1.Pax5|MA0027.1.En1|MA0028.1.ELK1|MA0077.1.SOX9|MA0078.1.Sox17|MA0079.2.SP1|MA0080.2.SPI1|MA0081.1.SPIB|MA0084.1.SRY|MA0088.1.znf143|MA0051.1.**IRF2**|MA0055.1.Myf|MA0095.1.YY1|MA0098.1.ETS1|MA0099.2.AP1|MA0101.1.REL|MA0102.2.CEBPA|MA0109.1.Hltf|MA0111.1.Spz1|MA0113.1.**NR3C1**|MA0114.1.HNF4A|MA0117.1.Mafb|MA0122.1.Nkx3-2|MA0089.1.NFE2L1::MafG|MA0092.1.Hand1::Tcfe2a|MA0132.1.Pdx1|MA0136.1.ELF5|MA0140.1.Tal1::Gata1|MA0150.1.NFE2L2|MA0151.1.ARID3A|MA0152.1.NFATC2|MA0153.1.HNF1B|MA0156.1.FEV|MA0157.1.FOXO3|MA0158.1.HOXA5|MA0161.1.NFIC|MA0163.1.PLAG1|MA0442.1.SOX10|MA0124.1.NKX3-1|MA0130.1.ZNF354C|MA0057.1.MZF1_5-13|MA0063.1.Nkx2-5|MA0068.1.Pax4	**49.792314**	1.139581552	**0**	1.539116742
"DCUN1D4"	MA0099.2.AP1|MA0135.1.Lhx3|MA0028.1.ELK1|MA0076.1.ELK4	**49.792314**	1.06131661	**5.7461843**	1.186087636

<table>
<tr><td>"DMRT2"</td><td>MA0035.2.Gata1|MA0048.1.NHLH1|MA0063.1.Nkx2-5|MA0065.2.PPARG::RXRA|MA0056.1.MZF1_1-4|MA0090.1.TEAD1|MA0092.1.Hand1::Tcfe2a|MA0055.1.Myf|MA0027.1.En1|MA0028.1.ELK1|MA0111.1.Spz1|MA0112.2.ESR1|MA0113.1.NR3C1|MA0117.1.Mafb|MA0125.1.Nobox|MA0130.1.ZNF354C|MA0144.1.Stat3|MA0152.1.NFATC2|MA0157.1.FOXO3|MA0161.1.NFIC|MA0095.1.YY1|MA0101.1.REL|MA0107.1.RELA|MA0084.1.SRY|MA0074.1.RXRA::VDR|MA0442.1.SOX10</td><td>49.792314</td><td>1.049091326</td><td>50.945784</td><td>0.993366826</td></tr>
<tr><td>"FHIT"</td><td>MA0105.1.NFKB1</td><td>49.792314</td><td>1.107902438</td><td>14.05416</td><td>0.8747493</td></tr>
<tr><td>"hCG_181549</td><td>MA0071.1.RORA_1</td><td>49.792314</td><td>1.053749103</td><td>0.520402</td><td>1.20043505</td></tr>
<tr><td>"KIAA1715"</td><td>MA0033.1.FOXL1</td><td>49.792314</td><td>0.926223105</td><td>45.966351</td><td>0.980166021</td></tr>
<tr><td>"LOC1001306</td><td>MA0009.1.T|MA0141.1.Esrrb|MA0068.1.Pax4</td><td>49.792314</td><td>1.063926475</td><td>14.05416</td><td>1.13275678</td></tr>
<tr><td>"LOC144817"</td><td>MA0149.1.EWSR1-FLI1|MA0079.2.SP1|MA0442.1.SOX10|MA0056.1.MZF1_1-4</td><td>49.792314</td><td>1.063289835</td><td>24.919109</td><td>0.910141042</td></tr>
<tr><td>"LOC441601"</td><td>MA0032.1.FOXC1|MA0061.1.NF-kappaB|MA0132.1.Pdx1|MA0151.1.ARID3A|MA0101.1.REL|MA0117.1.Mafb|MA0158.1.HOXA5|MA0154.1.EBF1</td><td>49.792314</td><td>1.064816985</td><td>37.305898</td><td>0.966912799</td></tr>
<tr><td>"LOC641365"</td><td>MA0048.1.NHLH1|MA0259.1.HIF1A::ARNT</td><td>49.792314</td><td>1.039187998</td><td>37.305898</td><td>1.045880841</td></tr>
<tr><td>"LOC642891"</td><td>MA0145.1.Tcfcp2l1</td><td>49.792314</td><td>1.097120793</td><td>5.7461843</td><td>0.858612661</td></tr>
</table>

"LOC727924"	MA0003.1.TFAP2A|MA0006.1.Arnt::**Ahr**|MA0057.1.MZF1_5-13|MA0079.2.SP1|MA0055.1.Myf|MA0056.1.MZF1_1-4|MA0119.1.TLX1::NFIC|MA0154.1.EBF1	**49.792314**	1.036972888	**37.305898**	0.975907897
"LOC730227"	MA0147.1.Myc	**49.792314**	1.095822	**24.919109**	0.925437955
"METTL9"	MA0057.1.MZF1_5-13|MA0067.1.Pax2|MA0056.1.MZF1_1-4|MA0098.1.ETS1|MA0105.1.NFKB1|MA0153.1.HNF1B|MA0159.1.RXR::RAR_DR5|MA0079.2.SP1|MA0080.2.SPI1	**49.792314**	1.075624895	**24.919109**	1.071118153
"MEX3B"	MA0104.2.Mycn|MA0147.1.Myc|MA0055.1.Myf|MA0073.1.RREB1	**49.792314**	1.045492222	**53.049636**	1.007175268
"MPV17L"	MA0162.1.Egr1|MA0163.1.PLAG1|MA0070.1**.PBX1**	**49.792314**	1.13925159	**0.1540868**	0.712569502
"NUDT7"	MA0033.1.FOXL1	**49.792314**	1.063829689	**45.966351**	0.975405278
"PLA2R1"	MA0073.1.RREB1|MA0079.2.SP1	**49.792314**	1.061783382	**24.919109**	1.06154859

"SCN8A"	MA0027.1.En1|MA0028.1.ELK1|MA0029.1.Evi1|MA0033.1.FOXL1|MA0035.2.Gata1|MA0036.1.GATA2|MA0037.1.GATA3|MA0038.1.Gfi|MA0043.1.**HLF**|MA0002.2.RUNX1|MA0003.1.TFAP2A|MA0004.1.Arnt|MA0018.2.CREB1|MA0060.1.NFYA|MA0063.1.Nkx2-5|MA0067.1.Pax2|MA0070.1.**PBX1**|MA0078.1.Sox17|MA0079.2.SP1|MA0080.2.SPI1|MA0081.1.SPIB|MA0088.1.znf143|MA0089.1.NFE2L1::MafG|MA0091.1.TAL1::TCF3|MA0092.1.Hand1::Tcfe2a|MA0093.1.USF1|MA0095.1.YY1|MA0098.1.ETS1|MA0099.2.AP1|MA0101.1.REL|MA0103.1.ZEB1|MA0105.1.NFKB1|MA0109.1.Hltf|MA0113.1.N**R3C1**|MA0117.1.Mafb	**49.792314**	1.085185448	**14.05416**	1.117350925
"SLC37A1"	MA0024.1.E2F1|MA0027.1.En1|MA0056.1.MZF1_1-4|MA0099.2.AP1|MA0103.1.ZEB1|MA0442.1.SOX10	**49.792314**	1.053929711	**24.919109**	0.942341098
"USP14"	MA0137.2.STAT1|MA0004.1.Arnt|MA0059.1.MYC::MAX|MA0147.1.Myc	**49.792314**	1.054509557	**24.919109**	0.948909134

"XKR6"	MA0080.2.SPI1	**49.792314**	0.927455909	**53.049636**	1.002082659
"ZMAT4"	MA0006.1.Arnt::**Ahr**|MA0042.1.FOXI1|MA0073.1.RREB1|MA0162.1.Egr1	**49.792314**	1.077372706	**45.966351**	1.043303652
"ADAM23"	MA0042.1.FOXI1|MA0025.1.NFIL3	**65.861377**	0.933311212	**37.305898**	0.959019738
"ATAD3A"	MA0046.1.HNF1A	**65.861377**	1.015793416	**14.05416**	1.098051269
"C3orf21"	MA0067.1.Pax2|MA0014.1.Pax5|MA0083.1.SRF|MA0003.1.TFAP2A|MA0159.1.RXR::RAR_DR5	**65.861377**	0.935826814	**45.966351**	0.986184057
"C7orf50"	MA0041.1.Foxd3|MA0033.1.FOXL1	**65.861377**	1.018505404	**24.919109**	1.072816705
"DKFZp686A1	MA0032.1.FOXC1|MA0089.1.NFE2L1::MafG	**65.861377**	1.017742932	**45.966351**	1.040190303
"EDAR"	MA0122.1.Nkx3-2	**65.861377**	1.018132462	**5.7461843**	0.863113338
"FBXO31"	MA0035.2.Gata1|MA0027.1.En1|MA0095.1.YY1|MA0073.1.RREB1|MA0056.1.MZF1_1-4|MA0117.1.Mafb|MA0037.1.GATA3|MA0140.1.Tal1::Gata1	**65.861377**	1.031467052	**37.305898**	0.970439589
"LOC150185"	MA0004.1.Arnt|MA0014.1.Pax5|MA0055.1.Myf|MA0093.1.USF1|MA0117.1.Mafb|MA0048.1.NHLH1|MA0133.1.BRCA1	**65.861377**	1.027498862	**37.305898**	0.959650186
"LOC284600"	MA0030.1.FOXF2|MA0056.1.MZF1_1-4|MA0145.1.Tcfcp2l1	**65.861377**	0.925975539	**50.945784**	0.984463923
"LOC654342"	MA0090.1.TEAD1|MA0098.1.ETS1|MA0061.1.NF-kappaB|MA0107.1.RELA|MA0101.1.REL|MA0105.1.NFKB1	**65.861377**	1.084894353	**0**	0.612496714
"MARCKS"	MA0061.1.NF-kappaB	**65.861377**	1.013841402	**5.7461843**	1.099476716
"MGC21881"	MA0163.1.PLAG1	**65.861377**	0.929597089	**37.305898**	0.950666181
"NEDD4L"	MA0106.1.TP53|MA0133.1.BRCA1	**65.861377**	0.920046483	**14.05416**	1.122532397
"NPPA"	MA0159.1.RXR::RAR_DR5	**65.861377**	1.044390019	**14.05416**	1.157277378
"PDLIM5"	MA0036.1.GATA2	**65.861377**	1.028157573	**50.945784**	1.017874716

"POR"	MA0003.1.TFAP2A|MA0014.1.Pax5|MA0028.1.ELK1|MA0032.1.FOXC1|MA0018.2.CREB1|MA0027.1.En1|MA0048.1.NHLH1|MA0055.1.Myf|MA0070.1.**PBX1**|MA0071.1.RORA_1|MA0076.1.ELK4|MA0077.1.**SOX9**|MA0078.1.Sox17|MA0079.2.SP1|MA0080.2.SPI1|MA0084.1.SRY|MA0088.1.znf143|MA0036.1.GATA2|MA0037.1.GATA3|MA0095.1.YY1|MA0098.1.ETS1|MA0099.2.AP1|MA0103.1.ZEB1|MA0111.1.Spz1|MA0117.1.Mafb|MA0130.1.ZNF354C|MA0136.1.ELF5|MA0144.1.Stat3|MA0151.1.ARID3A|MA0156.1.FEV|MA0158.1.HOXA5|MA0160.1.**NR4A2**|MA0161.1.NFIC|MA0164.1.Nr2e3|MA0258.1.ESR2|MA0442.1.SOX10|MA0089.1.NFE2L1::MafG|MA0092.1.Hand1::Tcfe2a	**65.861377**	1.019253777	**14.05416**	0.923088367
"PPIF"	MA0017.1.**NR2F1**	**65.861377**	1.017673579	**14.05416**	1.077351821
"psiTPTE22"	MA0146.1.Zfx|MA0052.1.MEF2A	**65.861377**	1.031886304	**24.919109**	1.059314368
"RNF168"	MA0144.1.Stat3	**65.861377**	0.915974226	**5.7461843**	1.17139959
"SLC30A8"	MA0061.1.NF-kappaB|MA0101.1.REL|MA0107.1.RELA	**65.861377**	1.028761192	**24.919109**	0.939272783
"SSH2"	MA0014.1.Pax5|MA0057.1.MZF1_5-13|MA0068.1.Pax4|MA0079.2.SP1|MA0088.1.znf143|MA0119.1.TLX1::NFIC	**65.861377**	1.024659158	**24.919109**	0.950527889
"THRA"	MA0048.1.NHLH1	**65.861377**	1.018271543	**50.945784**	1.013312408

"TUBGCP5"	MA0003.1.TFAP2A|MA0055.1.Myf|MA0037.1.GATA3|MA0056.1.MZF1_1-4|MA0073.1.RREB1|MA0079.2.SP1|MA0093.1.USF1|MA0112.2.ESR1|MA0117.1.Mafb|MA0119.1.TLX1::NFIC|MA0057.1.MZF1_5-13|MA0068.1.Pax4|MA0130.1.ZNF354C|MA0133.1.BRCA1|MA0154.1.EBF1|MA0160.1.**NR4A2**|MA0161.1.NFIC	**65.861377**	1.030435126	**37.305898**	1.05016892
"ZNF518B"	MA0163.1.PLAG1|MA0004.1.Arnt|MA0079.2.SP1	**65.861377**	0.9408506	**45.966351**	0.985818401
"ZNF70"	MA0018.2.CREB1|MA0027.1.En1|MA0032.1.FOXC1|MA0063.1.Nkx2-5|MA0087.1.Sox5|MA0124.1.NKX3-1|MA0157.1.FOXO3	**65.861377**	1.045008635	**1.6684098**	1.168172922
"AGAP1"	MA0006.1.Arnt::**Ahr**|MA0102.2.CEBPA|MA0073.1.RREB1|MA0033.1.FOXL1|MA0042.1.FOXI1|MA0162.1.Egr1	**69.16944**	0.909800848	**1.6684098**	1.300646832
"AGL"	MA0098.1.ETS1|MA0080.2.SPI1|MA0442.1.SOX10|MA0028.1.ELK1|MA0156.1.FEV|MA0136.1.ELF5|MA0149.1.EWSR1-FLI1	**69.16944**	0.941091438	**24.919109**	0.929074054
"CALN1"	MA0116.1.Zfp423	**69.16944**	0.932667166	**24.919109**	1.096819749
"CAPN2"	MA0052.1.MEF2A	**69.16944**	0.953979742	**24.919109**	0.953968055
"DENND3"	MA0095.1.YY1|MA0070.1.**PBX1**|MA0014.1.Pax5|MA0036.1.GATA2|MA0130.1.ZNF354C|MA0098.1.ETS1	**69.16944**	0.944989858	**5.7461843**	0.895625291
"hCG_179509	MA0083.1.SRF	**69.16944**	1.013144688	**50.945784**	0.987464275

"LOC375010"	MA0031.1.FOXD1|MA0033.1.FOXL1|MA0063.1.Nkx2-5|MA0078.1.Sox17|MA0052.1.MEF2A|MA0061.1.NF-kappaB|MA0101.1.REL|MA0105.1.NFKB1|MA0083.1.SRF|MA0084.1.SRY|MA0157.1.FOXO3|MA0158.1.HOXA5|MA0442.1.SOX10|MA0107.1.RELA|MA0133.1.BRCA1	**69.16944**	0.941954529	**24.919109**	0.93868914
"MAPK8"	MA0024.1.E2F1	**69.16944**	1.00930392	**24.919109**	0.935437682
"MCPH1"	MA0018.2.CREB1|MA0025.1.NFIL3|MA0004.1.Arnt|MA0033.1.FOXL1|MA0037.1.GATA3|MA0027.1.En1|MA0032.1.FOXC1|MA0056.1.MZF1_1-4|MA0059.1.MYC::MAX|MA0038.1.Gfi|MA0043.1**.HLF**|MA0093.1.USF1|MA0099.2.AP1|MA0101.1.REL|MA0102.2.CEBPA|MA0113.1.**NR3C1**|MA0114.1.HNF4A|MA0117.1.Mafb|MA0119.1.TLX1::NFIC|MA0124.1.NKX3-1|MA0125.1.Nobox|MA0130.1.ZNF354C|MA0063.1.Nkx2-5|MA0092.1.Hand1::Tcfe2a|MA0142.1.Pou5f1|MA0151.1.ARID3A|MA0152.1.NFATC2|MA0153.1.HNF1B|MA0158.1.HOXA5|MA0160.1.N**R4A2**|MA0164.1.Nr2e3|MA0132.1.Pdx1|MA0133.1.BRCA1	**69.16944**	1.007788775	**0.520402**	1.181917204

"PRNP"	MA0018.2.CREB1|MA0019.1.Ddit3::Cebpa|MA0014.1.Pax5|MA0028.1.ELK1|MA0033.1.FOXL1|MA0025.1.NFIL3|MA0027.1.En1|MA0042.1.FOXI1|MA0043.1.**HLF**|MA0063.1.Nkx2-5|MA0067.1.Pax2|MA0068.1.Pax4|MA0070.1.**PBX1**|MA0071.1.RORA_1|MA0074.1.RXRA::VDR|MA0077.1.**SOX9**|MA0078.1.Sox17|MA0079.2.SP1|MA0036.1.GATA2|MA0040.1.Foxq1|MA0087.1.Sox5|MA0088.1.znf143|MA0089.1.NFE2L1::MafG|MA0095.1.YY1|MA0098.1.ETS1|MA0099.2.AP1|MA0102.2.CEBPA|MA0113.1.**NR3C1**|MA0117.1.Mafb|MA0132.1.Pdx1|MA0133.1.BRCA1|MA0135.1.Lhx3|MA0142.1.Pou5f1|MA0143.1.Sox2|MA0150.1.NFE2L2|MA0151.1.ARID3A|MA0152.1.NFATC2|MA0153.1.HNF1B|MA0160.1.**NR4A2**|MA0161.1.NFIC|MA0442.1.SOX10|MA0081.1.SPIB|MA0084.1.SRY	**69.16944**	1.012830772	**14.05416**	0.9087468
"RCN2"	MA0146.1.Zfx|MA0024.1.E2F1|MA0055.1.Myf|MA0056.1.MZF1_1-4|MA0079.2.SP1	**69.16944**	1.006142169	**50.945784**	1.018051886
"SLAMF1"	MA0066.1.PPARG	**69.16944**	0.935797391	**14.05416**	0.88709559
ACSM1	MA0003.1.TFAP2A|MA0043.1.**HLF**|MA0040.1.Foxq1|MA0066.1.PPARG|MA0079.2.SP1|MA0081.1.SPIB|MA0084.1.SRY|MA0119.1.TLX1::NFIC|MA0143.1.Sox2|MA0144.1.Stat3|MA0160.1.**NR4A2**|MA0442.1.SOX10|MA0046.1.HNF1A|MA0058.1.MAX|MA0063.1.Nkx2-5|MA0037.1.GATA3|MA0024.1.E2F1	**69.16944**	1.001224525	**37.305898**	0.961188733
"CEP250"	MA0057.1.MZF1_5-13|MA0079.2.SP1	**70.946791**	0.961001827	**53.049636**	1.007770142

"DGKA"	MA0164.1.Nr2e3	**70.946791**	0.933839729	**0.1540868**	0.762616274
"KIF6"	MA0042.1.FOXI1	**70.946791**	0.951758235	**37.305898**	0.948546717
"SLAIN2"	MA0042.1.FOXI1|MA0030.1.FOXF2|MA0259.1.HIF1A::ARNT	**70.946791**	0.938253022	**24.919109**	1.085528605
"SSH1"	MA0036.1.GATA2|MA0039.2.Klf4|MA0057.1.MZF1_5-13|MA0070.1.**PBX1**|MA0073.1.RREB1|MA0162.1.Egr1|MA0095.1.YY1|MA0098.1.ETS1|MA0130.1.ZNF354C	**70.946791**	0.958757592	**37.305898**	1.038010457
"ZNF541"	MA0068.1.Pax4|MA0076.1.ELK4	**70.946791**	0.948489514	**53.049636**	1.01067928
"ETFDH"	MA0106.1.TP53	**72.891972**	0.947081839	**5.7461843**	1.168097378
"FLCN"	MA0083.1.SRF	**72.891972**	0.951691103	**1.6684098**	1.152586779
"TTC5"	MA0003.1.TFAP2A	**72.891972**	0.938019334	**0**	1.346374531
"ANXA10"	MA0042.1.FOXI1	**73.134386**	0.987256466	**5.7461843**	0.870504858
"ATXN10"	MA0130.1.ZNF354C	**73.134386**	0.978762545	**24.919109**	1.047946515
"BAGE"	MA0137.2.STAT1|MA0070.1.**PBX1**|MA0144.1.Stat3	**73.134386**	0.977709828	**14.05416**	0.930723627
"BCL8"	MA0164.1.Nr2e3|MA0079.2.SP1|MA0105.1.NFKB1	**73.134386**	0.972547876	**24.919109**	0.95204528
"C18orf2"	MA0033.1.FOXL1	**73.134386**	0.995745947	**50.945784**	1.014392455
"CRYL1"	MA0003.1.TFAP2A|MA0163.1.PLAG1|MA0130.1.ZNF354C|MA0067.1.Pax2	**73.134386**	0.928496076	**0.1540868**	0.674396604
"DYNLRB2"	MA0091.1.TAL1::TCF3|MA0033.1.FOXL1|MA0007.1.Ar|MA0025.1.NFIL3	**73.134386**	0.982066384	**37.305898**	1.063531189
"FAM100A"	MA0073.1.RREB1|MA0162.1.Egr1|MA0042.1.FOXI1|MA0006.1.Arnt::**Ahr**	**73.134386**	0.984554055	**50.945784**	0.994877753
"GNAO1"	MA0073.1.RREB1|MA0162.1.Egr1|MA0006.1.Arnt::**Ahr**|MA0042.1.FOXI1	**73.134386**	0.987758173	**53.049636**	1.00342142
"HMGA1"	MA0105.1.NFKB1|MA0068.1.Pax4|MA0101.1.REL|MA0061.1.NF-kappaB	**73.134386**	0.994923342	**24.919109**	0.947282222
"KCNJ14"	MA0155.1.INSM1|MA0092.1.Hand1::Tcfe2a|MA0068.1.Pax4	**73.134386**	0.983589611	**53.049636**	1.001286072
"KCNN4"	MA0116.1.Zfp423	**73.134386**	0.953192036	**5.7461843**	0.857885966
"LOC1001315	MA0069.1.Pax6	**73.134386**	0.989041616	**53.049636**	1.006068094

"LOC283788"	MA0089.1.NFE2L1::MafG|MA0147.1.Myc|MA0059.1.MYC::MAX	**73.134386**	0.951713191	**0**	0.394165531
"LOC339166"	MA0014.1.Pax5|MA0028.1.ELK1|MA0098.1.ETS1|MA0136.1.ELF5|MA0079.2.SP1|MA0080.2.SPI1|MA0442.1.SOX10|MA0152.1.NFATC2|MA0156.1.FEV	**73.134386**	0.991772691	**37.305898**	0.955651083
"LPXN"	MA0119.1.TLX1::NFIC	**73.134386**	0.96588317	**5.7461843**	1.134561478
"MARVELD3"	MA0079.2.SP1	**73.134386**	0.988961913	**14.05416**	1.092247016
"MGC34800"	MA0055.1.Myf|MA0139.1.CTCF|MA0018.2.CREB1|MA0141.1.Esrrb	**73.134386**	0.947862957	**1.6684098**	0.823619181
"MTMR15"	MA0115.1.NR1H2::RXRA	**73.134386**	0.977121567	**14.05416**	0.930626148
"NKD2"	MA0018.2.CREB1|MA0033.1.FOXL1|MA0036.1.GATA2|MA0042.1.FOXI1|MA0068.1.Pax4|MA0073.1.RREB1|MA0040.1.Foxq1|MA0151.1.ARID3A|MA0162.1.Egr1|MA0095.1.YY1|MA0006.1.Arnt::**Ahr**	**73.134386**	0.975974173	**37.305898**	1.038438157
"PLD5"	MA0130.1.ZNF354C	**73.134386**	0.94445617	**14.05416**	1.180436091
"PSMF1"	MA0024.1.E2F1|MA0059.1.MYC::MAX|MA0102.2.CEBPA	**73.134386**	0.992857539	**14.05416**	0.908578915
"PSPH"	MA0055.1.Myf|MA0067.1.Pax2|MA0083.1.SRF|MA0164.1.Nr2e3	**73.134386**	0.980648276	**1.6684098**	1.123787055
"SUPT4H1"	MA0125.1.Nobox	**73.134386**	0.964916112	**24.919109**	1.060557954

"TARSL2"	MA0004.1.Arnt|MA0006.1.Arnt::**Ahr**|MA0018.2.CREB1|MA0007.1.Ar|MA0028.1.ELK1|MA0029.1.Evi1|MA0019.1.Ddit3::Cebpa|MA0032.1.FOXC1|MA0038.1.Gfi|MA0039.2.Klf4|MA0041.1.Foxd3|MA0048.1.NHLH1|MA0055.1.Myf|MA0058.1.MAX|MA0059.1.MYC::MAX|MA0031.1.FOXD1|MA0067.1.Pax2|MA0069.1.Pax6|MA0071.1.RORA_1|MA0076.1.ELK4|MA0079.2.SP1|MA0090.1.TEAD1|MA0093.1.USF1|MA0104.2.Mycn|MA0063.1.Nkx2-5|MA0002.2.RUNX1|MA0158.1.HOXA5|MA0162.1.Egr1|MA0150.1.NFE2L2|MA0152.1.NFATC2|MA0122.1.Nkx3-2	**73.134386**	0.969381375	**0.1540868**	0.801423138
"TMPRSS13"	MA0073.1.RREB1|MA0105.1.NFKB1|MA0116.1.Zfp423|MA0149.1.EWSR1-FLI1|MA0163.1.PLAG1	**73.134386**	0.980495614	**45.966351**	0.975040011
"TNIP1"	MA0101.1.REL	**73.134386**	0.979340195	**24.919109**	0.941296001
"UBE4B"	MA0068.1.Pax4|MA0073.1.RREB1|MA0079.2.SP1	**73.134386**	0.965198154	**0.520402**	1.22973899

"USP22"	MA0002.2.RUNX1|MA0003.1.TFAP2A|MA0006.1.Arnt::**Ahr**|MA0018.2.CREB1|MA0027.1.En1|MA0036.1.GATA2|MA0038.1.Gfi|MA0063.1.Nkx2-5|MA0067.1.Pax2|MA0068.1.Pax4|MA0070.1.**PBX1**|MA0073.1.RREB1|MA0078.1.Sox17|MA0079.2.SP1|MA0081.1.SPIB|MA0084.1.SRY|MA0040.1.Foxq1|MA0056.1.MZF1_1-4|MA0057.1.MZF1_5-13|MA0061.1.NF-kappaB|MA0092.1.Hand1::Tcfe2a|MA0095.1.YY1|MA0098.1.ETS1|MA0099.2.AP1|MA0100.1.**Myb**|MA0101.1.REL|MA0102.2.CEBPA|MA0105.1.NFKB1|MA0107.1.RELA|MA0108.2.TBP|MA0109.1.Hltf|MA0111.1.Spz1|MA0113.1.**NR3C1**|MA0130.1.ZNF354C|MA0133.1.BRCA1|MA0135.1.Lhx3|MA0136.1.ELF5|MA0151.1.ARID3A|MA0152.1.NFATC2|MA0154.1.EBF1|MA0155.1.INSM1|MA0156.1.FEV|MA0158.1.HOXA5|MA0160.1.**NR4A2**|MA0161.1.NFIC|MA0162.1.Egr1|MA0442.1.SOX10|MA0090.1.TEAD1|MA0087.1.Sox5|MA0088.1.znf143|MA0089.1.NFE2L1::MafG	**73.134386**	0.990735385	**14.05416**	0.939636945

Differentially expressed genes between PFO vs PT, nearby a TFBS with a significant turnover

**SYMBOL**	**TF**	**chr**	**Phascons start**	**Phascons end**	**Gene start**	**Gene end**
"CA10"	MA0089.1.NFE2L1::MafG|MA0122.1.Nkx3-2|MA0068.1.Pax4|MA0033.1.FOXL1|MA0154.1.EBF1|MA0032.1.FOXC1|MA0027.1.En1	17chr	48538105	48538371	47062672	47592367
"WBSCR17"	MA0146.1.Zfx	chr7	71663666	71663746	70768371	70816520
"MDGA1"	MA0114.1.HNF4A	chr6	38700951	38701333	37708271	37710012
"PDE4DIP"	MA0156.1.FEV|MA0055.1.Myf	chr1	143232099	143232519	143388229	143751301
"SORCS1"	MA0046.1.HNF1A	chr10	108799369	108799525	108323410	108479862
"COL12A1"	MA0033.1.FOXL1|MA0006.1.Arnt::**Ahr**	chr6	76519878	76519905	75850761	75851940
"FLJ41649"	MA0009.1.T|MA0037.1.GATA3	chr6	41184784	41184851	40433330	40435198
"NFE2L3"	MA0073.1.RREB1	chr7	26986036	26986282	26192836	26193270
"OCA2"	MA0037.1.GATA3|MA0056.1.MZF1_1-4|MA0073.1.RREB1|MA0079.2.SP1|MA0105.1.NFKB1|MA0057.1.MZF1_5-13|MA0068.1.Pax4	chr15	26574366	26574521	25673627	26018061
"OCA2"	MA0105.1.NFKB1	chr15	26772538	26772806	25673627	26018061
"PTPN13"	MA0035.2.Gata1, MA0037.1.GATA3, MA0055.1.Myf, MA0067.1.Pax2, MA0117.1.Mafb MA0051.1.**IRF2,** MA0089.1.NFE2L1::MafG, MA0050.1.IRF1, MA0111.1.Spz1, MA0063.1.Nkx2-5, MA0018.2.CREB1 MA0133.1.BRCA	chr4	88755260	88755453	87734908	87955326

"PTPN13"	MA0035.2.Gata1 MA0100.1.**Myb** MA0133.1.BRCA1 MA0067.1.Pax2 MA0055.1.**Myf** MA0089.1.NFE2L1::MafG MA0037.1.GATA3 MA0111.1.Spz1	chr4	88755553	88755687	87734908	87955326
"SORCS1"	MA0046.1.HNF1A	chr10	108799369	108799525	108323410	108479862
"ELAVL2"	MA0163.1.PLAG1	chr9	24344040	24344156	23680104	23811755
"LPHN3"	MA0028.1.ELK1|MA0088.1.znf143|MA0090.1.TEAD1|MA0136.1.ELF5|MA0149.1.EWSR1-FLI1|MA0081.1.SPIB|MA0079.2.SP1	chr4	60972079	60972149	61749969	62620762
"NLGN1"	MA0115.1.NR1H2::RXRA	chr3	176112355	176112494	174785170	175483607
"PARP8"	MA0162.1.Egr1|MA0006.1.Arnt::**Ahr**|MA0042.1.FOXI1	chr5	51045261	51045318	50125832	50173926
"RPH3A"	MA0042.1.FOXI1|MA0073.1.RREB1|MA0084.1.SRY	chr12	112442592	112442646	111714038	111821065
"XYLT1"	MA0151.1.ARID3A	chr16	18252002	18252258	17108754	17260859
"ZNF438"	MA0073.1.RREB1|MA0006.1.Arnt::**Ahr**|MA0042.1.FOXI1|MA0068.1.Pax4|MA0162.1.Egr1	chr10	31856208	31856367	31173572	31174200
"ZNF488"	MA0061.1.NF-kappaB|MA0068.1.Pax4|MA0073.1.RREB1|MA0107.1.RELA|MA0133.1.BRCA1|MA0142.1.Pou5f1|MA0158.1.HOXA5	chr10	47807445	47807627	47993159	47993870

"ANKRD18A"	MA0002.2.RUNX1|MA0028.1.ELK1|MA0027.1.En1|MA0063.1.Nkx2-5|MA0014.1.Pax5|MA0017.1.**NR2F1**|MA	chr9	39475371	39475990	38584546	38584999
"EFNA5"	MA0070.1.**PBX1**|MA0038.1.Gfi	chr5	105771562	105771788	106740493	106741397
"LOC440570"	MA0019.1.Ddit3::Cebpa|MA0122.1.Nkx3-2	chr1	17890027	17890129	17072149	17073164
"RSPO3"	MA0091.1.TAL1::TCF3	chr6	127998066	127998096	127559192	127559871

Supplementary table 4

SYMBOL	GENENAME	TF with a TFBS turnover FDR<0,05
"CA10"	carbonic anhydrase X	Nkx3-2, Pax4, FOXL1, EBF1, FOXC1, En1,NFE2L1::MafG
"WBSCR17"	Williams-Beuren syndrome chromosome region 17	Zfx
"MDGA1"	MAM domain containing glycosylphosphatidylinositol anchor 1	HNF4A
"PDE4DIP"	phosphodiesterase 4D interacting protein	FEV, Myf
"SORCS1"	sortilin-related VPS10 domain containing receptor 1	HNF1A
"COL12A1"	collagen, type XII, alpha 1	FOXL1, **Arnt::Ahr**
"FLJ41649"	hypothetical LOC401260	GATA3,
"NFE2L3"	nuclear factor (erythroid-derived 2)-like 3	RREB1
"OCA2"	oculocutaneous albinism II	GATA3, MZF1_1-4, RREB1, SP1, NFKB1, MZF1_5-13, Pax4, NFKB1
"PTPN13"	protein tyrosine phosphatase, non-receptor type 13 (APO-1/CD95 (Fas)-associated phosphatase)	Gata1, GATA3, **Myf**, Pax2, Mafb, **IRF2**, IRF1, Spz1, Nkx2-5, CREB1, NFE2L1::MafG, **Myb**, BRCA1
"ELAVL2"	ELAV (embryonic lethal, abnormal vision, Drosophila)-like 2 (Hu antigen B)	PLAG1
"LPHN3"	latrophilin 3	ELK1, znf143, TEAD1, ELF5, EWSR1-FLI1, SPIB, SP1
"NLGN1"	neuroligin 1	NR1H2::RXRA
"PARP8"	poly (ADP-ribose) polymerase family, member 8	Egr1, FOXI1, **Arnt::Ahr**
"RPH3A"	rabphilin 3A homolog (mouse)	FOXI1, RREB1, SRY
"XYLT1"	xylosyltransferase I	ARID3A
"ZNF438"	zinc finger protein 438	RREB1, FOXI1, Pax4, Egr1, **Arnt::Ahr**
"ZNF488"	zinc finger protein 488	NF-kappaB, Pax4, RREB1, RELA, BRCA1, Pou5f1, HOXA5
"ANKRD18A"	ankyrin repeat domain 18A	RUNX1, ELK1, En1, Nkx2-5, Pax5, **NR2F1**, MZF1_5-13, SRY, znf143, YY1, ETS1, AP1, CEBPA, Hltf, ESR1, SP1, SPI1, SPIB, Pdx1, BRCA1, ELF5, Esrrb, ARID3A, NFATC2, HNF1B, EBF1, FEV, **NR4A2**, SOX10, **NR3C1**, Mafb, Nkx3-2, FOXL1, MZF1_1-4, NFE2L1::MafG, Hand1::Tcfe2a
"EFNA5"	ephrin-A5	**PBX1**, Gfi
"LOC440570"	LOC440570	Nkx3-2, Ddit3::Cebpa
"RSPO3"	R-spondin 3 homolog (Xenopus laevis)	TAL1::TCF3

Supplementary table 5

Differentially expressed genes between PFOvsPT, differentially regulated by Hum/Chimp FoxP2

SYMBOL	PFOvsPT.d	PFOvsPT.FC	PFOvsPT.q	Hum/Chimp Foxp2 differentially regulated genes	Hum/Chimp Foxp2 similarly regulated genes
FSTL5	-9.13405901	5.144152677	0	FSTL5	NA
TFAP2D	-3.50782293	3.658017759	0	TFAP2D	NA
PCDH17	-5.06712243	2.341529223	0	PCDH17	NA
PROM1	-4.64610054	2.243684276	0	PROM1	NA
FGF14	-4.16133602	1.89013512	0	FGF14	NA
CDCA7L	-3.20491701	1.821544626	0	CDCA7L	NA
HIST1H2AC	-3.22817879	1.750763743	0	HIST1H2AC	NA
IGFBP3	-3.73725018	1.622755315	0	IGFBP3	NA
FAM181B	-2.44839686	1.447237007	0	MGC33846	NA
SNCAIP	-2.39993038	1.444568076	0	SNCAIP	NA
ZCCHC12	-2.49345568	1.400604405	0	ZCCHC12	NA
B3GNT2	-2.23637806	1.331500784	0	B3GNT1	NA
LRRC8C	-1.96587673	1.283370606	0.192062394	LRRC8C	NA
FAM43A	-1.87846026	1.282273341	0.192062394	FAM43A	NA
MAOB	-1.57097754	1.20465675	0.884199553	MAOB	NA
CACNB2	1.758489199	0.803207464	0.569217175	CACNB2	NA
C6orf48	2.036054613	0.797705279	0.113295232	C6orf48	NA
LRRC4C	1.772317163	0.783808549	0.569217175	LRRC4C	NA
DPYD	1.91158004	0.757162176	0.192062394	DPYD	NA
ZNF702P	2.620344205	0.708615727	0	ZNF702	NA
MGST1	1.860475221	0.701691591	0.192062394	MGST1	NA

IGFBP4	2.382813383	0.684181343	0	IGFBP4	NA
EFNB2	2.068676182	0.67820706	0.113295232	EFNB2	NA
FRZB	2.87648549	0.666397665	0	FRZB	NA
GOLSYN	3.318493885	0.652824912	0	FLJ20366	NA
FLJ35409	3.748400979	0.578889729	0	FLJ35409	NA
MRAP2	2.394926779	0.513374376	0	C6orf117	NA
RNF182	4.710640808	0.479628608	0	RNF182	NA

Differentially expressed genes between PFOvsPT, similarly regulated by Hum/Chimp FoxP2

SYMBOL	PFOvsPT.d	PFOvsPT.FC	PFOvsPT.q	Hum/Chimp Foxp2 differentially regulated genes	Hum/Chimp Foxp2 similarly regulated genes
VSTM2L	-7.69224838	5.181405401	0	NA	C20orf102
CBLN4	-2.64785393	1.618761792	0	NA	CBLN4
CDH12	-3.09316342	1.701936008	0	NA	CDH12
FRMD3	2.812540314	0.667817242	0	NA	FRMD3
IL7	-4.01132728	3.629542474	0	NA	IL7
LMO4	-5.43330418	2.097623295	0	NA	LMO4

NEUROG2	-9.53696955	4.355236891	0	NA	NEUROG2
NNAT	-3.60534578	1.521518938	0	NA	NNAT
RFTN1	-2.45953464	1.379481017	0	NA	RAFTLIN
IGSF21	1.819825649	0.764397197	0.192062394	NA	IGSF21
STX3	1.932884244	0.769041276	0.192062394	NA	STX3A
CDH11	-1.68075954	1.267706284	0.569217175	NA	CDH11

Differentially expressed genes between 17vs19, differentially regulated by Hum/Chimp FoxP2

SYMBOL	17vs19.d	17vs19.FC	17vs19.q	Hum/Chimp Foxp2 differentially regulated genes	Hum/Chimp Foxp2 similarly regulated genes
RSRC1	-3.56506296	1.713336184	0	RSRC1	NA
EYA1	-3.22782472	1.505148939	0	EYA1	NA
H2BFS	-3.08914357	1.466974572	0	H2BFS	NA
TMEM100	-3.00072485	1.605912561	0	TMEM100	NA
DCN	-2.79332946	1.41786959	0	DCN	NA
FAM43B	3.968117564	0.63173163	0	FAM43B	NA
EDNRA	-2.51439268	1.506405019	0.154086839	EDNRA	NA
MAN1A1	-2.34859792	1.474033488	0.154086839	MAN1A1	NA
PRPH2	2.82645521	0.70562354	0.154086839	PRPH	NA
PHACTR2	-2.19176152	1.276628492	0.52040198	PHACTR2	NA
TMTC2	-2.17682852	1.459025452	0.52040198	TMTC2	NA
HIST1H2BH	-2.11428146	1.357029526	0.52040198	HIST1H2BH	NA
RDX	-2.01184849	1.227806594	0.52040198	RDX	NA
ISLR2	-1.97589554	1.330749641	0.52040198	ISLR2	NA

TIMP1	-1.97274759	1.281802248	0.52040198	TIMP1	NA

Differentially expressed genes between PFOvsPT, similarly regulated by Hum/Chimp FoxP2

SYMBOL	17vs19.d	17vs19.FC	17vs19.q	Hum/Chimp Foxp2 differentiall y regulated genes	Hum/Chimp Foxp2 similarly regulated genes
EGR1	-3.25867222	1.70147338	0	NA	EGR1
VIP	3.145241633	0.728152587	0	NA	VIP
LBH	-3.18915584	1.329253496	0	NA	LBH
BOC	-2.80684746	1.276563183	0	NA	BOC
GNG8	2.09394221	0.663116055	0.52040198	NA	GNG8
